# N4-Substituted Piperazinyl Norfloxacin Derivatives with Broad-Spectrum Activity and Multiple Mechanisms on Gyrase, Topoisomerase IV, and Bacterial Cell Wall Synthesis

**DOI:** 10.1021/acsbiomedchemau.3c00038

**Published:** 2023-08-30

**Authors:** Ahmed M. Kamal El-sagheir, Ireny Abdelmesseh Nekhala, Mohammed K. Abd El-Gaber, Ahmed S. Aboraia, Jonatan Persson, Ann-Britt Schäfer, Michaela Wenzel, Farghaly A. Omar

**Affiliations:** † Medicinal Chemistry Department, Faculty of Pharmacy, Assiut University, Assiut 71526, Egypt; ‡ Division of Chemical Biology, Department of Life Sciences, Chalmers University of Technology, 412 96 Gothenburg, Sweden; § Center for Antibiotic Resistance Research in Gothenburg (CARe), 405 30 Gothenburg, Sweden

**Keywords:** fluoroquinolones, norfloxacin, multidrug resistance, molecular docking, bacterial cytological profiling, cell wall synthesis

## Abstract

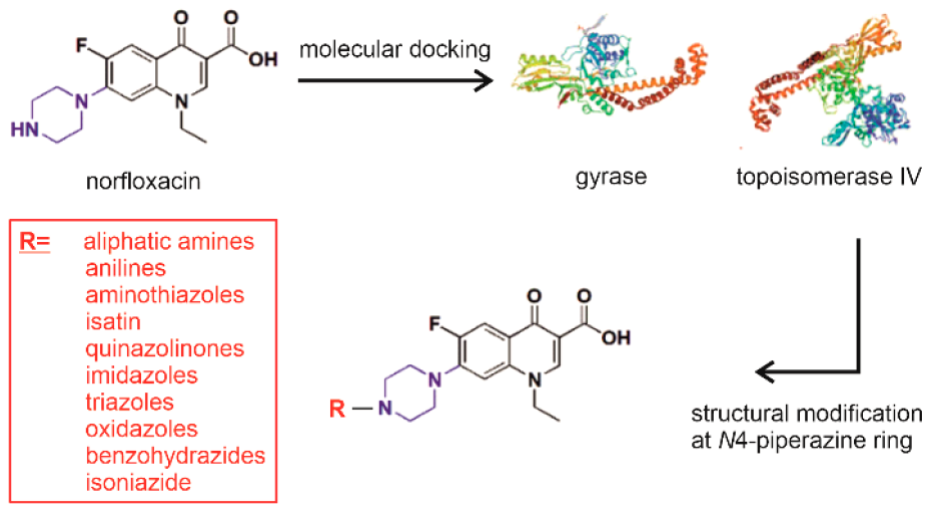

Fluoroquinolones are an important class of antibiotics with broad-spectrum antibacterial and antitubercular activity. Here, we describe the design and synthesis of a series of 38 *N*4-substituted piperazinyl norfloxacin derivatives. Their activity and mechanism of action were characterized using *in silico*, *in vitro*, and *in vivo* approaches. Several compounds displayed interesting activities against both Gram-negative and Gram-positive bacteria, and few displayed antimycobacterial activity, whereby some were as potent as norfloxacin and ciprofloxacin. Molecular docking experiments suggested that the new derivatives inhibit both DNA gyrase and DNA topoisomerase IV in a similar manner as norfloxacin. Selecting the most promising candidates for experimental mode of action analysis, we confirmed DNA gyrase and topoisomerase IV as targets of all tested compounds using enzymatic *in vitro* assays. Phenotypic analysis of both *Escherichia coli* and *Bacillus subtilis* confirmed a typical gyrase inhibition phenotype for all of the tested compounds. Assessment of possible additional targets revealed three compounds with unique effects on the *B. subtilis* cell wall synthesis machinery, suggesting that they may have an additional target in this pathway. Comparison with known cell wall synthesis inhibitors showed that the new compounds elicit a distinct and, so far, unique phenotype, suggesting that they act differently from known cell wall synthesis inhibitors. Interestingly, our phenotypic analysis revealed that both norfloxacin and ciprofloxacin displayed additional cellular effects as well, which may be indicative of the so far unknown additional mechanisms of fluoroquinolones.

## Introduction

Fluoroquinolones are an important class of antibiotics with broad-spectrum activity against Gram-positive, Gram-negative, and mycobacterial pathogens. To date, three fluoroquinolones, namely, ciprofloxacin, moxifloxacin, and levofloxacin, are on the WHO’s list of essential medicines, the latter two for treatment of tuberculosis (https://list.essentialmeds.org/). The original quinolones, e.g., nalidixic acid, possessed poor pharmacokinetics, limited activity against Gram-positive bacteria, and a tendency to quickly develop resistance.^1^ Norfloxacin was the first patented fluoroquinolone and paved the way for a range of antibacterial and antitubercular drugs with significant improvements of their pharmacokinetic profile, potency, and activity spectrum.^1−3^ Quinolones and fluoroquinolones have been and still are continuously improved, resulting in different “generations” with improved activity, spectrum, side effects, and resistance frequency. The latest, fourth generation inhibits two related bacterial enzymes, DNA gyrase and DNA topoisomerase IV, which are involved in introducing and relaxing supercoils during DNA replication and nucleoid separation.^4^

In Gram-negative bacteria, such as *Escherichia coli*, and in mycobacteria like *Mycobacterium tuberculosis*, the primary target of fluoroquinolones is DNA gyrase, while topoisomerase IV serves as the secondary target. This is reversed in Gram-positive bacteria such as *Staphylococcus aureus*, where DNA topoisomerase IV is the more susceptible target. Inhibition of these enzymes by fluoroquinolones leads to stalling of the enzyme-DNA complex, resulting in disruption of DNA replication and nucleoid separation, which leads to DNA damage in the form of double strands breaks, nucleoid packing defects, impaired cell division, and ultimately cell death.^5−7^

Since the development of norfloxacin, fluoroquinolones have gained more importance in the therapy of bacterial infections due to their broad antibacterial spectrum and excellent bioavailability. However, the incidence of quinolone resistance has been steadily rising. The mechanism of active site resistance to quinolones is associated with mutations in the *gyrA* and *parC* genes, encoding the A subunits of DNA gyrase and topoisomerase IV, respectively.^8^ Mutations in these genes result in amino acid substitutions that structurally change the target protein and, subsequently, the drug-binding affinity of the enzyme.^9^ Other resistance mechanisms involve decreased uptake mediated by cell envelope modifications and increased efflux due to overexpression of drug efflux pumps.^10^ Therefore, efforts have been undertaken to increase the potency of fluoroquinolones and develop resistance-breaking derivatives.

**Figure 1:**
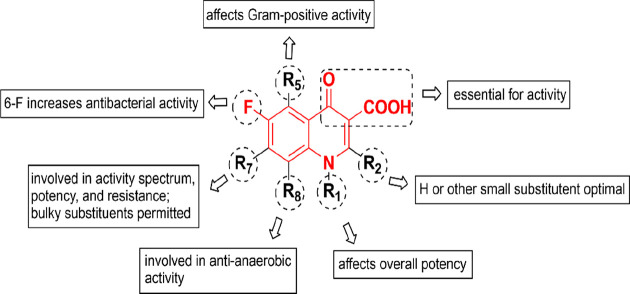
Possible structural modifications of the fluoroquinolone lead structure. Red: 4-oxo-1,4-dihydroquinoline-3-carboxylic acid skeleton; black: possible substituents. Adapted with permission under a Creative Commons license [CC-BY 4.0] from ref (7). Copyright 2020 MDPI.

Structure–activity relationship (SAR) studies showed that the 4-oxo-1,4-dihydroquinoline-3-carboxylic acid skeleton is an essential pharmacophore for binding to DNA gyrase^11^ and that the 6-fluoro substituent augments the antibacterial activity.^12^ While the 7-piperazine ring substituent increases the activity against Gram-negative bacteria, alkylation of the *N*4-piperazine ring enhances the activity against Gram-positive organisms. The C-7 substituent, the only position, where substitution of a bulky functional group is permitted, greatly influences their antibacterial potency, spectrum, and safety.^13,14^ Moreover, the C-7 substituent has been shown to play a critical role in the design of resistance-breaking fluoroquinolones (Figure 1). A large number of existing fluoroquinolone derivatives have been synthesized by introduction of an additional functional moiety on the *N*4-piperazine ring to increase the overall lipophilicity of the molecule, some of which were found to exhibit enhanced antibacterial activity.^15^

**Figure 2:**
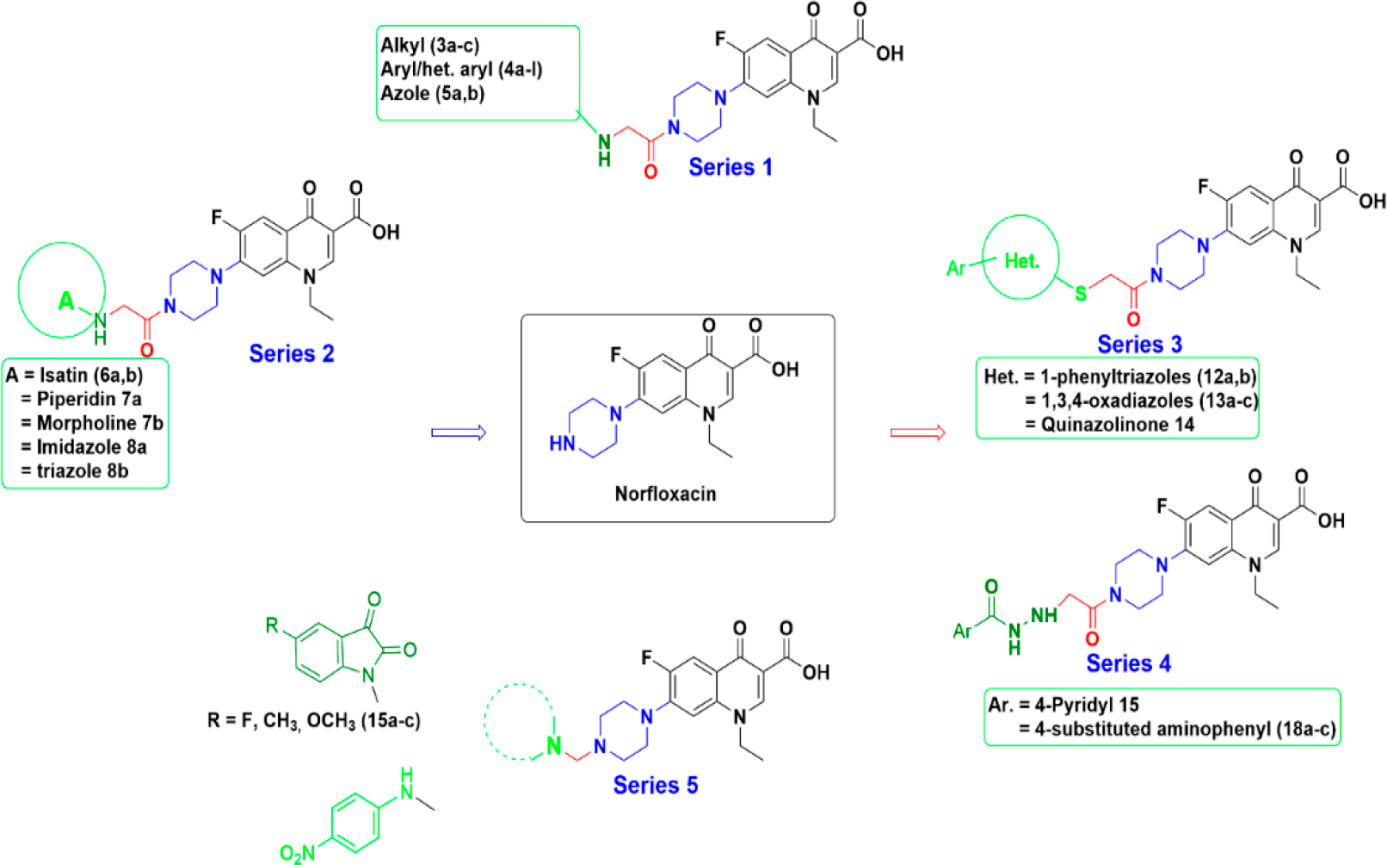
Designed series (1–5) of *N*4-substituted piperazinyl norfloxacin derivatives. Black: basic quinolone nucleus; blue: piperazine moiety; red: linker; green: added structural moieties.

Based on the reported SAR of fluoroquinolones^9,18^ and on our work,^16,17^ we set out to design and synthesize novel *N*4-substituted piperazinyl derivatives of norfloxacin in an attempt to improve its potency and possibly enable new target interactions. Guided by a primary molecular docking study, a broad range of diverse aliphatic, cyclic, aromatic, and heterocyclic substituents were selected, including aliphatic amines, anilines, and nitrogen-containing heterocycles such as aminothiazoles, isatin, quinazolinones, imidazoles, triazoles, oxadiazoles, benzohydrazides, and isoniazid, which were added to norfloxacin using *N*-acetyl, thioacetyl, or methylene linkers (Figure 2). These substituents were chosen to encompass a wide range of hydrophobic, electronic, and topological properties to enable studying the effects of structural changes at that position on the antibacterial profile. This broad strategy promises to yield innovative norfloxacin derivatives with the potential to combat antibacterial resistance. Importantly, the newly introduced *N*4-substituents might afford extra binding potentials either for additional interaction with the target enzymes gyrase and topoisomerase IV or with secondary antibacterial targets, adding an additional antibacterial functionality to the molecule.

## Experimental Section

Experimental details can be found in section 9 of the Supporting Information. Synthesis of intermediates and test compounds is described in Texts S9, S10 and Scheme S6. Yields and reaction times of compounds are displayed in Table S14. Bacterial test strains are listed in Table S15. Testing of biological activity, molecular modeling, mode of action studies, and HPLC analysis of compounds are described in Texts S11–S15.

## Results and Discussion

### Compound Synthesis

N4-Substituted piperazinyl norfloxacin derivatives (Figure 2, Series 1–5) were synthesized as depicted in Schemes S1–S5. The chemical structures of the prepared compounds were elucidated by elemental analysis and spectral techniques (see Figures S1–S46 for corresponding ^1^H NMR and ^13^C NMR data and Figure S47 for elemental analysis). Details on the chemical synthesis and compound characterization for each series are described in Text S1.

### Antibacterial activity

Antibacterial activity of the newly synthesized norfloxacin derivatives **3**–**21** was evaluated against six wild type test strains (Table 1): *E. coli* W3110, *Pseudomonas aeruginosa* PAO1, and *Klebsiella pneumoniae* ATCC 10031^19^ as Gram-negative test strains, *S. aureus* CCUG1800T and *Enterococcus faecalis* ATCC 19433^20^ as Gram-positive test strains, and *M. tuberculosis* MC26020 (live-attenuated strain for use in BSL-II laboratories)^21^ as a model for mycobacteria. Additionally, compounds were tested against two fluoroquinolone-resistant strains, namely a norfloxacin-resistant clinical isolate of *E. coli* and a methicillin-resistant *S. aureus* strain (MRSA) (ATCC43300).^22^

**Table 1:** MICs of Norfloxacin Derivatives in μM^a^

	Gram-negative strains				Gram-positive strains			mycobacterial strain
ID	*E. coli* (W3110)	*E. coli*^b^	*P. aeruginosa* (PAO1)	*K. pneumoniae* (ATCC 10031)	*S. aureus* (CCUG1800T)	*S. aureus* (ATCC 43300) *MRSA*^b^	*E. faecalis* (ATCC 19433)	*M. tuberculosis* (MC26020)
INH								1.82
Nor	0.39	50.1	6.26	9.39	3.13	100.21	4.69	1.56
Cip	0.37	193.17	3.01	7.54	3.01	96.58	6.03	2.26
**2**	6.31	>1293.54	192	121.27	**2.52**	161.69	6.31	>1293.54
**3a**	79.12	1265.94	632.97	1265.94	4.94	>1265.94	9.89	>1265.94
**3b**	76.47	>1223.53	229.41	152.94	**2.38**	>1223.53	7.16	152.94
**3c**	17.45	**34.91**	279.33	69.83	**2.18**	139.66	**2.18**	104.75
**4a**	8.84	70.72	141.44	35.36	**2.21**	>1131.54	**4.42**	141.44
**4b**	30.11	>963.54	722.66	>963.54	**1.88**	>963.54	7.52	3.76
**4c**	65.71	1051.5	>1051.5	>1051.5	**2.05**	>1051.5	5.13	>1051.5
**4d**	514.59	257.29	>1029.18	257.29	**2.05**	1029.18	**4.02**	12.06
**4e**	8.29	66.32	663.21	**8.29**	**2.07**	**66.32**	**2.07**	>1061.13
**4f**	132.64	>1061.13	663.21	1061.13	**2.07**	>1061.13	**4.14**	>1061.13
**4g**	16.58	66.32	530.56	66.32	**2.07**	132.64	16.58	8.29
**4h**	128.9	>1031.23	1031.23	>1031.23	4.02	**32.22**	6.04	**1.51**
**4i**	34	68.01	408.1	544.13	**2.12**	102.02	17	272.06
**4j**	64.71	>1035.36	129.42	>1035.36	**2.02**	>512	258.84	258.84
**4k**	65.51	>1048.19	524.09	>1048.19	**2.04**	>1048.19	32.75	98.26
**4l**	282.26	282.26	1129.07	282.26	8.82	>1129.07	17.64	211.7
**5a**	34.82	**34.82**	139.28	**6.52**	**2.17**	557.13	**4.35**	13.05
**5b**	>1004.8	>1004.8	>1004.8	>1004.8	62.8	>1004.8	31.4	>1004.8
**16**	64.45	**32.22**	515.61	12.08	**2.01**	**64.45**	**2.01**	**1**
**6a**	63.18	1010.89	1010.89	1010.89	**1.97**	>1010.89	**1.97**	126.36
**6b**	>874.64	81.99	>874.64	>874.64	6.83	>874.64	10.24	109.33
**7a**	35.99	>1151.85	>1151.85	143.98	8.99	575.92	**4.49**	71.99
**7b**	71.67	143.34	860.08	>1146.77	4.47	286.69	8.95	1.67
**8a**	4.67	74.86	149.73	**9.35**	**2.33**	299.46	14.03	2.33
**8b**	33.79	135.18	135.18	512	**2.11**	**16.89**	**2.11**	>1081.51
**12a**	3.72	**21.03**	11.92	190.81	**2.98**	>672.98	23.85	381.63
**12b**	23.85	381.63	11.92	>672.98	**1.49**	>672.98	23.85	>672.98
**13a**	11.66	>746.7	746.7	>746.7	11.66	**23.33**	64.66	>746.7
**13b**	>746.7	>746.7	746.7	373.35	**2.91**	>746.7	5.83	>746.7
**13c**	>758.37	189.59	758.37	>758.37	11.84	>758.37	11.84	256
**14**	238.11	476.22	>952.45	128	14.88	>952.45	29.76	>952.45
**18a**	6.21	>795.5	198.87	74.57	**1.55**	>795.5	**4.66**	596.62
**18b**	24.85	>795.5	198.87	795.5	**1.55**	>795.5	6.21	>795.5
**18c**	>808.75	**25.27**	>808.75	>808.75	6.31	101.09	25.27	>808.75
**20a**	3.02	**16.11**	17.12	**8.05**	8.05	>1031.3	**3.02**	64.45
**20b**	5.07	>1039.59	16.24	**3.04**	32.48	259.89	6.09	129.94
**20c**	4.91	1006.88	62.93	503.44	31.46	**15.73**	251.72	>1006.88
**21**	**0.266**	**17.04**	**2.13**	**4.26**	**2.13**	**34.08**	**2.13**	6.39

a MICs lower than that of norfloxacin against the respective strain are indicated in bold.

b Norfloxacin-resistant strains. INH = isoniazid, Nor = norfloxacin, Cip = ciprofloxacin.

Most compounds showed activity against at least one of the test strains in the low micromolar range, whereby the *S. aureus* type strain CCUG1800T was most susceptible. While only one compound was clearly below the clinical breakpoint of ciprofloxacin, defined as 0.25 mg/L by EUCAST (https://www.eucast.org/clinical_breakpoints) (compound **21**, MIC = 0.266 μM → 0.125 mg/L against *E. coli* W3110), several compounds showed higher activity than their parent compound norfloxacin (highlighted in bold in Table 1). None of the compounds came close to clinical breakpoint values against the fluoroquinolone-resistant test strains, yet several compounds were still more active than norfloxacin and ciprofloxacin against the resistant Gram-negative (**3c**, **5a**, **16**, **12a**, **18c**, **20a**, **21**) and Gram-positive test strains (**4e**, **4h**, **16**, **8b**, **13a**, **20c**, **21**). This is a good starting point for further improvement of the lead structure and lets us draw conclusions about which of our *N*4-piperazine substitutions were beneficial for antibacterial activity.

For the Gram-negative test strains, compounds **5a**, **20a**, **20b**, **21**, **18c**, and **12a** displayed the highest activity. Notably, compound **21** was more active against norfloxacin against all tested strains, with the exception of *M. tuberculosis*. These results show that Mannich bases of norfloxacin (series **5**) exerted the highest activity against Gram-negative bacteria, suggesting that both isatin and *p*-nitrophenylamino moieties improve the activity of norfloxacin against these pathogens. This is possibly achieved through inducing additional interactions at the binding site of the DNA gyrase enzyme and/or enhancing lipophilicity, which is correlated with the ability of fluoroquinolones to cross the bacterial cell envelope.^10,23^

For the Gram-positive test strains, derivatives **4b**, **4e**, **6a**, **8b**, **20c**, and **18a** exhibited the highest antibacterial activity. Compounds **20c** and **8b** stood out by their enhanced activity against fluoroquinolone-resistant MRSA, being 6.3-fold and 5.9-fold more active than norfloxacin. Our results suggest that both the addition of *N*-acetyl substituents and the formation of Mannich bases at *N*4 of the piperazine ring enhance the activity against Gram-positive bacteria, which was observed mainly in series **1,2**, and **5** and can be considered a promising starting point for the development of new effective norfloxacin derivatives against resistant Gram-positive strains.

The antimycobacterial activity of isoniazid-norfloxacin hybrid **16** was similar to that of isoniazid and norfloxacin, indicating that the molecular hybridization of isoniazid with norfloxacin at least did not interfere with antibacterial activity. However, none of the synthesized compounds showed strongly improved activity compared to norfloxacin and isoniazid.

### *In Silico* Analysis

#### Molecular Modeling of Compound Properties

Following the assessment of antibacterial activity, we performed different molecular modeling studies on the synthesized compound set, including quantitative structure–activity relationship (QSAR) analysis (see Texts S2 and S3, Tables S1–S6, Figure S48), prediction of physicochemical parameters signifying drug-likeness (see Text S4, Table S7), prediction of pharmacokinetics and pharmacodynamics properties using absorption, distribution, metabolism, and excretion (ADME), and toxicity predictions (see Texts S5–S8, Tables S8 and 9). Cytotoxicity was also experimentally tested for two exemplary compounds, **4a** and **4e**, which were selected for being the most potent inhibitors of DNA gyrase and topoisomerase *in vitro* (see section *In Vitro* Inhibition of DNA Gyrase and Topoisomerase IV). Toxicity was tested against human neuroblastoma (SH-SY5Y) cell lines using a 3-[4,5-dimethylthiazol-2-yl]-2,5-diphenyl tetrazolium bromide (MTT) assay.^46,47^ Norfloxacin and the apoptosis-inducing kinase inhibitor staurosporine were used as controls. Compounds **4a** and **4e** showed similar IC_50_ values (45.26 ± 2.48 and 37.46 ± 1.68 μM, respectively), which were comparable to that of norfloxacin (48.23 ± 3.92 μM) and significantly higher than that of staurosporine (19.52 ± 0.97).

#### Molecular Docking

Molecular docking studies were performed on *S. aureus* DNA gyrase and *Acinetobacter baumannii* DNA topoisomerase IV. To this end, we picked the most interesting compounds based on structural properties and MIC data: **4b**, **4e**, **6a**, **12b**, **16**, **18a**, and **21** for DNA gyrase and **4a**, **4e**, **8a**, **12a**, **18a**, and **21** for DNA topoisomerase IV. Here, we discuss **4b** and **18a** bound to gyrase, and **12a** and **21** bound to topoisomerase IV. Data on the remaining compounds as well as norfloxacin are available as supporting material (Text S8, Figures S50–S61, Table S10).

**Figure 3:**
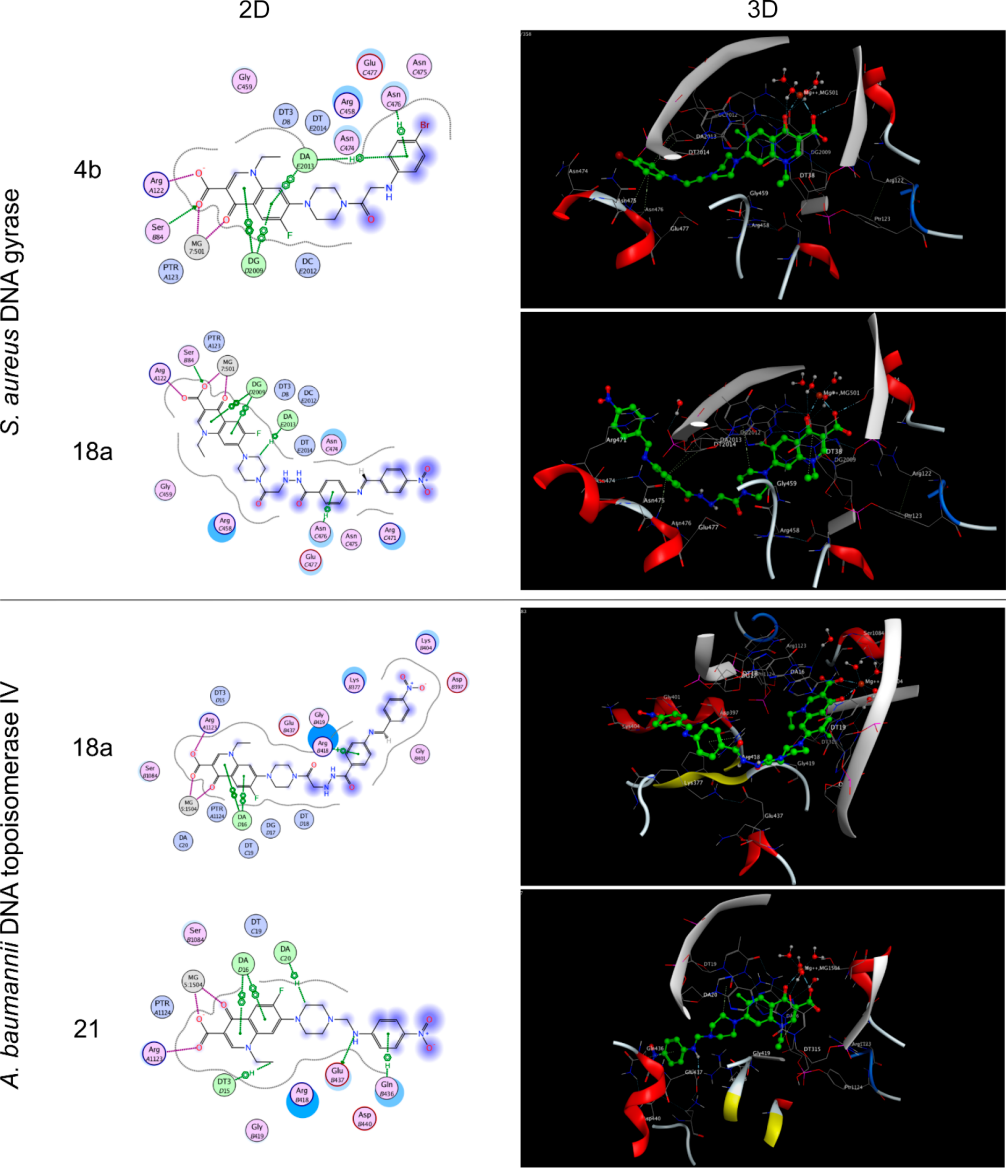
2D and 3D molecular docking studies of compounds **4b** and **18a** on *S. aureus* DNA gyrase (PDB: 5CDQ) and compound **18a** and **21** on *A. baumannii* DNA topoisomerase IV (PDB: 2XKK).

The first docking study was performed on the three-dimensional crystal structure of *S. aureus* DNA gyrase complexed with moxifloxacin (UniProt accession ID: Q99XG5, PDB code: 5CDQ)^24^ using the MOE 2020.01 software. The binding modes of compounds **4b** and **18a** (docking scores: −12.21 and −12.76 kcal/mol, respectively) are illustrated in Figure 3 (top). For these compounds, the oxygen of the carboxylic carbonyl group formed a H-bond with the Ser B84 residue. The ketonic and carboxylic carbonyl groups formed two coordination bonds with Mg^2+^ through their oxygen atoms with average lengths of 2.47 and 2.28 Å, respectively. In addition, the oxygen of the carboxylic acid group interacted with the Arg A122 residue through H-bonding, and the quinolone ring of compound **4b** interacted with nitrogenous bases like DA E2013 and DG D2009 through π–π stacking.

In addition to these main interactions, the newly designed compounds formed extra binding interactions with other amino acid residues, mediated by the structural moieties added to the *N*4-piperazine ring of norfloxacin. For compound **4b**, π–cation and π–H bond interactions were formed between the aromatic ring of 4-bromophenyl and the Asn C476 residue. In compound **18a**, two π–H bonds were formed between the phenyl ring of the hydrazide moiety and the Asn C476 and Gly B117 residues. These interactions might stabilize the compound–enzyme–DNA complex, which further accounted for a high binding score and may be a predictor of good inhibitory activity.

The second docking study was performed on the three-dimensional crystal structure of *A. baumannii* topoisomerase IV complexed with moxifloxacin (UniProt accession ID: B0VP98, PDB code: 2XKK).^25^ The binding modes of compounds **18a** and **21** (docking scores of −11.96 and −11.89 kcal/mol, respectively) are illustrated in Figure 3 (bottom), showing that the oxygen of the carboxylic carbonyl group formed a H-bond with the Arg A1123 residue. The ketonic and carboxylic carbonyl groups formed two coordination bonds with Mg^2+^ through their oxygen atoms. Additionally, the quinolone ring of the compounds was involved in an interaction with nitrogenous bases like DA D16 and DA C20 through π–π stacking and π-H bonding. The nitro group of compound **18a** formed a H-bond with Lys B404 and the phenyl ring formed a π-cation interaction with Arg B418. The NH group of compound **21** interacted with the Glu B437 residue through a H-bond, and the phenyl ring formed a π-H bond with the Gln *B*436 residue.

Taken together, our molecular docking studies revealed the ability of the tested *N*4-piperazinyl norfloxacin derivatives to interact with the key amino acids in the active sites of DNA gyrase and topoisomerase IV in a manner similar to norfloxacin (see Figures S51 and S56).

### *In Vitro* Inhibition of DNA Gyrase and Topoisomerase IV

**Figure 4:**
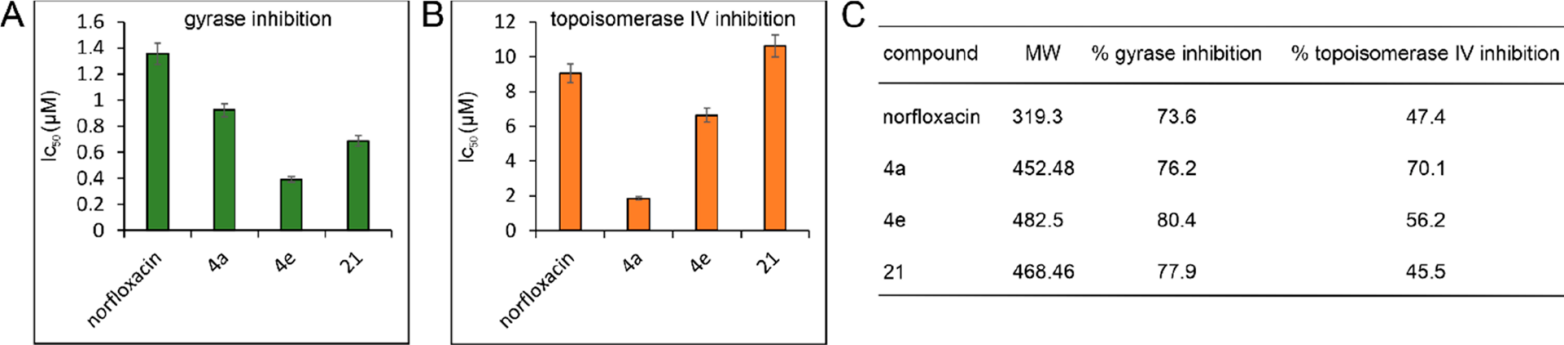
*In vitro* inhibition of DNA gyrase and DNA topoisomerase IV. (A) IC_50_ values against *E. coli* DNA gyrase. (B) IC_50_ values against *E. coli* topoisomerase IV. Error bars represent the standard deviation of 2 replicates. (C) Percent inhibition at 10 μM of the different compounds.

Following *in silico* analysis, we proceeded to validate the mechanism of action of selected derivatives against purified *E. coli* DNA gyrase and topoisomerase IV in enzymatic *in vitro* assays.^26^ To this end, we selected three promising compounds, **4a** and **4e**, which were among the most active compounds against Gram-positive bacteria, and **21**, which showed the best activity across the whole panel of test strains. Figure 4 shows the resulting IC_50_ values and % inhibition at 10 μM of the respective compounds (see Figures S62 and S63 for raw data). All tested compounds exhibited lower IC_50_ values than those of norfloxacin against DNA gyrase. Compound **21** showed IC_50_ values similar to those of norfloxacin, while **4a** and **4e** had considerably lower IC_50_ values. Similar results were obtained for topoisomerase IV, explaining the higher antibacterial activity of these derivatives compared to norfloxacin.

### *In Vivo* Mechanism of Action in Gram-Negative Bacteria

#### Gyrase Inhibition

We proceeded to confirm the inhibition of gyrase and topoisomerase IV in living bacterial cells. As models we picked *E. coli* as Gram-negative and *Bacillus subtilis* as Gram-positive representative. To this end, we selected the most promising compounds of each series and subjected them to bacterial cytological profiling (BCP). BCP is a rapid and powerful tool to get a first insight into the mechanism of action of an antibiotic.^27^ In the case of fluoroquinolones, this method is particularly powerful, since this group of antibiotics elicits a highly specific gyrase inhibition phenotype, which is characterized by extreme nucleoid condensation into an oval shaped structure at midcell (Figure S64).

**Figure 5:**
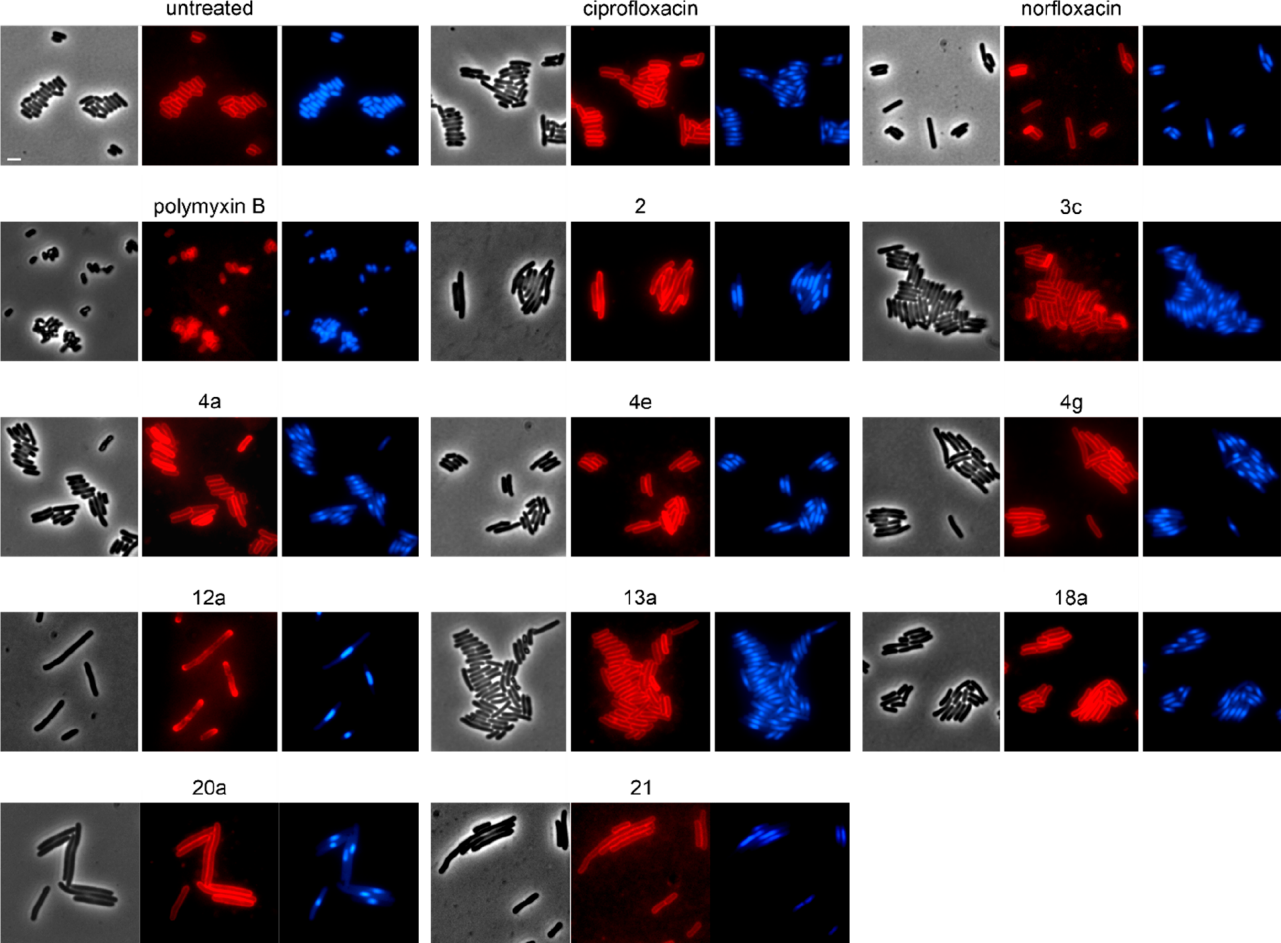
Bacterial cytological profiling of *E. coli* W3110. Cells were treated with 1xMIC of the respective compounds for 1 h prior to staining with fluorescence dyes FM4-64 (membrane, red) and DAPI (nucleoid, blue). Inhibition of DNA gyrase manifests in strongly condensed oval shaped nucleoids. Foci in the FM4-64 stain indicate membrane damage. Note that this membrane dye does not discriminate between the inner and outer membrane and in intact cells accumulates mostly in the outer membrane. Scale bar 2 μm.

Derivatives **2**, **4a**,**e**,**g**, **3c**, **20a**, **21**, **18a**, **12a**, **13a**, **20a**, and **21** were chosen as the most potent compounds against Gram-negative bacteria and subjected to BCP in *E. coli* W3110. Ciprofloxacin and norfloxacin were used as controls for gyrase inhibition.^28^ Polymyxin B was included as control for membrane damage,^4^ since the chemical derivatization of antibiotic lead compounds, especially when targeted toward higher lipophilicity, may result in off-target activity on the bacterial cell membrane. As shown in Figure 5, all tested norfloxacin derivatives showed a clear gyrase inhibition phenotype, apparent by strong nucleoid condensation into midcell localized oval structures.

#### Inner Membrane Activity

Corresponding phase contrast images appeared dark, and membrane stains were smooth, indicating no cell lysis and no membrane damage. However, FM4-64, like most membrane dyes, stains the outer membrane of *E. coli*, at least unless the outer membrane is permeabilized allowing the dye access to the inner membrane. It is therefore difficult to distinguish between inner and outer membrane damage using membrane dyes alone. To more accurately assess possible inner membrane damage, we additionally examined *E. coli* BCB472 expressing a green-fluorescent protein (GFP) fusion to the membrane protein GlpT, which can be used as a proxy to visualize the inner membrane in a dye-independent manner.^29^ None of the tested derivatives affected the localization of this protein suggesting that they do not damage the inner membrane of *E. coli* (Figure S65). Table S11 shows a summary of the BCP results and their interpretation.

#### Outer Membrane Activity

To assess whether the compounds affect outer membrane integrity, we performed synergy assays with mupirocin (Table S12). This antibiotic is able to inhibit the *E. coli* isoleucine tRNA synthase but cannot pass its outer membrane and is thus poorly active.^30^ Compounds that permit the passage of mupirocin over the outer membrane result in synergy (fractional inhibitory index factor (FICI) ≤ 0.5). When the outer membrane is permeabilized by a compound that directly interacts with and disturbs lipopolysaccharides (LPS), strong synergy is observed (polymyxin B nonapeptide, FICI = 0.0234). When instead the synthesis of LPS is disturbed, moderate synergy is observed (see ACHN-975,^31^ FICI = 0.2969). None of the tested compounds displayed synergy with mupirocin (Table S12). However, it should be noted that compound **7b** came very close to the cutoff value of 0.5 (FICI = 0.5313). Interestingly, ciprofloxacin displayed synergy with mupirocin similar to that of ACHN-975 (FICI = 0.2813), which was not the case for norfloxacin (FICI = 0.8125). This could hint at a possible secondary effect of ciprofloxacin on the integrity of the outer membrane, possibly through a mechanism similar to that of ACHN-975 rather than polymyxin B.

Taken together, all tested compounds showed a typical gyrase inhibition phenotype in *E. coli*, which is consistent with both molecular docking and enzymatic gyrase and topoisomerase IV inhibition assays. In this organism, we could not find evidence of additional mechanisms of action. However, we found moderate synergistic activity of ciprofloxacin with mupirocin, which may point to an additional activity of ciprofloxacin that norfloxacin and its tested derivatives do not possess.

### *In Vivo* Mechanism of Action in Gram-Positive Bacteria

#### Gyrase Inhibition

We proceeded testing the compounds with the most promising activity against Gram-positive species (**2**, **4a**,**c**,**e**,**f**,**k**,**l**, **I6**, **6a**, **8b**, **3c**, **20a**, **21**, **18a**, **12b**, and **13b**) in a corresponding BCP assay using our Gram-positive model *B. subtilis* DSM402 (Figure S66, Table S13). We chose this model for its rod shape because the typical gyrase inhibition phenotype is very clear in rod-shaped bacteria but cannot be distinguished in cocci such as *S. aureus*. Instead of polymyxin B, which is not active against Gram-positive bacteria, the lipopeptide daptomycin was used as control for membrane damage.^22^ All tested compounds elicited a typical gyrase inhibition phenotype, confirming that they retained the mechanism of their parent compound norfloxacin. Only one tested compound, **13b**, did not show a clear nucleoid condensation in all cells, suggesting that it may be a weaker gyrase/topoisomerase IV inhibitor than the other tested compounds.

#### Membrane Activity

While none of the compounds induced cell lysis, cells treated with derivatives **4c**, **4f**, **16**, **3c**, **20a**, **18a**, **18b**, and **12b** showed patchy FM4-64 stains, indicating some degree of membrane damage in *B. subtilis*. Interestingly, this was also the case for norfloxacin and ciprofloxacin. To assess whether this membrane damage was caused by the formation of ion-conducting pores, we performed a membrane depolarization assay using the fluorescence dye DiSC(3)5.^32^ None of the tested compounds caused dissipation of the membrane potential (Figure S67–68). The same results were obtained for norfloxacin and ciprofloxacin (Figures S66–S68, Table S13), suggesting that membrane effects may be a common, yet so far undiscovered, part of the mechanism of fluoroquinolones against Gram-positive bacteria.

#### Effects on Cell Wall Synthesis

Spots in the FM4-64 membrane stain can not only be caused by membrane damage itself, but also by compounds that interfere with cell wall synthesis e.g., by accumulation or clustering of lipid-linked cell wall precursors.^22,33^ Since the membrane spots that we observed could not be explained by depolarization, we examined cell wall synthesis in more detail.

In a first screen, we examined peptidoglycan integrity with an established acetic acid/methanol fixation protocol using *B. subtilis* DSM402.^34,35^ If the cell wall is compromised, the protoplast can protrude through cell wall breaches, which is promoted by the fixation and visible as “bubbles” on the cell surface. As shown in Figure S69, this assay responds to different classes of cell wall synthesis inhibitors, including those with membrane-bound (vancomycin, tunicamycin) and intracellular targets (fosfomycin). None of the tested compounds caused a clear increase of cell wall-compromised cells, yet compounds **12b** and **18b** showed a slight increase compared to the untreated control, and compounds **2**, **6b**, **8b**, **13b**, **20a**, and **21** came close to the cutoff value.

Acetic acid/methanol fixation is a fast and simple first screen for effects on the peptidoglycan cell wall. However, it only tests positive if the incorporation of cell wall precursors is inhibited when at the same time cell wall autolysin activity is maintained. Further, it is sensitive to the compound concentration and does not react to all types of cell wall synthesis inhibition.^34^ For these reasons, we employed a second assay for inhibition of cell wall synthesis, which is based on MreB mobility. For this, we chose compounds **12b** and **18b**, which tested slightly positive in the peptidoglycan integrity assay, as well as two compounds that just reached the threshold level, namely, **20a** and **21**.

**Figure 6:**
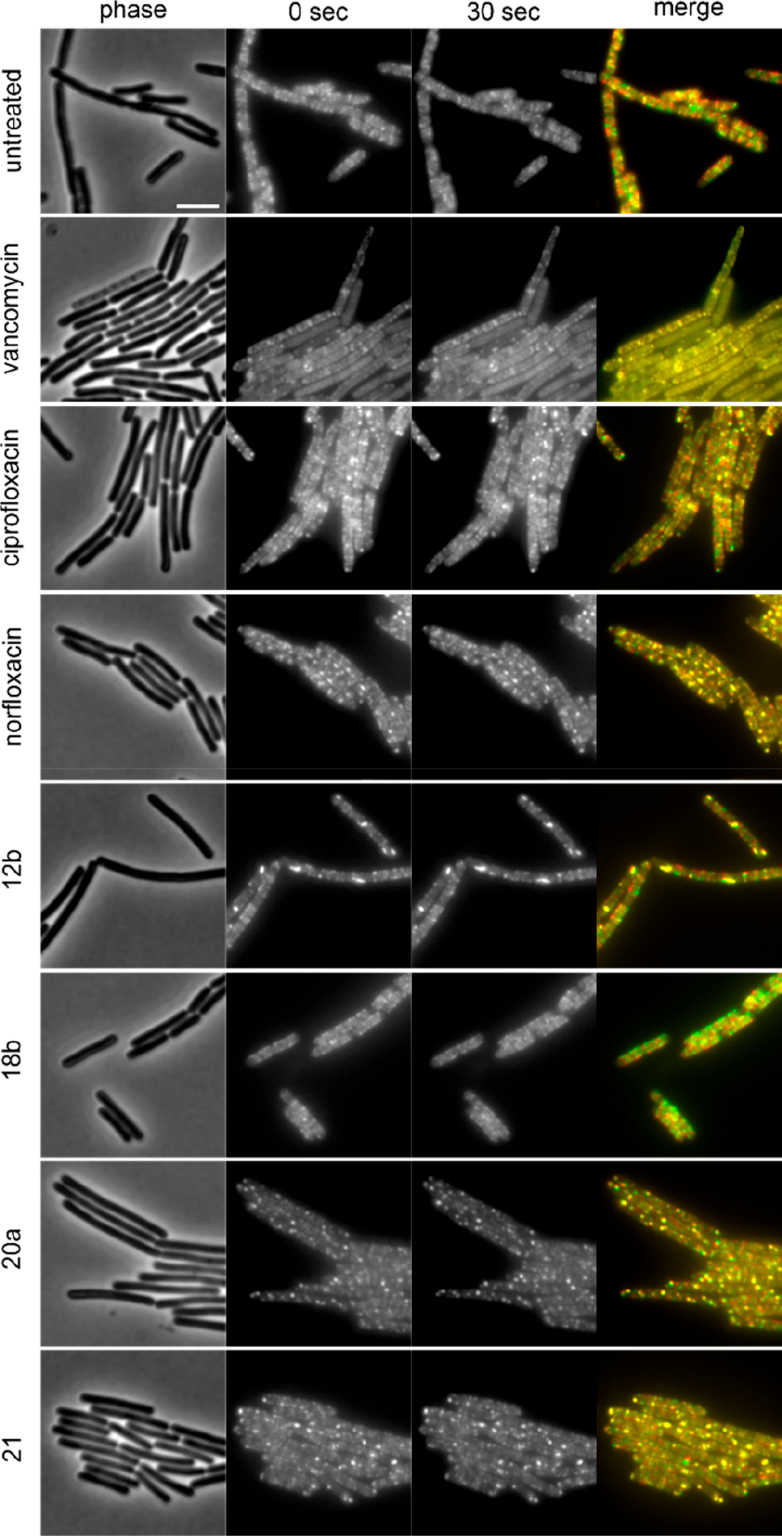
MreB motility in *B. subtilis* MW10 after treatment with norfloxacin derivatives. Expression of msfGFP-MreB was induced with 0.3% xylose. Pictures were taken after 1 h of antibiotic exposure. Two images of the same field of view were recorded 30 s apart and overlaid in ImageJ to visualize MreB mobility. A perfect overlap (yellow) indicates stalled MreB movement, while distinct red and green foci are indicative of MreB mobility. Scale bar 2 μm.

MreB is an actin homologue that forms filamentous structures along the lateral axis of the cell. It moves along the length of the cells in a spiraling pattern thereby driving lateral peptidoglycan synthesis.^36,37^ MreB mobility is sensitive to inhibition of peptidoglycan synthesis and so far, every cell wall synthesis inhibitor we tested abolished MreB movement (see Figure S70 for examples). Figure 6 shows the MreB mobility of compounds **12b**, **18b**, **20a**, and **21**.

While compound **18b** had no effect on either MreB mobility or localization, compound **12b** did affect its localization, causing a few large clusters to appear at the membrane. Yet, the remaining, nonclustered MreB was not restricted in mobility. Compounds **20a** and **21** elicited a similar yet distinct phenotype, showing much smaller clusters and more clusters per cell. Strikingly, the same effect was observed for norfloxacin but not ciprofloxacin, which did not affect either MreB mobility or localization. This observation suggests that these effects on MreB localization are not an inherent property of fluoroquinolones in general but are common to norfloxacin and at least some of its derivatives. It also implies that their effects on cell wall synthesis are not a consequence of gyrase/topoisomerase IV inhibition but a distinct secondary mechanism of action.

In all samples, except for the positive control vancomycin, MreB that was not clustered retained at least some mobility, suggesting that the compounds may not directly interfere with the cell wall synthesis machinery but rather affect this pathway in an indirect manner. MreB localization can also be affected by membrane depolarization, yet this leads to detachment of the protein from the cell membrane,^38^ while mobility of the remaining membrane-bound protein is typically not affected. This phenotype was not observed here, which is in line with our DiSC(3)5 measurements (Figure S67).

The different phenotypes that the selected compounds elicited on MreB suggest that they impair cell wall synthesis by distinct mechanisms. Interestingly, none of the established cell wall synthesis inhibitors that we tested showed a phenotype similar to that of compounds **20a** and **21** (Figures 6 and S70). We did also not observe this specific phenotype in any previous mode of action study that included MreB,^22,38−42^ suggesting that these compounds may have a so far unknown mechanism of action. Therefore, we decided to test other proteins involved in the cell wall synthesis pathway.

**Figure 7:**
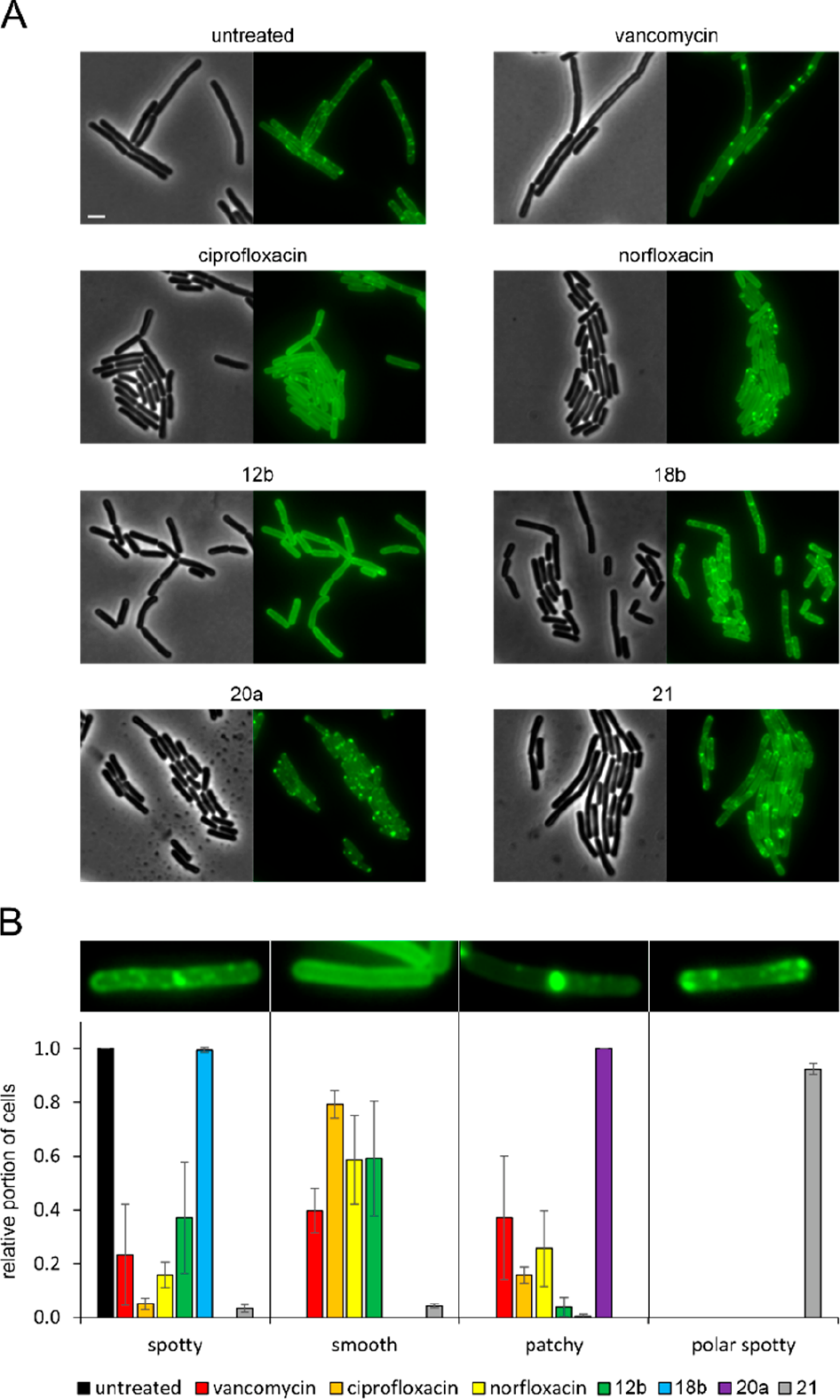
Effects on the localization of the lipid II synthase MurG. (A) Fluorescence and phase contrast microscopy of *B. subtilis* TNVS175. Expression of MurG-msfGFP was induced with 0.05% xylose. Cells were treated with 1x MIC of the respective compounds for 30 min (vancomycin) or 1 h (all other compounds) prior to microscopy. Scale bars are 2 μm. (B) Quantification of microscopy images. Error bars represent the standard deviation of triplicate experiments. A minimum of 50 cells was counted per sample. Total numbers of counted cells: untreated: 300, vancomycin: 221, ciprofloxacin: 204, norfloxacin: 224, **12b**: 63, **18b**: 300, **20a**: 430, **21**: 415.

To this end, we first examined the localization of the essential lipid II synthase MurG. In fast-growing cells, MurG localizes in small spots at the cell membrane, while in slow-growing cells, its localization appears smoother. If peptidoglycan synthesis is inhibited, it forms large clusters in the membrane (see vancomycin control in Figure 7). If the compound additionally disturbs membrane microdomains, which play a role in coordinating cell wall synthesis,^43^ the peripheral membrane protein MurG may also detach from the cell membrane.

In line with the MreB data, we did not observe any effect of compound **18b**, corroborating the notion that this derivative does not interfere with cell wall synthesis. After treatment with **12b**, MurG appeared entirely smooth, likely due to a strongly reduced growth speed. Interestingly, compounds **20a** and **21**, which showed similar effects on MreB, elicited fundamentally different phenotypes on MurG. **20a** caused distinct MurG clusters all over the cells, which is in line with distinct membrane spots observed in the BCP and points to the formation of membrane domains. Compound **21** showed a novel phenotype that has not been observed for MurG before. While the small foci that are characteristic of normal MurG localization did not dissipate completely, they disappeared from the long axis of the cells and instead were only visible in the proximity of the cell poles. This is a curious observation, since in *B. subtilis* cell wall synthesis is active at the lateral cell axis and the cell division septum, but not at the cell poles.^43^

Norfloxacin and ciprofloxacin showed a mostly smooth phenotype, with up to 30% larger MurG clusters. Both compounds also showed membrane spots in the BCP assay, suggesting that they may cause the formation of large membrane domains that could attract MurG.

**Figure 8:**
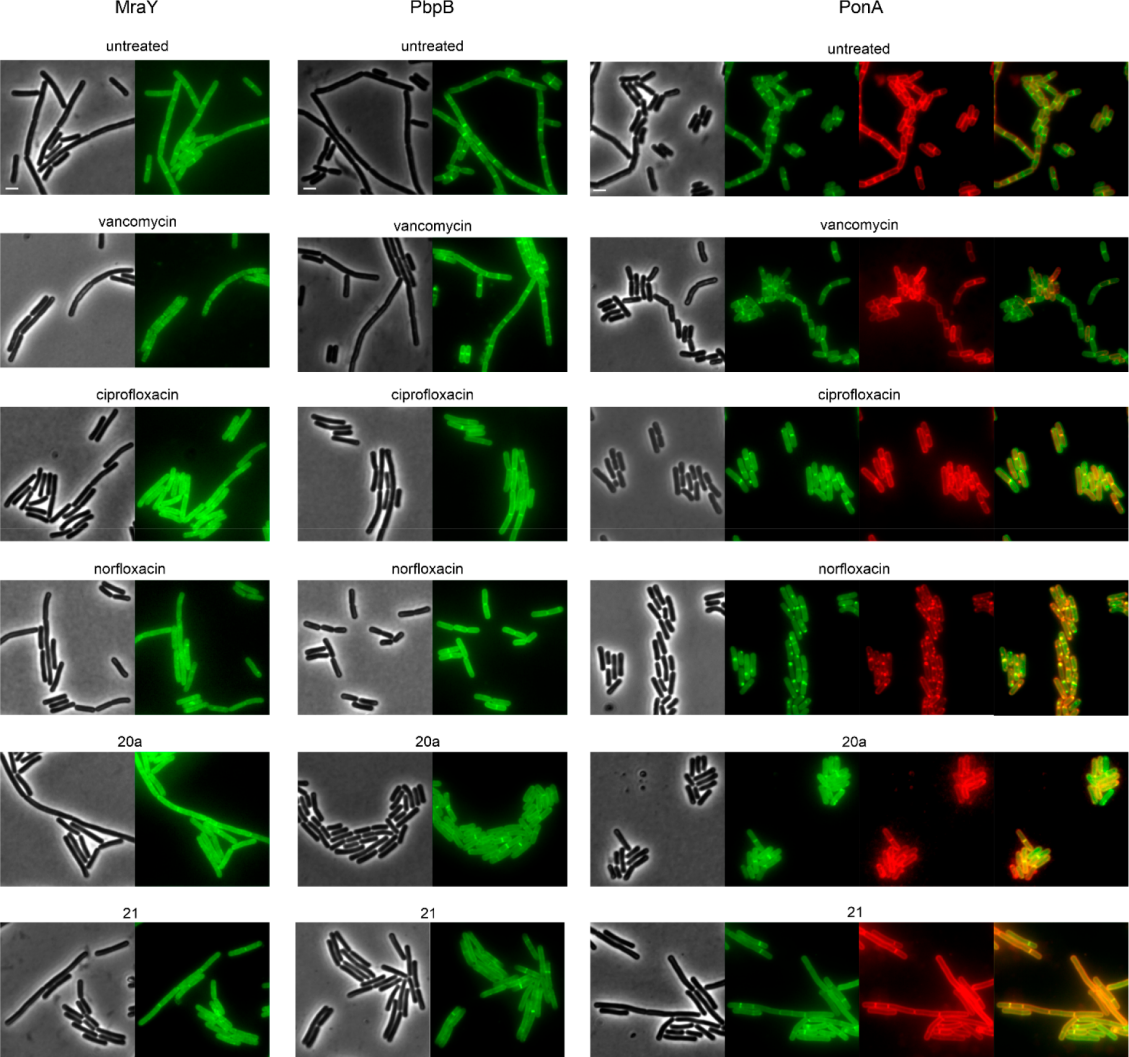
Influence on localization of cell wall synthesis proteins MraY, PbpB, and PonA. *B. subtilis* TNVS284 (MraY-msfGFP), EKB46 (msfGFP-PbpB), and TNVS45 (mGFP-PonA) were grown until early log phase in Muller Hinton broth supplemented with 0.1% xylose to induce expression of the GFP fusion proteins. Cells were treated with 1× MIC of the respective compounds for 30 min (vancomycin) or 1 h (all other compounds) prior to microscopy. TNVS45, which showed a spotty localization in the norfloxacin samples was additionally stained with FM4-64 to visualize colocalization with membrane patches. Scale bars 2 μm.

Compounds **20a** and **21** displayed distinct and unique phenotypes in the MurG assay, corroborating the notion that they affect cell wall synthesis and act in a novel manner. To further asses their effects on the cell wall synthesis machinery, we investigated the localization of the cell wall synthesis proteins MraY, PbpB, and PonA, all of which are integral membrane proteins. The lipid I synthase MraY catalyzes the peptidoglycan biosynthesis step preceding MurG,^44^ while PbpB and PonA are penicillin-binding proteins that catalyze the incorporation of the final precursor into the peptidoglycan layer.^45^ The localization of these proteins is usually insensitive to both membrane disturbances and inhibition of cell wall synthesis.^22^ Thus, they do not delocalize upon treatment with vancomycin (Figure 8).

Given the unusual phenotypes observed for MreB and MurG, we were curious to know if the effects of compounds **20a** and **21** would extend to these less sensitive proteins. Yet, neither compound affected the localization of MraY, PbpB, or PonA. Curiously, norfloxacin had a very notable effect on PonA, showing accumulation in distinct foci at the cell membrane, but no effect on the other two proteins. To assess whether these foci are related to the formation of membrane domains, as observed in the BCP (Figure S66), we costained cells expressing *ponA-gfp* with the membrane dye FM4-64 (Figure 8). Indeed, we observed a clear overlap between GFP foci and membrane patches, indicative of membrane domains.

Table 2 shows a summary of all cell wall assays performed in *B. subtilis*. In conclusion, compounds **20a** and **21** showed distinct effects on the cell wall synthesis machinery that have not been observed in previous studies, suggesting that they may possess a novel secondary mechanism of action that affects cell wall synthesis. Whether this is a direct interaction with a component of this pathway or an indirect effect due to the inhibition of a different target remains to be elucidated. It is curious that these compounds did not test positive in the peptidoglycan integrity assay, yet this may be consistent with a novel mechanism since the fixation assay does not react to all types of cell wall synthesis inhibition. Compound **12b** also showed effects on the cell wall synthesis machinery yet delivered surprising results. While it did cause membrane foci and clustered MreB, it diminished the spotty pattern of MurG but did not cluster this protein. This may point to yet another mechanism of cell wall synthesis inhibition or indirect effects, possibly to nondisruptive membrane impairment. Compound **18a** did not show any effects on the cell wall synthesis machinery. Interestingly, norfloxacin did affect MreB, MurG, and PonA localization, while ciprofloxacin only affected MurG. This suggests that fluoroquinolones may have secondary effects on cell wall synthesis. It will be interesting to investigate whether these effects can be linked to the inhibition of DNA gyrase and topoisomerase IV or are representative of an independent secondary mechanism of these compounds. The observation that norfloxacin and ciprofloxacin had markedly different effects rather suggests the latter.

**Table 2:** Summary of Cell Wall Synthesis Experiments in *B. subtilis* DSM402^a^

				protein localization				
compound	μM	PG integrity	MreB mobility	MurG	MraY	PbpB	PonA	CWB inhibited?
untreated		intact	mobile	spotty	rough	smooth	septal	no
Cip	3.01	intact	mobile	smooth/spotty	rough	smooth	septal	no
Nor	18.11	intact	mobile with static foci	smooth/spotty	rough	smooth	septal/patchy	no
Van	0.68	compromised	static with foci	patchy/dispersed	rough	smooth	septal	yes
D-Cyc	293.85	compromised	static with foci	patchy	n.d.	n.d.	n.d.	yes
Fos	72.43	compromised	static with foci	patchy/dispersed	n.d.	n.d.	n.d.	yes
Tun	19.58	compromised	static	patchy	n.d.	n.d.	n.d.	yes
**2**	1.89	intact	n.d.	n.d.	n.d.	n.d.	n.d.	no
**3c**	1.09	intact	n.d.	n.d.	n.d.	n.d.	n.d.	no
**4a**	8.84	intact	n.d.	n.d.	n.d.	n.d.	n.d.	no
**4e**	2.07	intact	n.d.	n.d.	n.d.	n.d.	n.d.	no
**4f**	6.21	intact	n.d.	n.d.	n.d.	n.d.	n.d.	no
**4k**	6.14	intact	n.d.	n.d.	n.d.	n.d.	n.d.	no
**4l**	70.56	intact	n.d.	n.d.	n.d.	n.d.	n.d.	no
**6a**	3.94	intact	n.d.	n.d.	n.d.	n.d.	n.d.	no
**8b**	33.79	intact	n.d.	n.d.	n.d.	n.d.	n.d.	no
**12b**	21.03	slightly compromised	mobile with static foci	smooth	n.d.	n.d.	n.d.	yes^b^
**13b**	5.83	intact	n.d.	n.d.	n.d.	n.d.	n.d.	no
**16**	2.01	intact	n.d.	n.d.	n.d.	n.d.	n.d.	no
**18a**	49.71	intact	n.d.	n.d.	n.d.	n.d.	n.d.	no
**18b**	3.10	slightly compromised	mobile with static foci	spotty	n.d.	n.d.	n.d.	yes^b^
**20a**	16.11	intact	mobile with static foci	patchy	rough	smooth	septal	yes^b^
**21**	2.13	Intact	mobile with static foci	polar spotty	rough	smooth	septal	yes^b^

a CWB: cell wall biosynthesis, Cip: ciprofloxacin, Nor: norfloxacin, Van: vancomycin, D-cyc: d-cycloserine, Fos: fosfomycin, Tun: tunicamycin.

b Possibly indirect or partial inhibition or novel mechanism.

## Conclusion

A series of *N*4-substituted piperazinyl derivatives of norfloxacin were designed and synthesized aiming at improving antibacterial activity. Several derivatives displayed activities that were comparable to or better than norfloxacin. Selecting the most promising candidates, gyrase and topoisomerase IV inhibition was confirmed for all tested compounds *in silico*, *in vitro*, and *in vivo*. Interestingly, compounds **12b**, **20a**, and **21** displayed unique effects on the bacterial cell wall synthesis machinery, suggesting that they may have a secondary target in this pathway or a target in another process that in turn, influences cell wall synthesis. Since their effects were distinct from each other as well as from norfloxacin and ciprofloxacin, we conclude that this activity is not a simple consequence of gyrase/topoisomerase IV inhibition, but likely an independent secondary mode of action. This could prove advantageous for resistance development, since multitarget antibiotics display slower resistance development rates that single-target antibiotics.^48^

Interestingly, we also found evidence for a secondary mechanism of norfloxacin and, to a lesser extent ciprofloxacin. While these effects were clearly different from those elicited by **12b**, **20a**, and **21** in most assays, this observation implies that we do not understand the mechanism of action of classical fluoroquinolones as well as generally assumed. Further research will be needed to elucidate a possible causality between gyrase/topoisomerase IV inhibition and impairment of the cell wall synthesis machinery or an underlying independent mechanism of action.

## Supporting Information Available

Chemical synthesis, chemical characterization of synthesized compounds, QSAR, prediction of drug likeness, prediction of ADME/Tox, additional molecular docking data, additional mode of action data, HPLC analysis of lead compounds, methods, and supplementary references (PDF)
Dock result PDB of **4b** on 5CDQ (PDB)
Dock result PDB of **18a** on 2XKK (PDB)

## Supplementary Material

bg3c00038_si_001.pdf

bg3c00038_si_002.pdb

bg3c00038_si_003.pdb
