# Supplementary material for: N4-Substituted Piperazinyl Norfloxacin Derivatives with Broad-Spectrum Activity and Multiple Mechanisms on Gyrase, Topoisomerase IV, and Bacterial Cell Wall Synthesis
Source: ACS Bio Med Chem Au. 2023 Aug 30;3(6):494–506. doi: 10.1021/acsbiomedchemau.3c00038 (PMC10739246; doi:10.1021/acsbiomedchemau.3c00038)
Supplement: Supplementary file 1 — bg3c00038_si_001.pdf [file bg3c00038_si_001.pdf]

N4-substituted piperazinyl norfloxacin derivatives with broad-spectrum activity and multiple mechanisms on gyrase, topoisomerase IV, and bacterial cell wall synthesis

Ahmed M. Kamal El-sagheir[1#], Ireny Abdelmesseh Nekhala[2#], Mohammed K. Abd El-Gaber[1], Ahmed S. Aboraia[1], Jonatan Persson[2,3], Ann-Britt Schäfer[2,3], Michaela Wenzel[2,3*], Farghaly A. Omar[1*].

[1]Medicinal Chemistry Department, Faculty of Pharmacy, Assiut University, Assiut, Egypt, 71526.

[2]Division of Chemical Biology, Department of Life Sciences, Chalmers University of Technology, 412 96 Gothenburg, Sweden.

[3]Center for Antibiotic Resistance Research in Gothenburg (CARe), 405 30 Gothenburg, Sweden.

[#]These authors contributed equally to this work

*Corresponding authors: Michaela Wenzel (wenzelm@chalmers.se) and Farghaly A. Omar (farghalyomar@pharm.aun.edu.eg)

Table of contents

1. **Chemical synthesis**
   Text S1: Compound synthesis
   Scheme S1-5: Synthesis of compound series 1-5
2. **Chemical characterization of synthesized compounds**
   Fig. S1-46: ^{1}HNMR (a) and ^{13}CNMR spectra (b) of the synthesized compounds
   Fig. S47: Elemental analysis certificates
3. **QSAR**
   Text S2: Quantitative structure activity relationship (QSAR)
   Text S3: QSAR model
   Tab. S1: Values of molecular descriptors for training and test set compounds
   Fig. S48: Correlation matrix of the calculated descriptors
   Tab. S2: The experimental and predicted activities (LogMIC), residuals and Z-Scores for the tested compounds calculated using normal validation $PRED, $RES and $Z-SCORE and the corresponding cross-validation properties, $XPRED, $XRES and $XZ-SCORE
   Tab. S5: Abbreviations of validation parameters
   Tab. S6: Experimental and predicted LogMIC of test set compounds
   Fig. S49: Correlation between Exp Log MIC and Pred Log MIC of validation test set
4. **Prediction of drug likeness**
   Text S4: Prediction of physicochemical properties
   Tab. S7: Physicochemical properties of norfloxacin and designed compounds
5. **Prediction of ADME/Tox**
   Text S5: *In silico* prediction of ADME/Tox
   Text S6: ADME/Tox prediction using pKCSM lab
   Tab. S8: Predicted ADME/TOX properties by pKCSM
   Text S7: ADME prediction by SwissADME
   Tab. S9: ADME properties predicted by SwissADME
6. **Molecular docking**
   Text S8: Molecular docking
   Fig. S50: 2D and 3D interactions of co-crystallized ligand moxifloxacin with DNA gyrase (PDB ID: 5CDQ)
   Fig. S51-55: 2D and 3D interactions of compounds with DNA gyrase (PDB ID: 5CDQ)
   Fig. S56: 2D and 3D interactions of co-crystallized ligand moxifloxacin with DNA topoisomerase IV (PDB ID: 2XKK)
   Fig. S57-61: 2D and 3D interactions of compounds with DNA topoisomerase IV (PDB ID: 2XKK)
   Tab. S10: Interaction energies (Kcal/mol) and MIC (µM) of the tested compounds with DNA-gyrase/ topoisomerase IV
7. **Mode of action studies**

Fig. S62: % inhibition of investigated compounds and norfloxacin on *E. coli* DNA gyrase
Fig. S63: % inhibition of investigated compounds and norfloxacin on *E. coli* DNA topoisomerase IV
Fig. S64: Bacterial cytological profiling of *B. subtilis* DSM402 treated with antimicrobial compounds that affect DNA integrity
Fig. S65: Bacterial cytological profiling of *E. coli* BCB472
Tab. S11: Results summary of BCP in *E. coli*
Tab. S12: Results of checkerboard assays of norfloxacin derivatives combined with mupirocin
Fig. S66: Bacterial cytological profiling of *B. subtilis*. Fluorescence and phase contrast microscopy of *B. subtilis* DSM402
Tab. S13: Results summary of BCP in *B. subtilis* DSM402
Fig. S67: Effects of the membrane potential in *B. subtilis* DSM 402
Fig. S68: Ciprofloxacin derivatives do not interfere with DiSC(3)5 fluorescence
Fig. S69: Effects on peptidoglycan synthesis
Fig. S70: MreB motility in *B. subtilis* MW10 after treatment with different cell wall synthesis inhibitors
Fig. S71-73: Chromatogram of compounds **4e, 4a,** and **21**

8. **HPLC analysis of lead compounds**
9. **Methods**

Text S9: Synthesis of intermediates
Scheme S1: Synthesis of intermediates
Text S10: Chemical synthesis
Tab. S14: Yield and reaction time of target compounds.
Text S11: Antimicrobial activity
Tab S14: Strains used in this study
Text S12: Cytotoxicity studies
Text S13: Molecular modeling
Text S14: Mode of action studies
Text S15: HPLC analysis

10. **References**

Text S16: References

1. Chemical synthesis

Text S1: Compound synthesis

*N*4-substituted piperazinyl norfloxacin derivatives (**Series 1 - 5**) were synthesized as depicted in **Schemes S1-5**. Chemical structures of the prepared compounds were elucidated by elemental analysis and spectral techniques (see **Fig. S1-46** for corresponding ^{1}H NMR and ^{13}C NMR data and **Fig. S47** for elemental analysis). In case of **series 1**, the targeted derivatives **3a-c, 4a-l, and 5a-b** were prepared as outlined in **Scheme 1**. The key intermediate *N*4-chloroacetylpiperzinyl norfloxacin **2** was prepared by treatment of norfloxacin **1** with chloroacetylchloride using triethylamine as acid scavenger in THF [18]. Reaction of compound **2** with different amines in the presence of K_2CO_3 and a catalytic amount of KI afforded the *N*4-substiutedaminoacetyl-piperazin-1-yl norfloxacin derivatives **3a-c, 4a-l, and 5a-b**. A series of representative alkyl and cycloalkylamines (ethyl, propyl and cyclohexyl amines) were selected to afford the corresponding *N4*-alkylanimoacetylpiperazinyl derivatives **3a–c.** The ^{1}H NMR spectra of compounds **3a-c** were characterized by the signals of the aliphatic protons at δ ~ 0.8 – 2 ppm. The corresponding *N*4-substituted phenylamino- (**4a – k**), 4-pyridylamino- (**4l**), and 2-thiazlylamino- (**5a-b**) acetyl analogs showed characteristic bands in IR spectra at 3460-3430 cm^{-1}, 3370-3310 cm^{-1}, 1715-1705 cm^{-1}, 1665-1660 cm^{-1}, and 1640-1615 cm^{-1} attributed to the NH, OH, acidic C=O, amidic C=O, and quinolone C=O groups, respectively. Beside the expected aromatic protons, the ^{1}H NMR spectra revealed a characteristic singlet at δ ~ 3.90 - 4.6 ppm due to the methylene protons of the acyl linker NH-$\underline{CH_2}$-CO and broad singlets at δ ~ 5.24 - 6.8 ppm assigned to $\underline{NH}$-CH_2 proton.

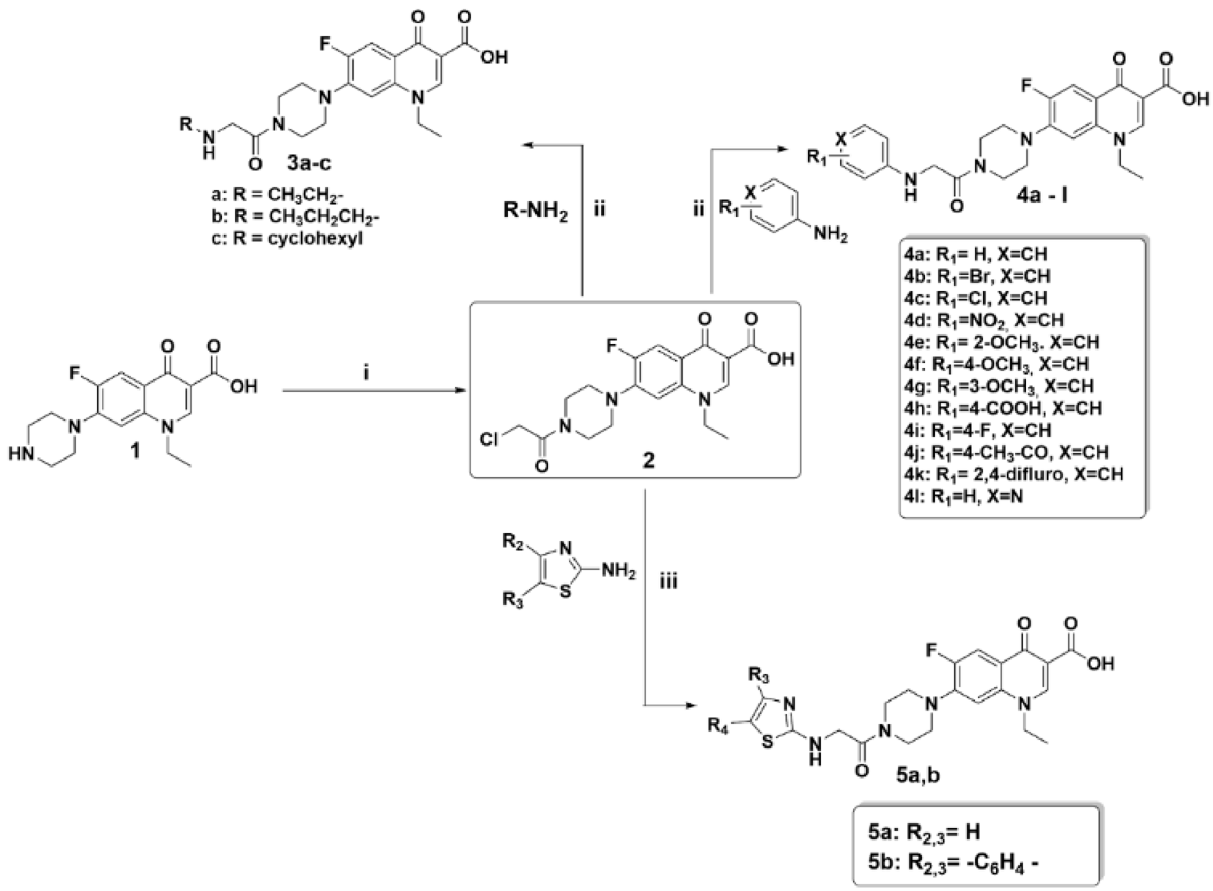

Scheme 1: Synthesis of *N*4-substiutedaminoacetyl piperazinyl norfloxacin derivatives. Reagents and conditions: i) $ClCOCH_2Cl$, Et_3N, THF, Reflux; ii) DMF, K_2CO_3, KI, 80 ºC; iii) Dioxane, K_2CO_3, KI, 80 ºC.

Series 2 represents modifications of the linker separating the added substituents and the norfloxacin skeleton. Thereby, the acetyl linker is directly attached to *N*-atom incorporated into cyclic structures e.g. isatin, piperidine, morpholine, imidazole, as well as a triazole nucleus. The synthetic procedures shown in **Scheme 2** are largely analogous to that in scheme **1**, using the chloroacetyl key intermediate **2** but replacing DMF as solvent with acetonitrile and using triethylamine as acid scavenger instead of K_2CO_3. *N*4-isatin-1-yl-acetylpiperazinyl derivatives **6a-b** demonstrated IR spectra signifying the OH stretching bands of norfloxacin at 3365-3245 cm^{-1} and strong isatin carbamidic C=O stretching at 1755-1745 cm^{-1}. The ^{1}H-NMR spectra revealed singlet signal at δ ~ 4.73 - 4.83 ppm due to the NH-$\underline{CH_2}$-CO protons in addition to the expected signals of the aromatic protons. The ^{1}H-NMR spectra of *N*4-piperidin-1-ylacetyl- and *N*4-

morpholinylacetyl-piperazine derivatives **7a-b** exhibited the expected signals of aliphatic protons at δ ~ 0.8 – 2 ppm. It is noteworthy, that the synthesis of *N*4-imidazolylacetyl- derivative **8a** as well as the *N*4-triazolylacetyle analog **8b** necessitated the use of diisopropylethylamine (DIPEA) as basic catalyst in refluxing acetonitrile, resulting in excellent yields of 72-77%. Examination of the ^{1}H-NMR spectrum of **8b** showed a singlet signal at δ = 5.59 ppm assigned to NH-<u>CH</u>$_2$-CO protons and a singlet at δ = 8.81 ppm attributed to proton of the triazole ring, where compound **8a** showed three characteristic signals that were two doublet signals at δ =7.52 and 7.25 ppm and singlet signal at δ =7.41 ppm attributed to protons of the imidazole ring.

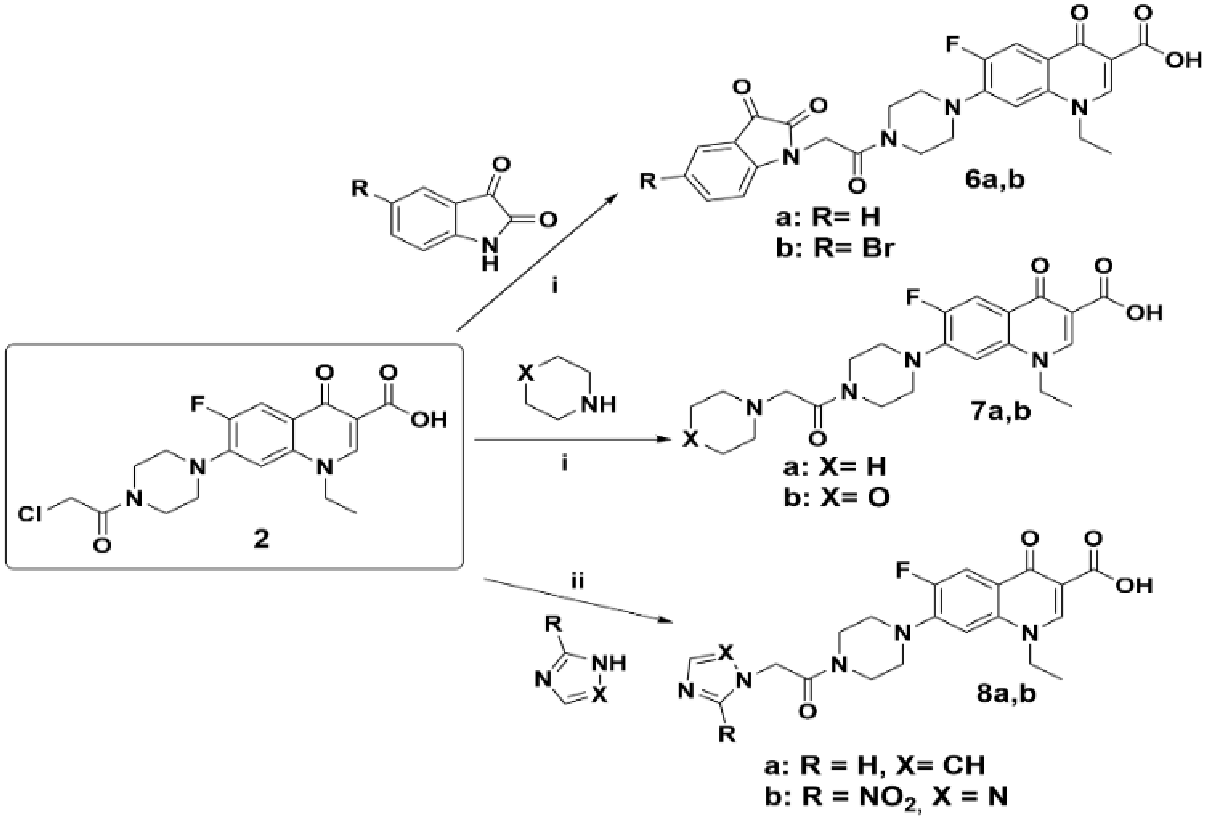

Scheme 2: Synthesis of N4-cyclic aminoacetyl piperazinyl norfloxacin derivatives. Reagents and conditions: i) CH_3CN, Et_3N, KI, 60-80 ºC; ii) CH_3CN, DIPEA, 60-80 ºC.

The linker modification in **series 3** involves a thioether linkage **(-S-)** to the acetyl residue instead of **(-N-)**. The substitution reaction includes treatment of the 2-chloroacetyl intermediate **2** with the respective 2-mercapto-1,3,4-triazole derivatives **9a-b**; 2-mercapto-1,3,4-oxadiazole derivatives

10a-c; or 2-mercaptoquinazolin-4(3*H*)-one **11** as illustrated in **Scheme 3**. The targeted compounds include: The triazolyl thioacetyl derivatives **12a-b**; 1,3,4-oxadiazolyl derivative **13a–c**; and quinazolinon-2yl-thioacetylanalog **14**. The ^{1}H NMR spectrum of compound **14** showed an exchangeable singlet signal at δ 12.64 ppm assigned to the NH proton of the quinazoline ring. The IR spectra showed absorption bands at 3365-3245 cm^{-1}, 1730-1720 cm^{-1}, 1665-1645 cm^{-1}, and 1630-1620 cm^{-1} attributed to the OH, acidic C=O, quinolone C=O and amidic C=O groups, respectively.

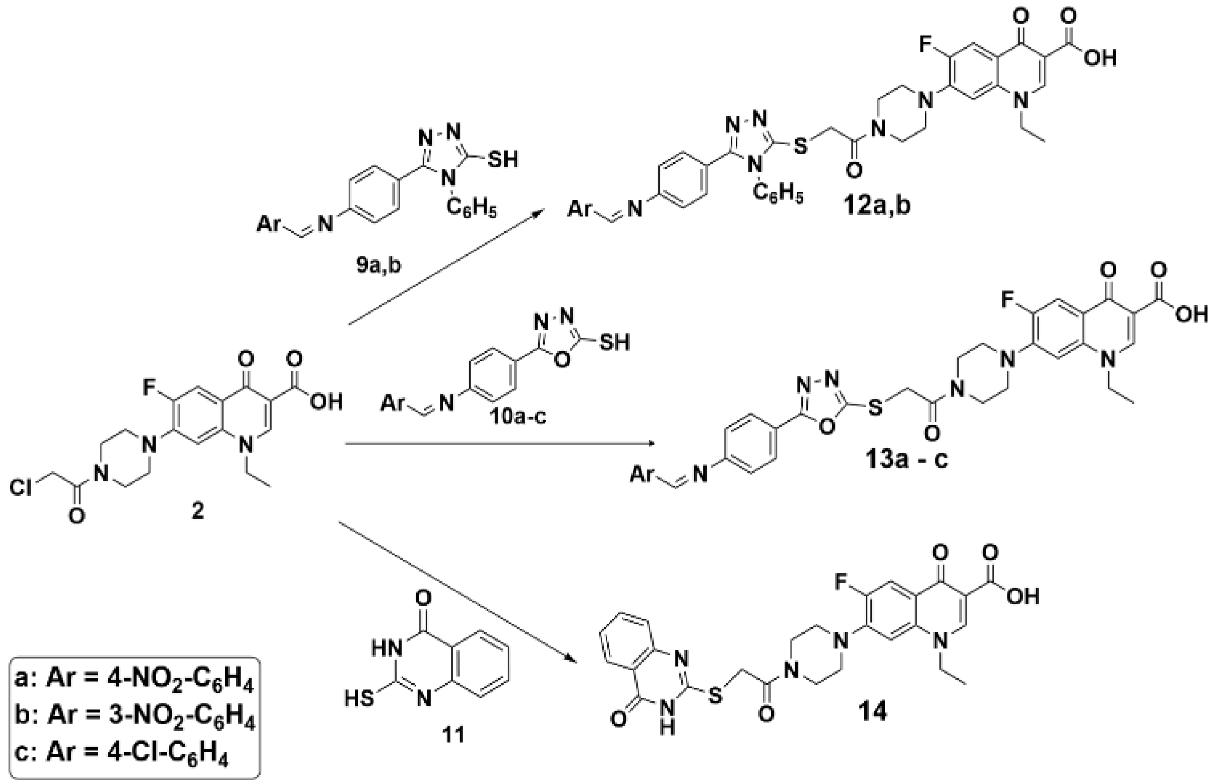

Scheme 3: Synthesis of the targeted N4-substituted thioacetyl piperazine derivatives. Reagents and conditions: acetonitrile, K_2CO_3, KI, reflux / acetonitrile, Et_3N, KI, reflux.

Series 4 involves incorporation of a hydrazide moiety (-CONHNH-) between the acetyl residue and the pending aryl or heteroaryl substituents. **Scheme 4** illustrates the synthetic approaches for preparation of compounds **16** and **18a-c** through reaction of the 2-chloroacetyl intermediate 2 and

isonicotinic acid hydrazide **15** (INH) or the respective 4-arylidene aminophenylcarbohydrazides **17a-c**, respectively. Compound **15** represents a hybrid molecule of norfloxacin and the well-known antimycobacterial agent isoniazid and was designed to implement a new chemical entity with enhanced antimicrobial activity. The IR spectrum showed absorption bands at 3415 cm^{-1} due to the NH stretching. Furthermore, the ^{1}H NMR spectrum of **17** revealed two exchangeable peaks at δ 3.5 and 8.9 ppm assigned to the two NH protons. The ^{1}H NMR spectra displayed the anticipated aromatic protons and a characteristic singlet at δ 4.5 - 5.3 ppm due to the NH-$\underline{CH_2}$-CO protons and a broad singlet at δ 8.20 - 8.7ppm assigned to NH proton.

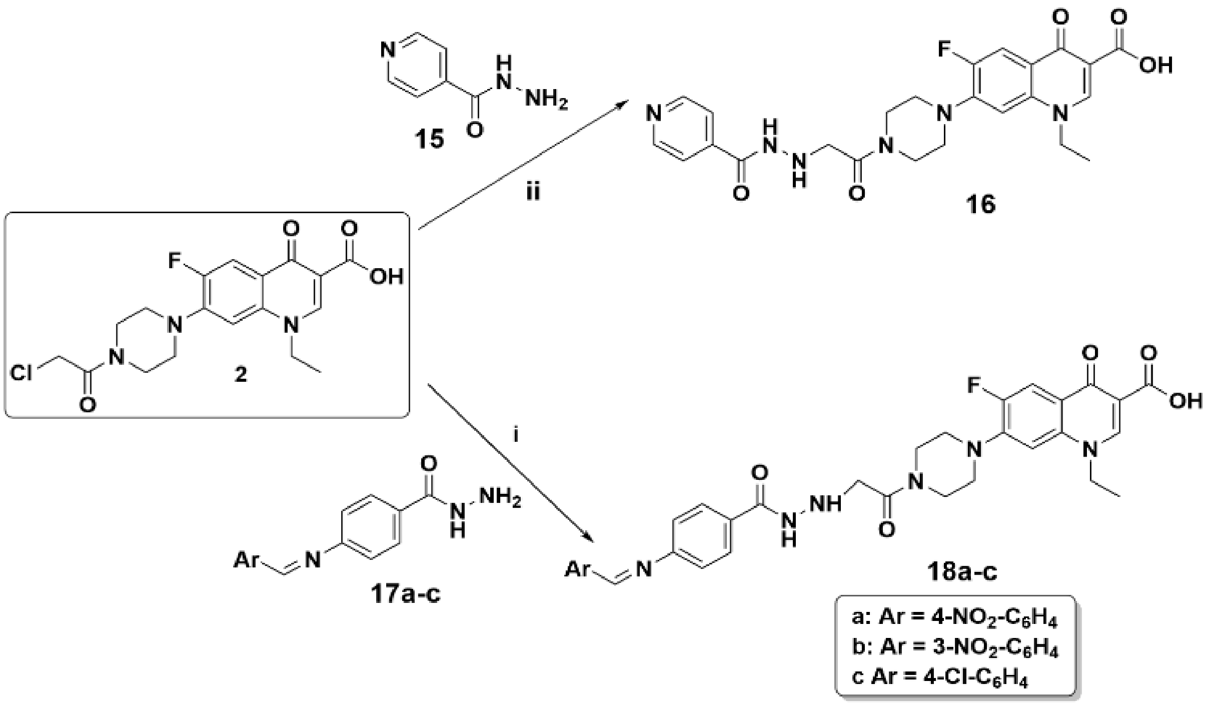

Scheme 4: Synthesis of 2-hydrazidoacetyl linked compounds. Reagents and conditions: (i) acetonitrile, K_2CO_3, KI, reflux. (ii) acetonitrile, Et_3N, KI, reflux.

In **series 5** the acetyl linkage at *N*4-of the piperazinyl moiety has been replaced by methylene spacer (-CH_2-). The synthesis proceeds through application of Mannich conditions as shown in **Scheme 5**, whereby condensation of norfloxacin **1**, formalin, and the respective acidic proton components commenced. The resulting Mannich bases **20a-c** and **21** showed IR absorption bands

at 3450 cm^{-1}, 1750, 1715 cm^{-1}, and 1620 cm^{-1} attributed to the OH, amidic C=O, acidic C=O, and quinolone C=O respectively. 1H-NMR spectra demonstrated the presence of a singlet signal (2H) at $\delta \sim 3.98 - 4.56$ ppm assigned to $-N\underline{CH_2}N-$ protons, in addition to the signals at the aromatic region assigned to isatin and 4-nitroaniline protons.

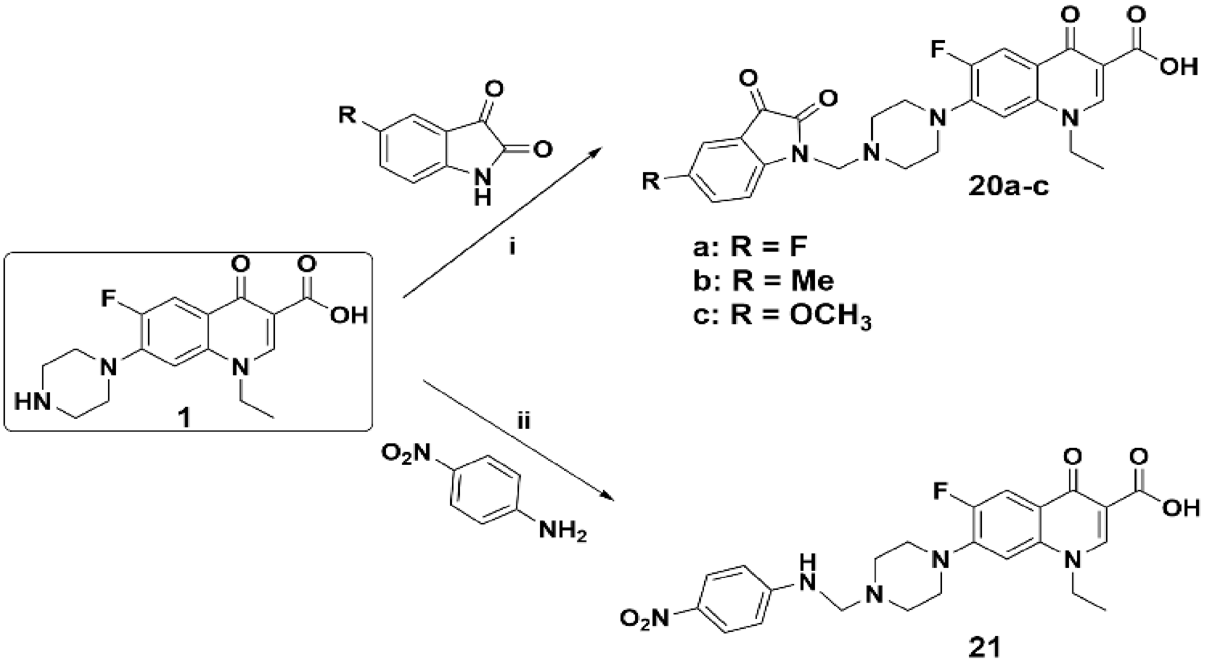

Scheme 5: Synthesis of Mannich bases of norfloxacin. Reagents and conditions: (i) MeOH, HCHO, reflux. (ii) EtOH, HCHO, glacial acetic acid, reflux.

2. Chemical characterization of synthesized compounds

Compound 2

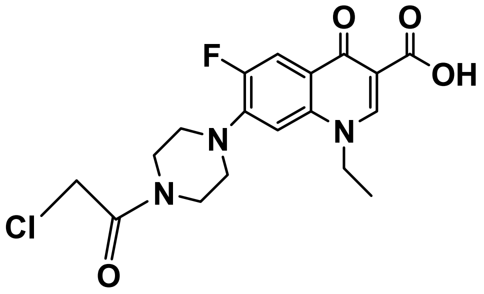

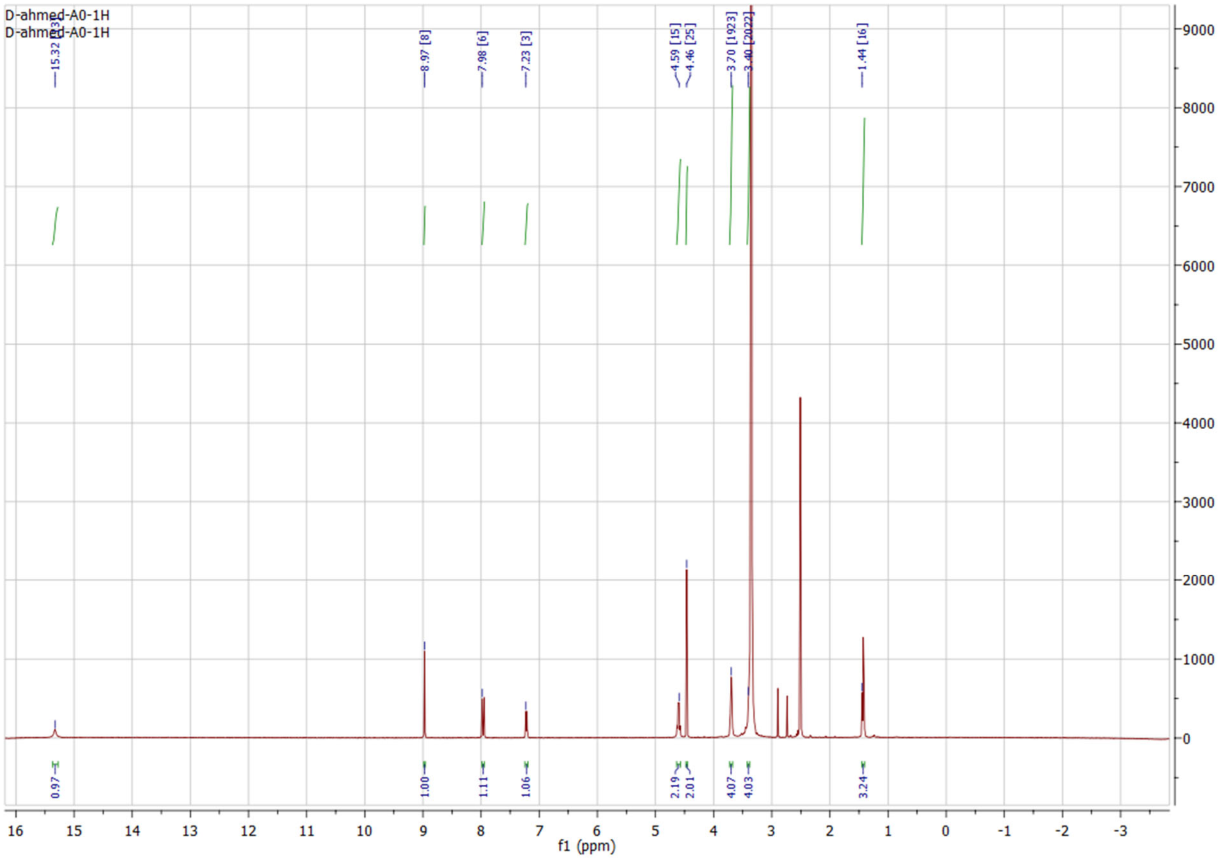

Figure S1: ^{1}HNMR

Compound 4a

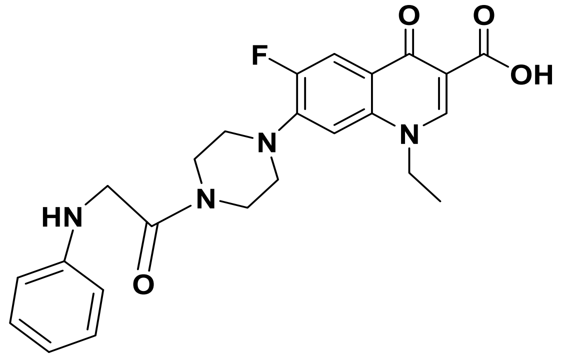

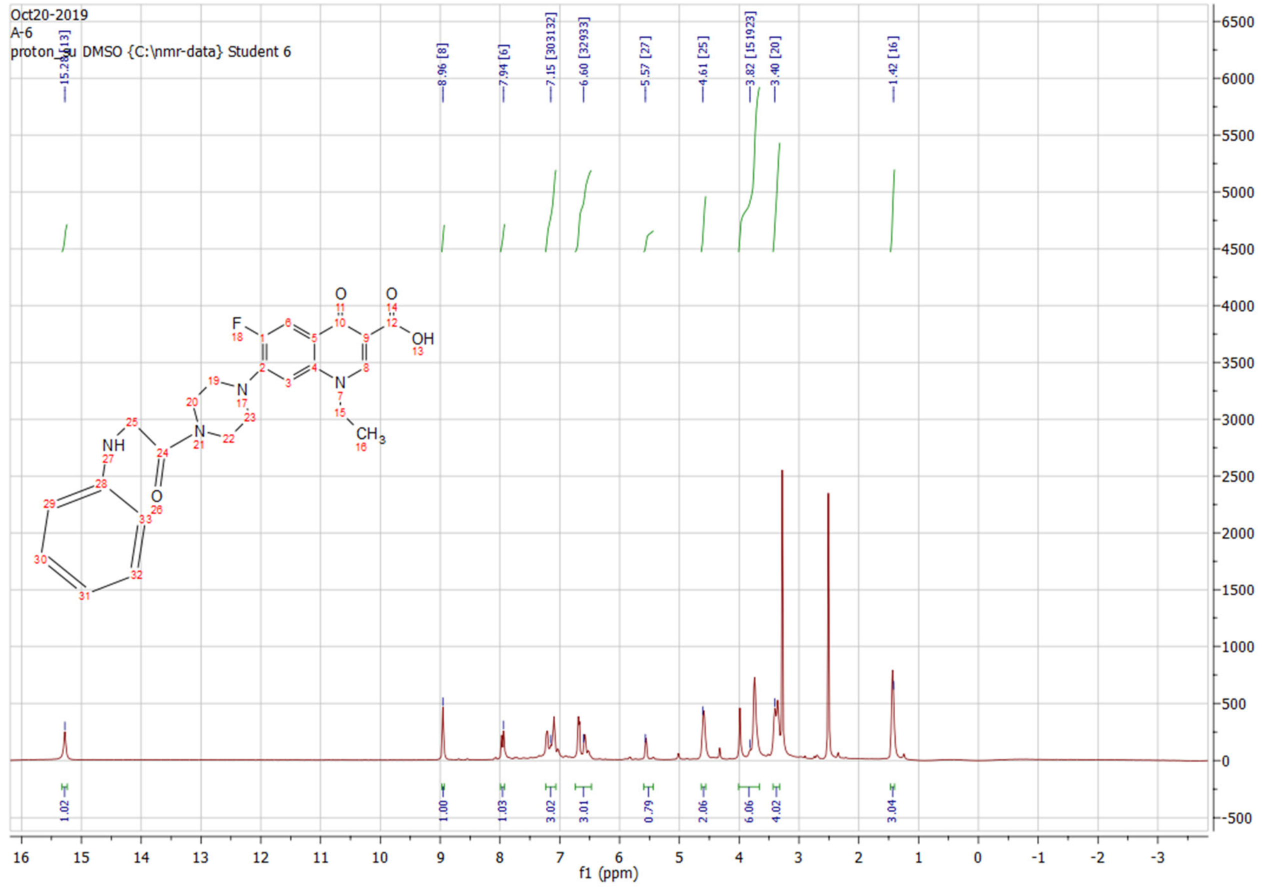

Figure S2a: ^{1}HNMR

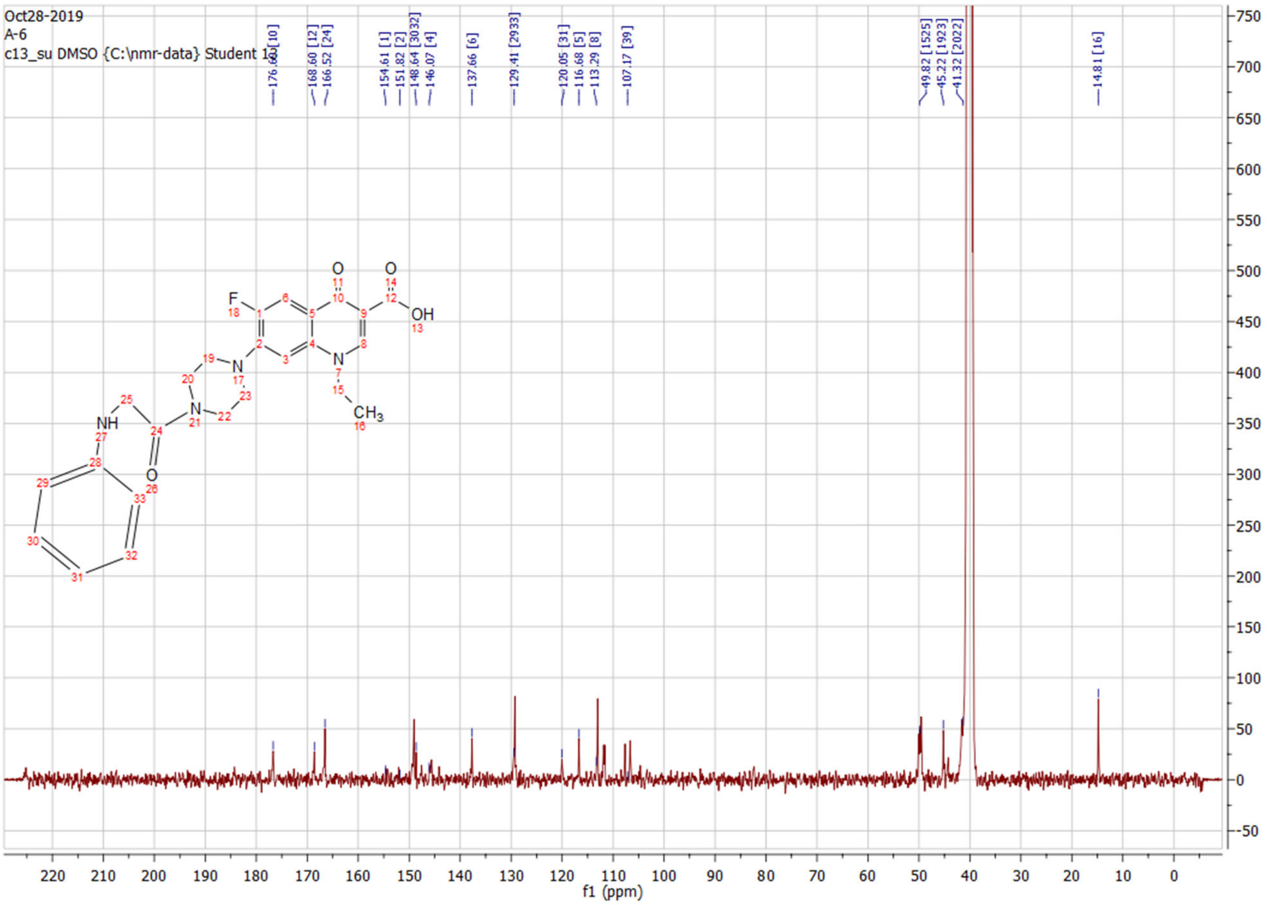

Figure S2b: ^{13}CNMR

Compound 4b

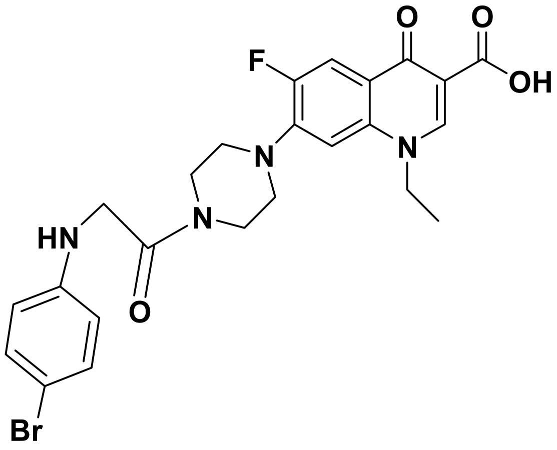

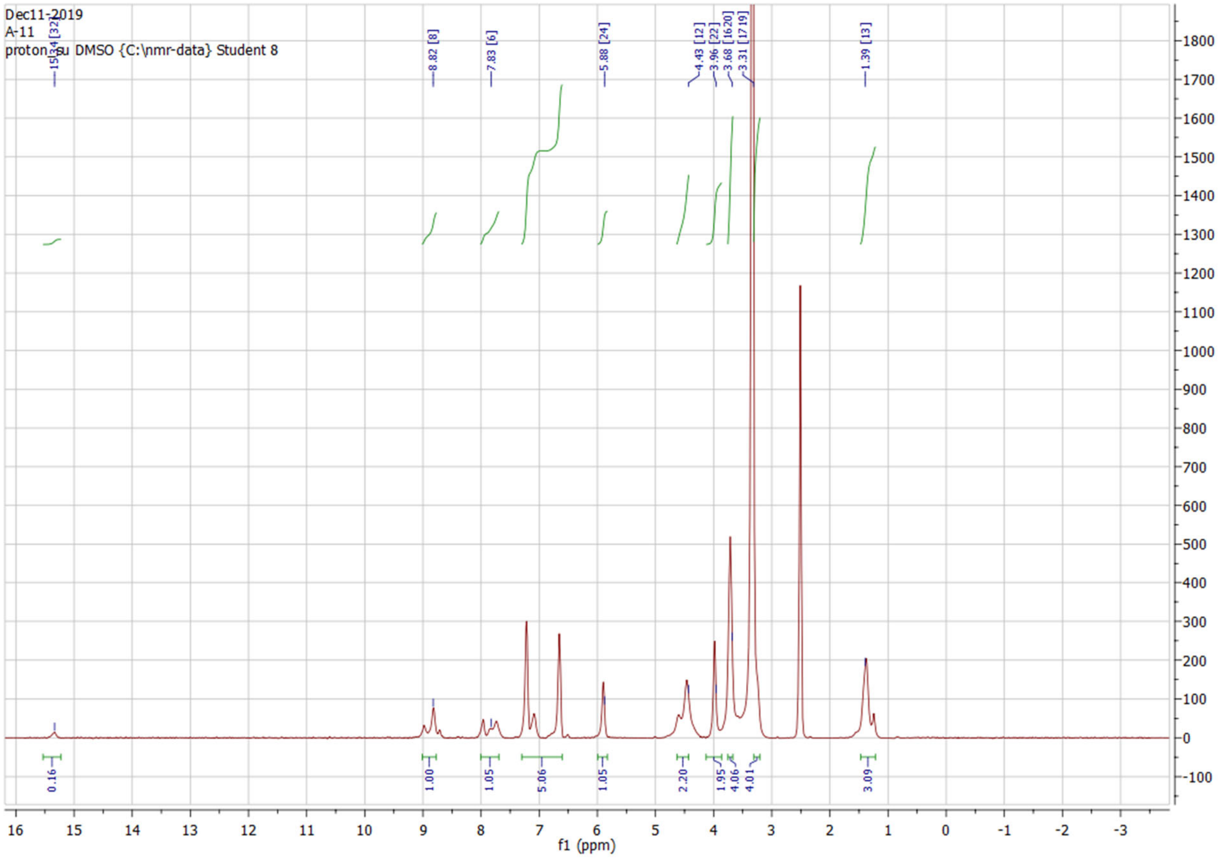

Figure S3a: ^{1}HNMR

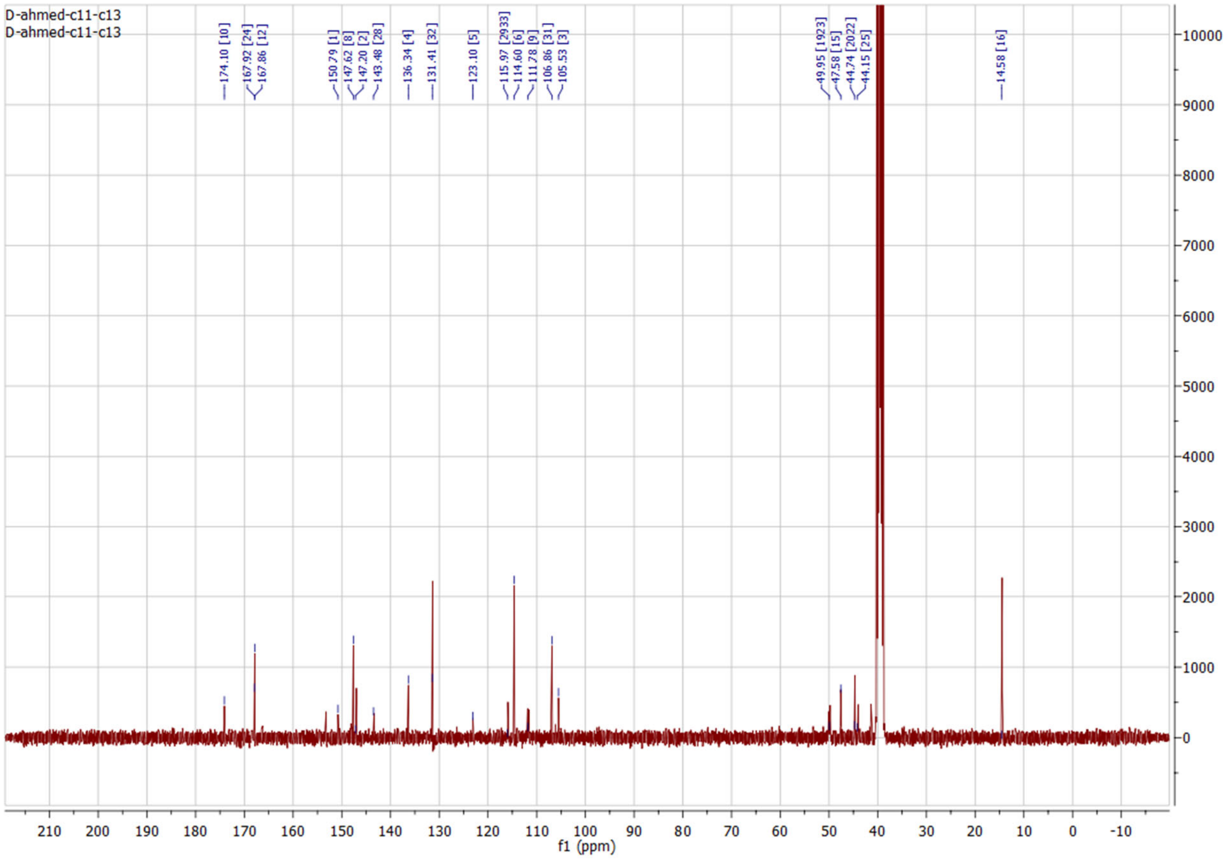

Figure S3b: ^{13}CNMR

Compound 4c

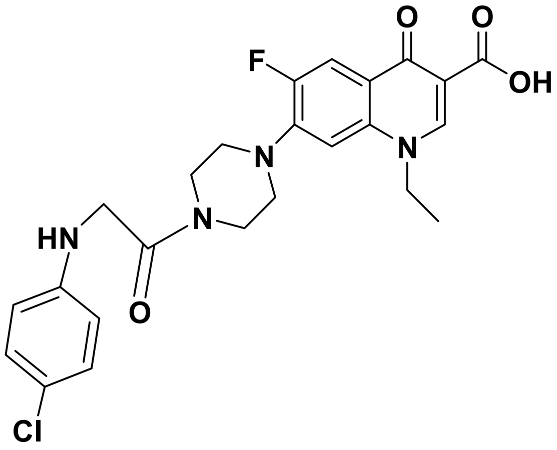

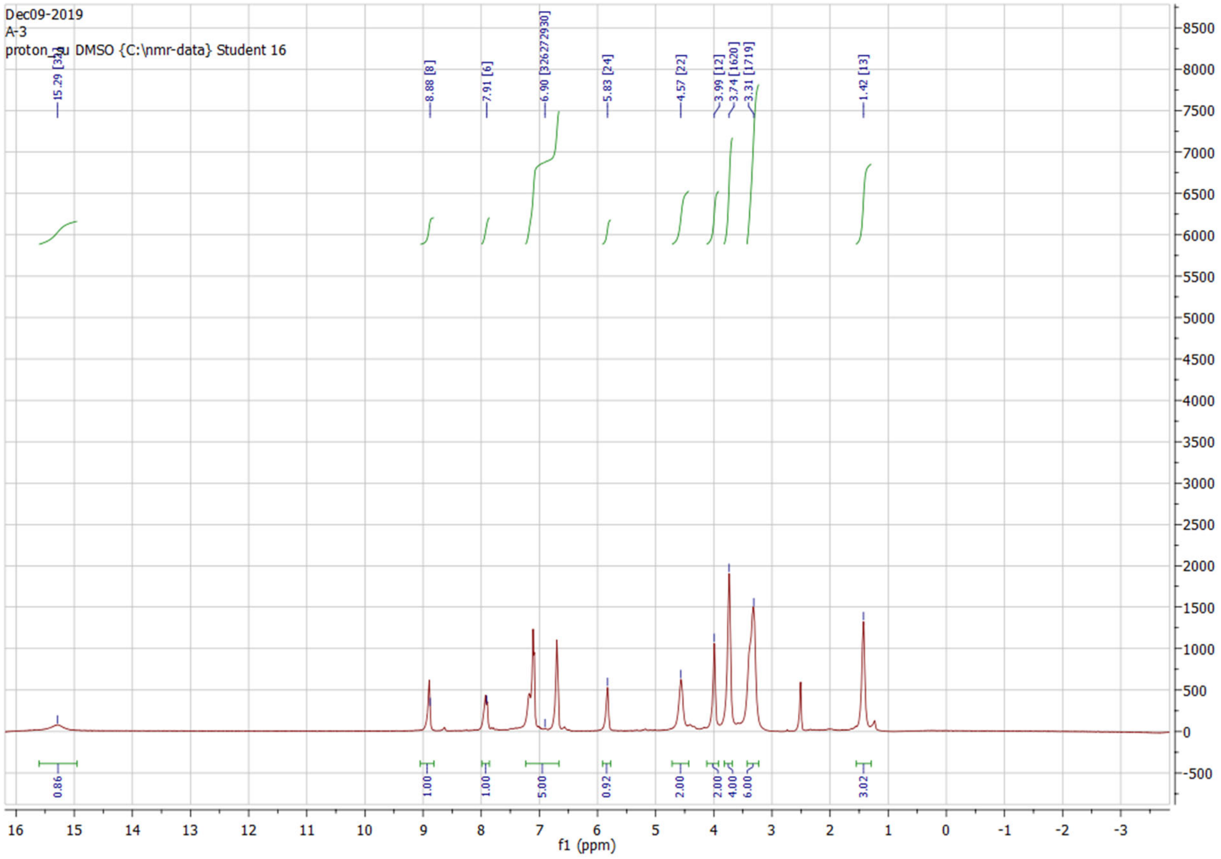

Figure S4a: ^{1}HNMR

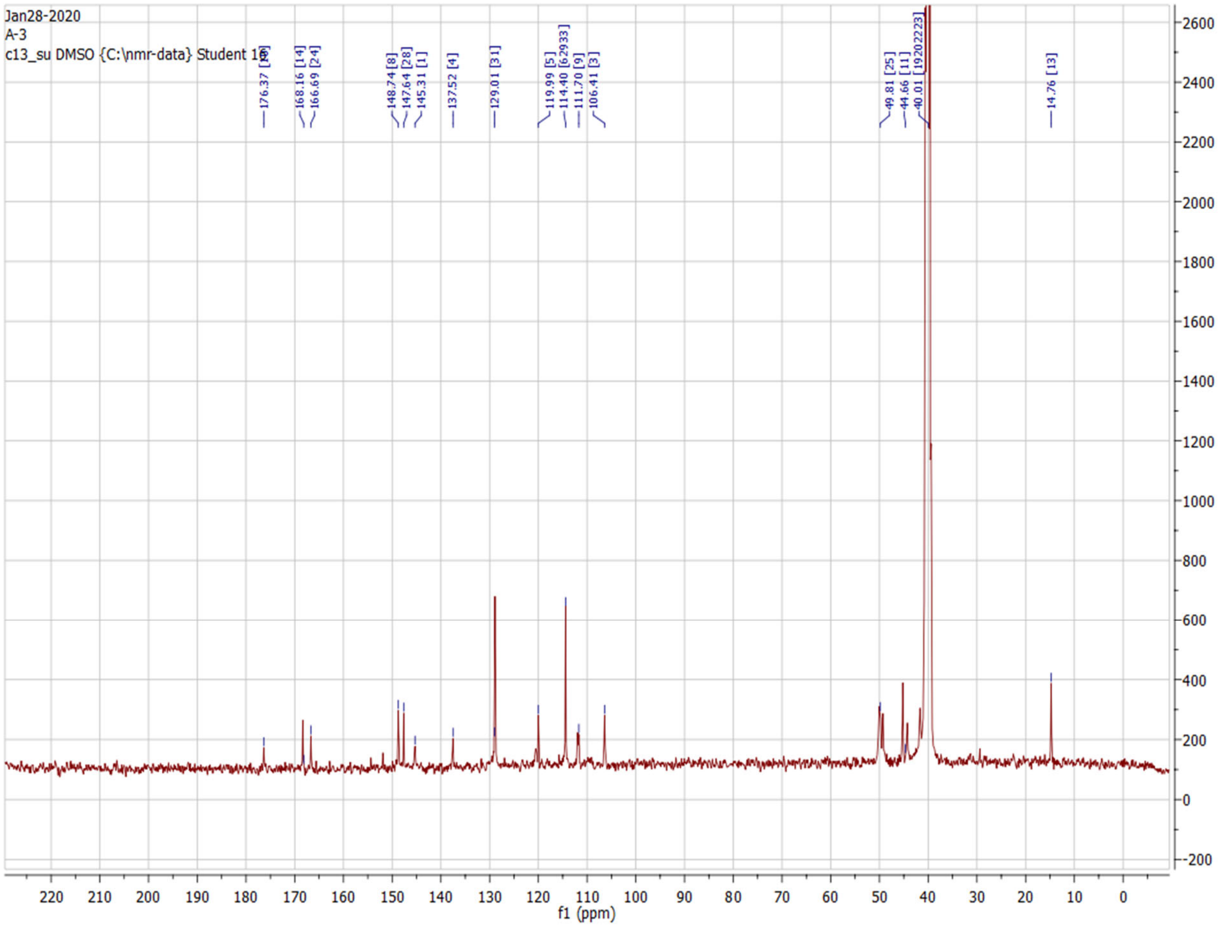

Figure S4b: ^{13}CNMR

Compound 4d

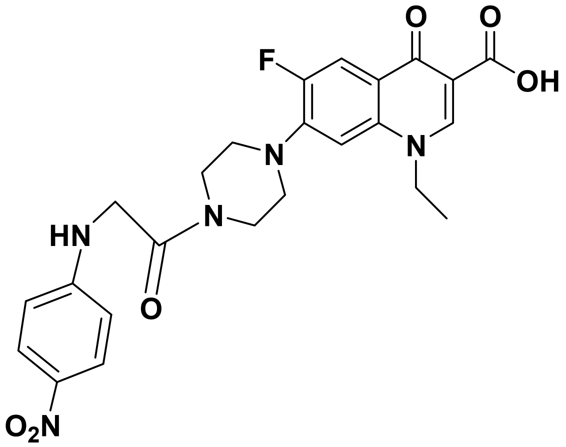

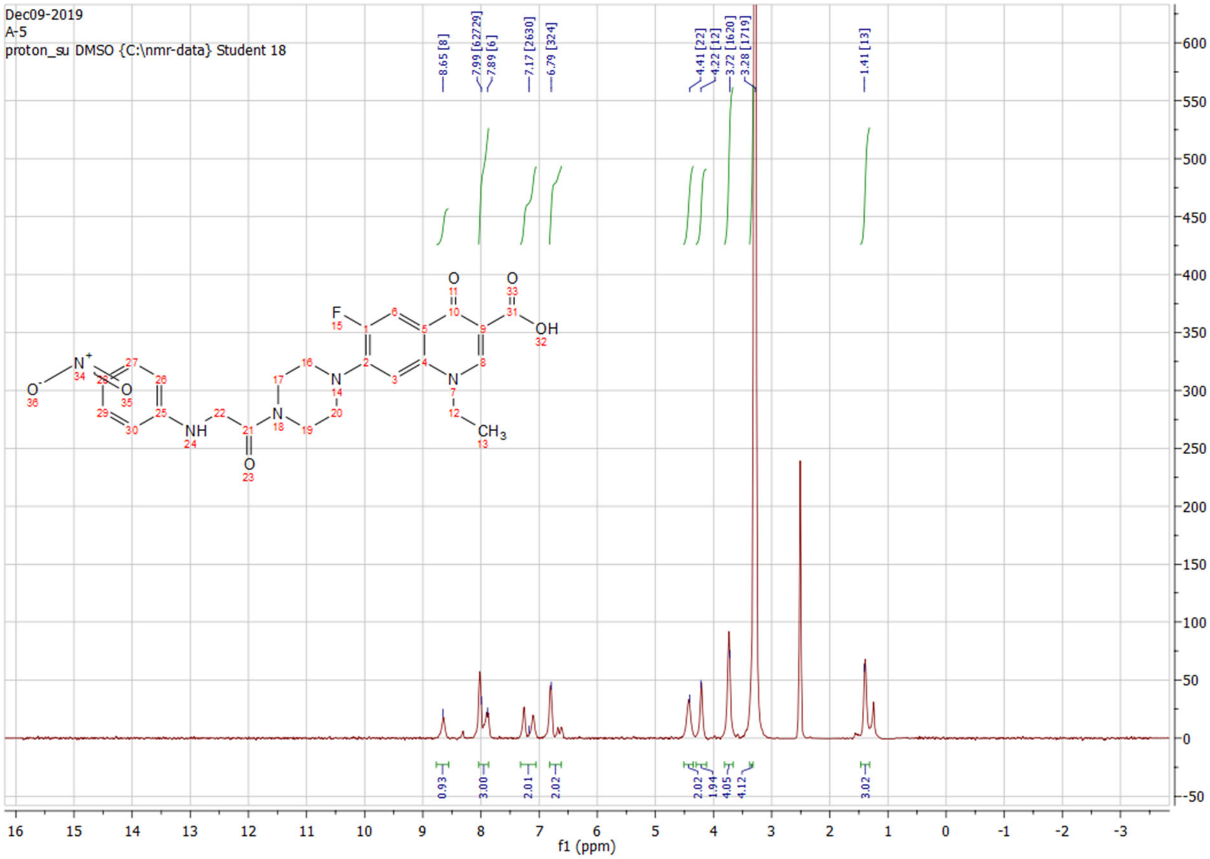

Figure S5a: ^{1}HNMR

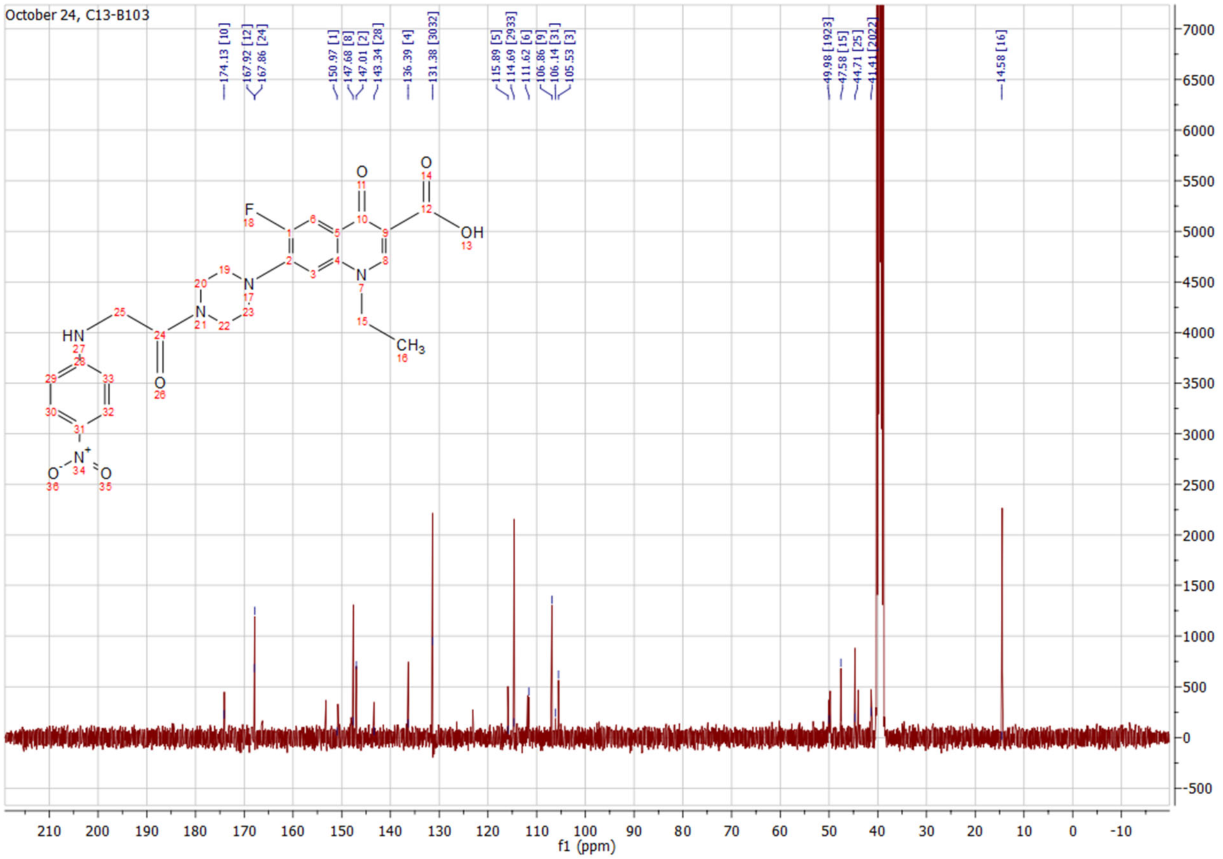

Figure S5b: ^{13}CNMR

Compound 4e

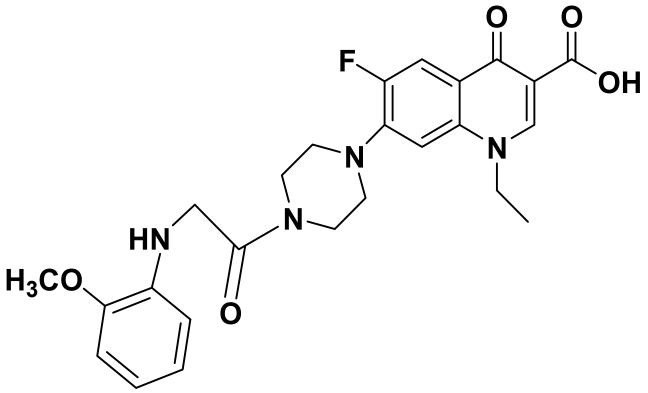

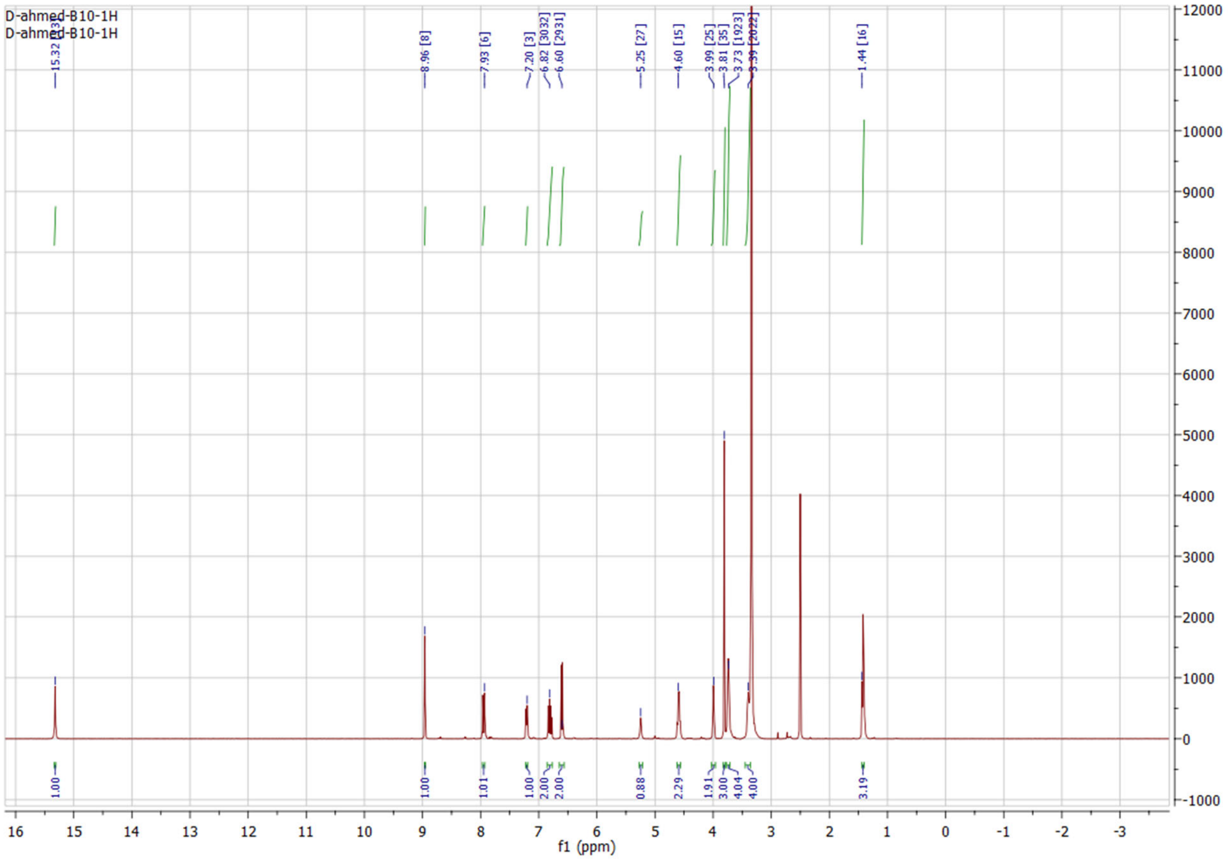

Figure S6a: ^{1}HNMR

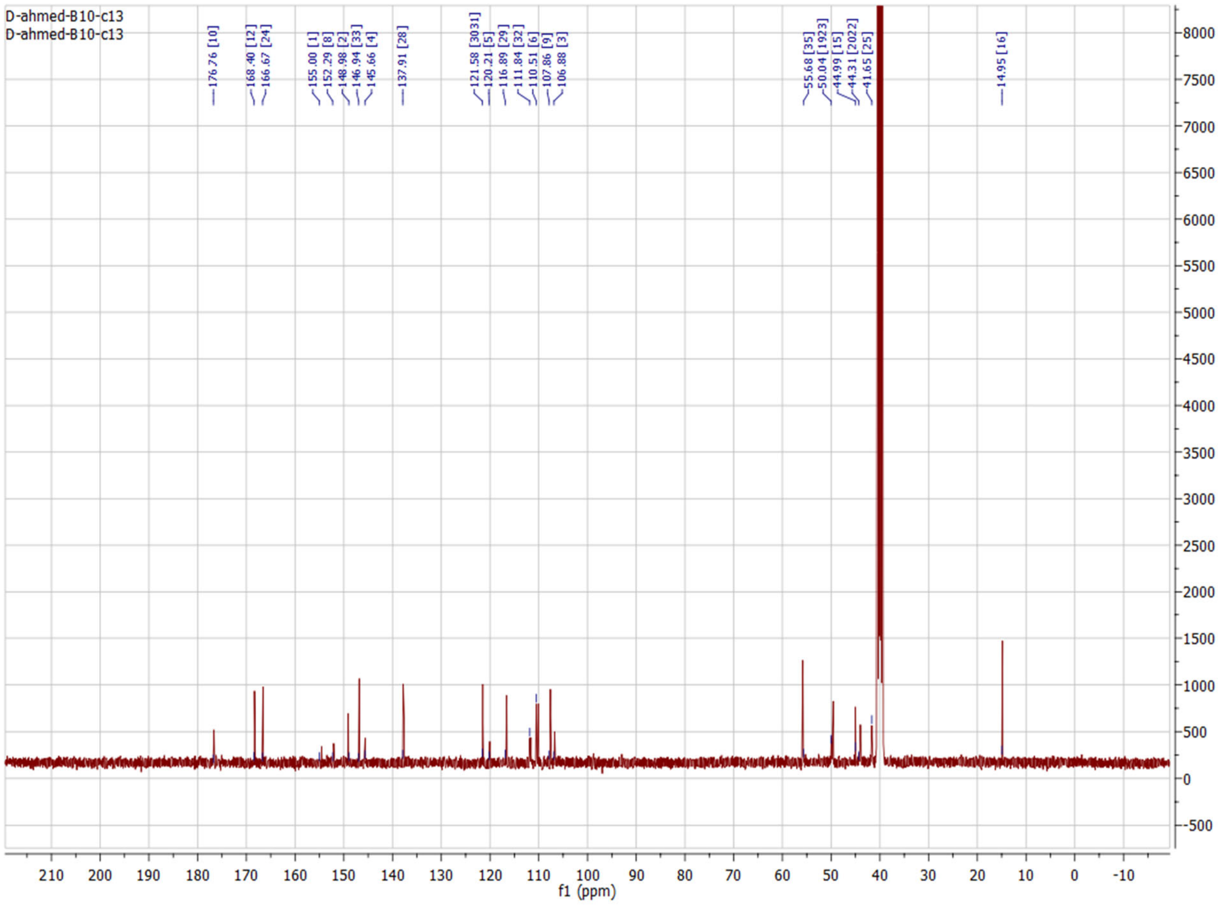

Figure S6b: ^{13}CNMR

Compound 4f

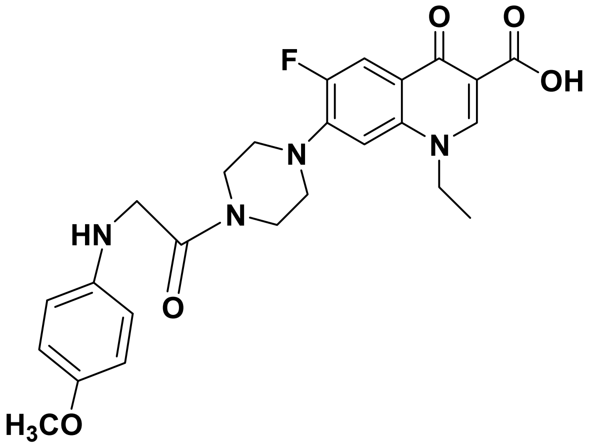

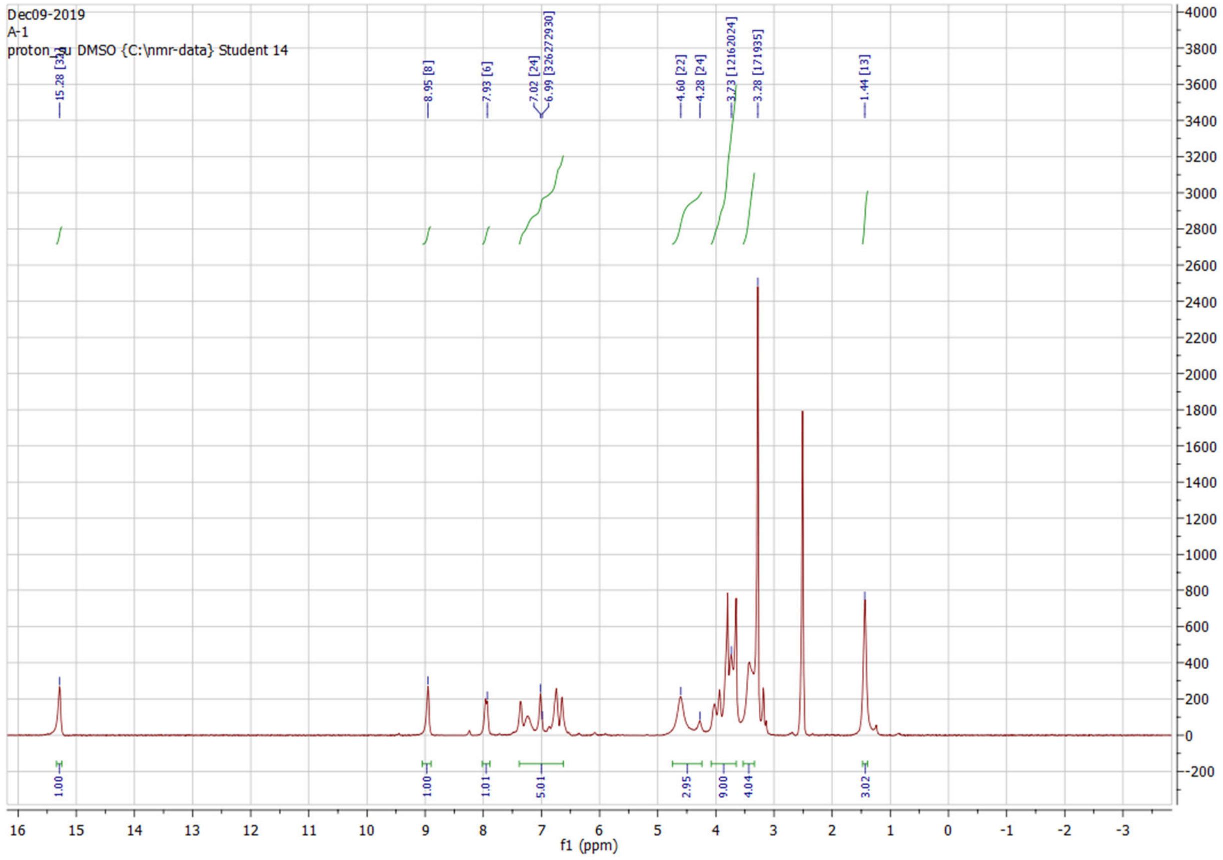

Figure S7a: ^{1}HNMR

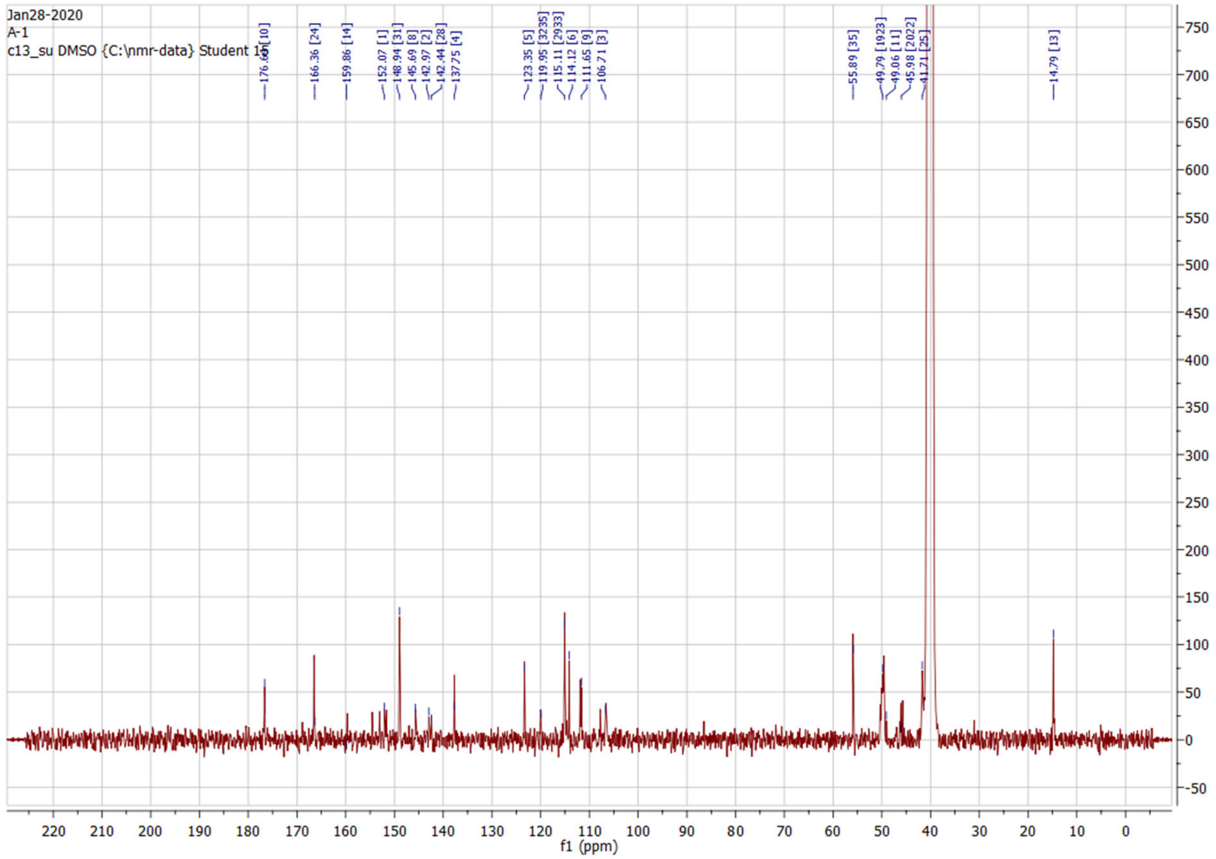

Figure S7b: ^{13}CNMR

Compound 3b

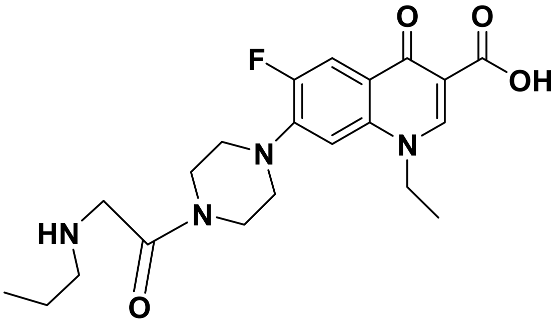

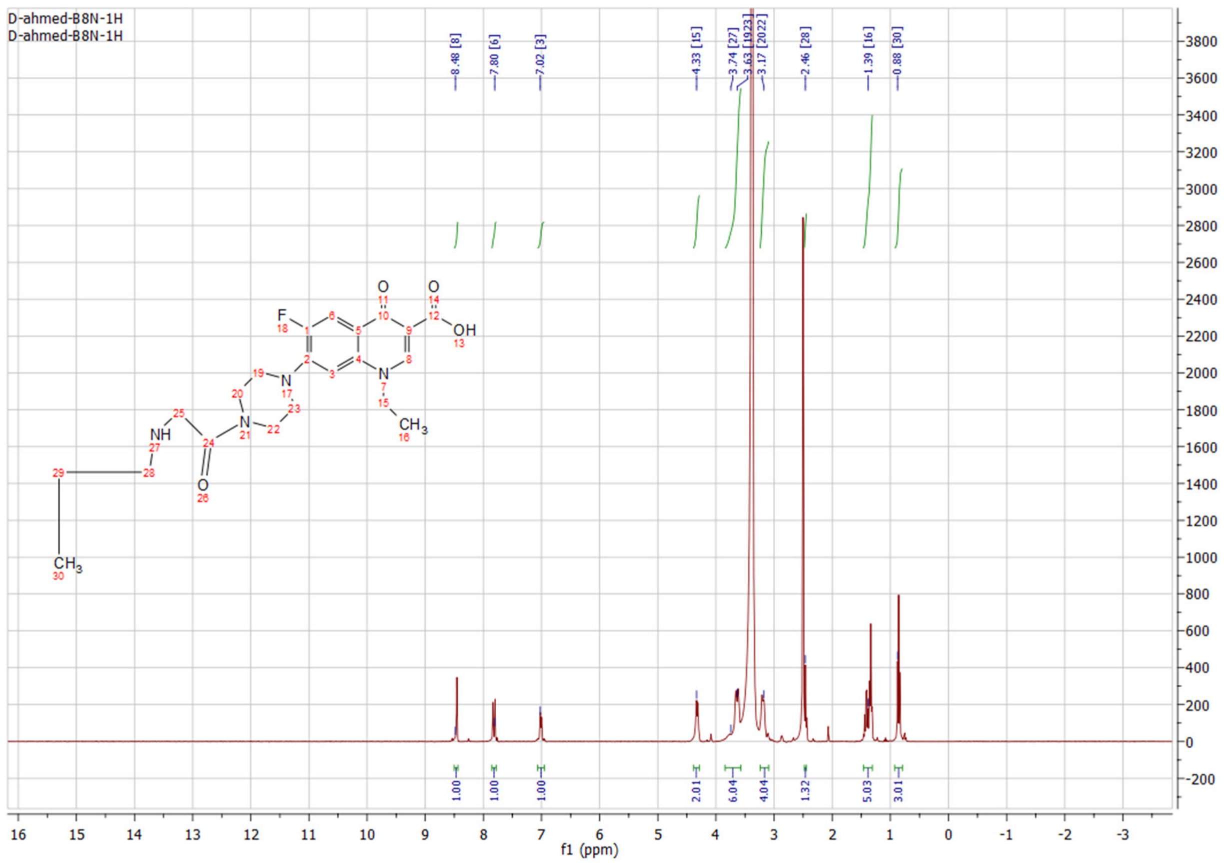

Figure S8a: ^{1}HNMR

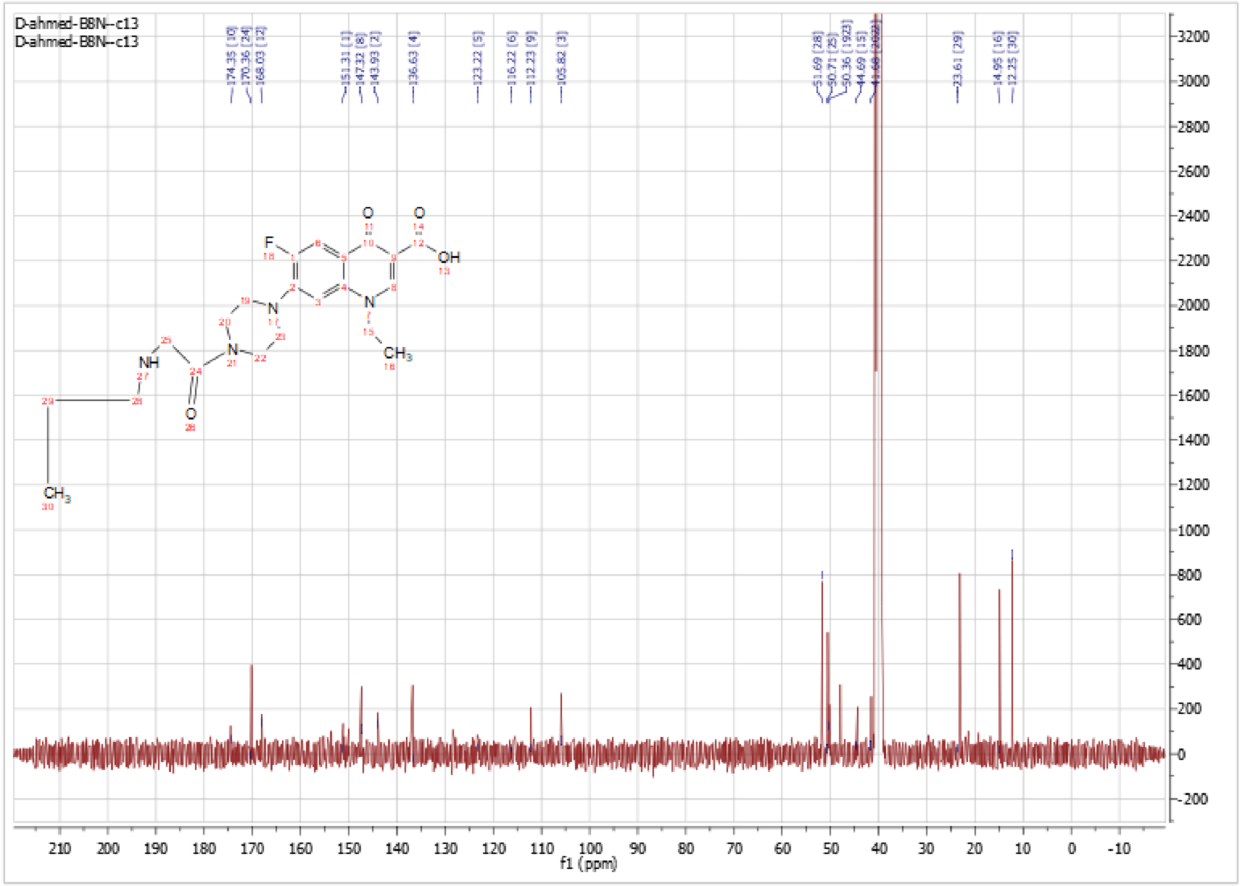

Figure S8b: ^{13}CNMR

Compound 4k

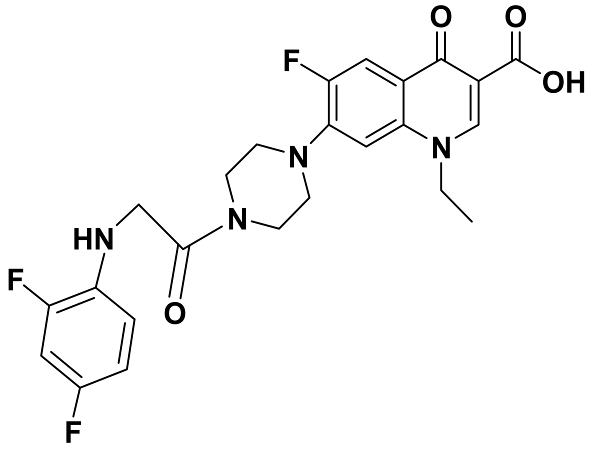

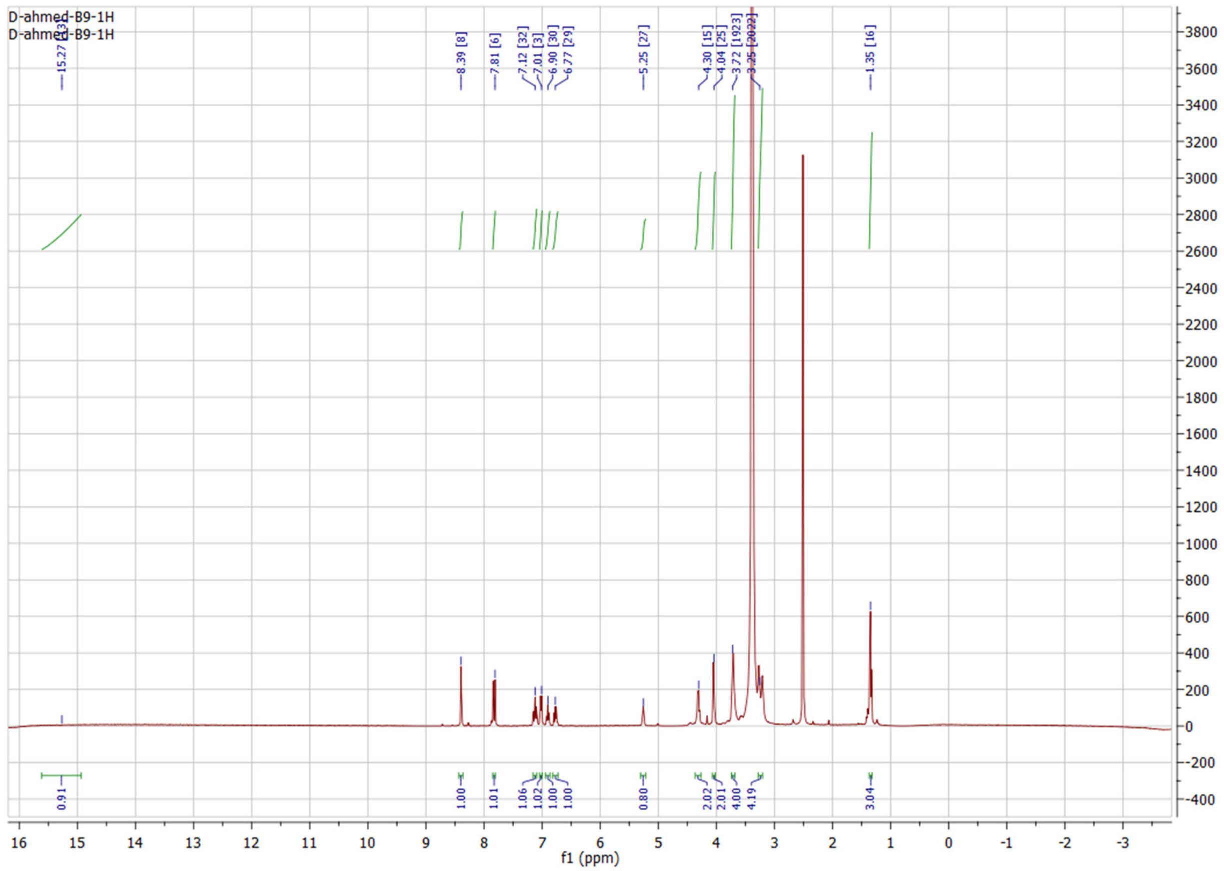

Figure S9a: ^{1}HNMR

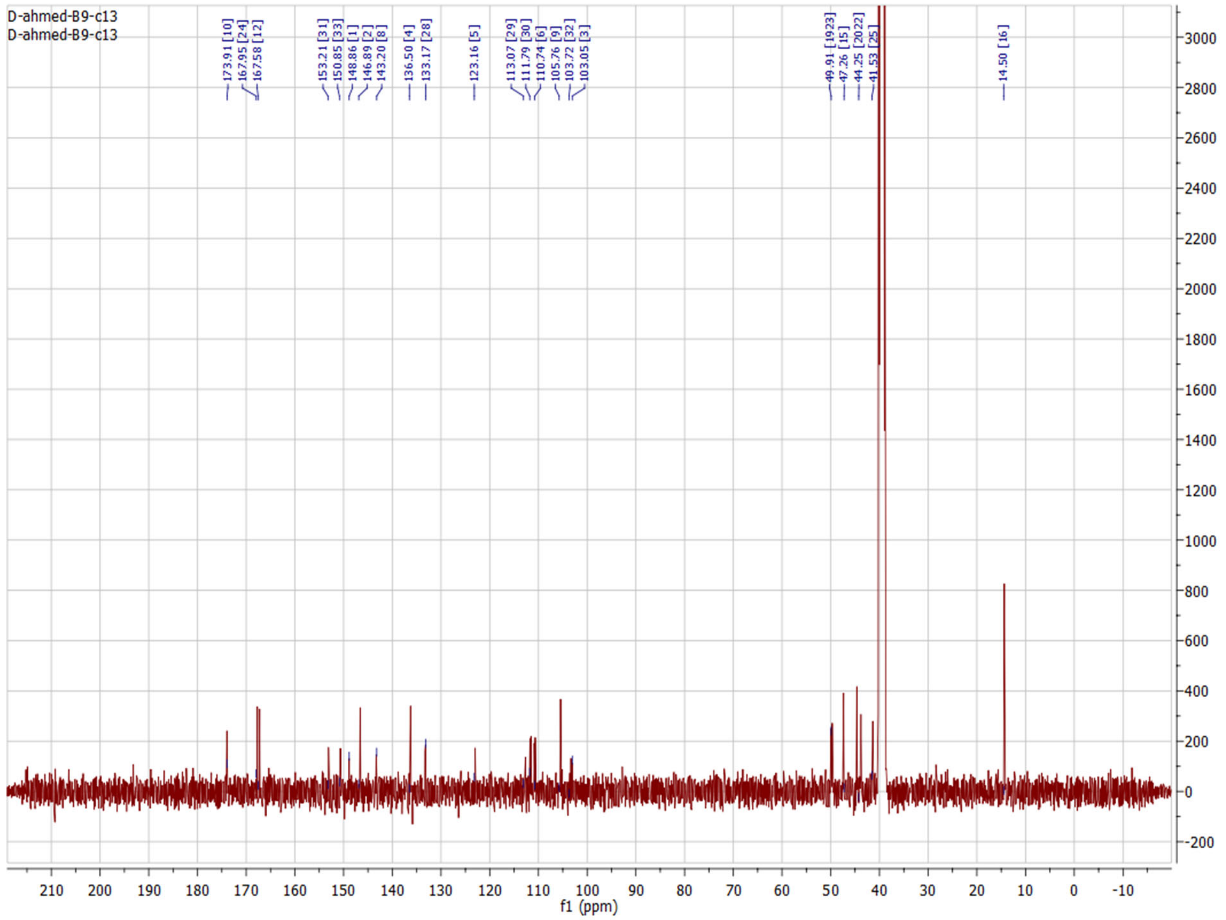

Figure S9b: ^{13}CNMR

Compound 7a

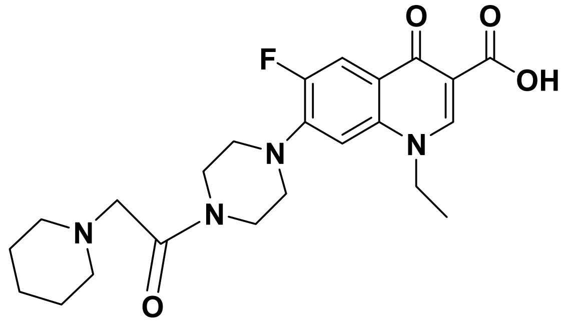

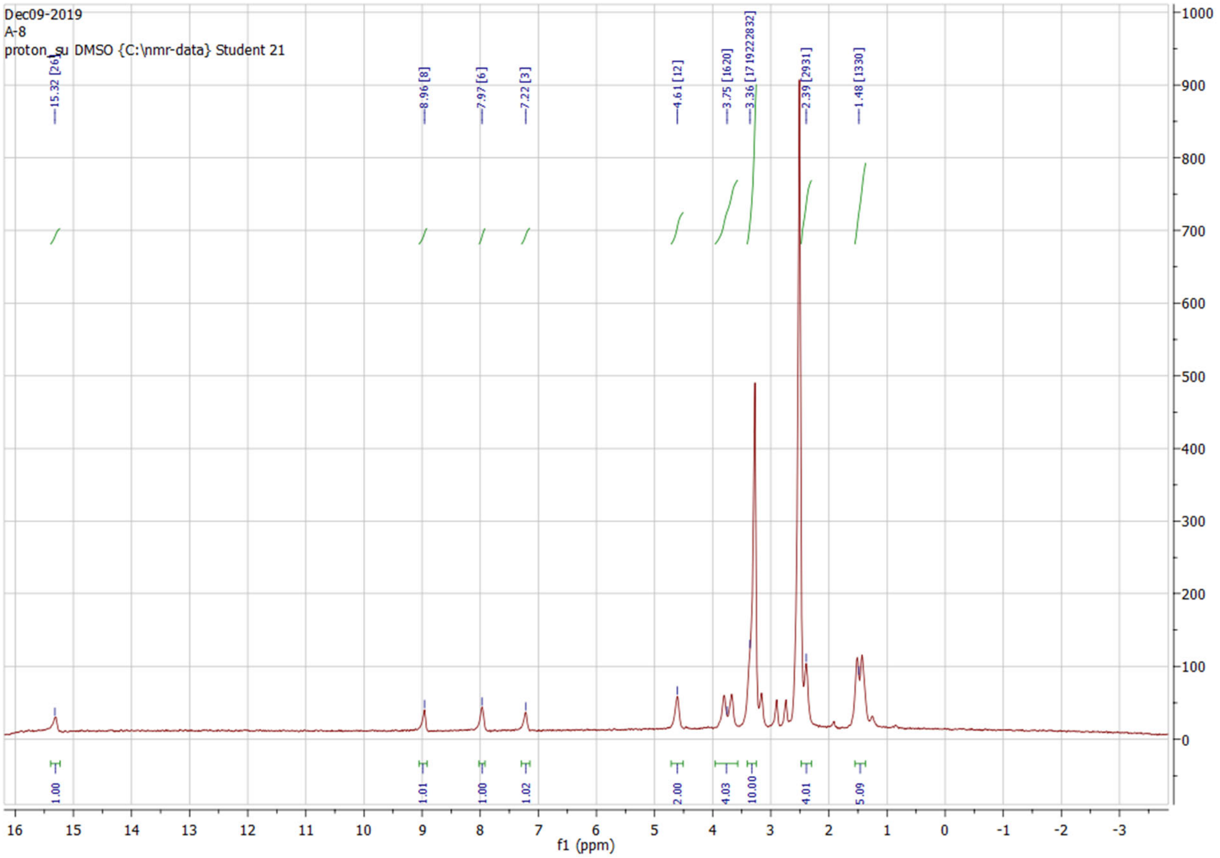

Figure S10a: ^{1}HNMR

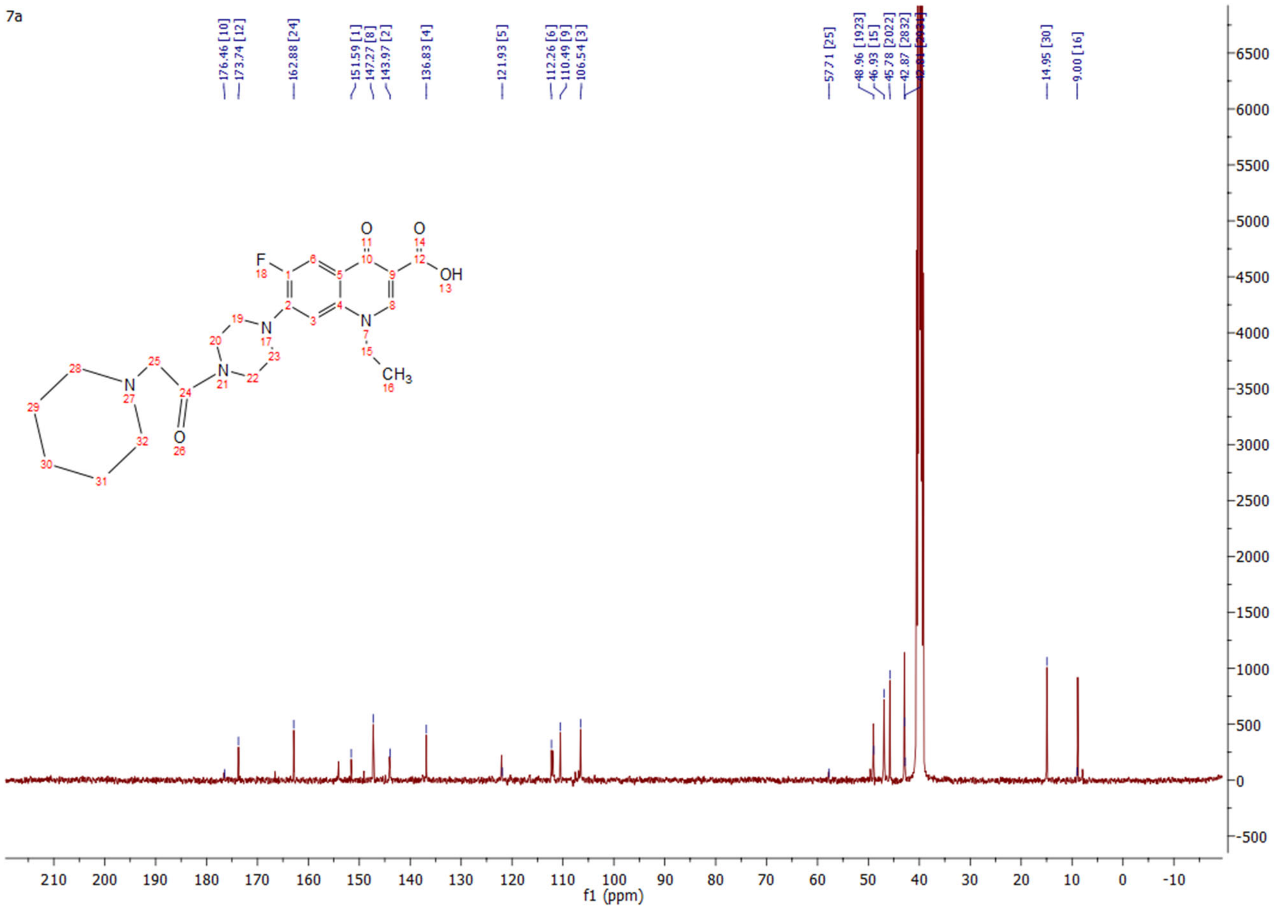

Figure S10b: ^{13}CNMR

Compound 4l

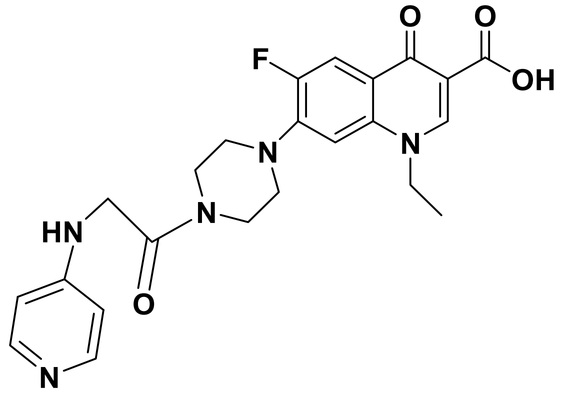

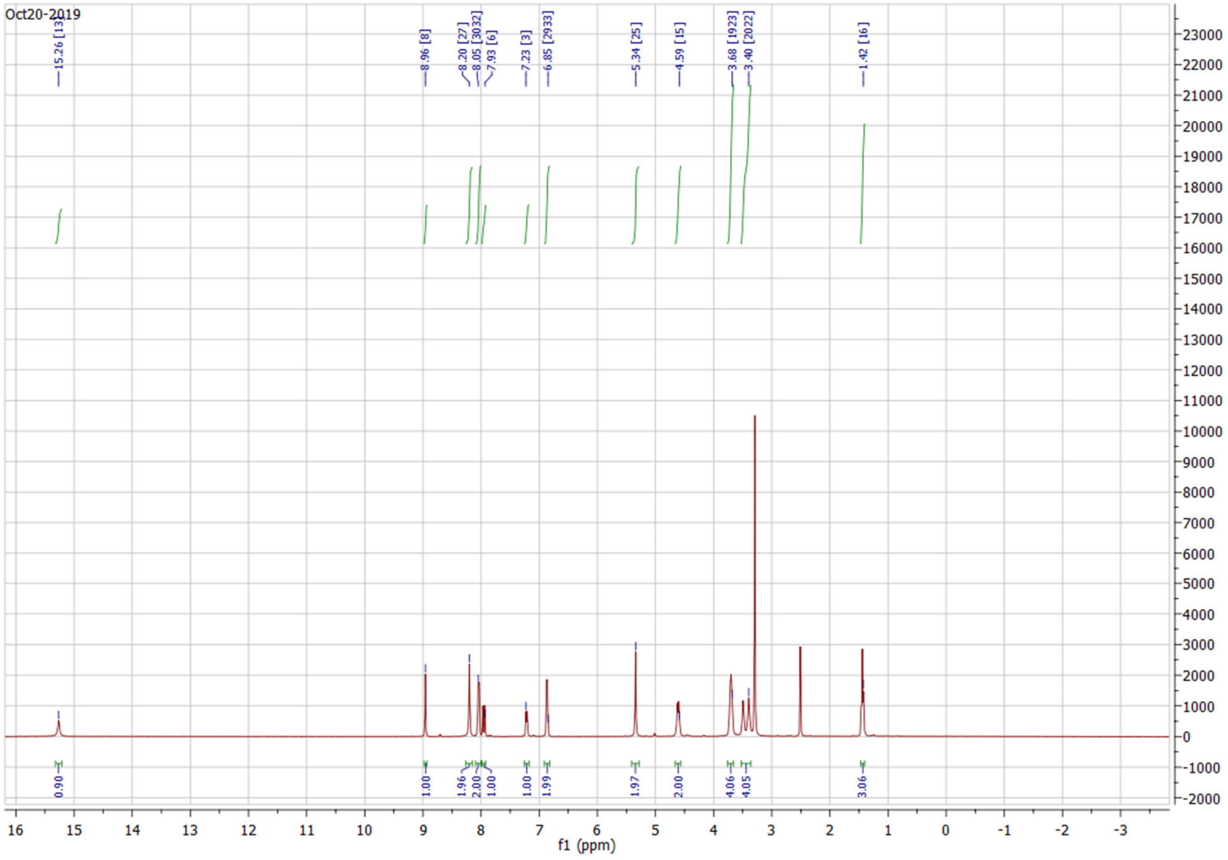

Figure S11a: ^{1}HNMR

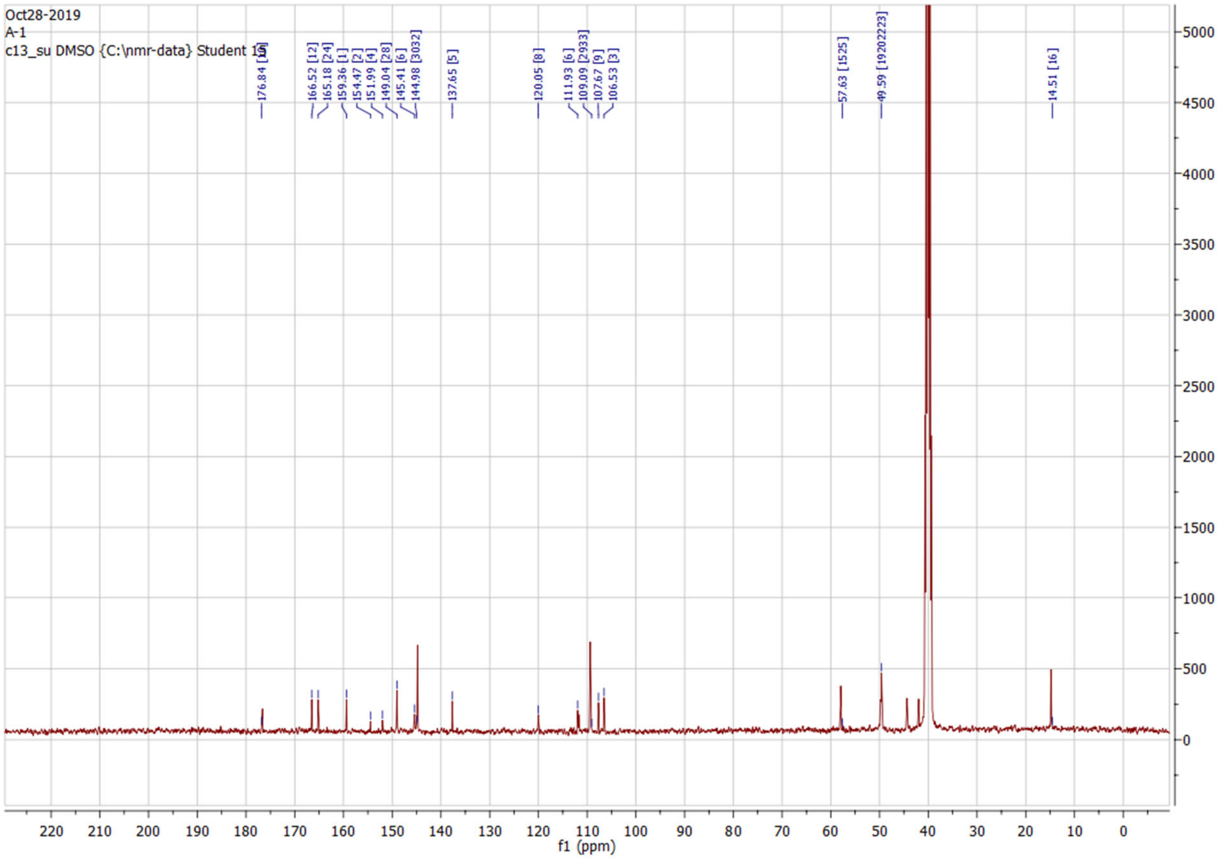

Figure S11b: ^{13}CNMR

Compound 4h

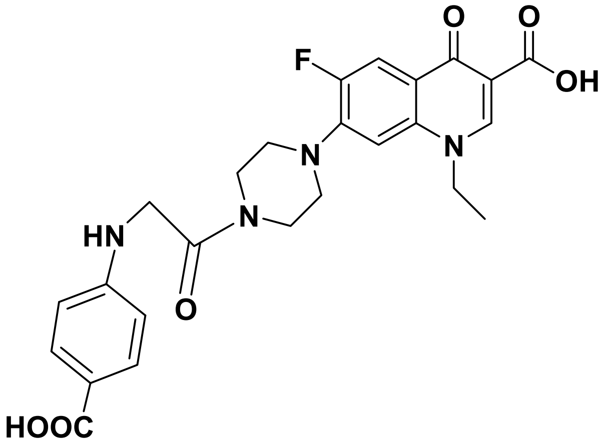

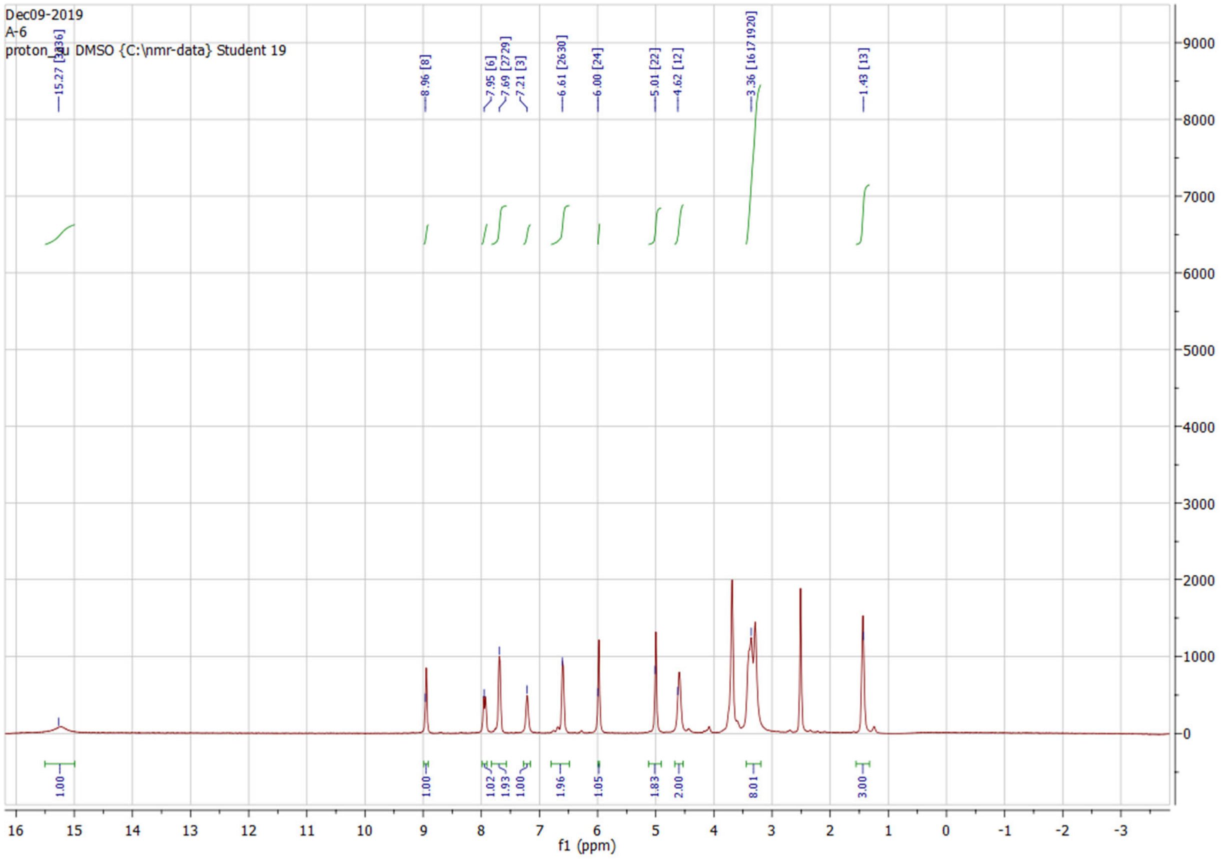

Figure S12a: ^{1}HNMR

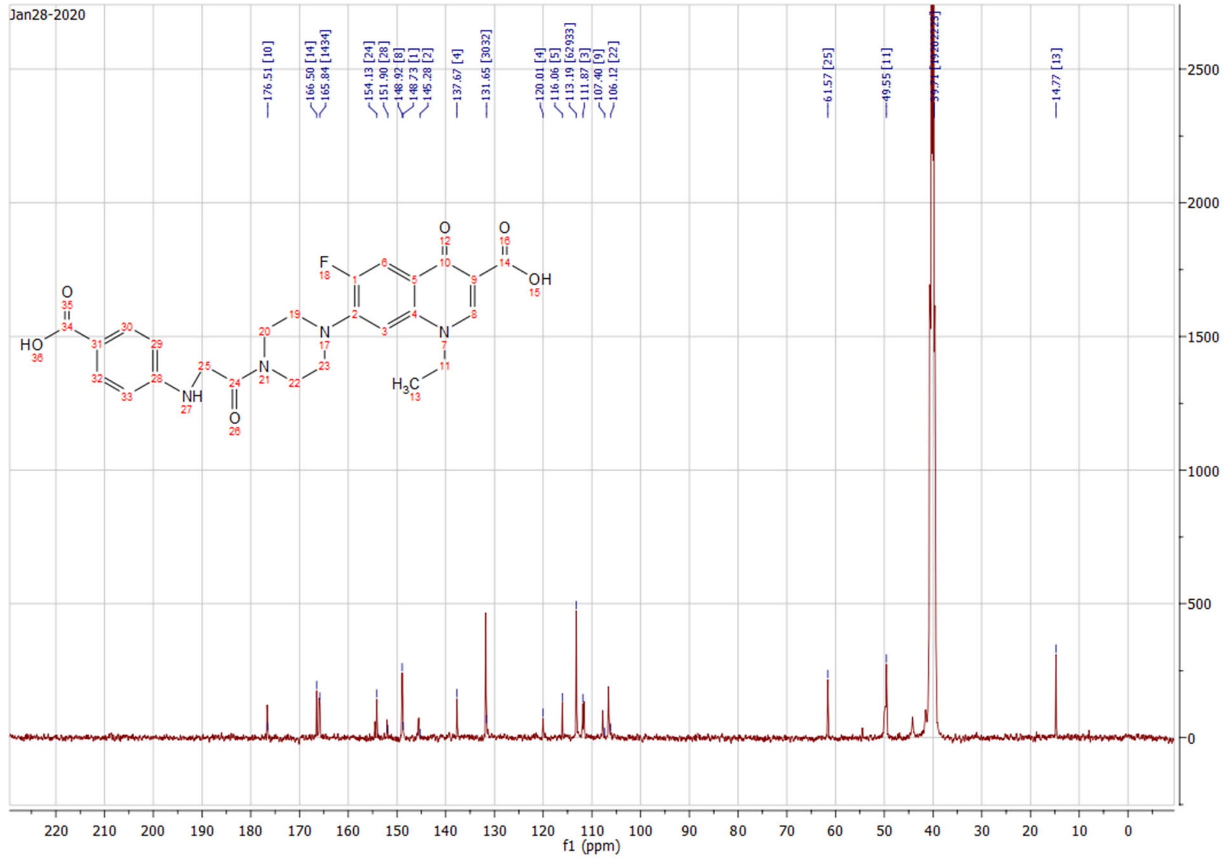

Figure S12b: ^{13}CNMR

Compound 7b

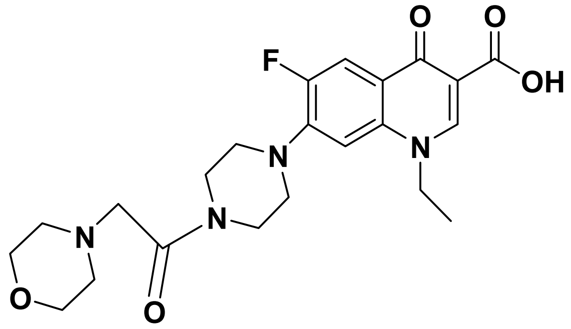

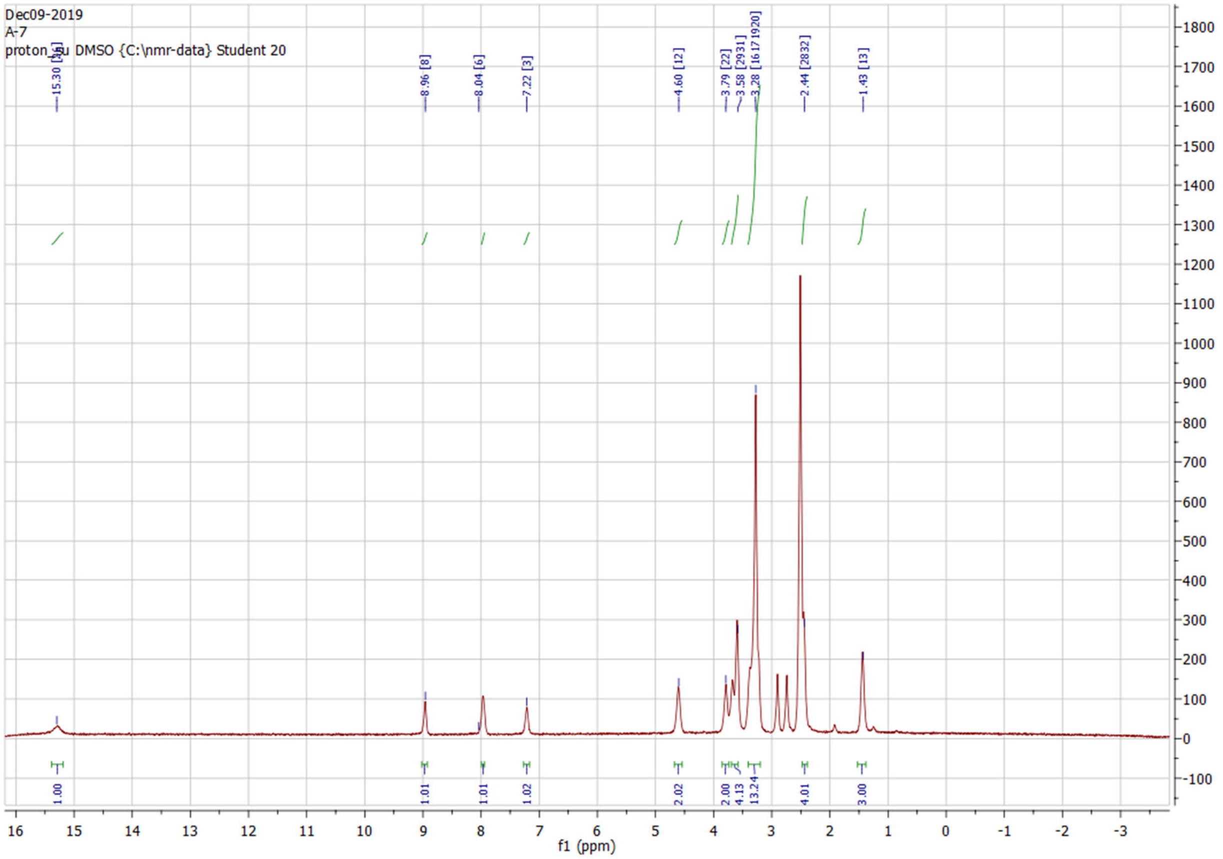

Figure S13a: ^{1}HNMR

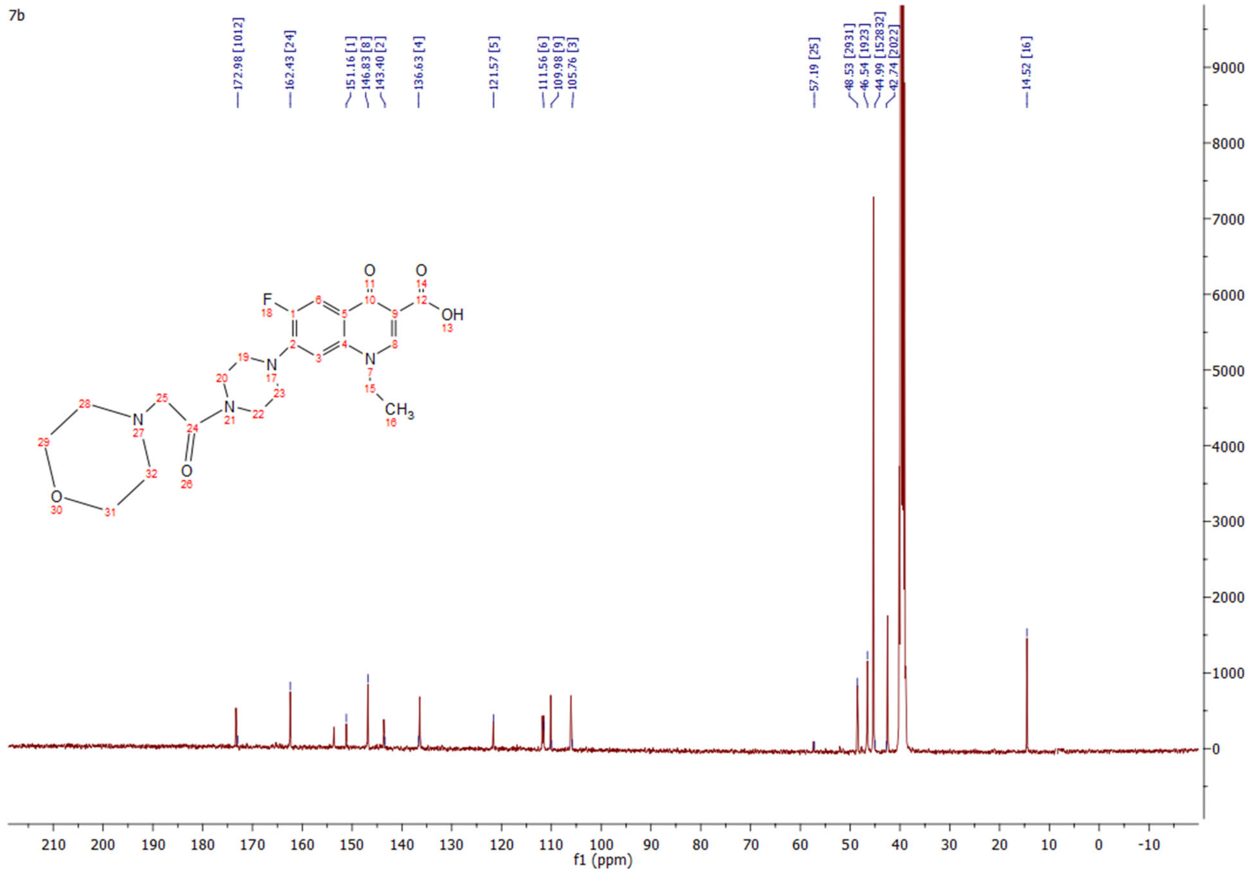

Figure S13b: ^{13}CNMR

Compound 4g

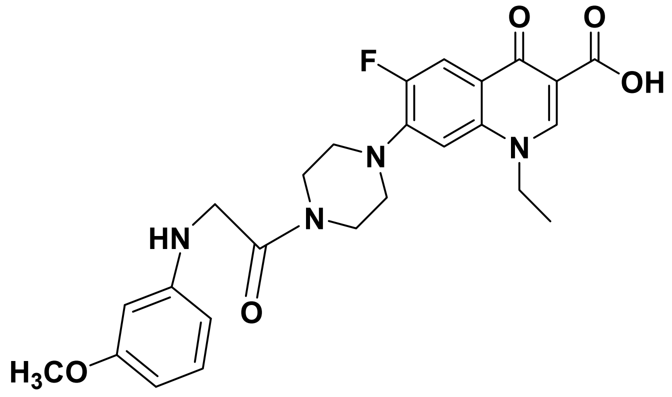

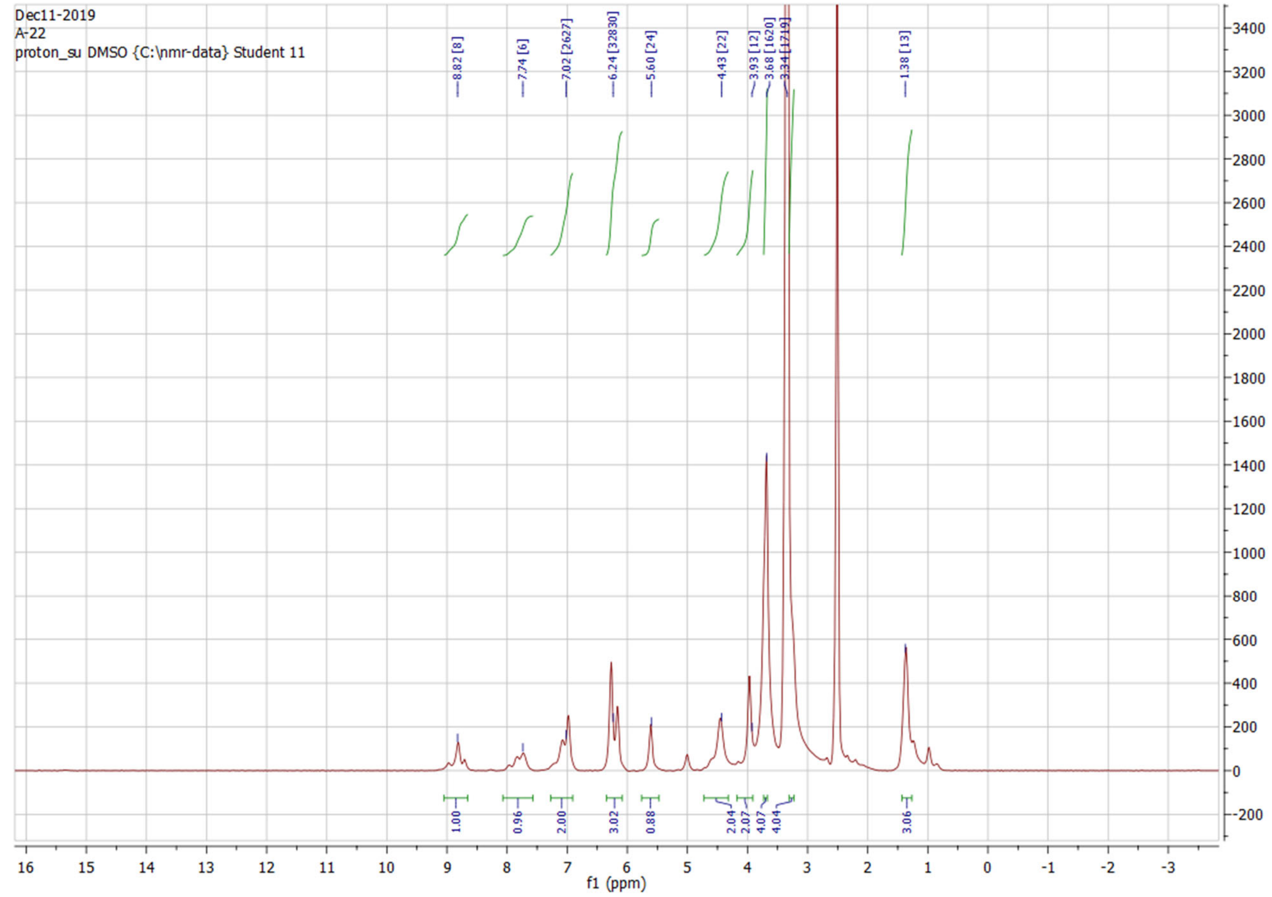

Figure S14a: ^{1}HNMR

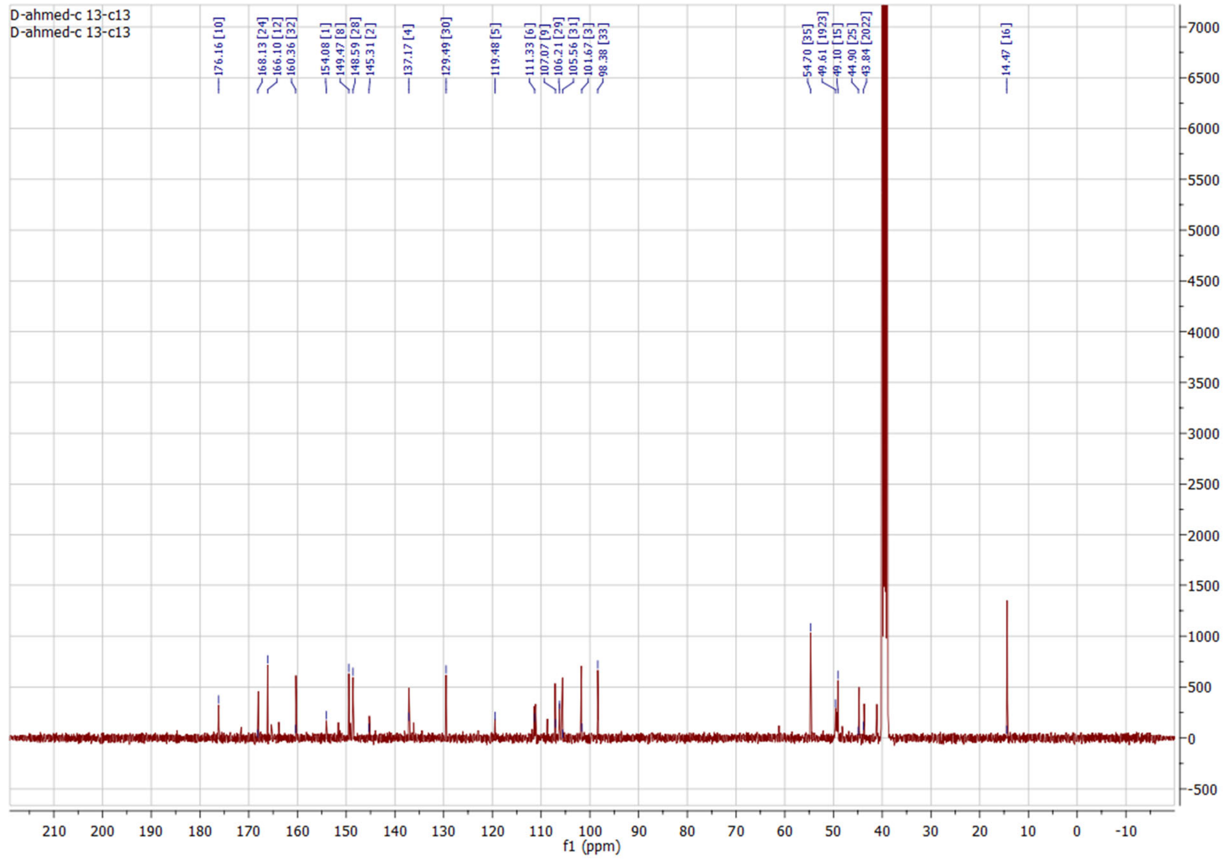

Figure S14b: ^{13}CNMR

Compound 3a

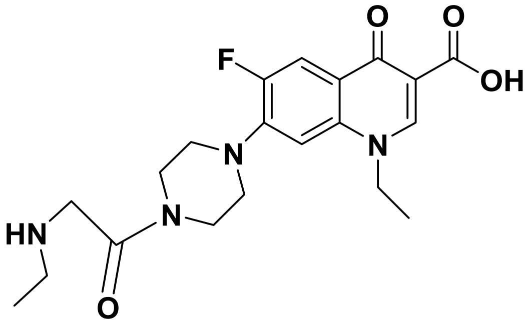

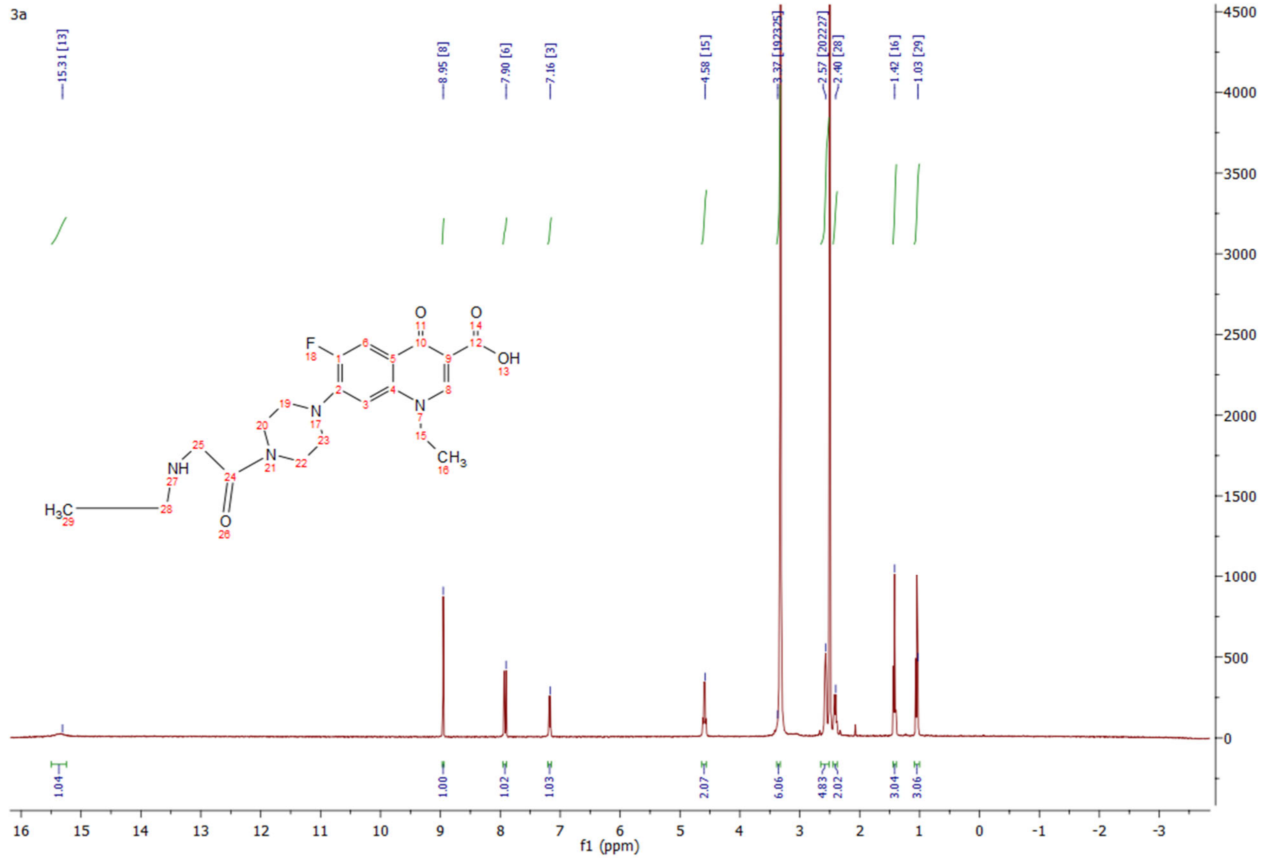

Figure S15a: ^{1}HNMR

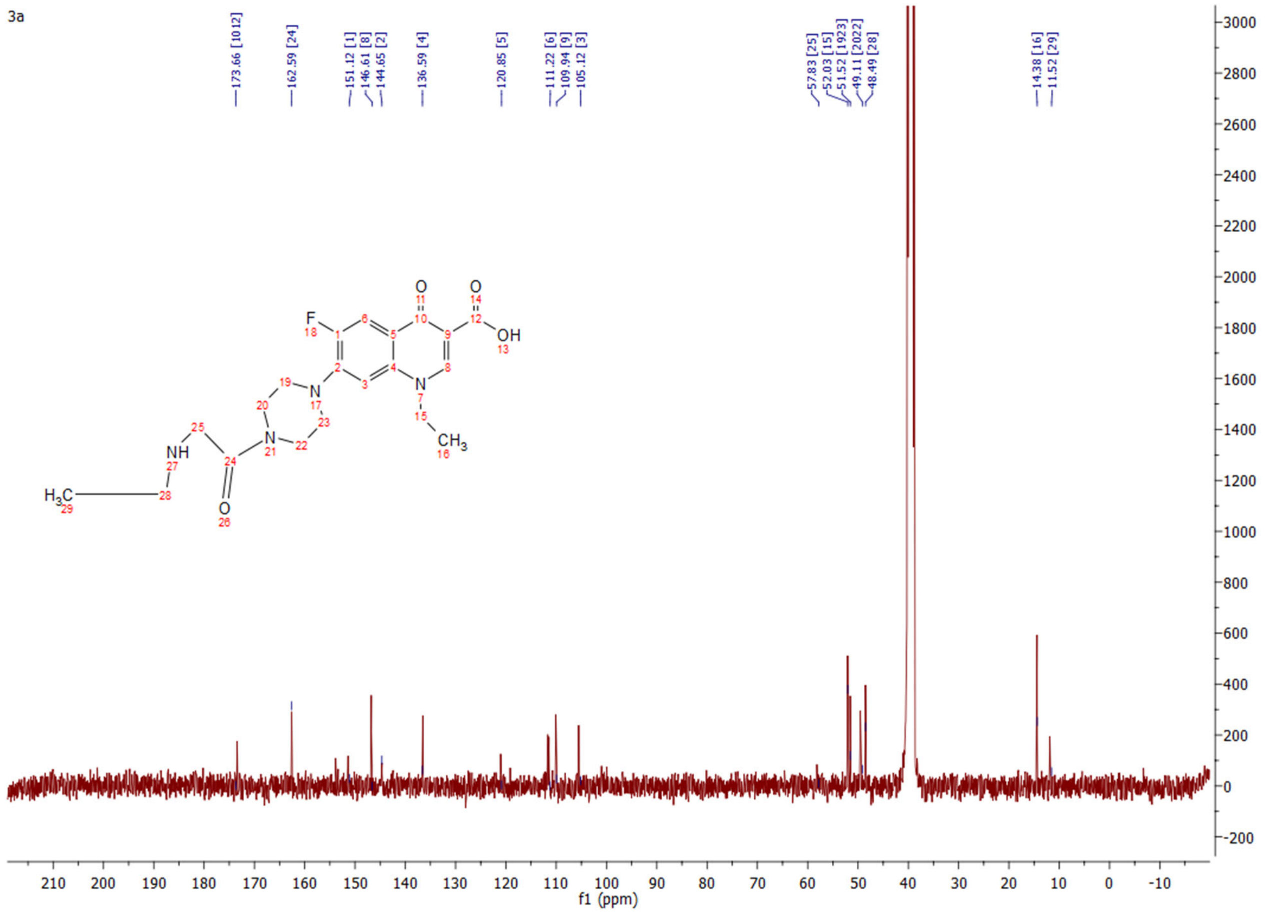

Figure S15b: ^{13}CNMR

Compound 16

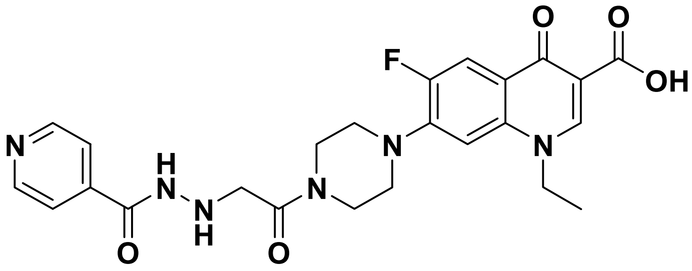

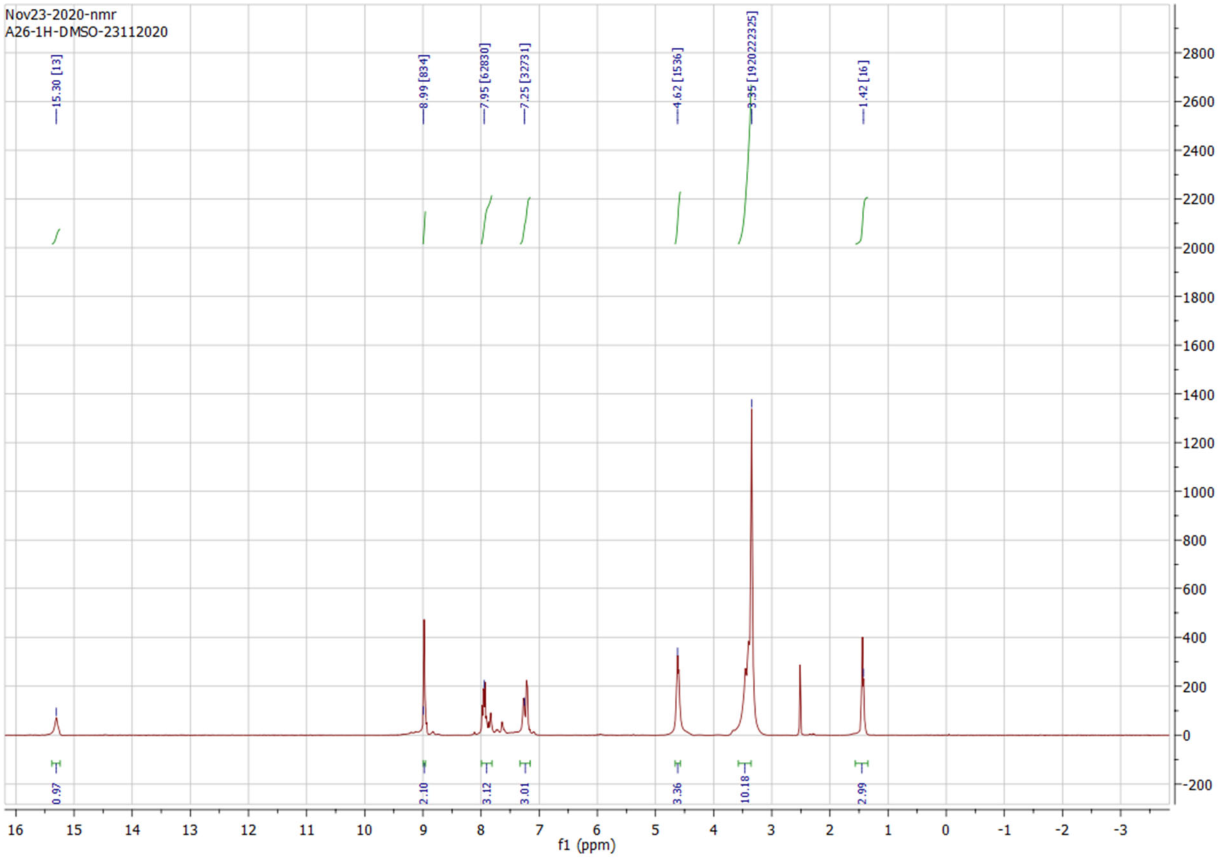

Figure S16a: ^{1}HNMR

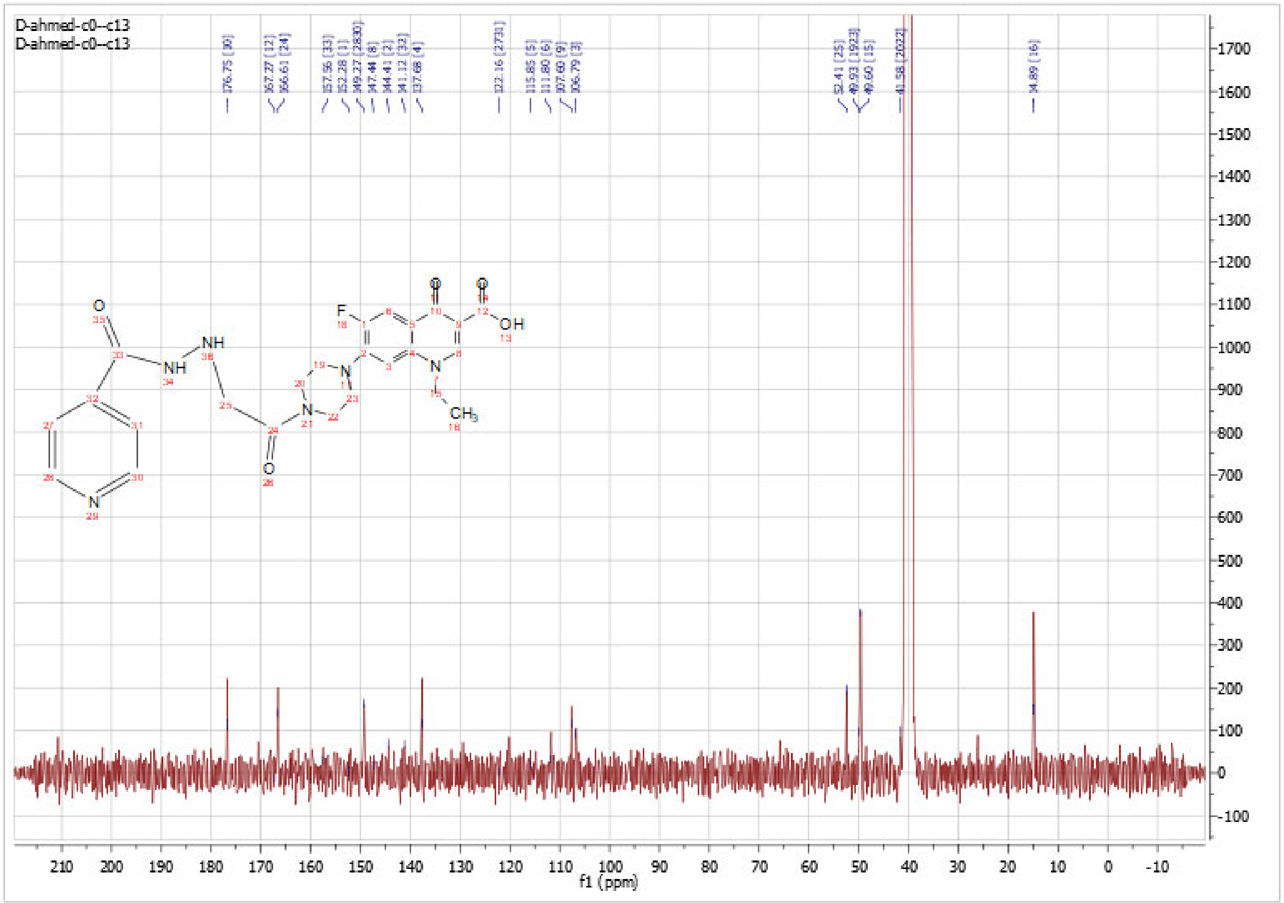

Figure S16b: ^{1}HNMR

Compound 6a

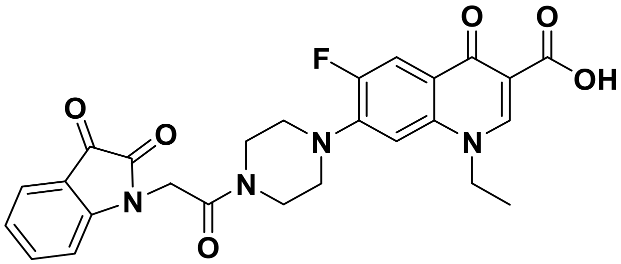

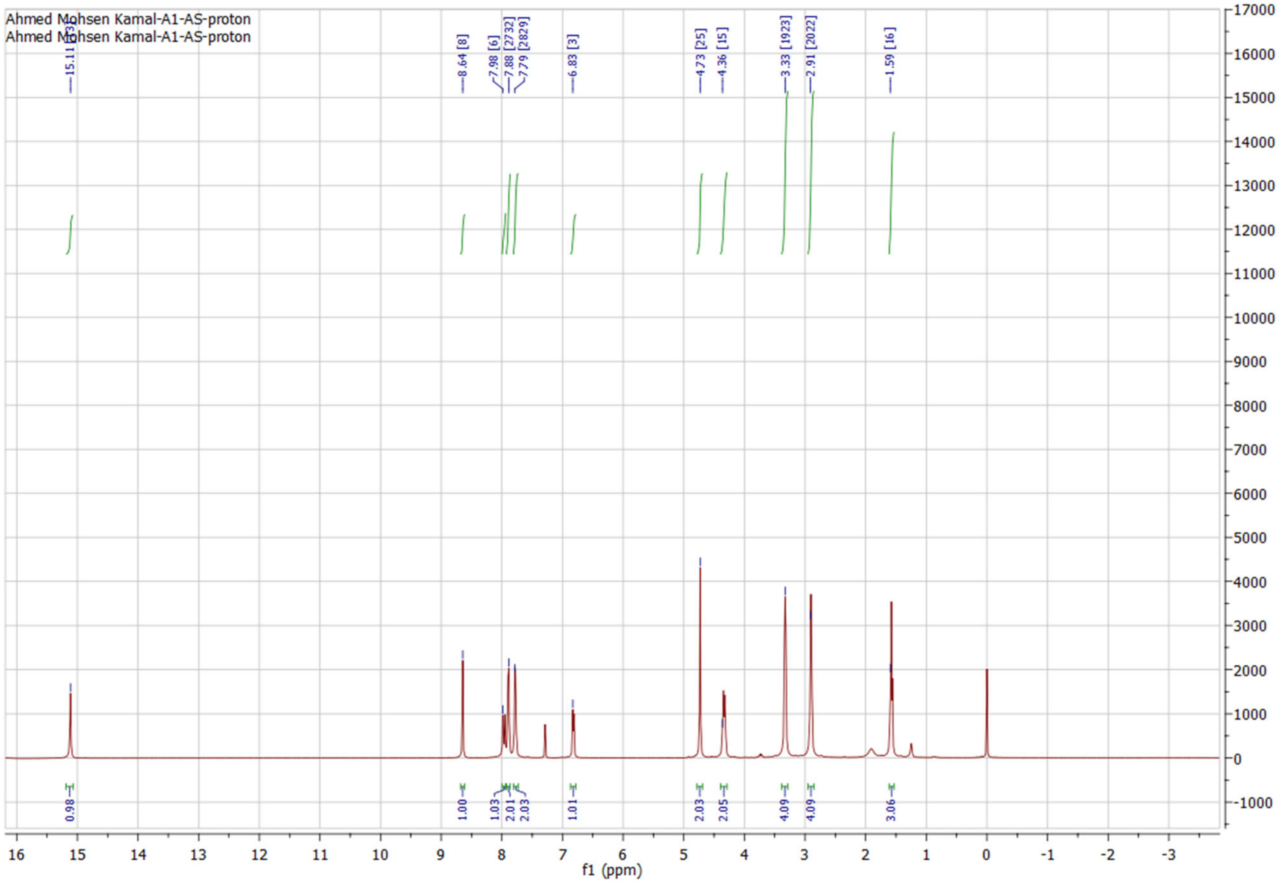

Figure S17a: ^{1}HNMR

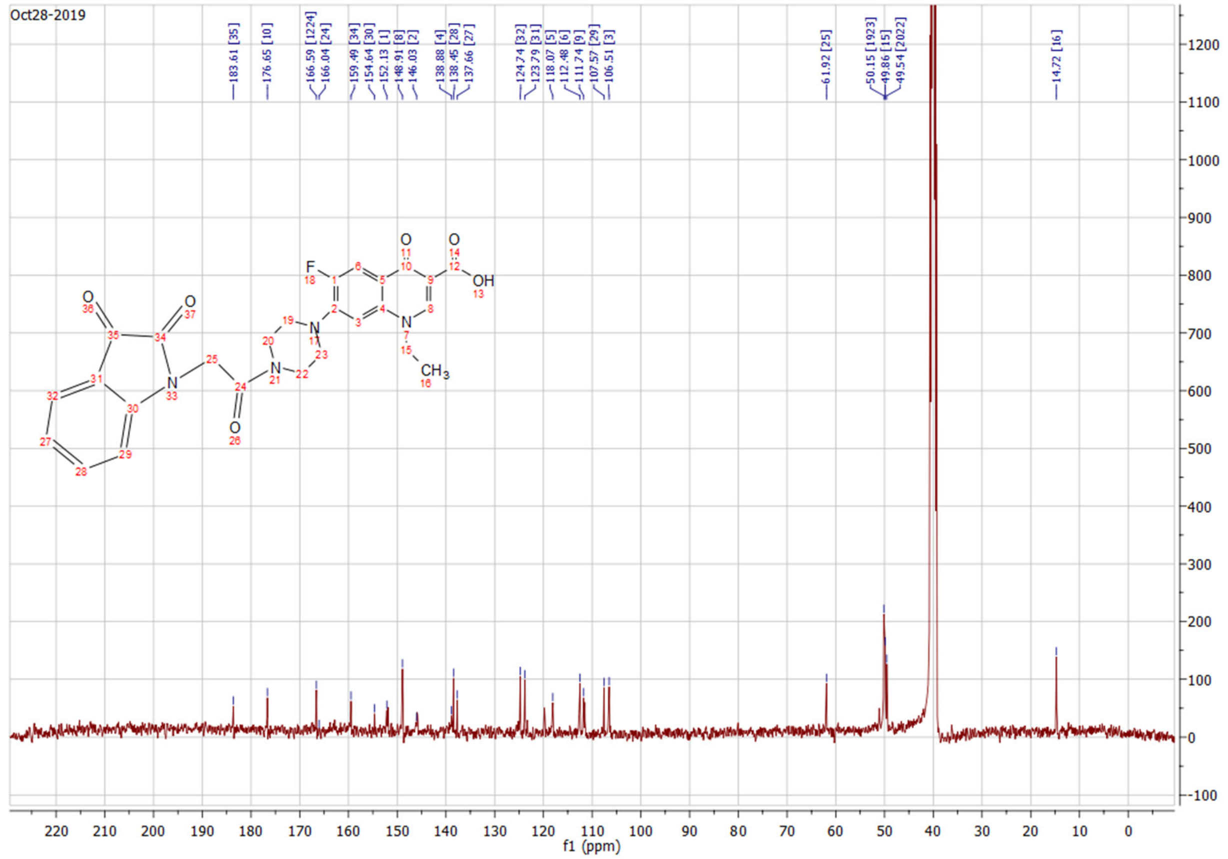

Figure S17b: ^{13}CNMR

Compound 6b

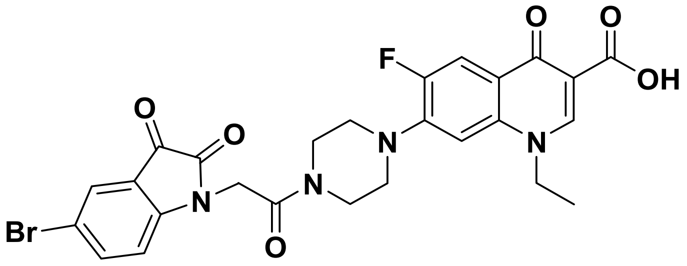

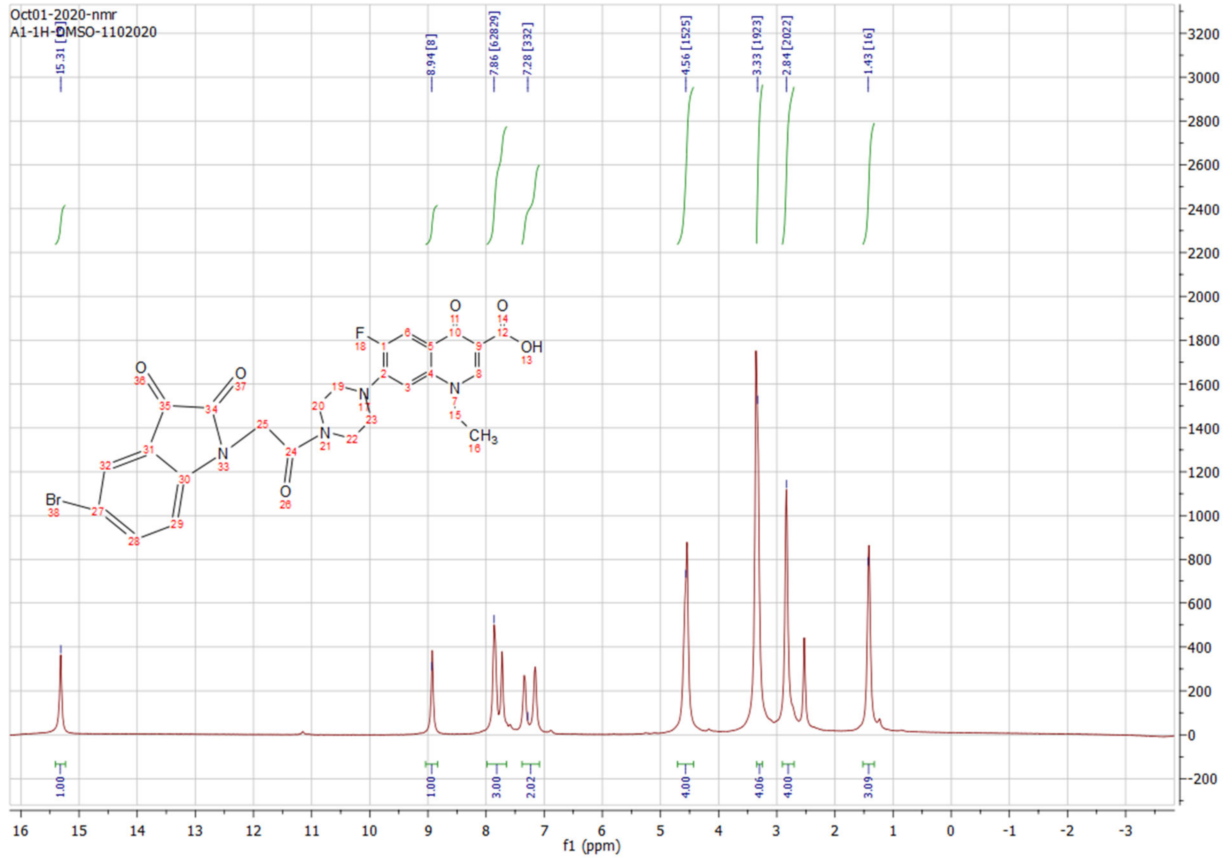

Figure S18a: ^{1}HNMR

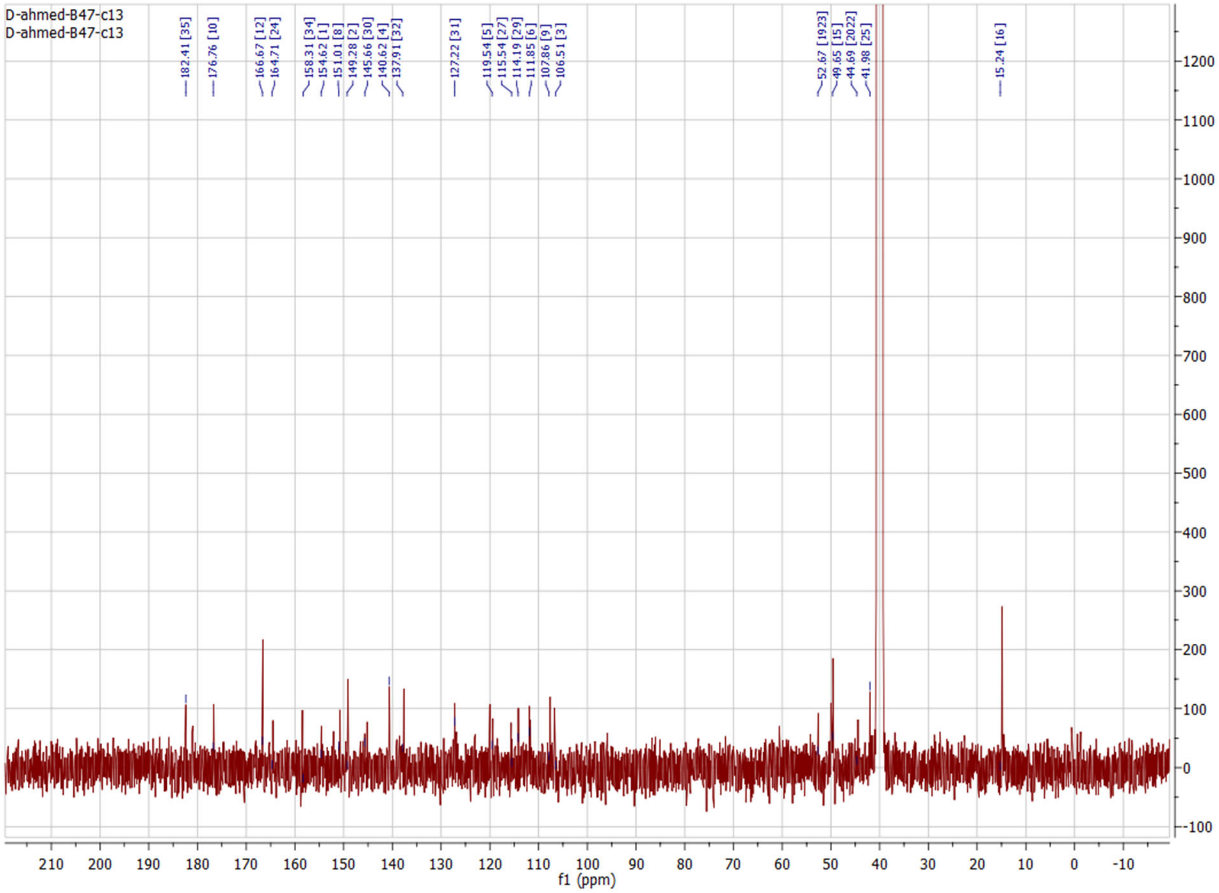

Figure S18b: ^{13}CNMR

Compound 5a

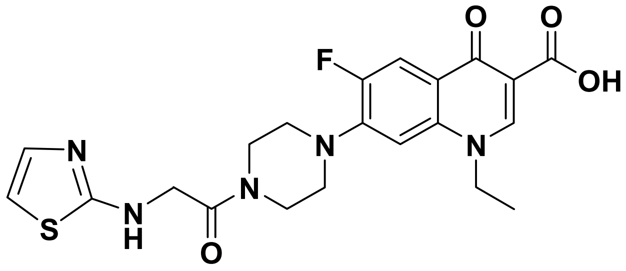

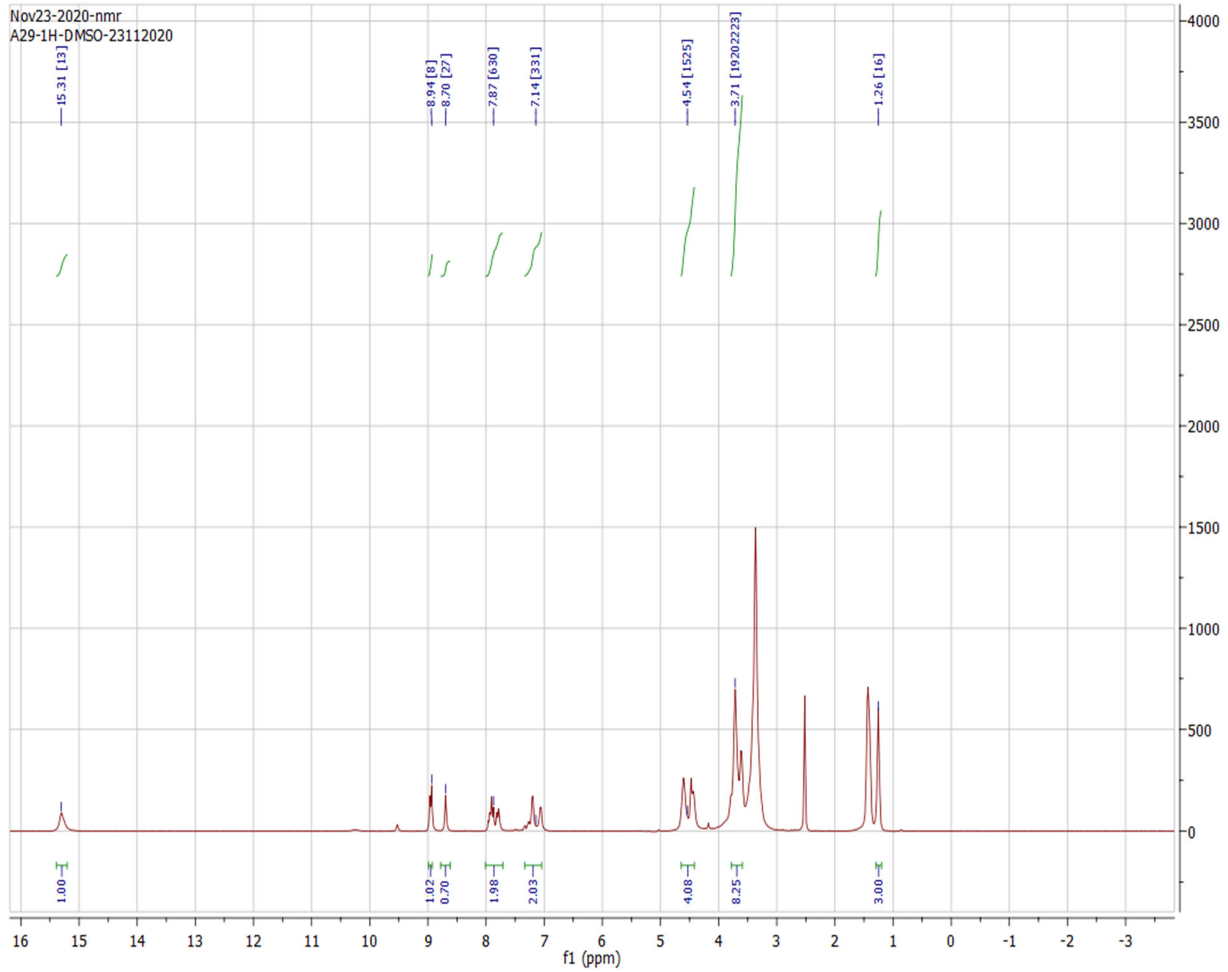

Figure S19a: ^{1}HNMR

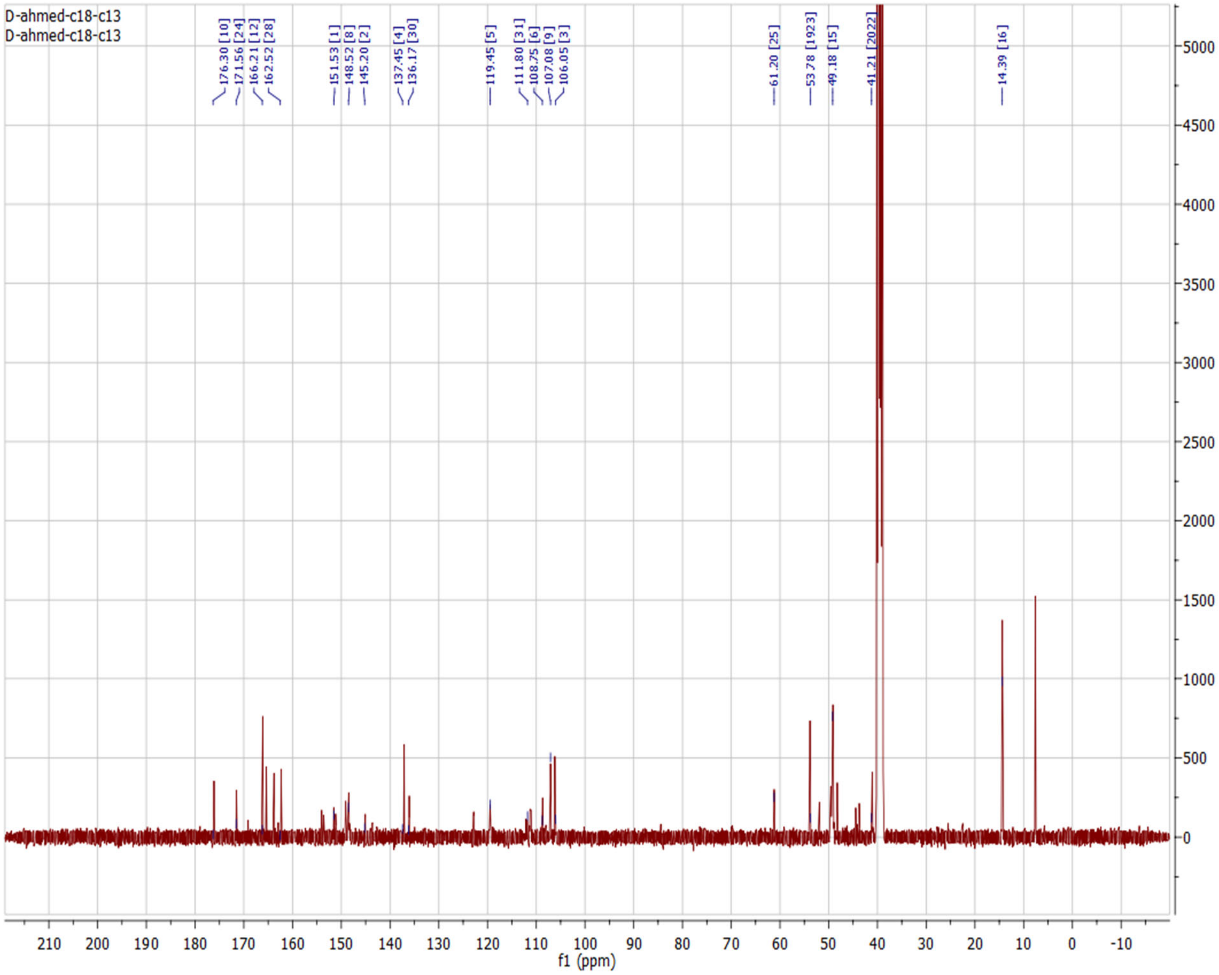

Figure S19b: ^{13}CNMR

Compound 5b

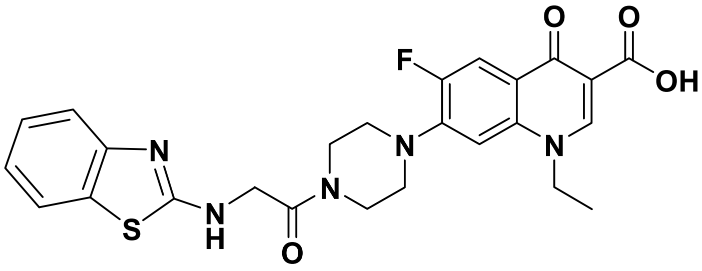

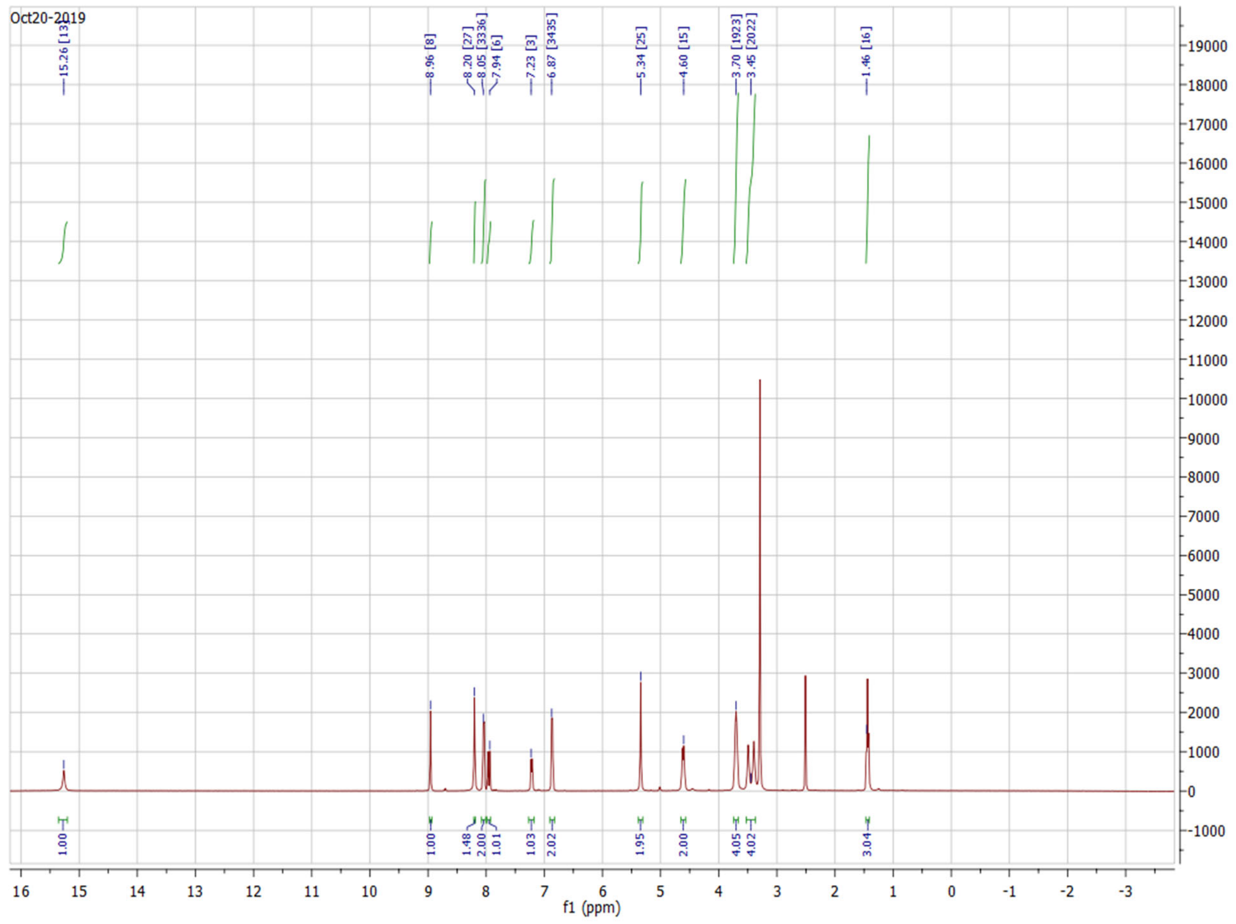

Figure S20a: ^{1}HNMR

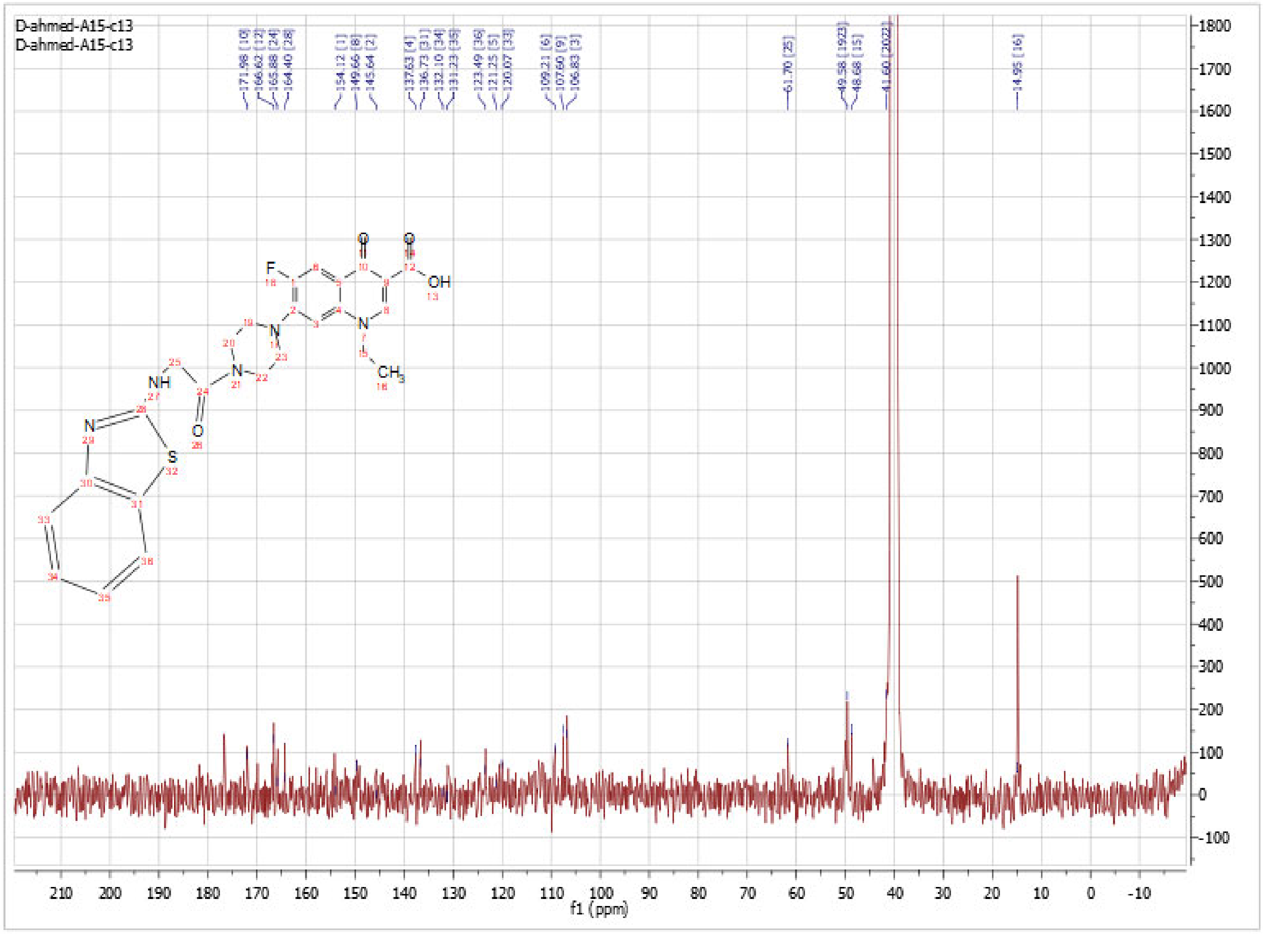

Figure S20b: ^{13}CNMR

Compound 4j

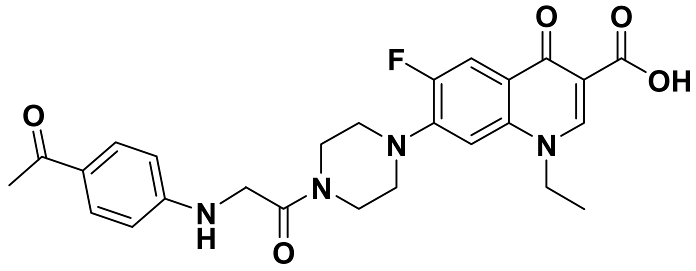

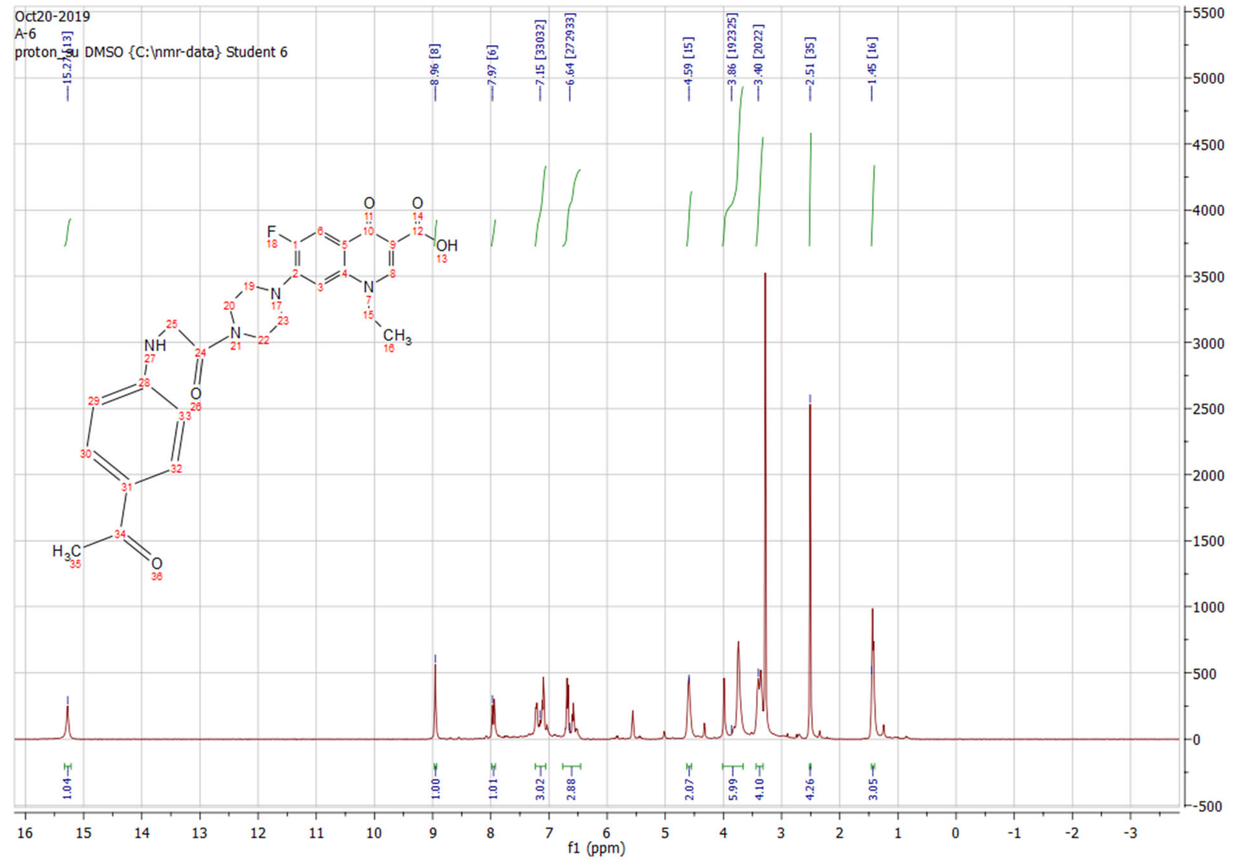

Figure S21a: ^{1}HNMR

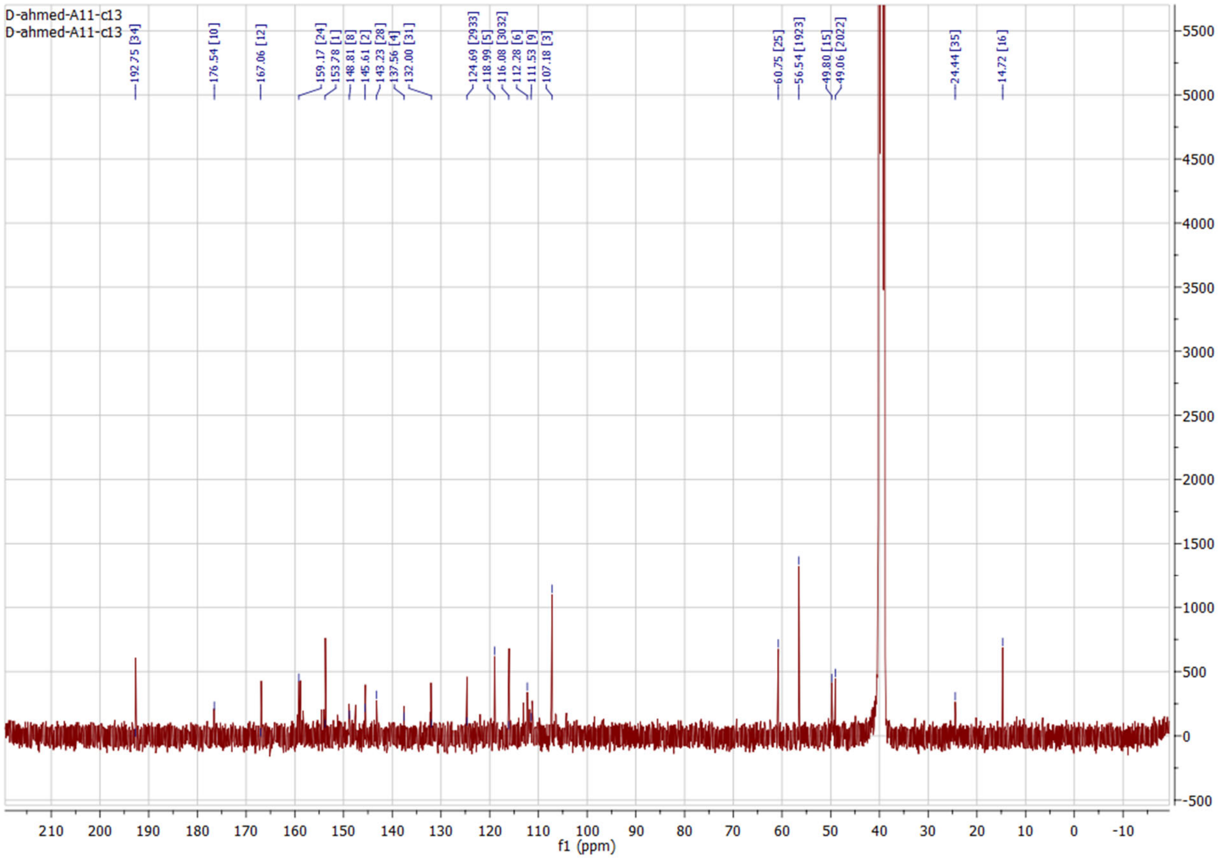

Figure S21b: ^{13}CNMR

Compound 4i

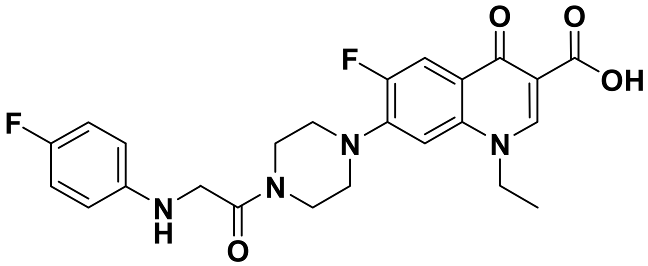

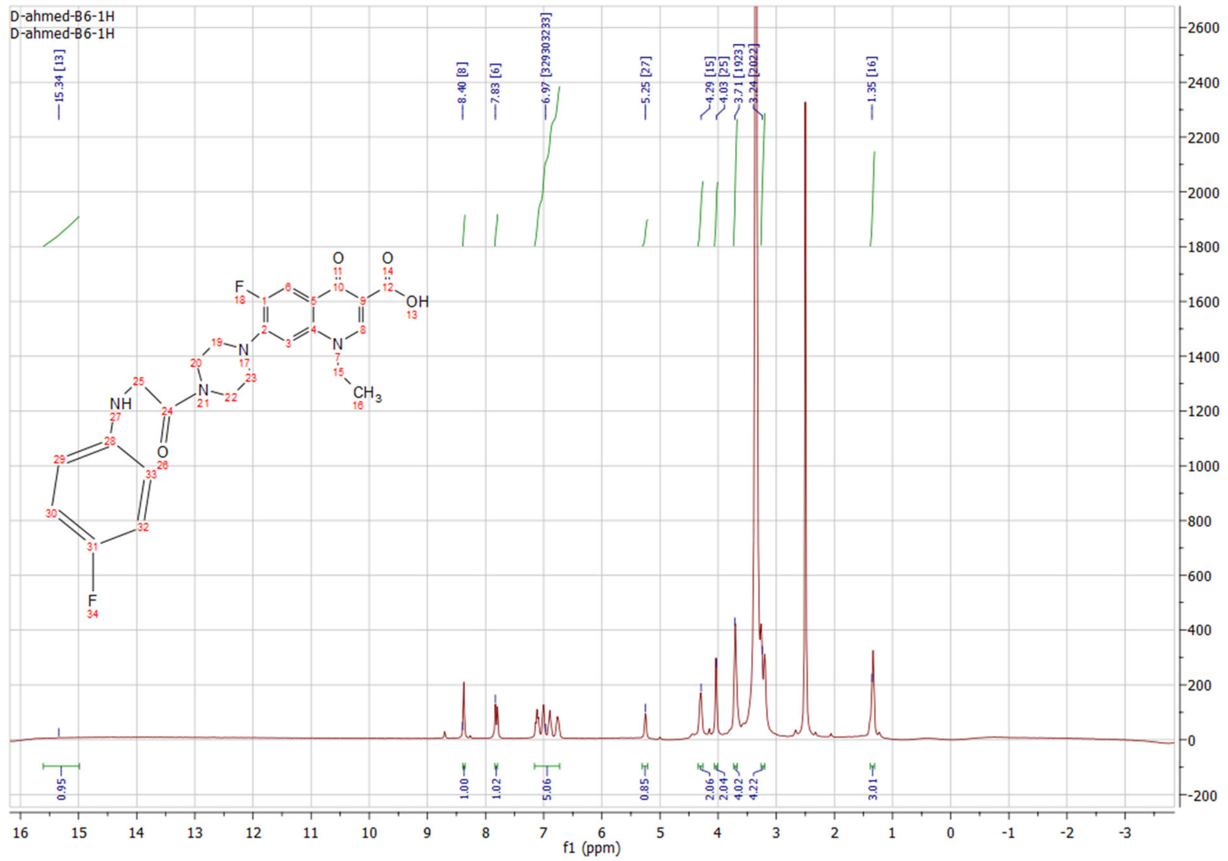

Figure S22a: ^{1}HNMR

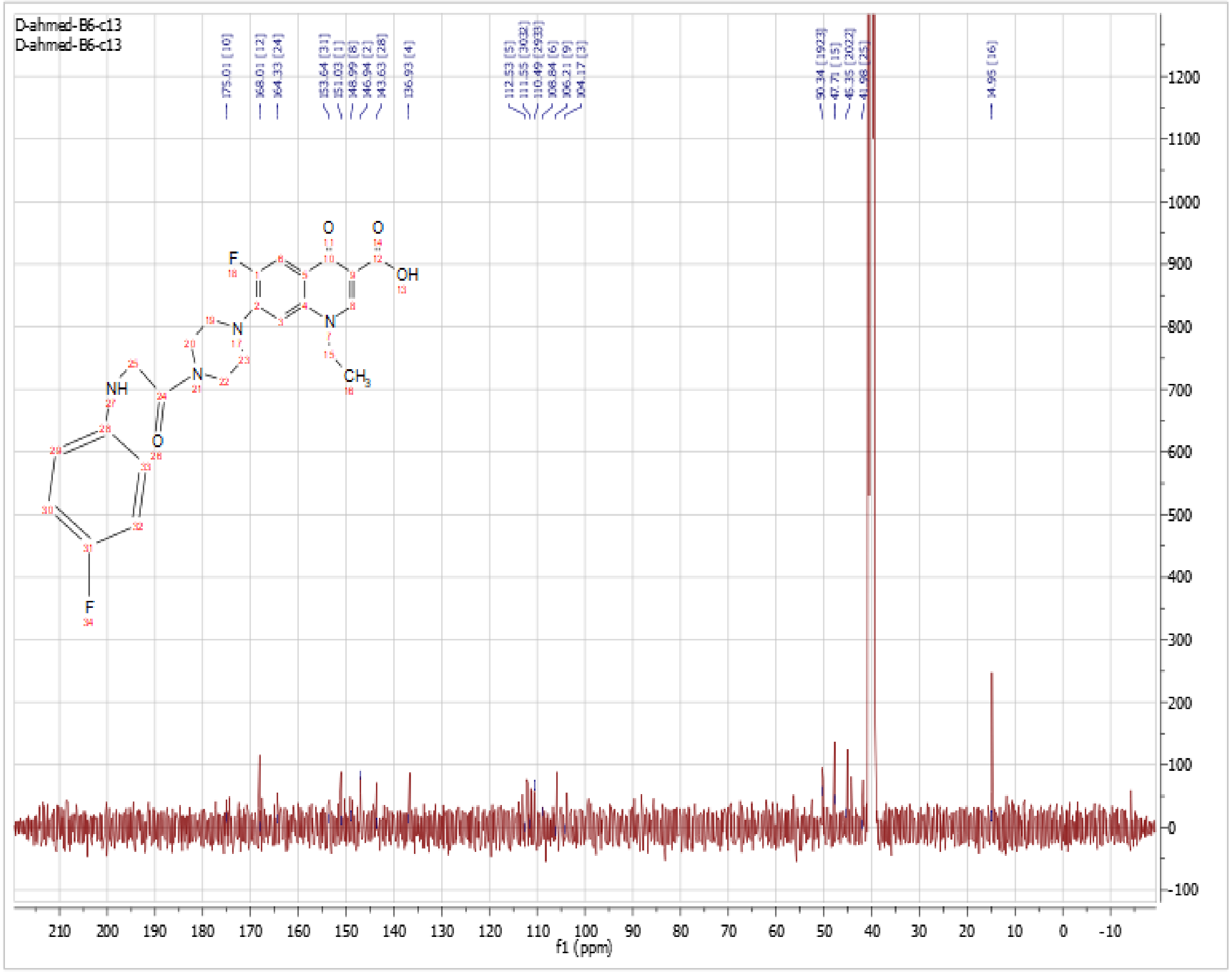

Figure S22b: ^{13}CNMR

Compound 8b

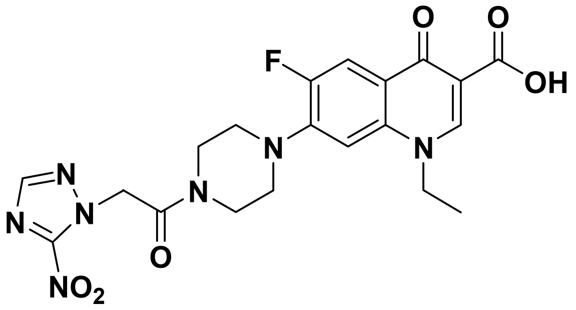

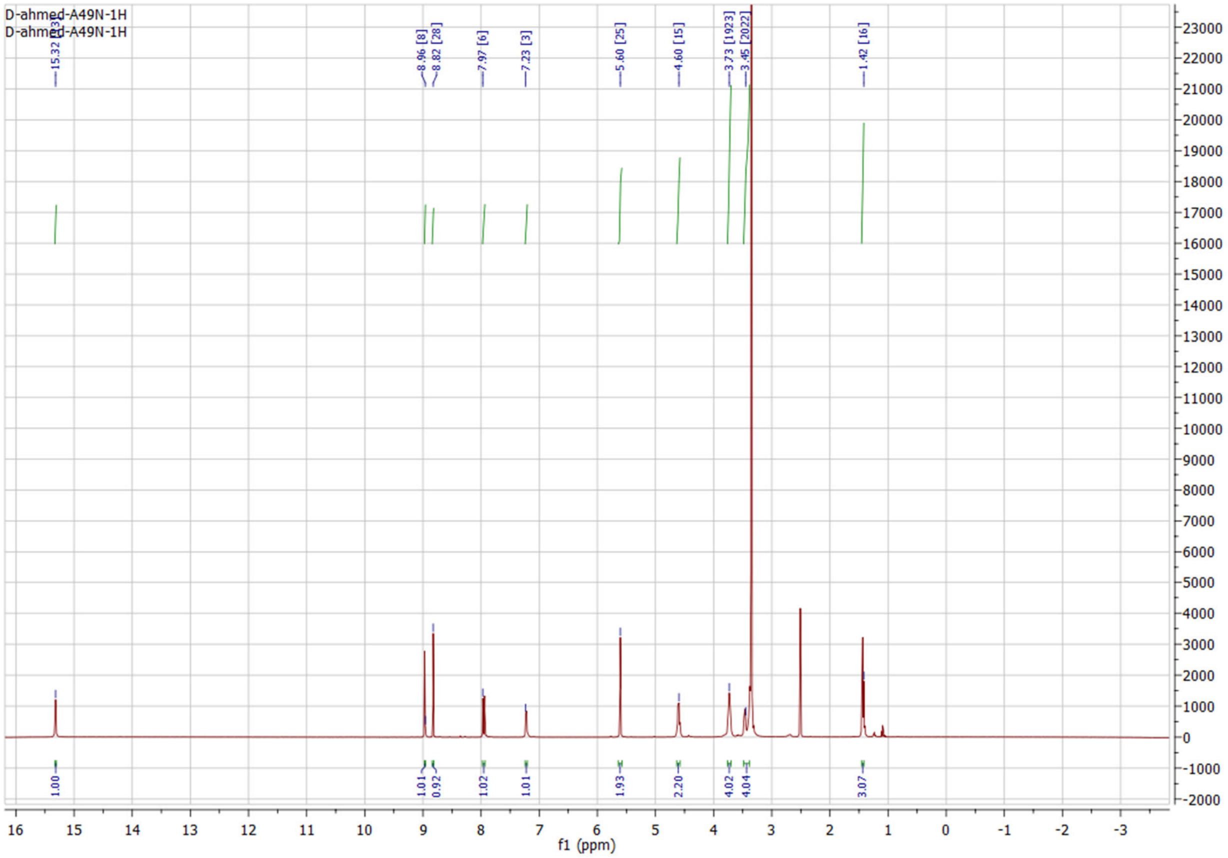

Figure S23a: ^{1}HNMR

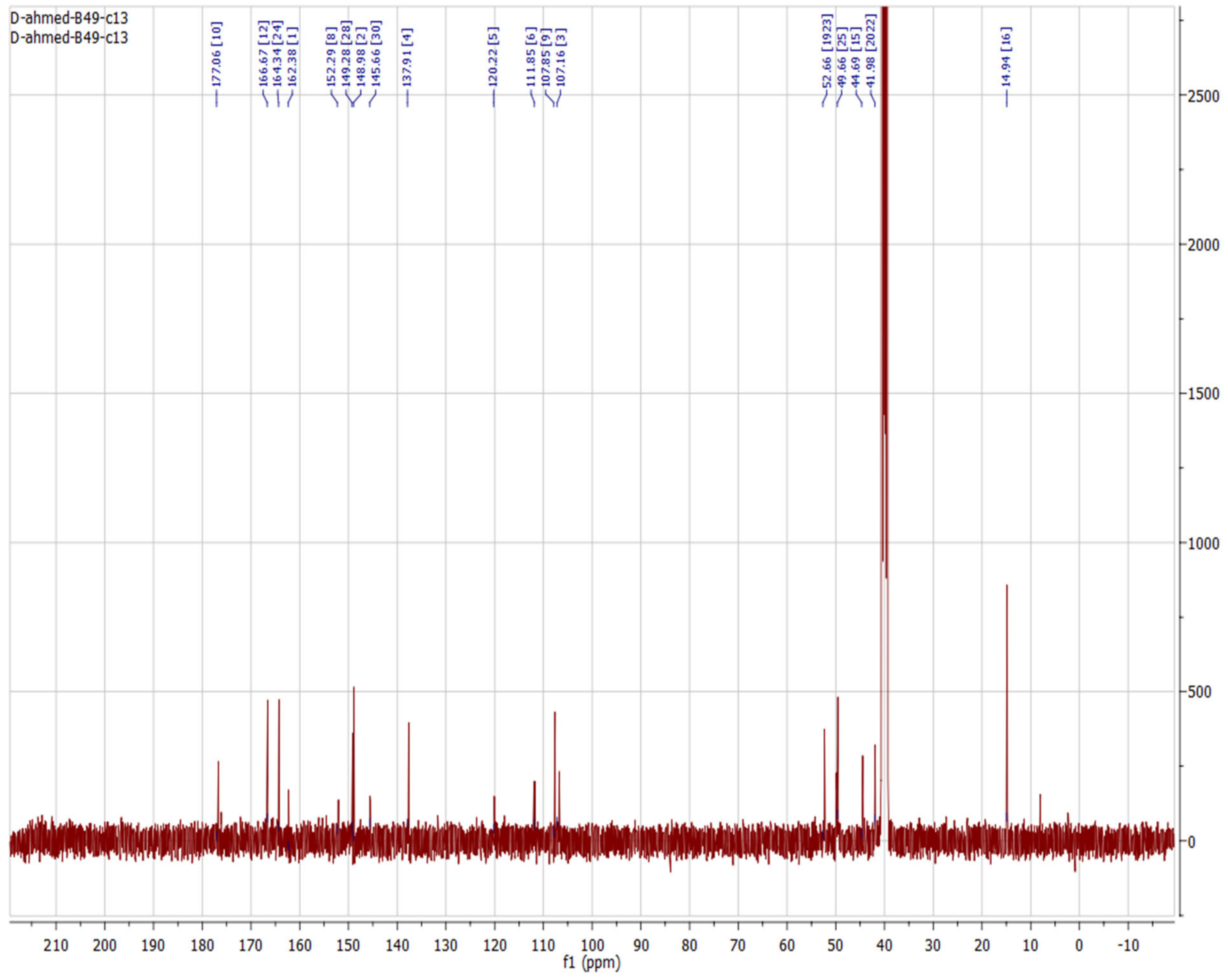

Figure S23b: ^{13}CNMR

Compound 14

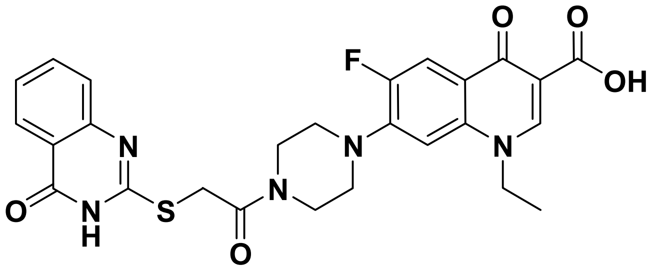

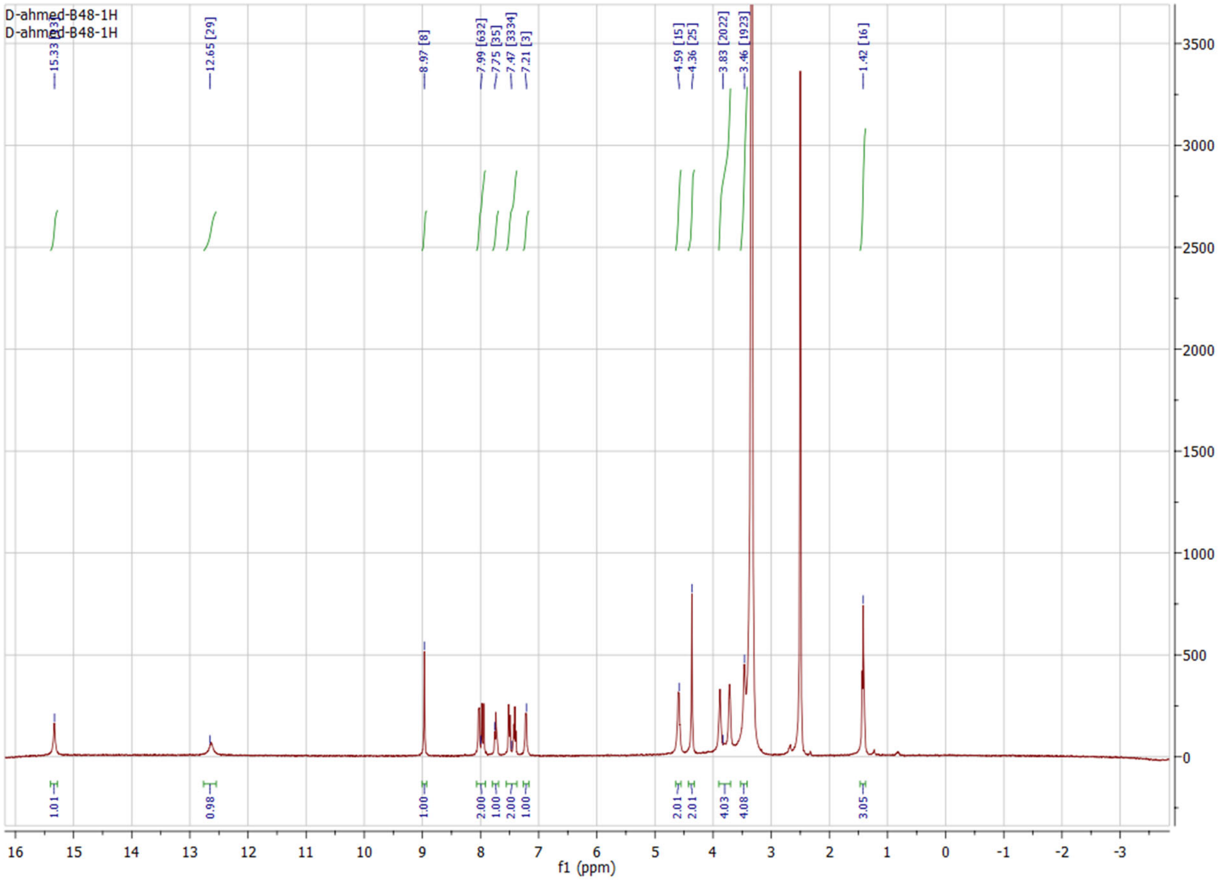

Figure S24a: [1]HMNR

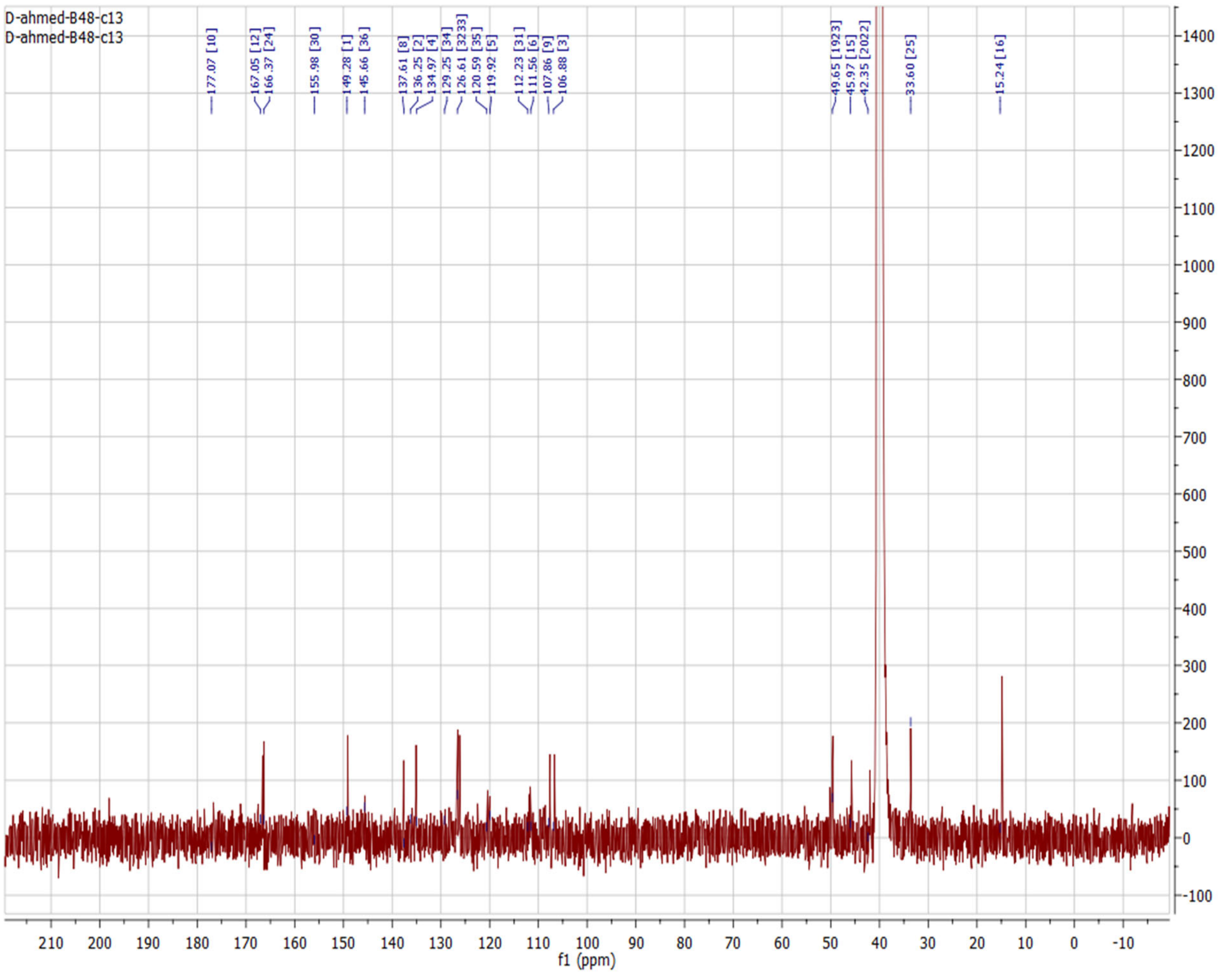

Figure S24b: ^{13}CNMR

Compound 20a

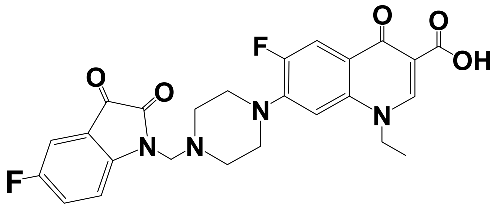

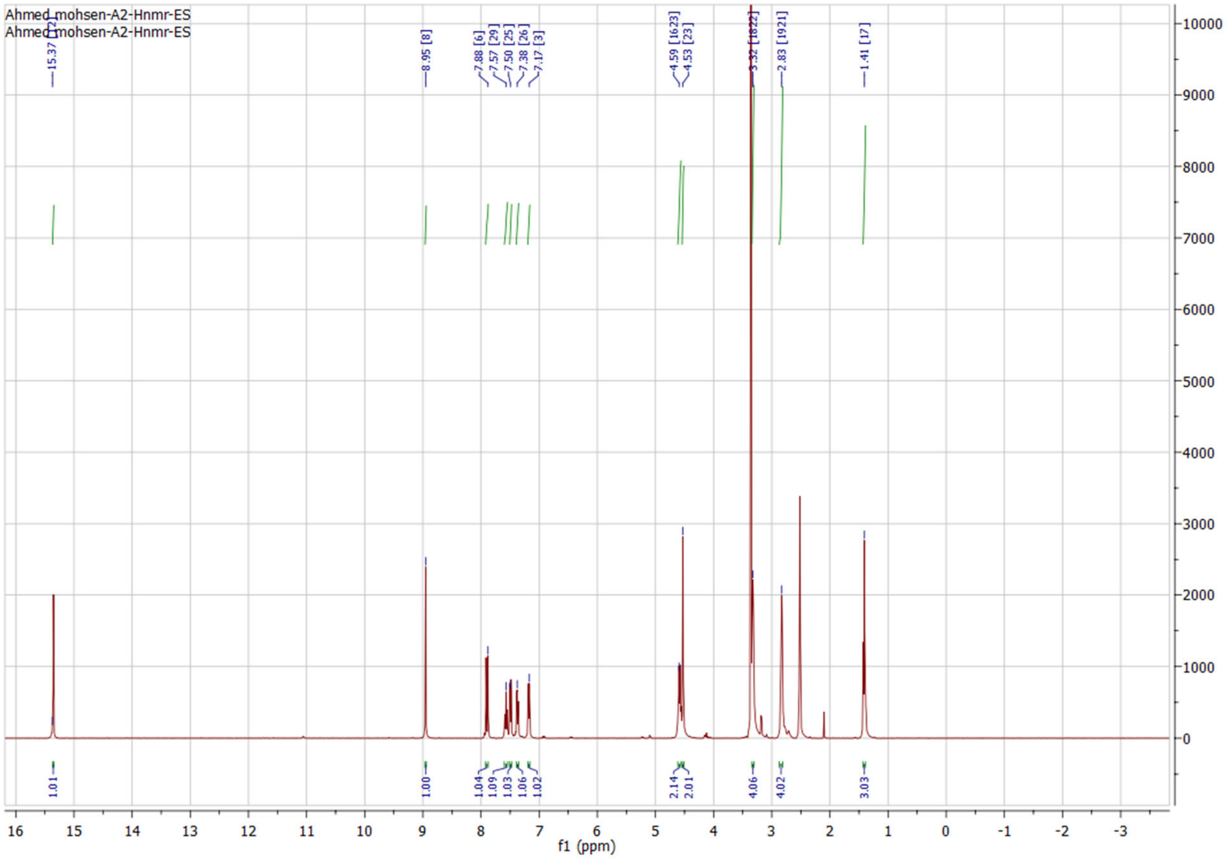

Figure S25a: ^{1}HNMR

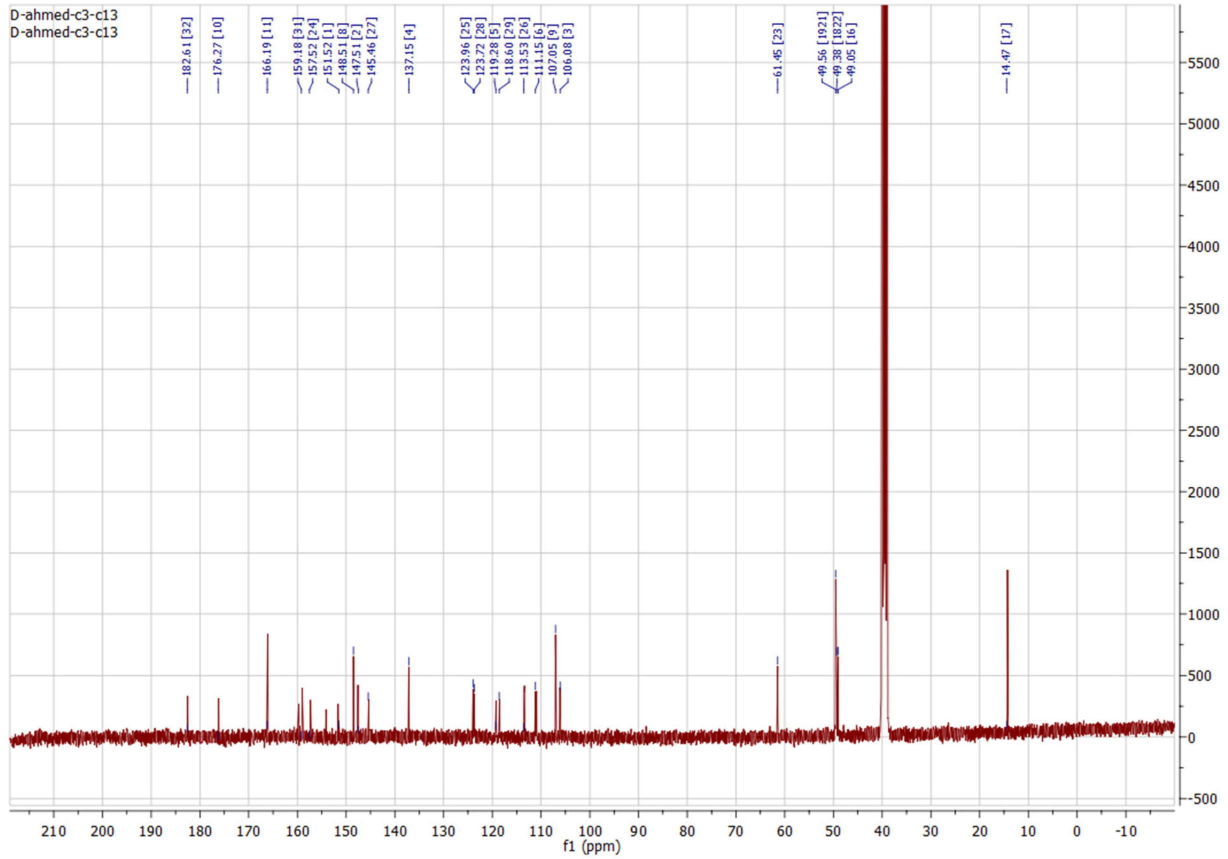

Figure S25b: ^{13}CNMR

Compound 8a

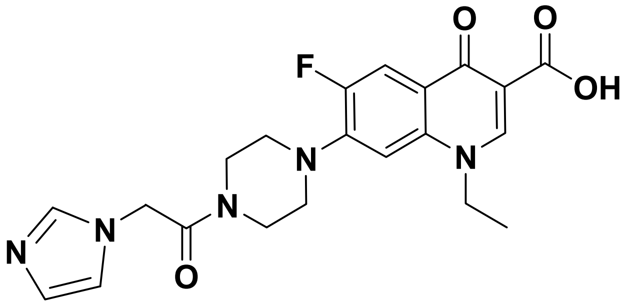

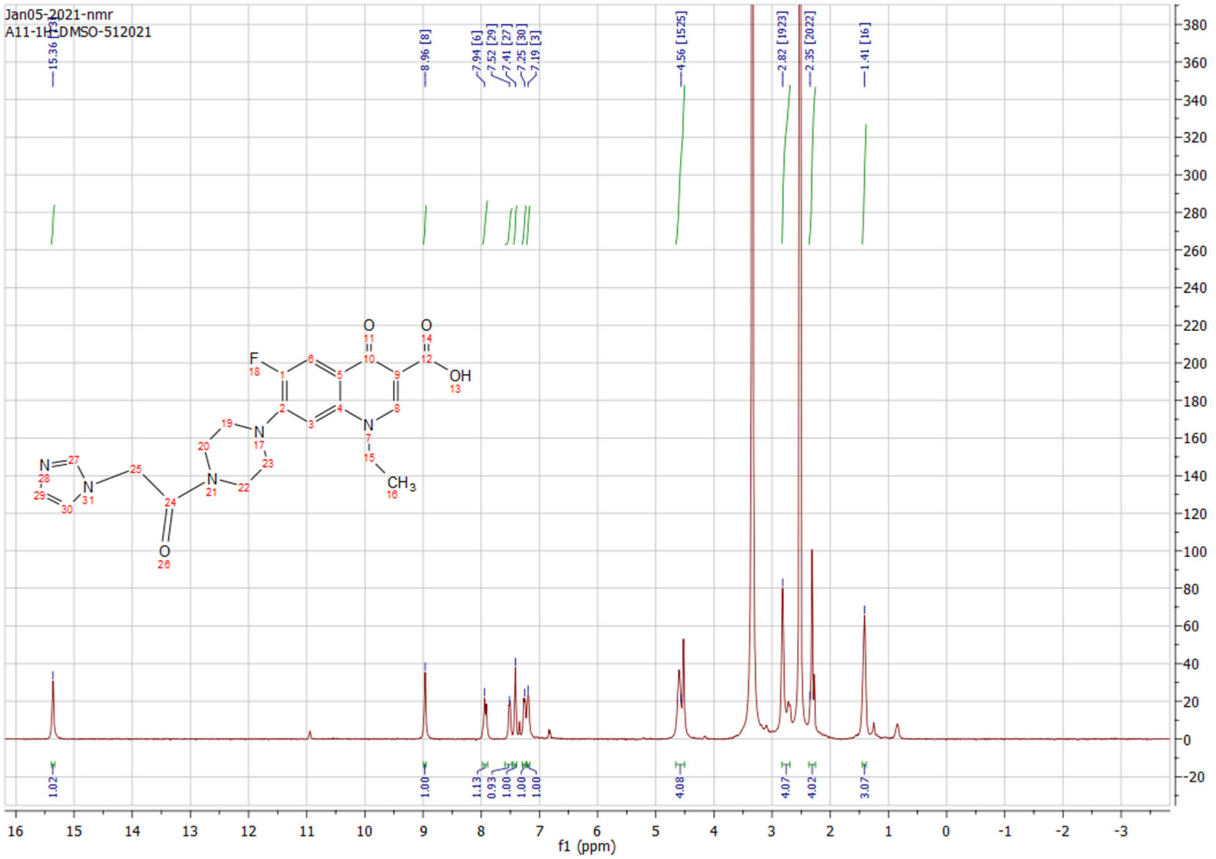

Figure S26a: ^{1}HNMR

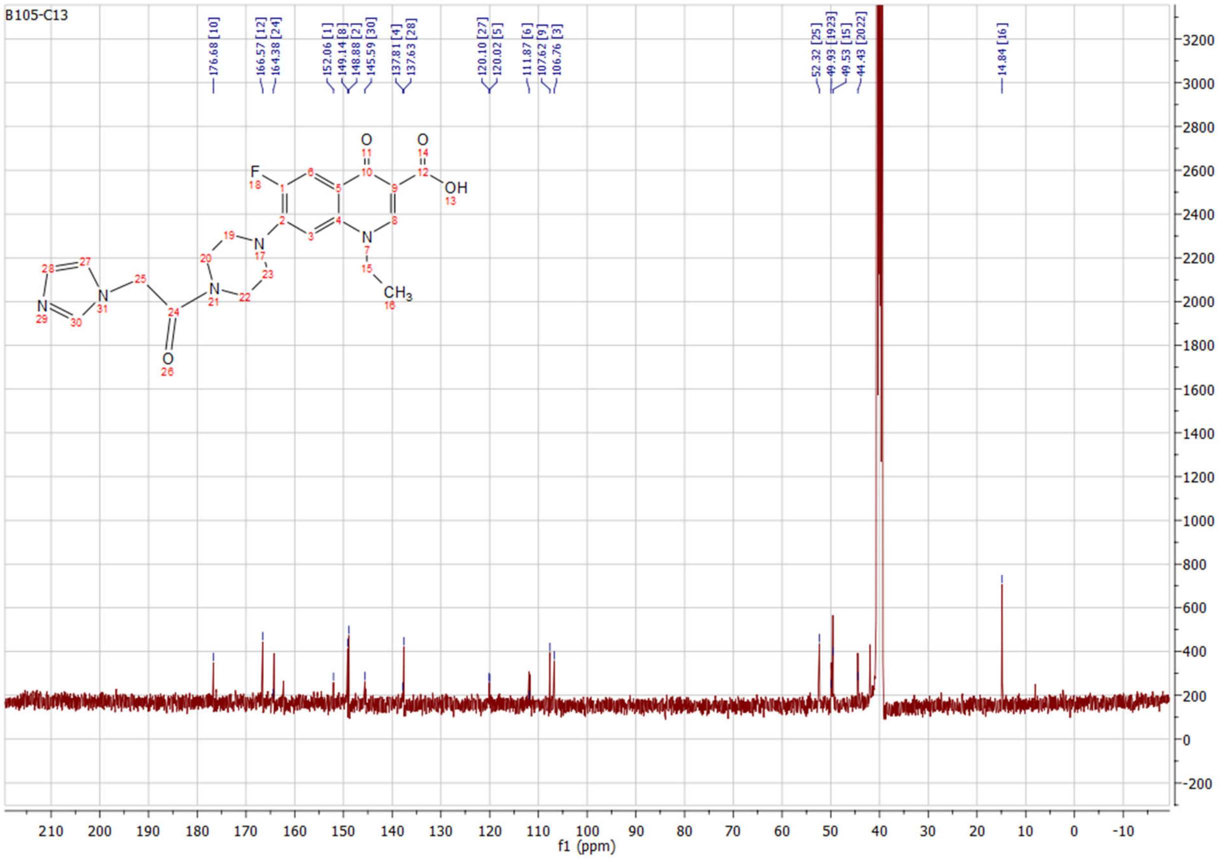

Figure S26b: ^{13}CNMR

Compound 3c

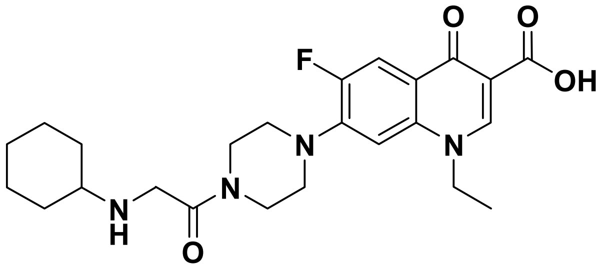

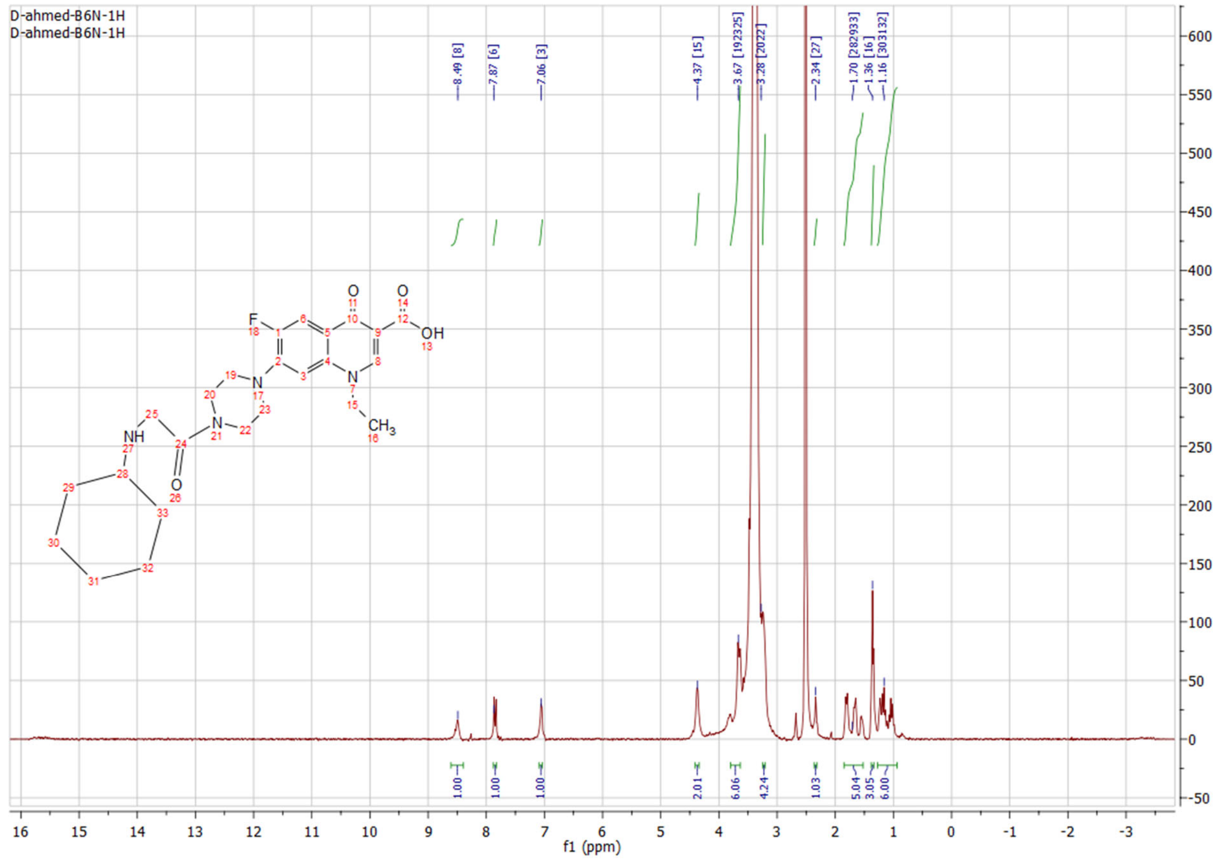

Figure S27a: ^{1}HNMR

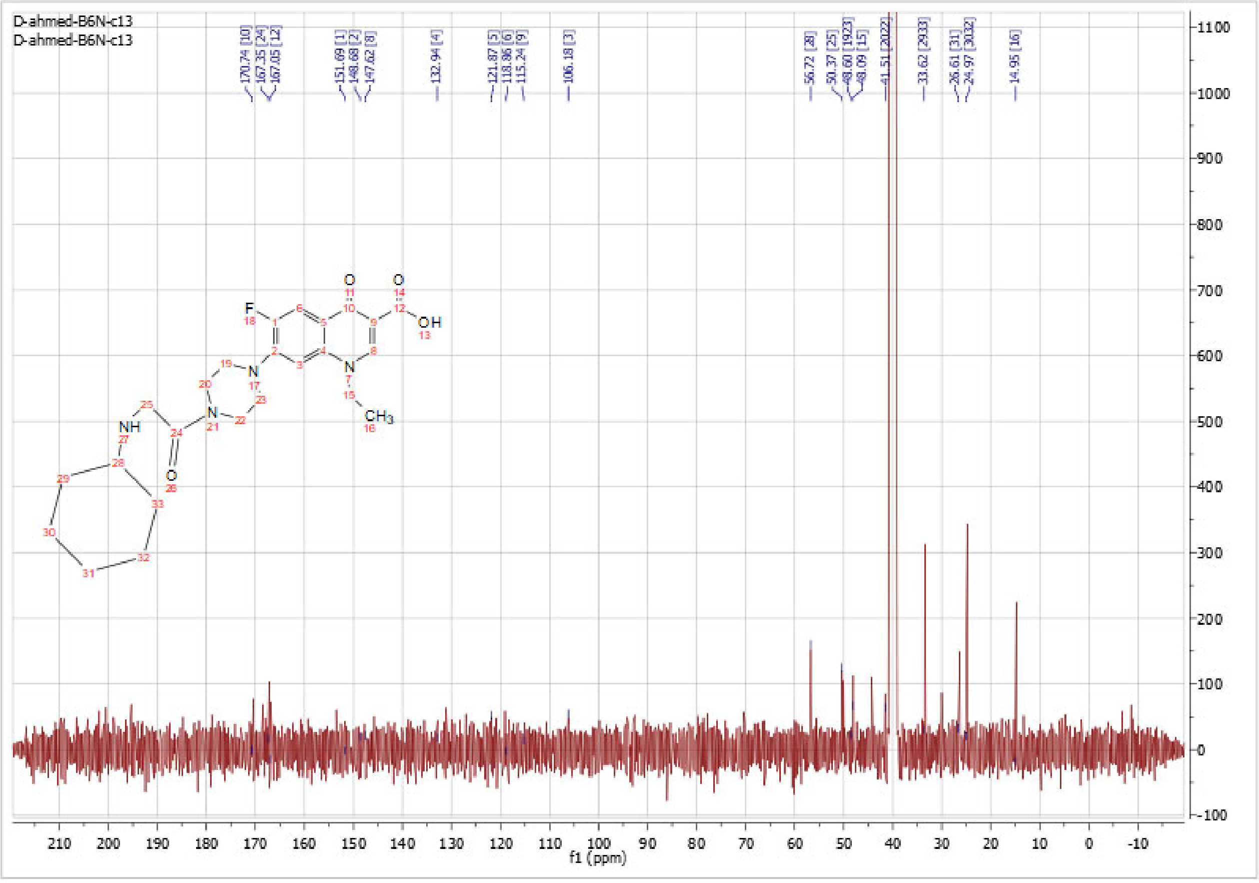

Figure S27b: ^{13}CNMR

Compound 20b

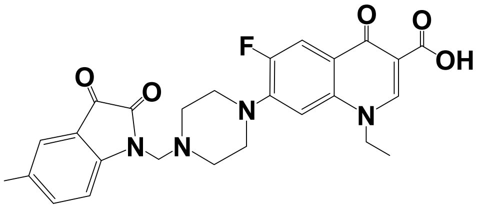

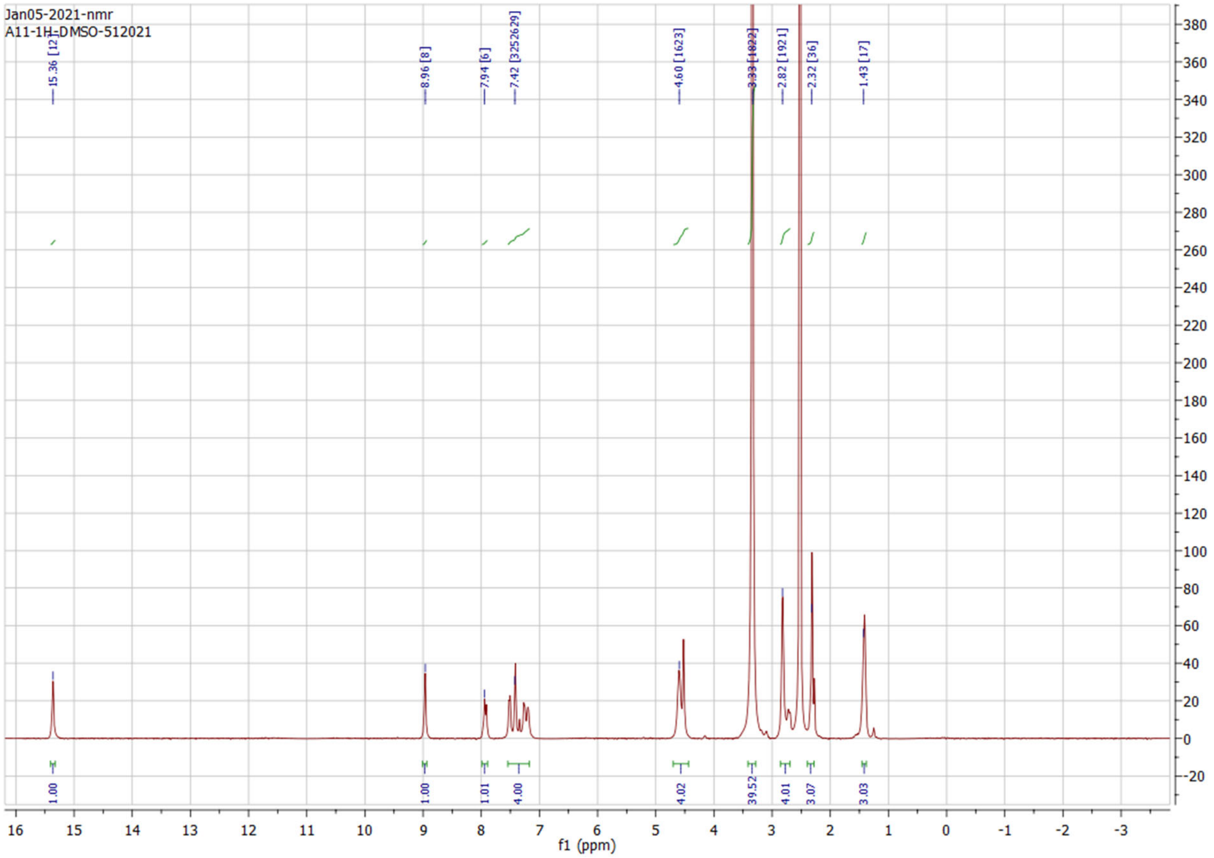

Figure S28a: ^{1}HNMR

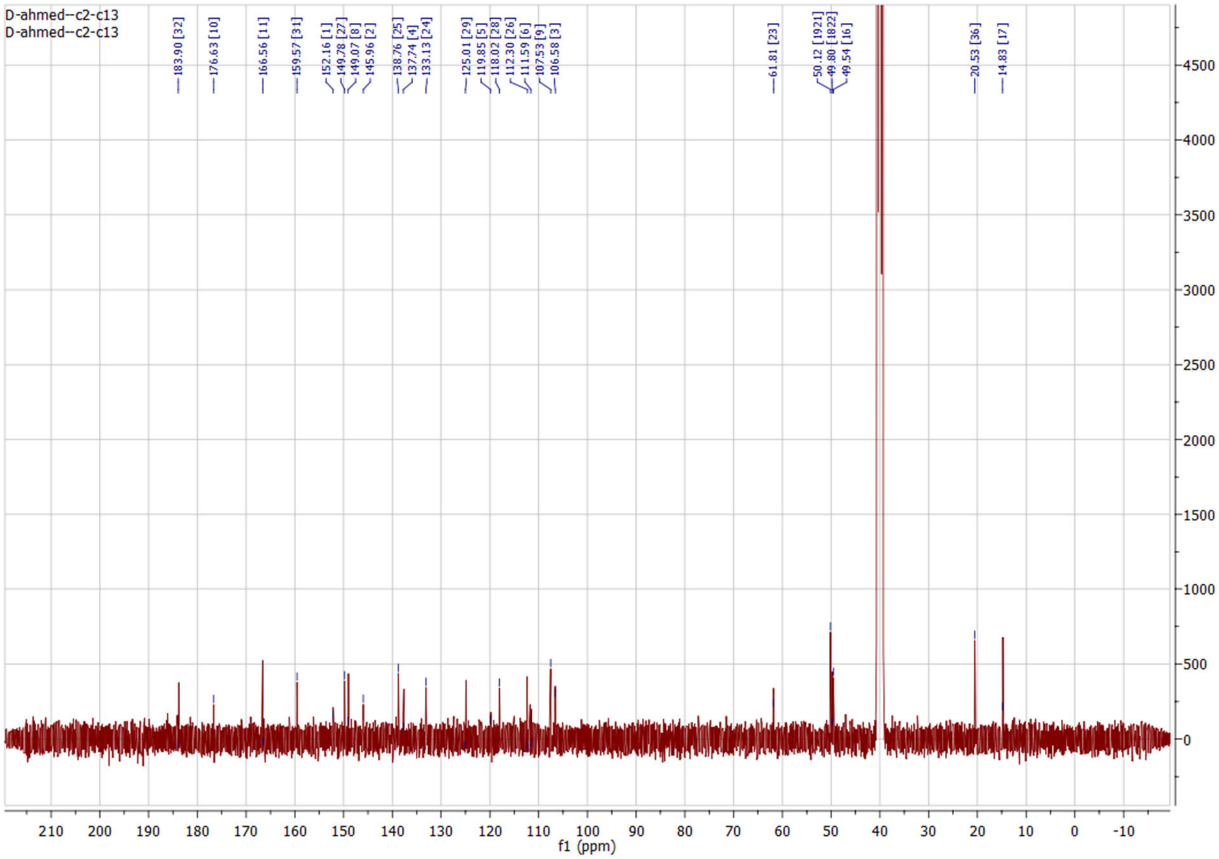

Figure S28b: ^{13}CNMR

Compound 20c

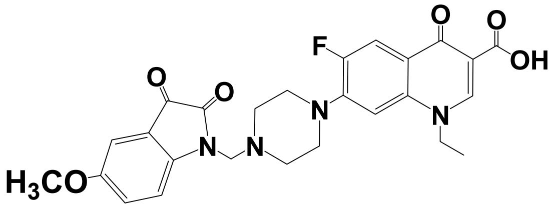

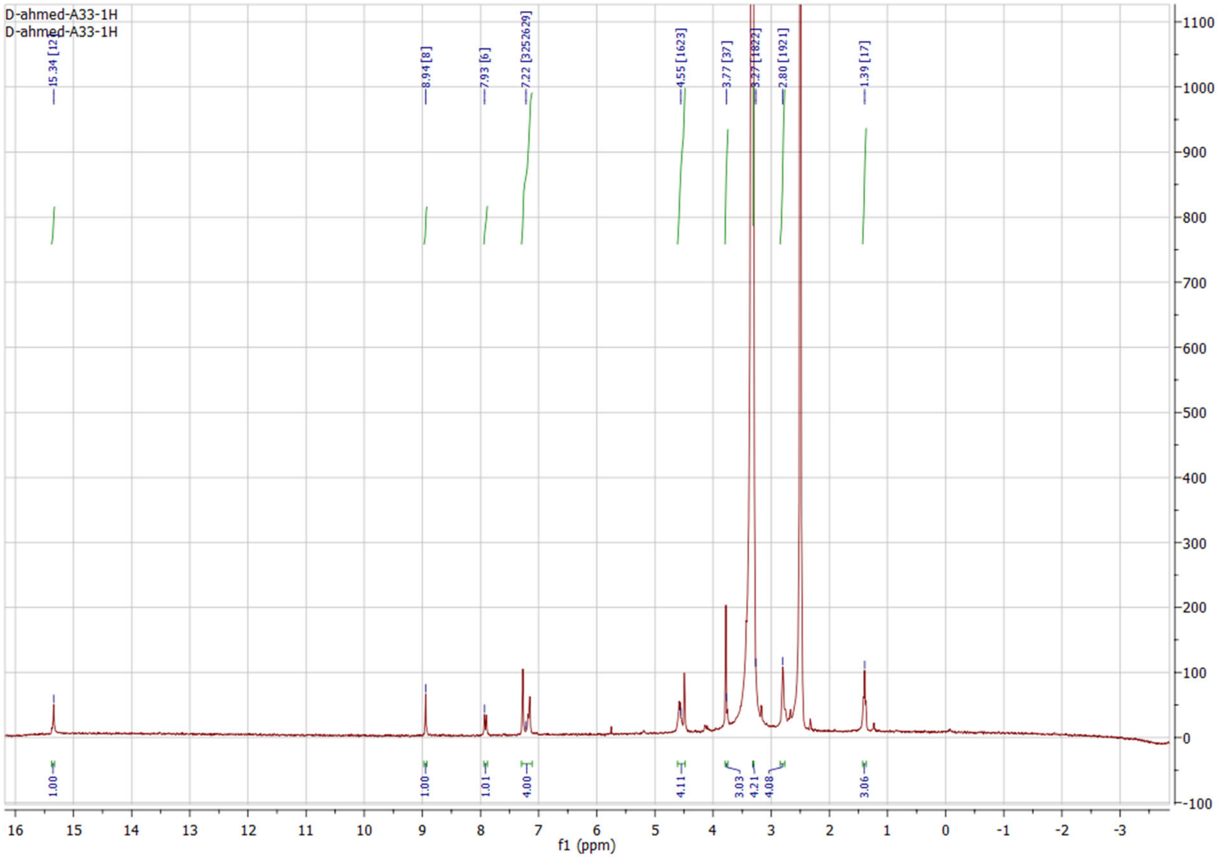

Figure S29a: ^{1}HNMR

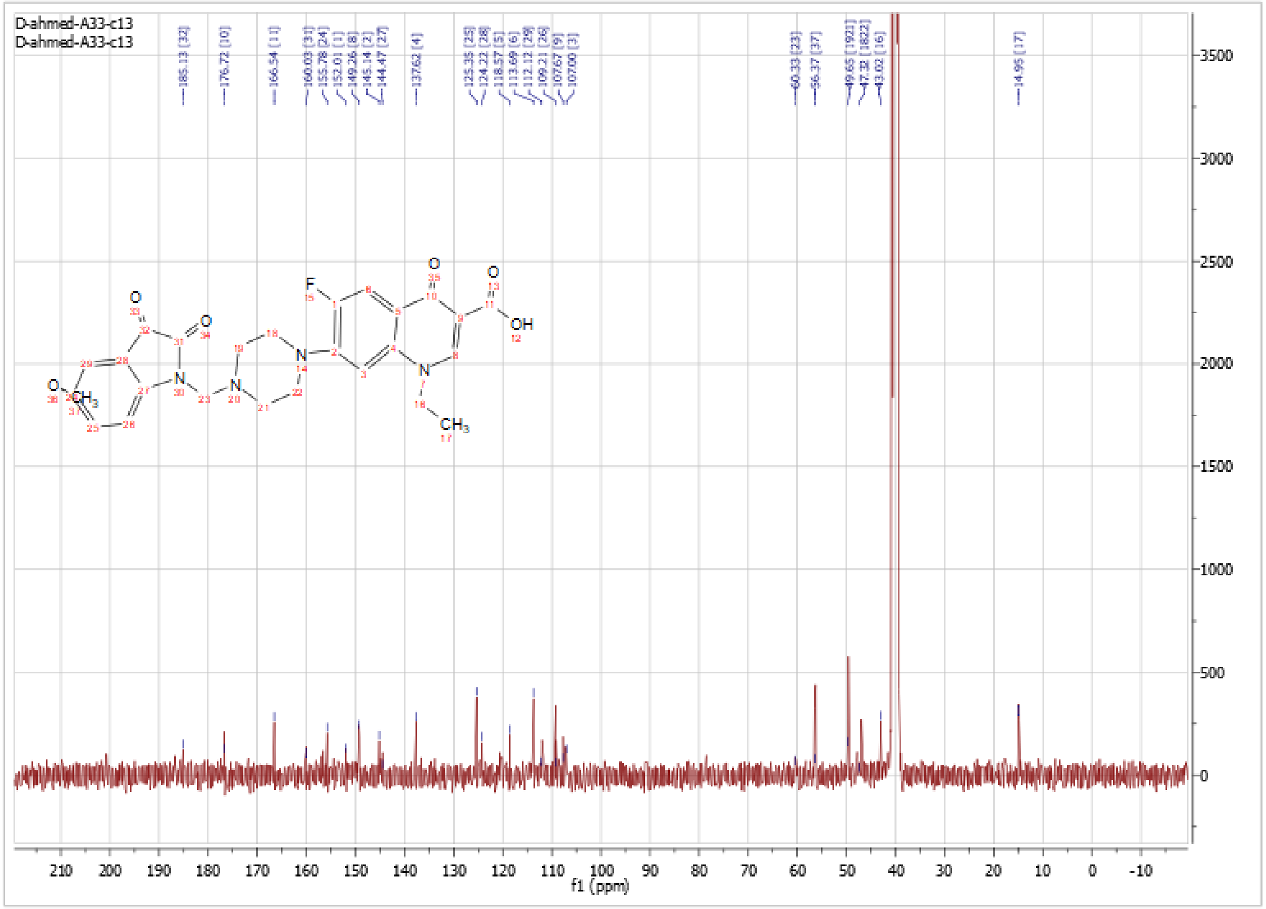

Figure S29b: ^{13}CNMR

Compound 21

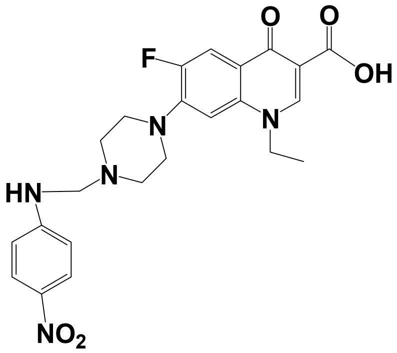

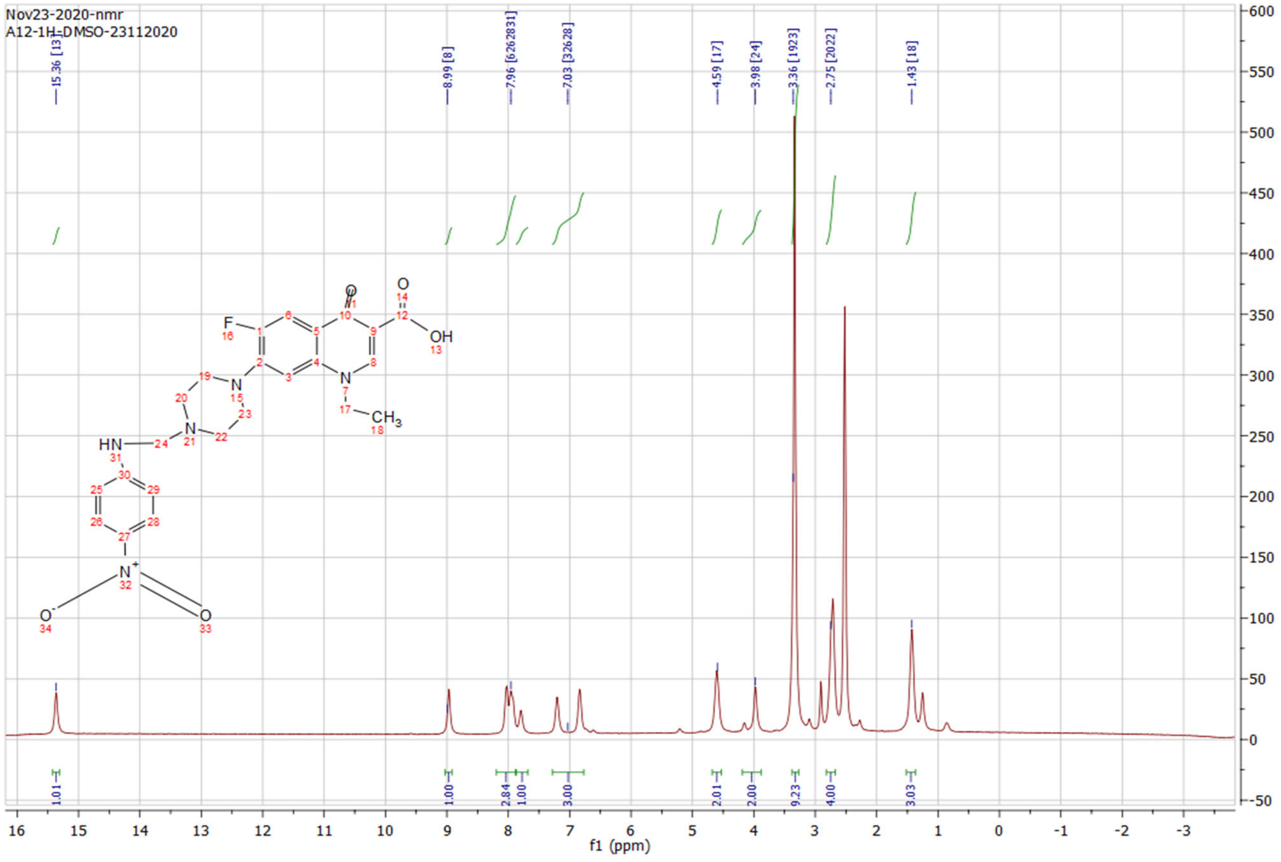

Figure S30a: ^{1}HNMR

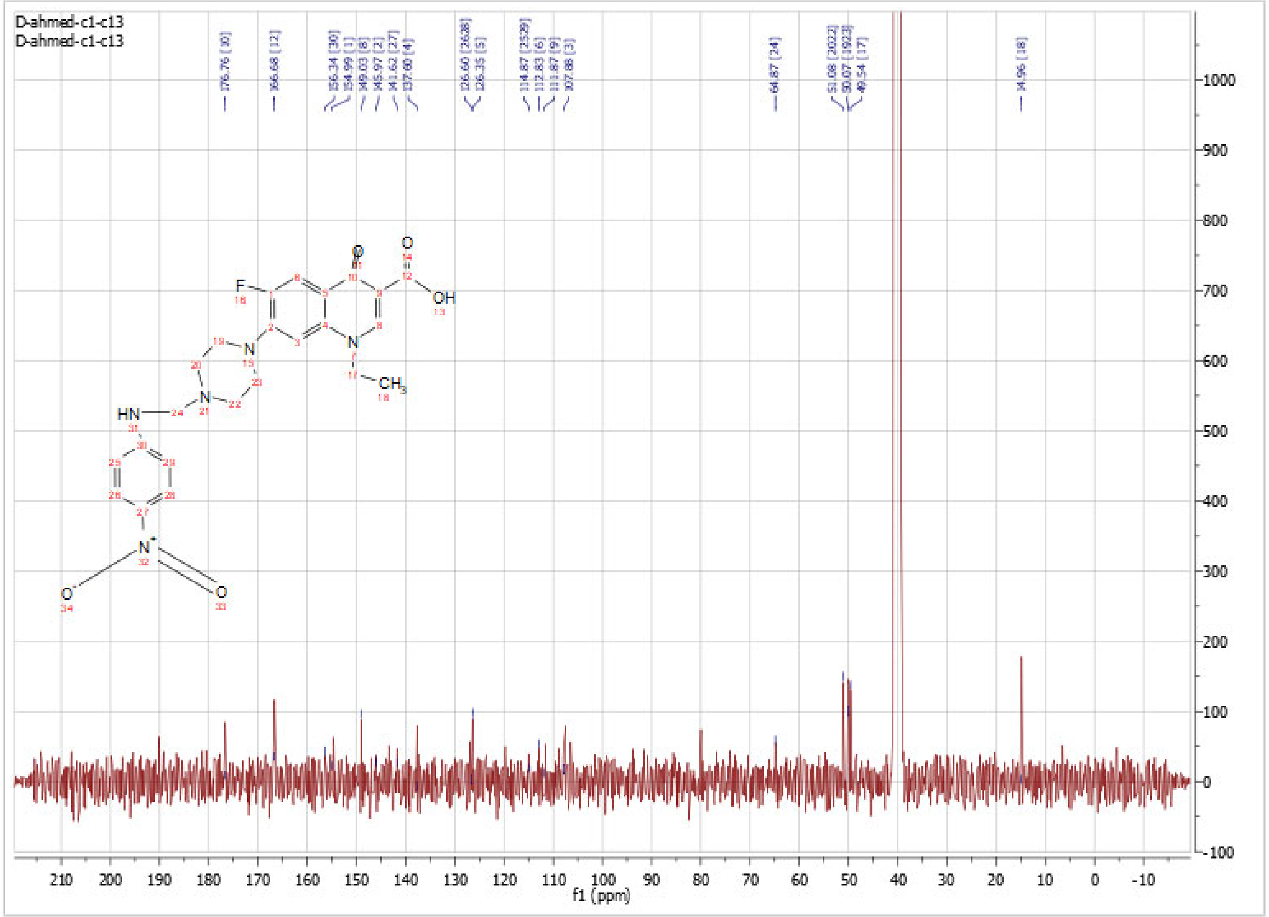

Figure S30b: ^{13}CNMR

Compound 17a

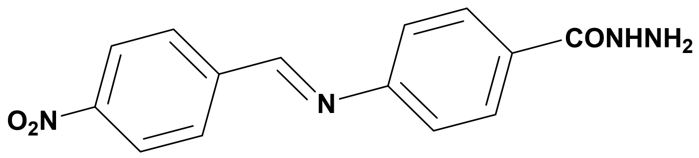

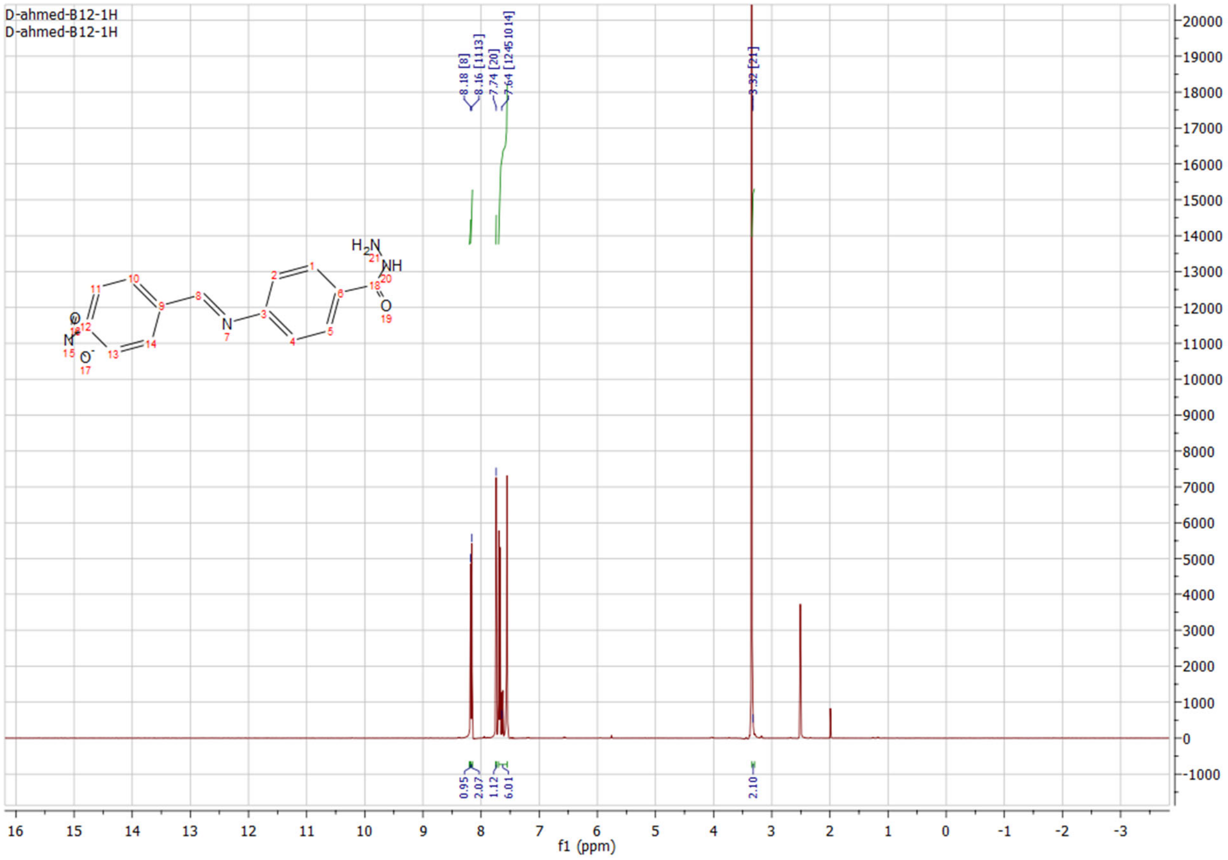

Figure S31a: ^{1}HNMR

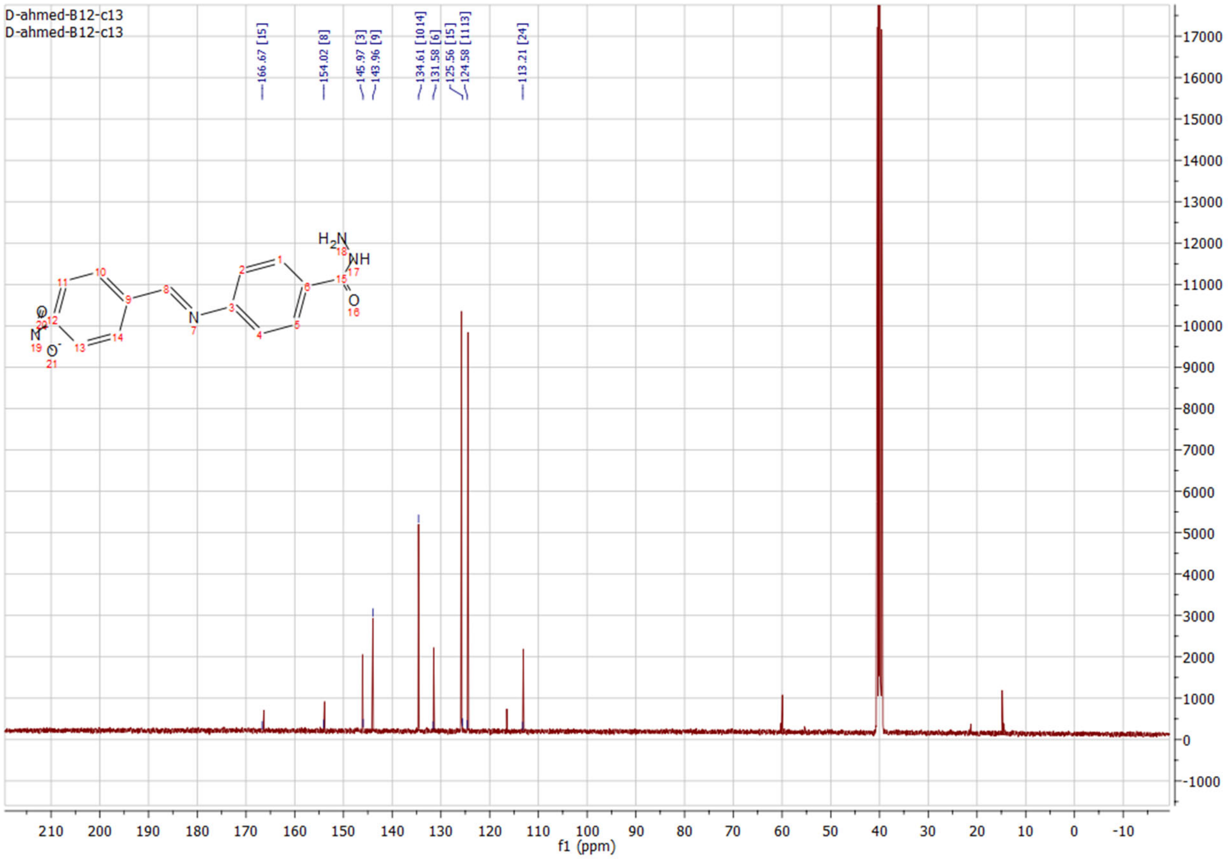

Figure S31b: ^{13}CNMR

Compound 17b

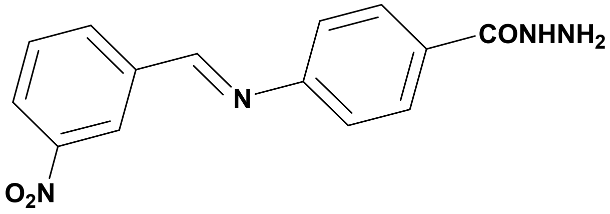

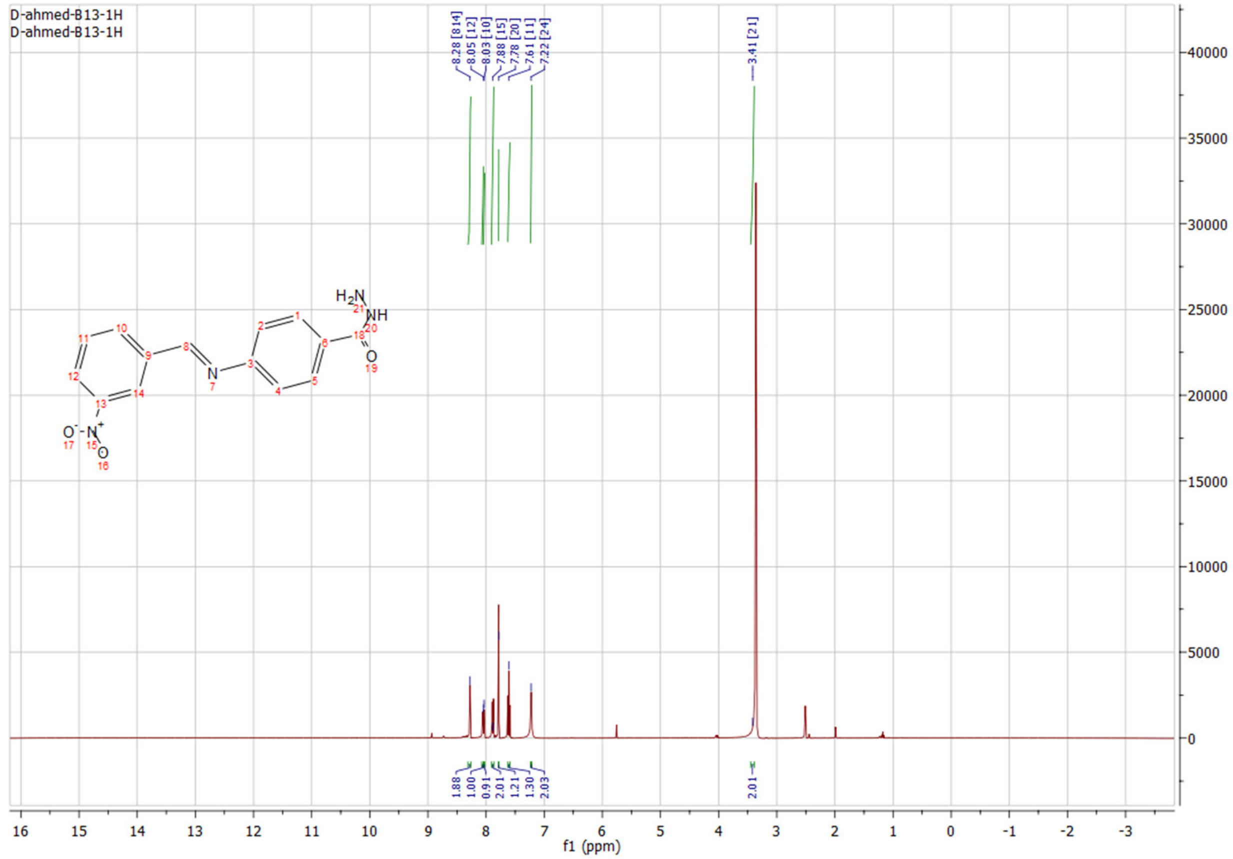

Figure S32a: ^{1}HNMR

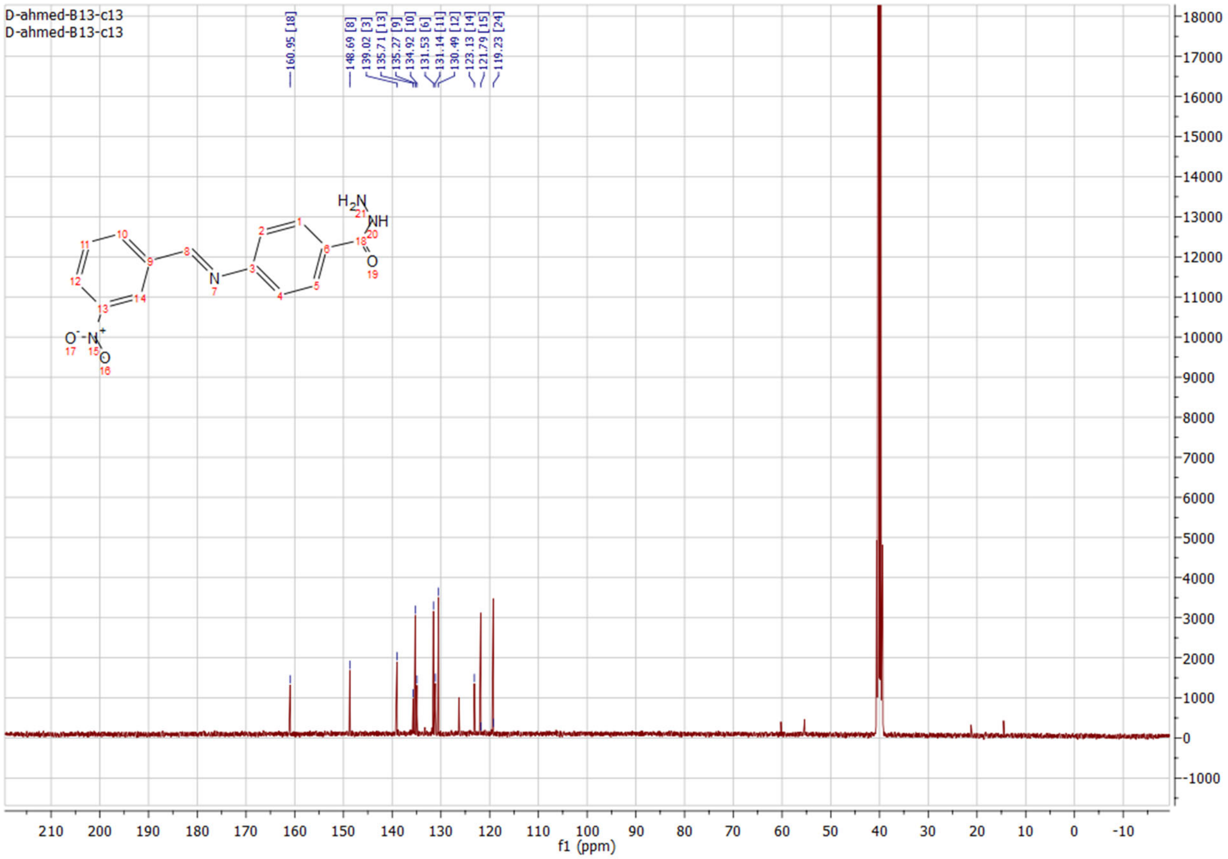

Figure S32b: ^{13}CNMR

Compound 17c

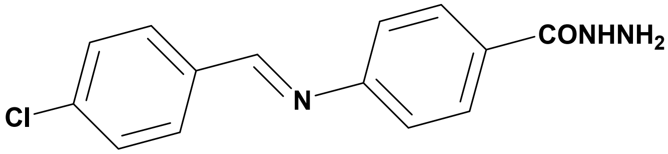

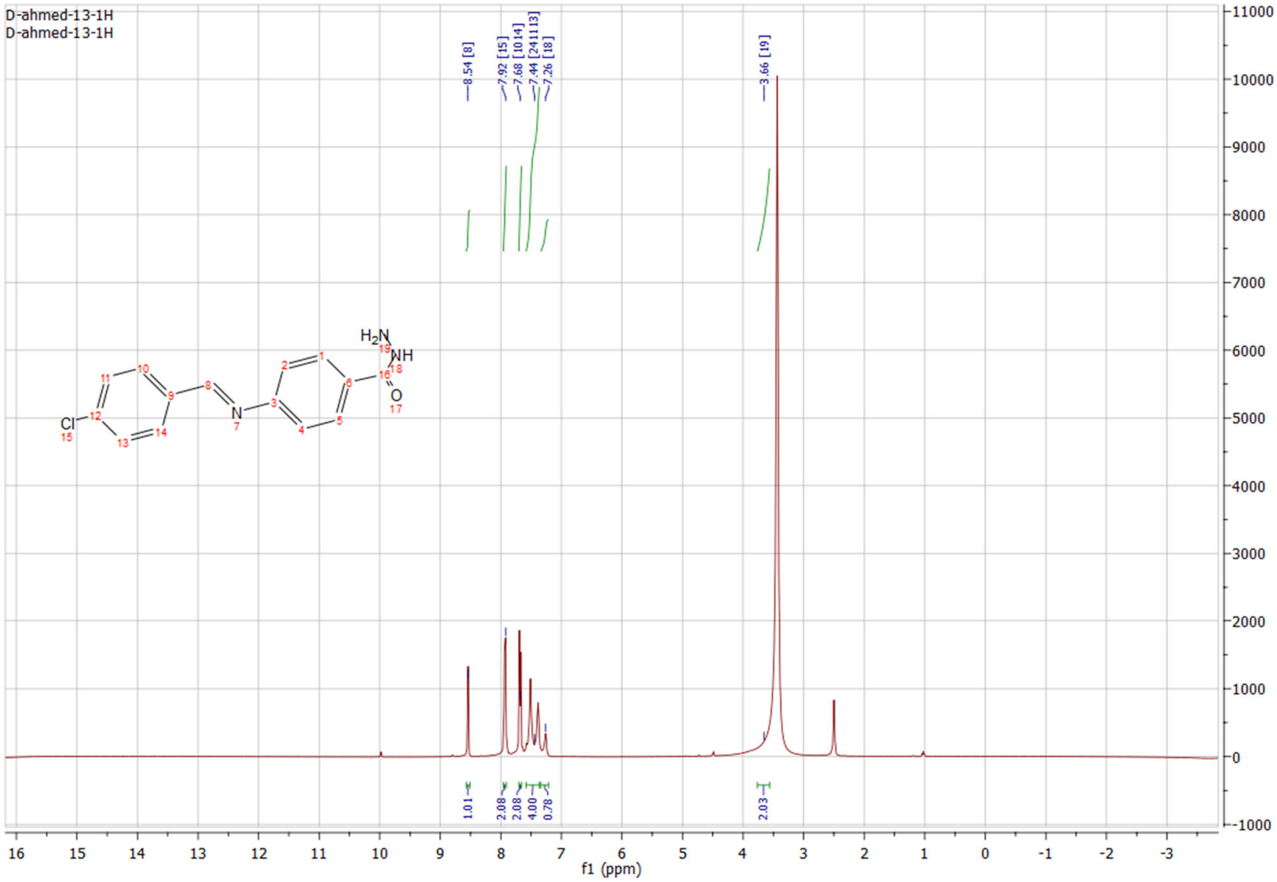

Figure S33a: ^{1}HNMR

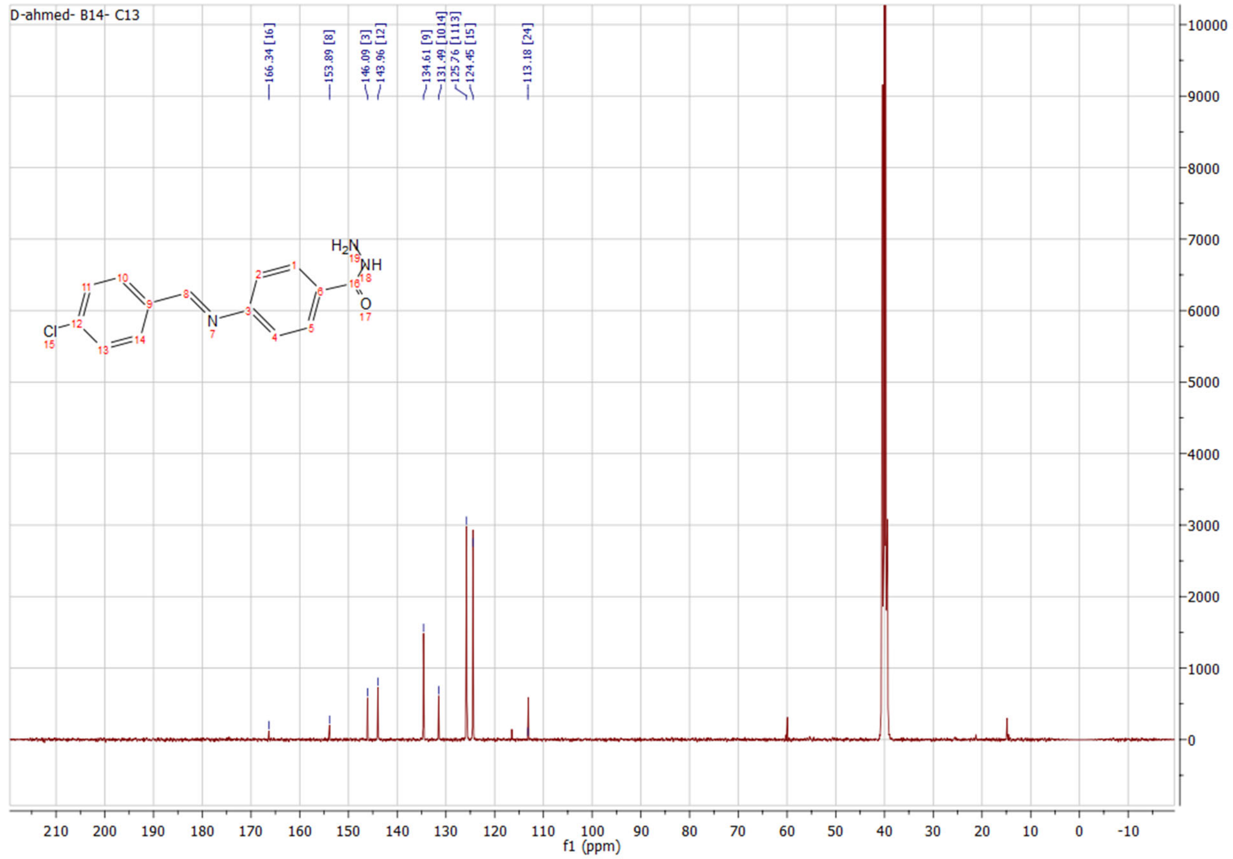

Figure S33b: ^{13}CNMR

Compound 9a

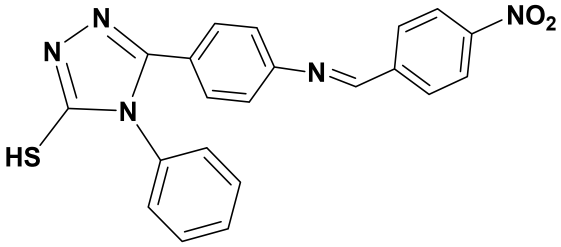

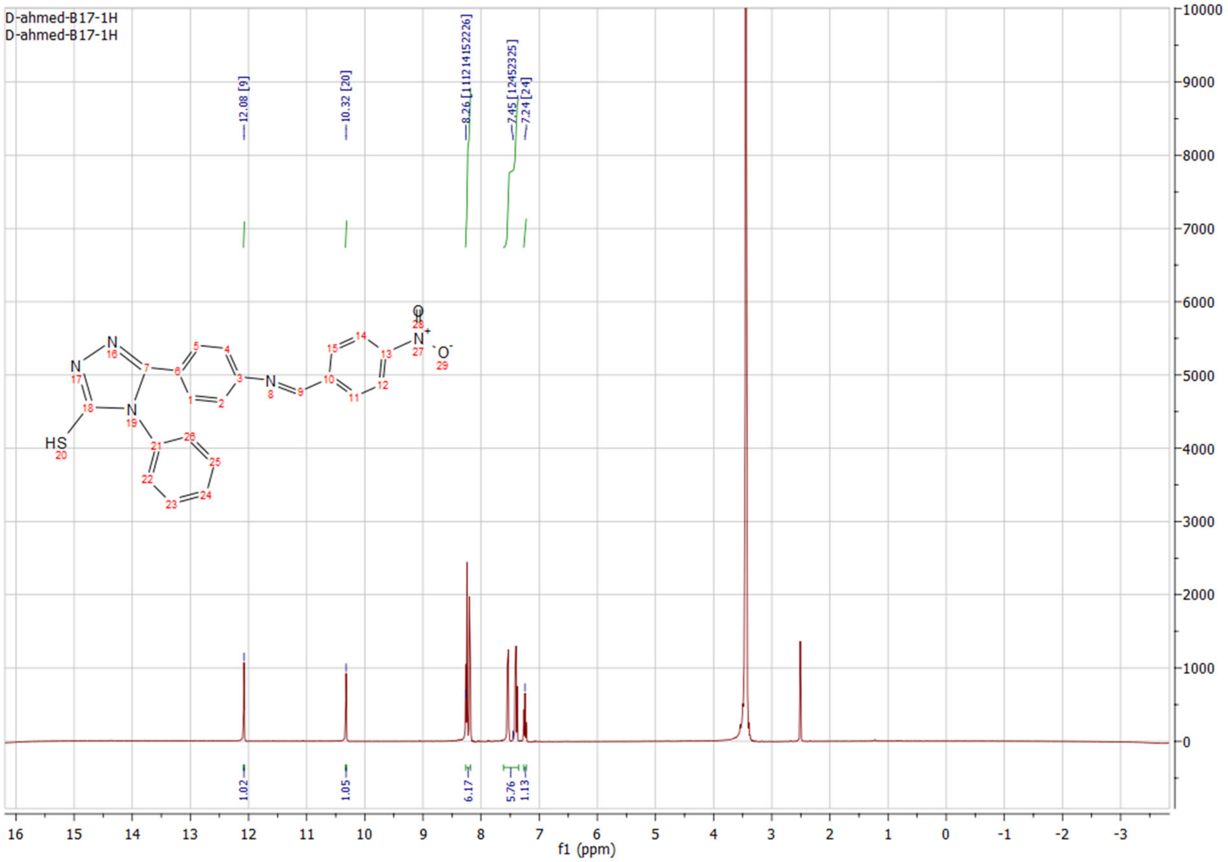

Figure S34a: ^{1}HNMR

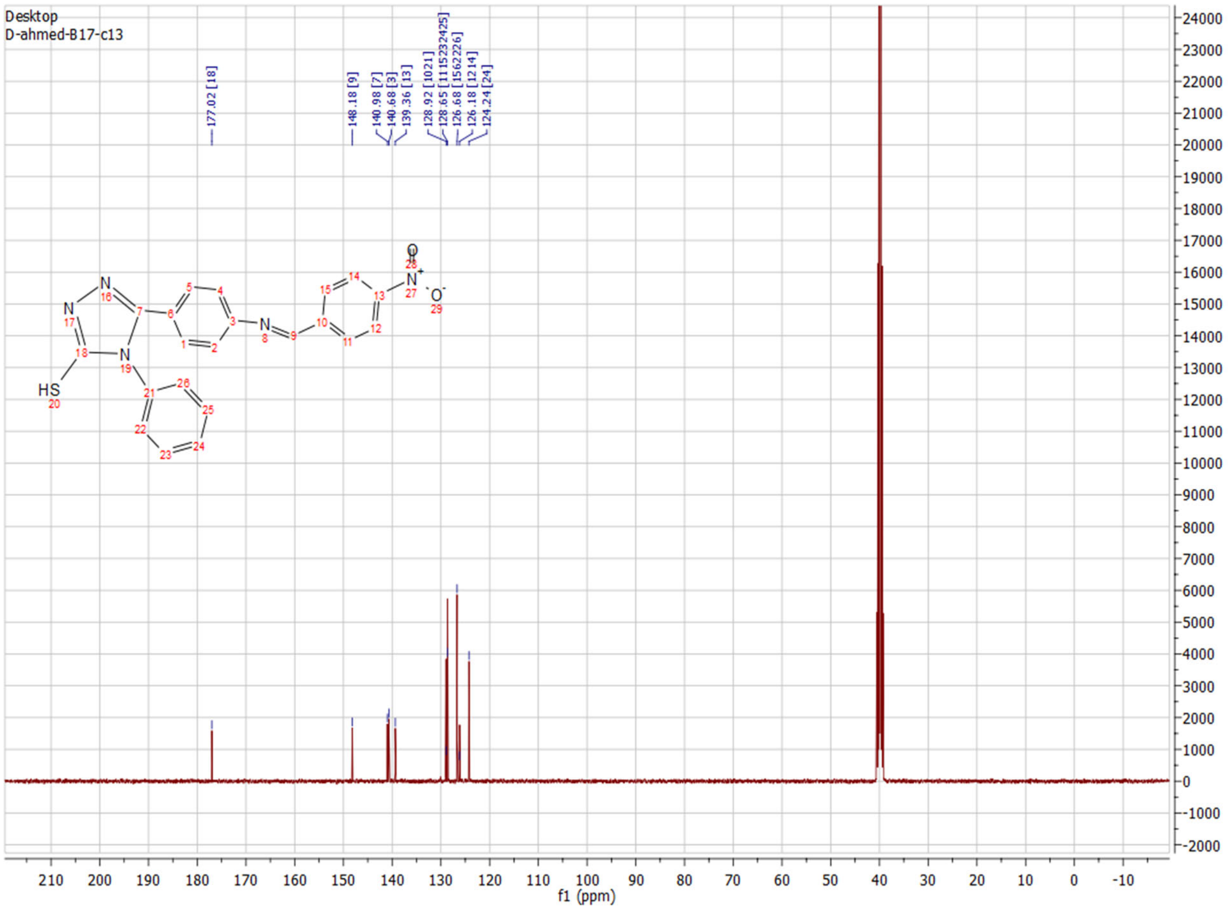

Figure S34b: ^{13}CNMR

Compound 9b

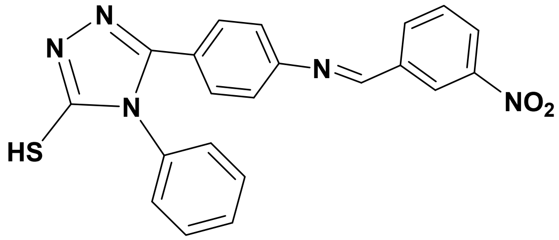

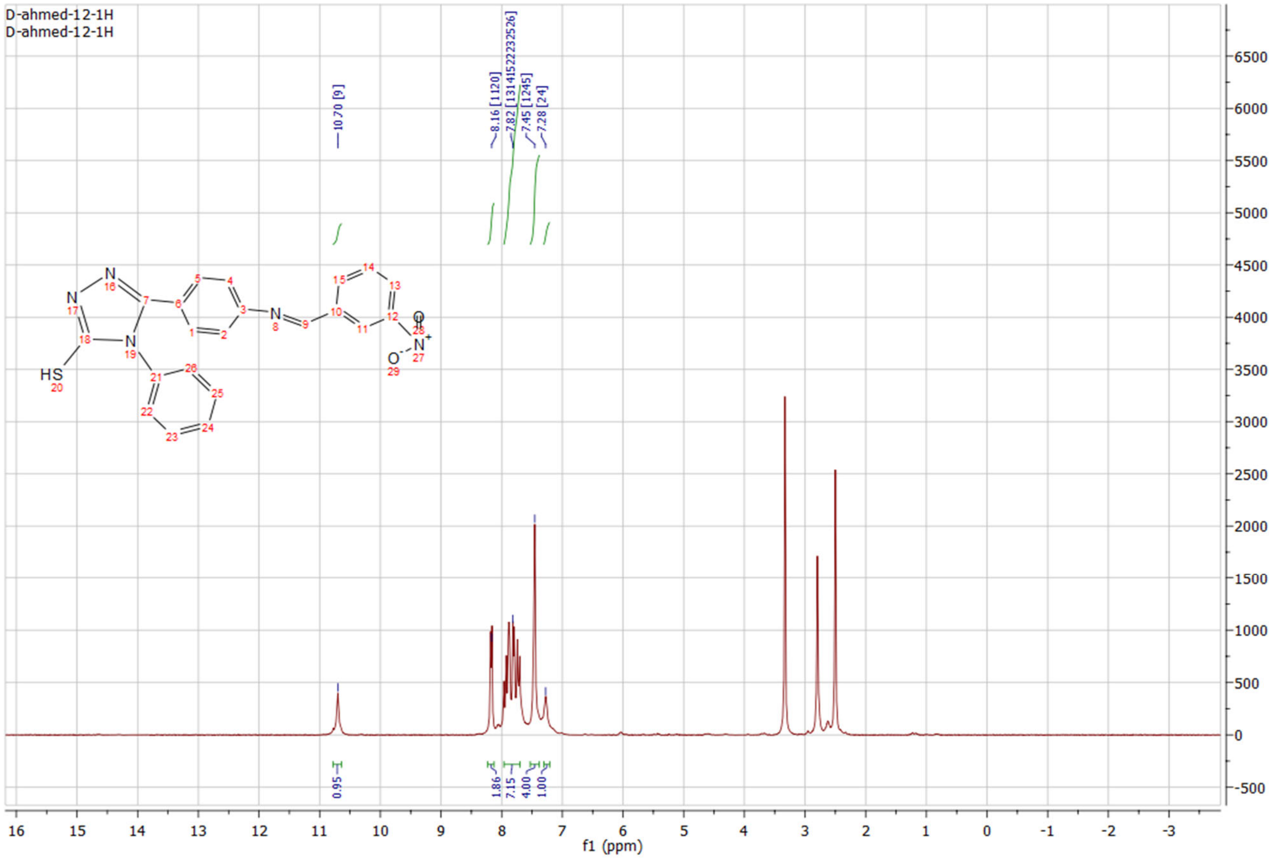

Figure S35a: ^{1}HNMR

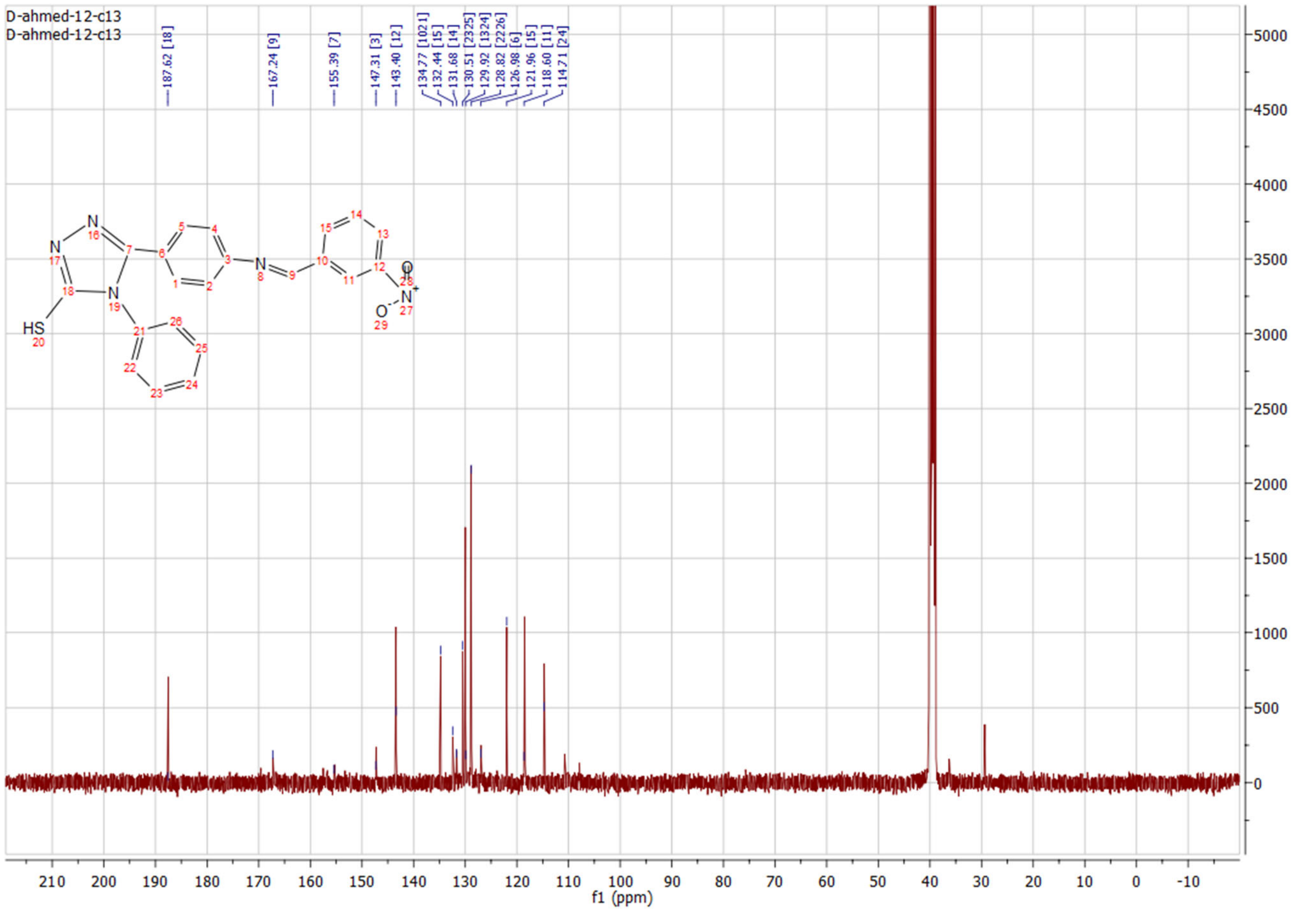

Figure S35b: ^{13}CNMR

Compound 10a

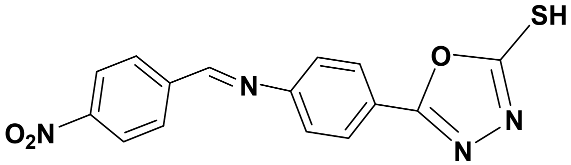

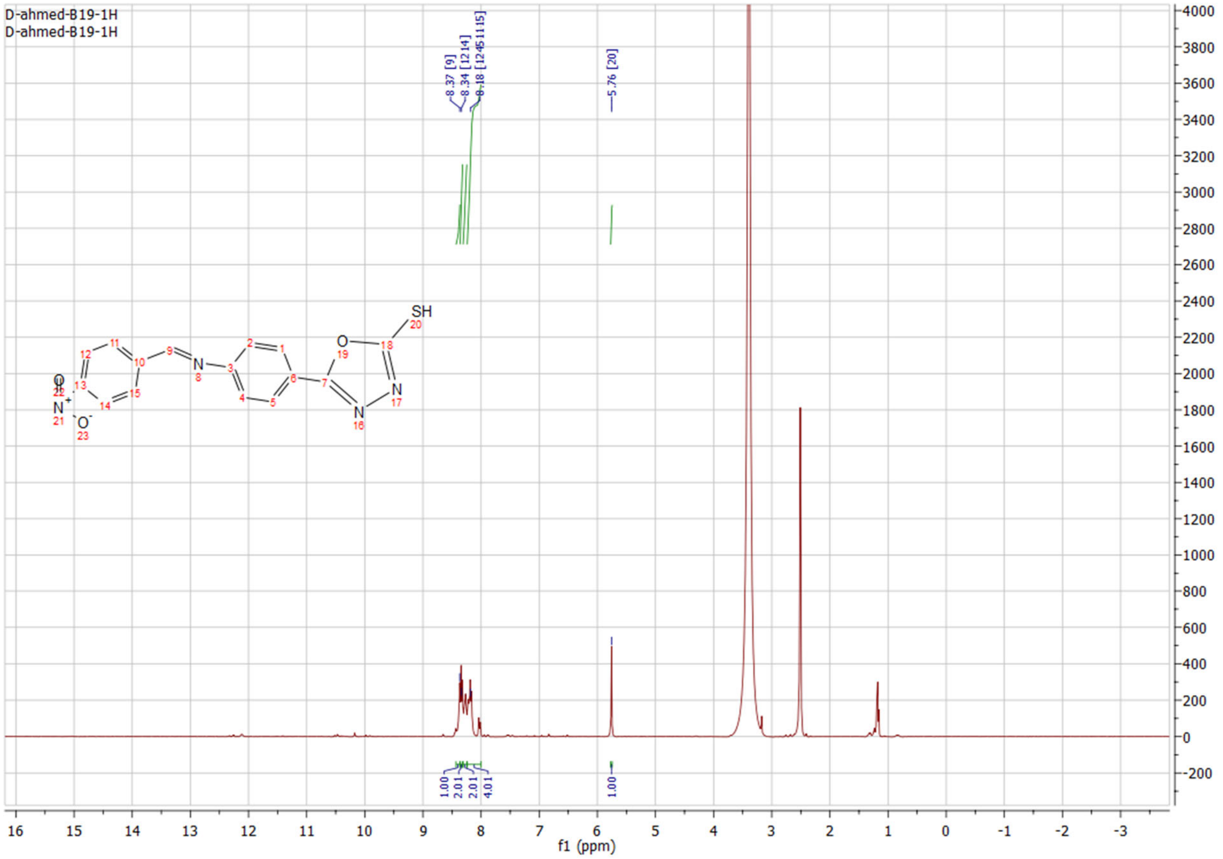

Figure S36a: ^{1}HNMR

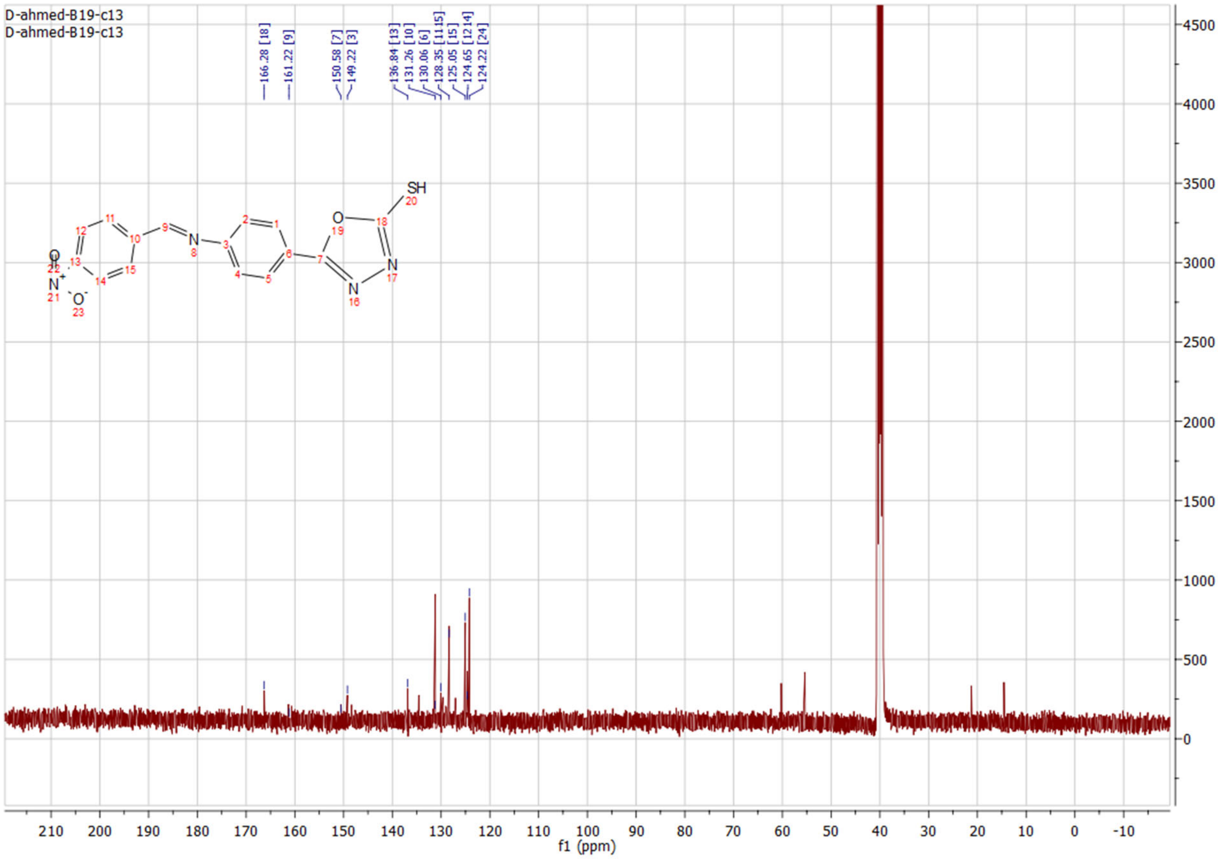

Figure S36b: ^{13}CNMR

Compound 10b

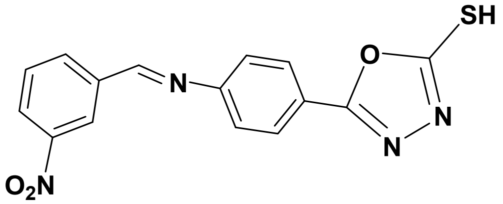

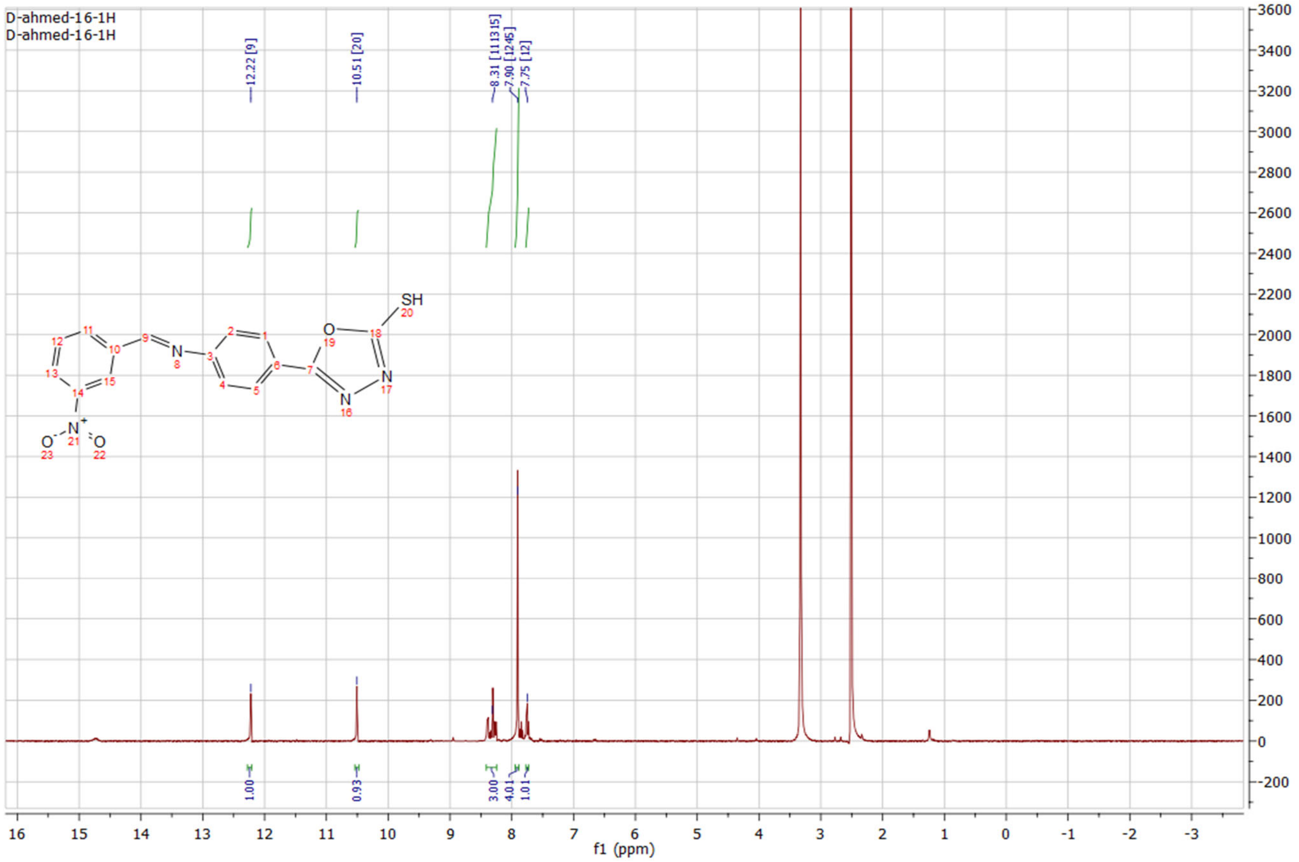

Figure S37a: ^{1}HNMR

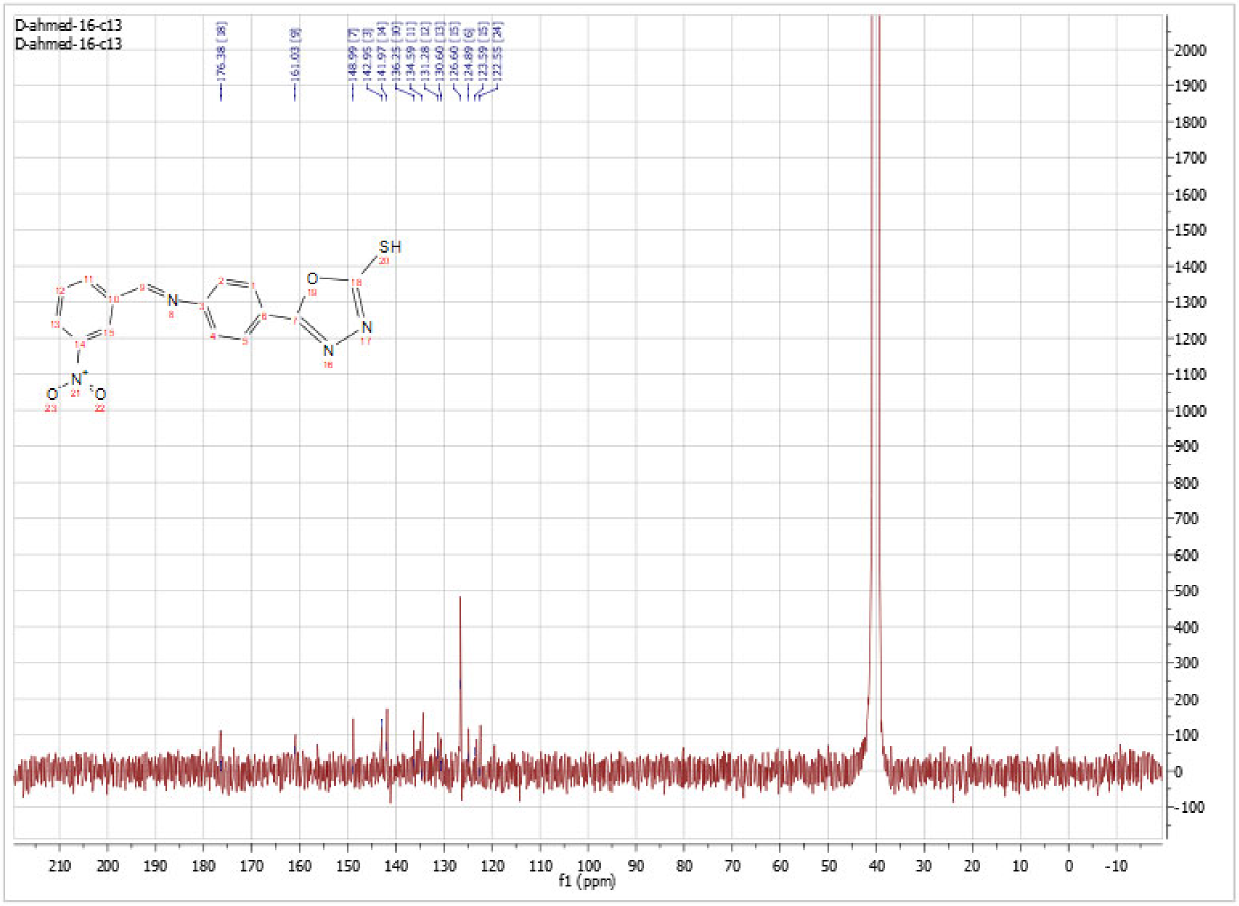

Figure S37b: ^{13}CNMR

Compound 10c

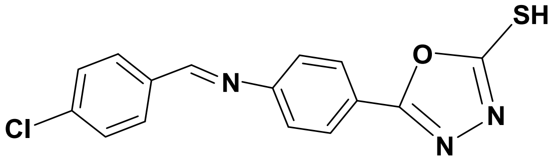

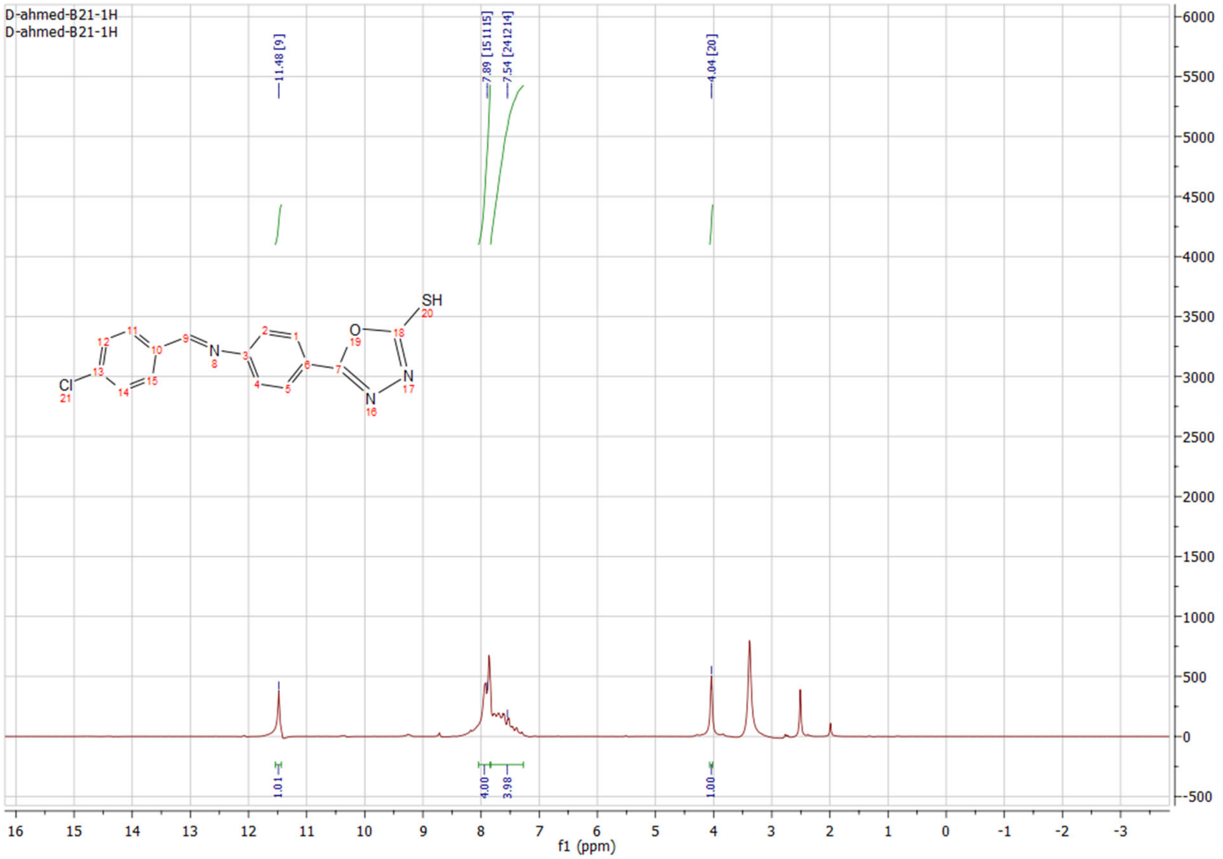

Figure S38a: ^{1}HNMR

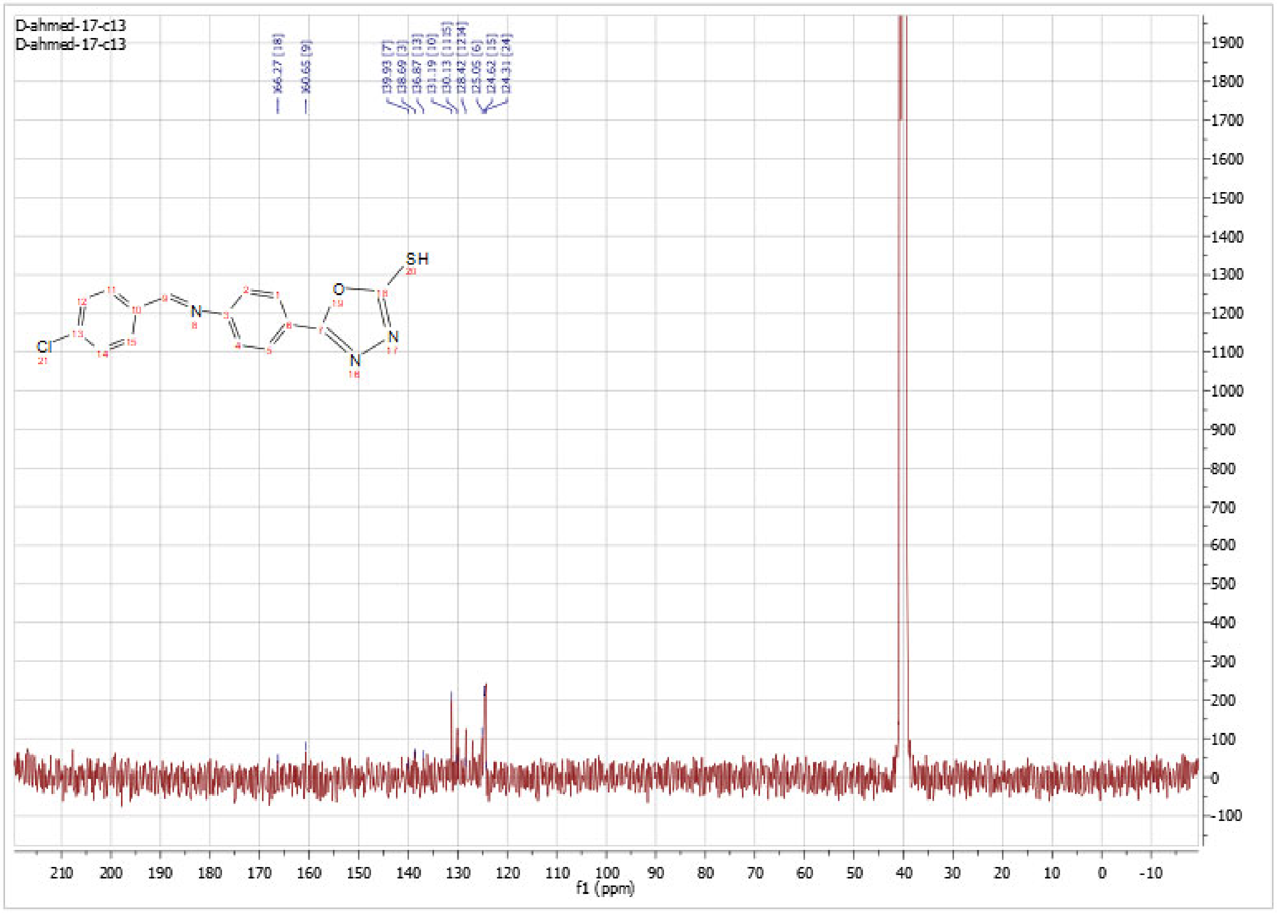

Figure S38b: ^{13}CNMR

Compound 18a

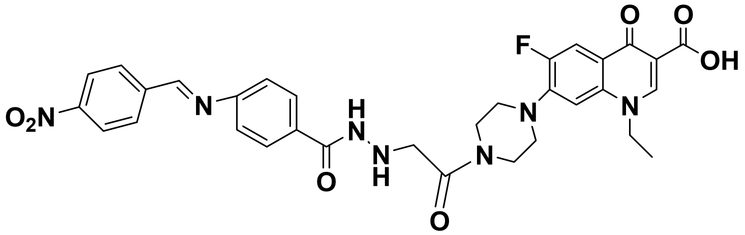

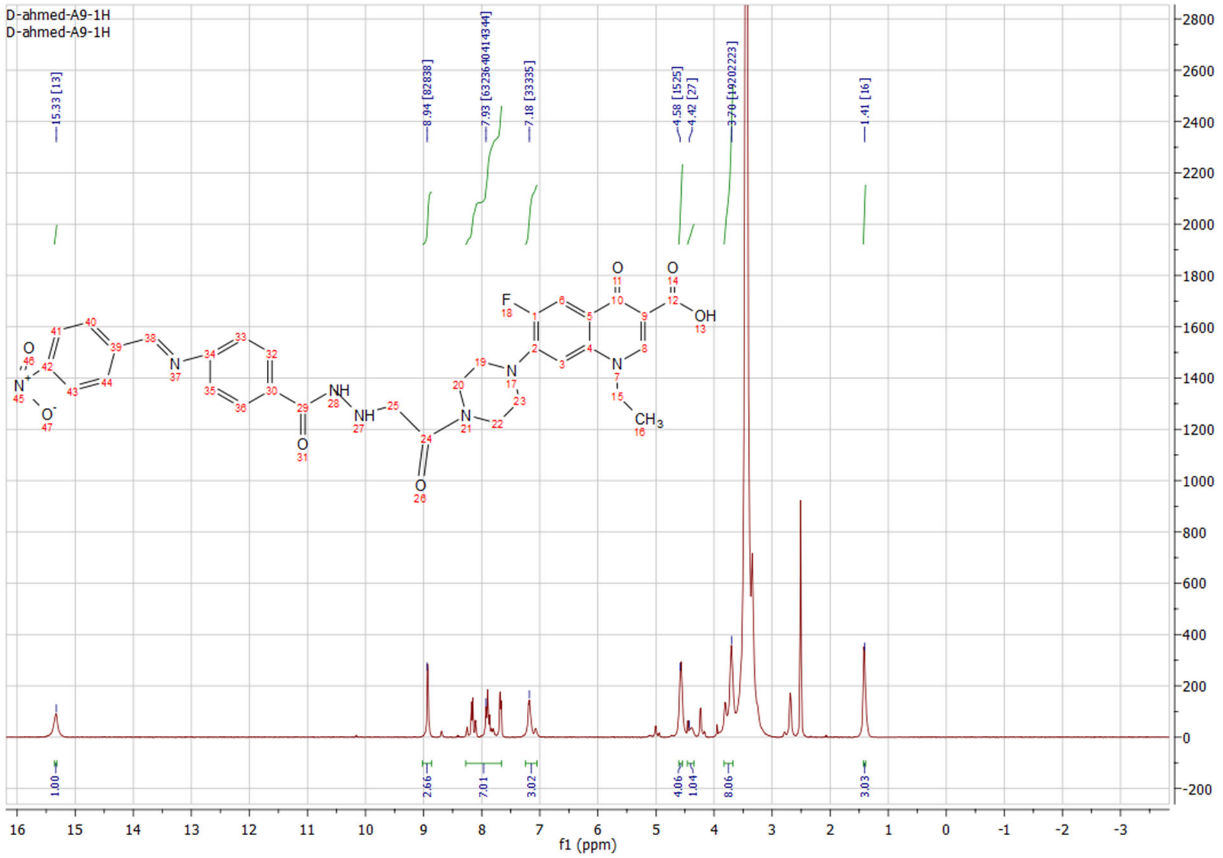

Figure S39a: ^{1}HNMR

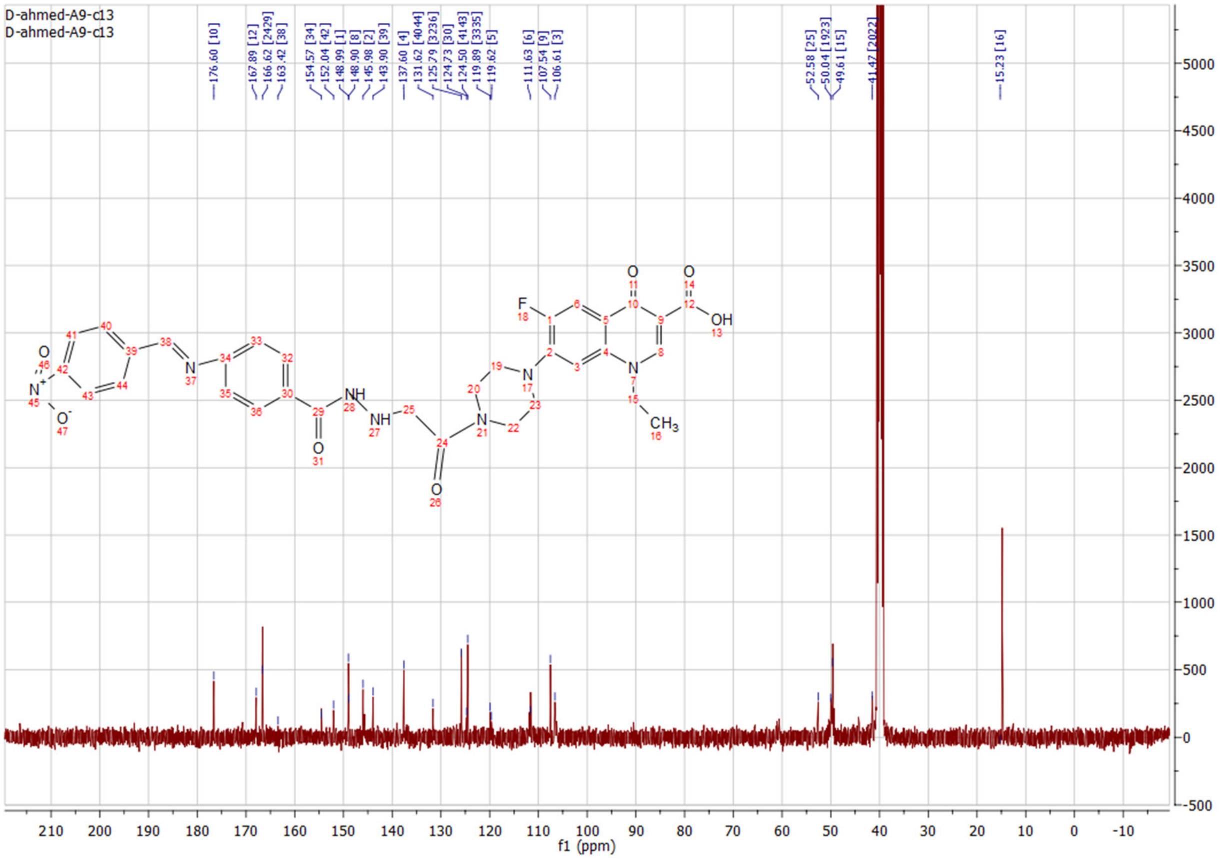

Figure S39b: ^{13}CNMR

Compound 18b

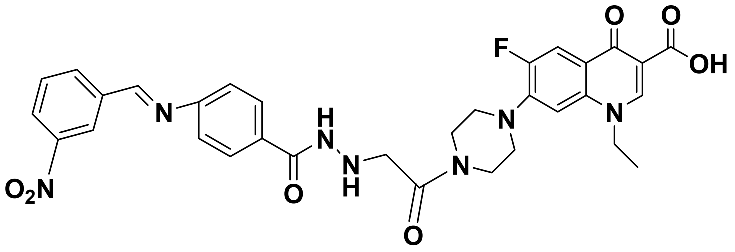

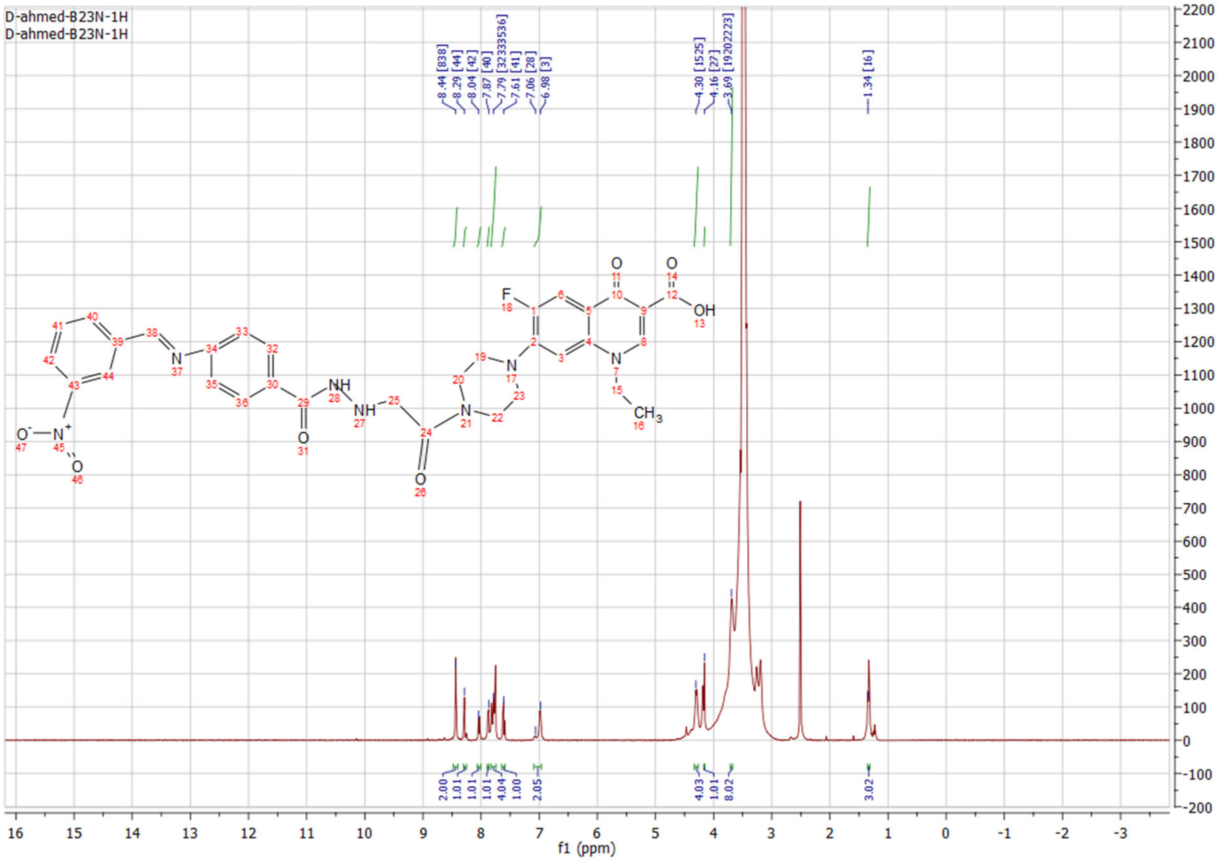

Figure S40a: ^{1}HNMR

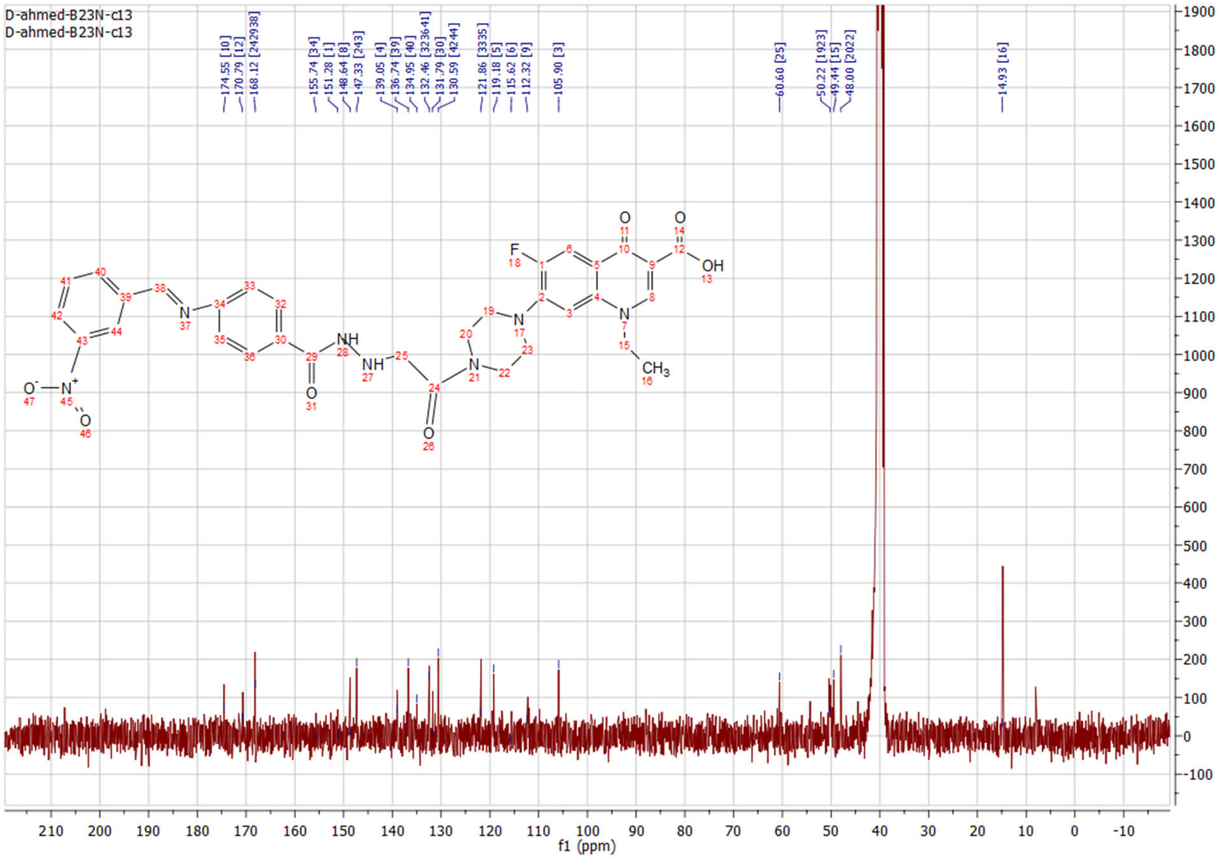

Figure S40b: ^{13}CNMR

Compound 18c

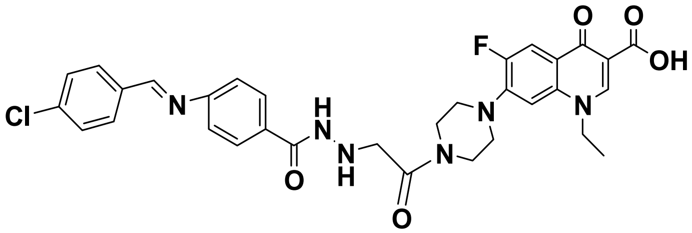

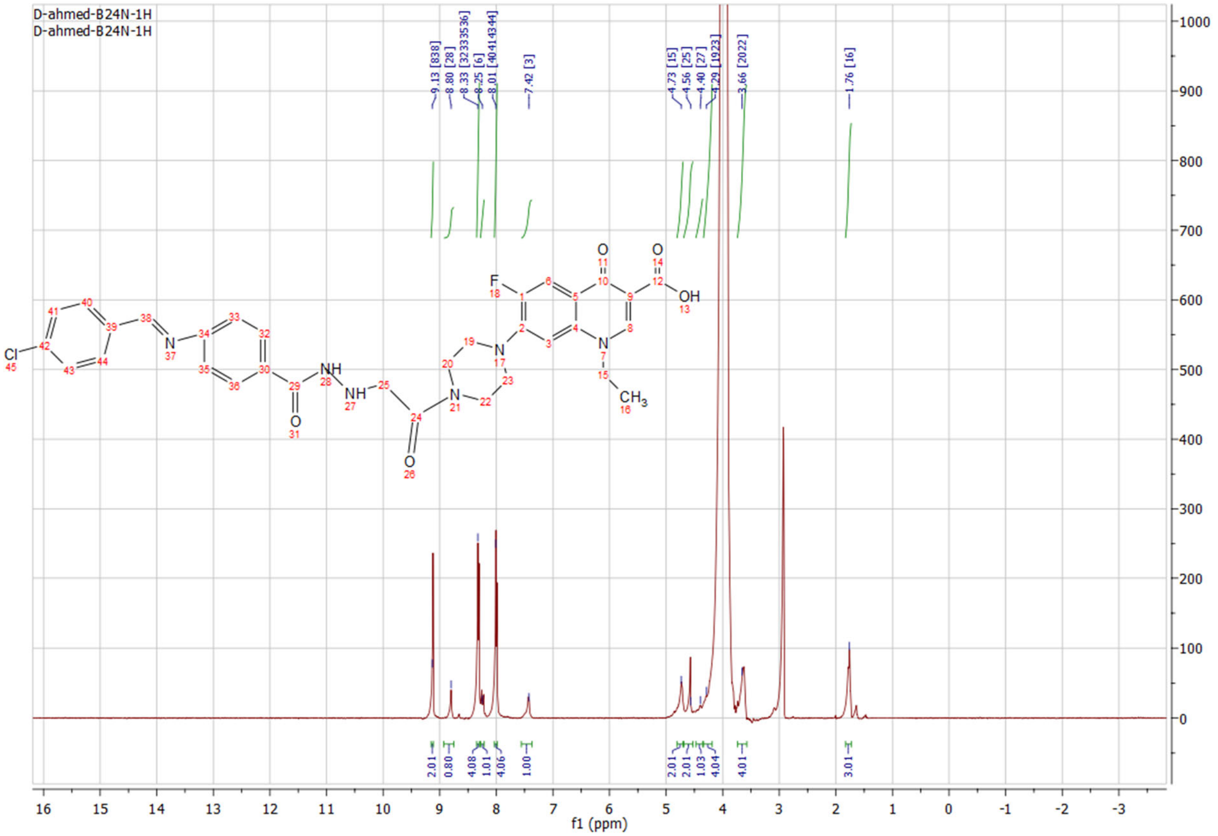

Figure S41a: ^{1}HNMR

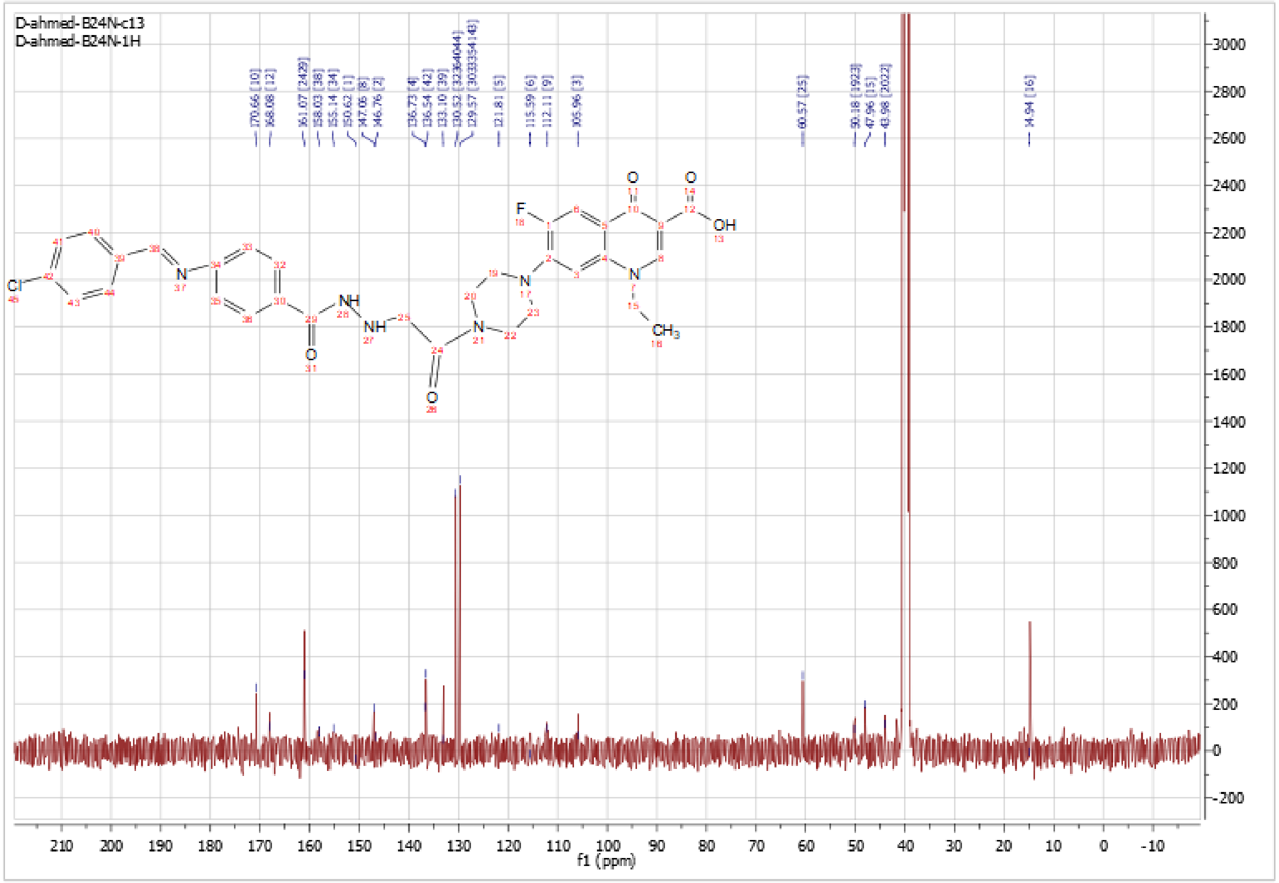

Figure S41b: ^{13}CNMR

Compound 12a

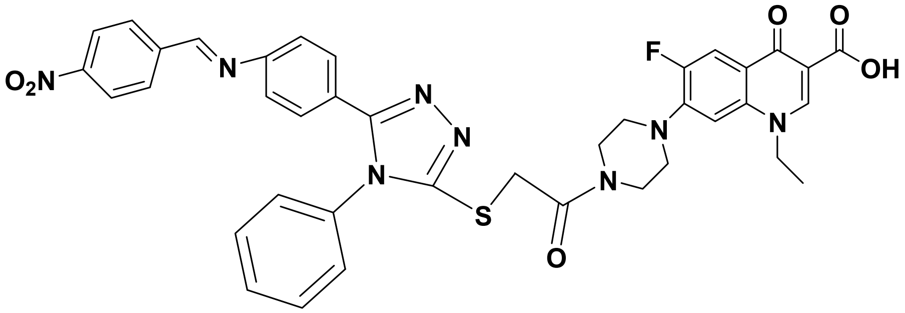

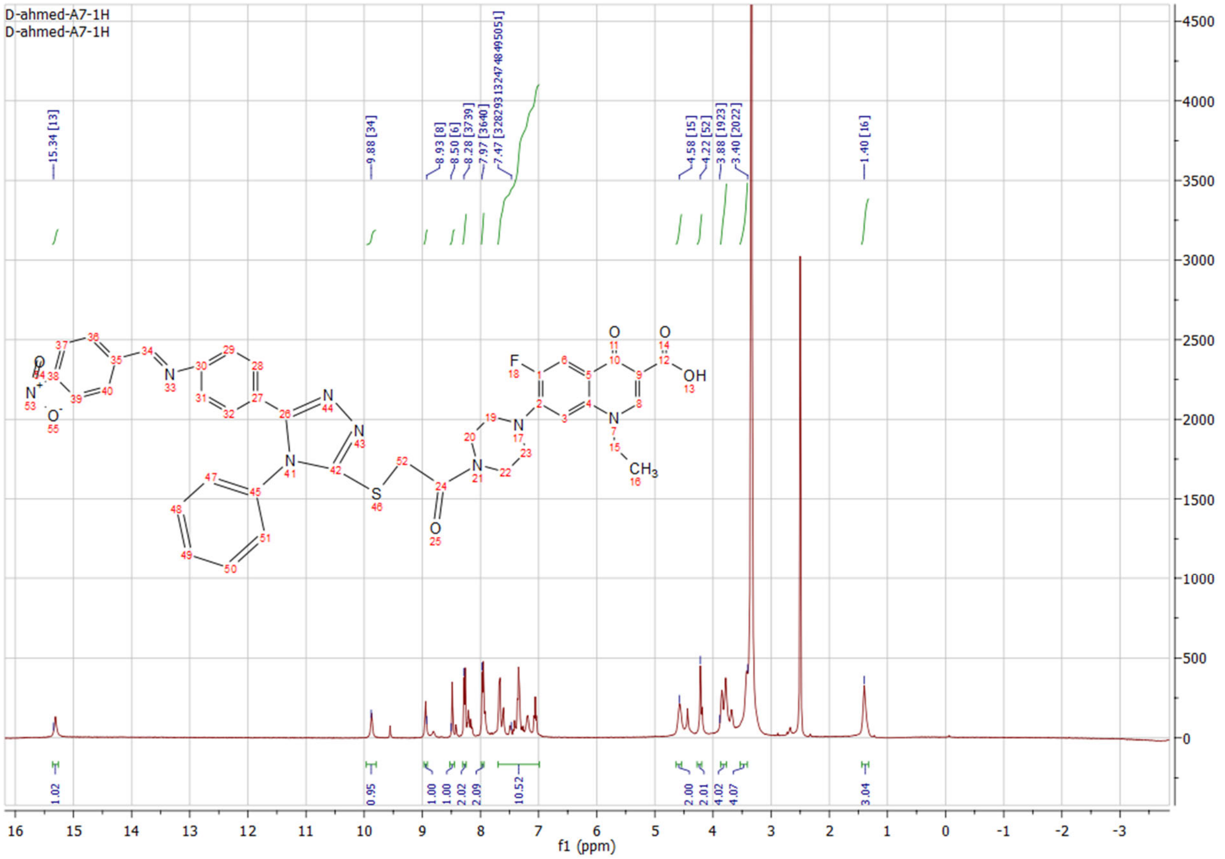

Figure S42a: ^{1}HNMR

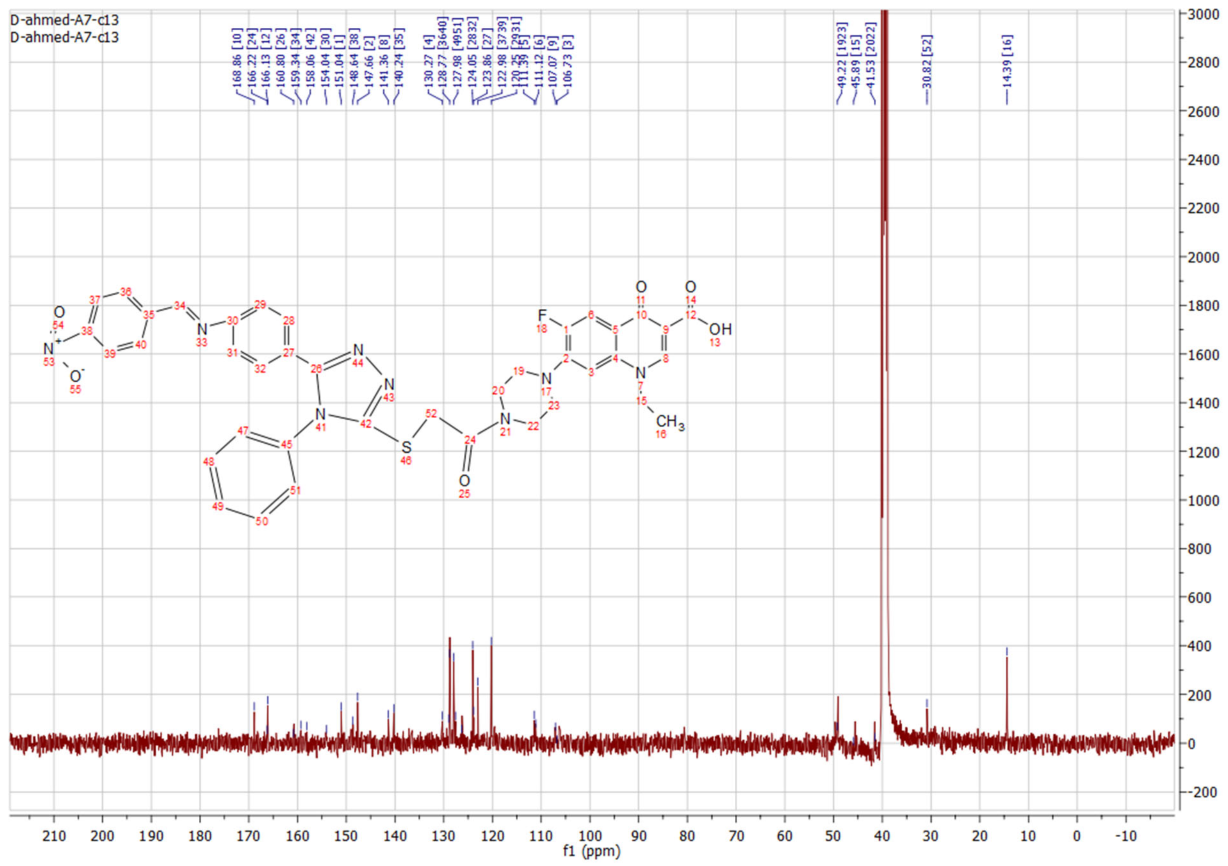

Figure S42b: ^{13}CNMR

Compound 12b

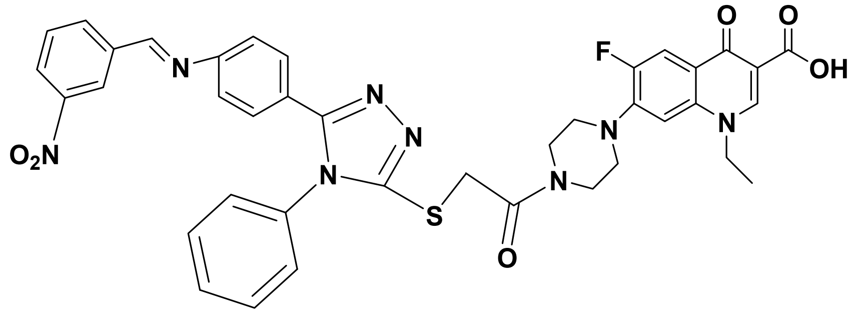

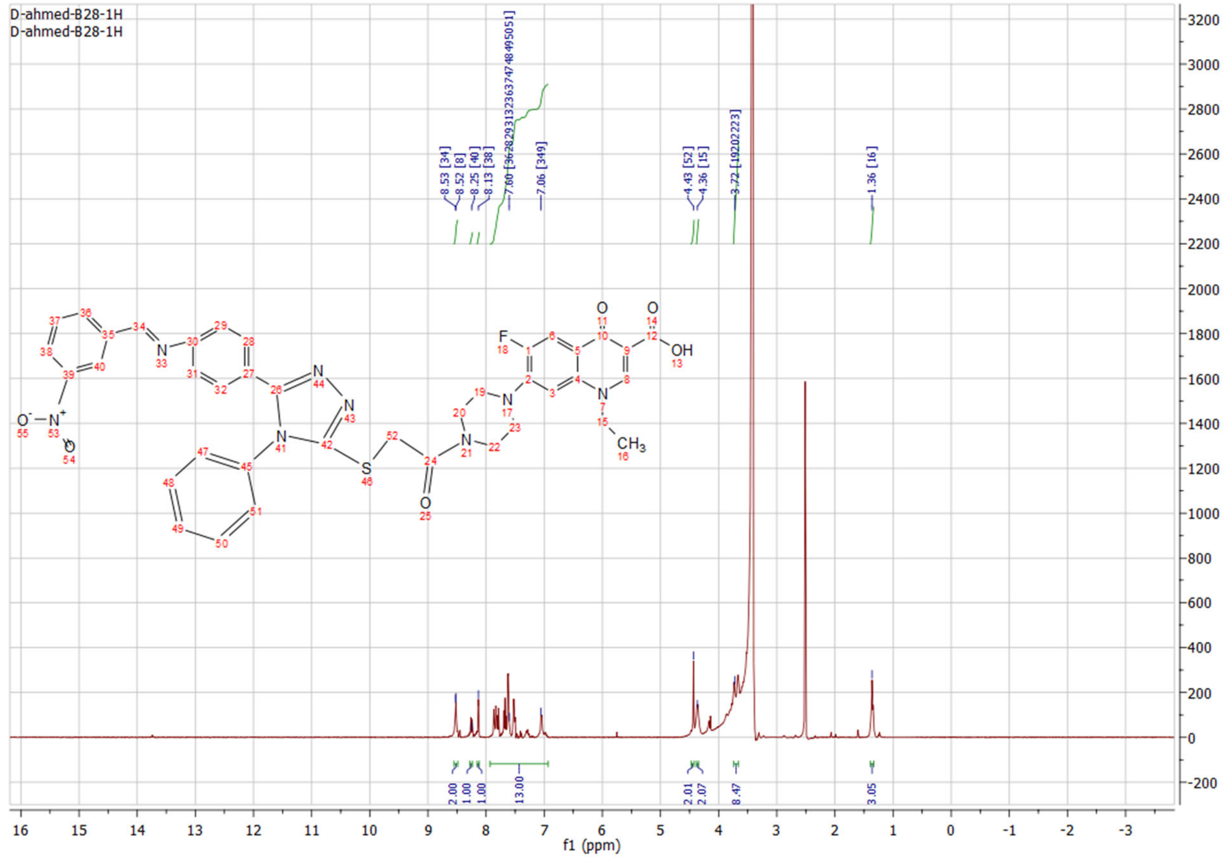

Figure S43a: ^{1}HNMR

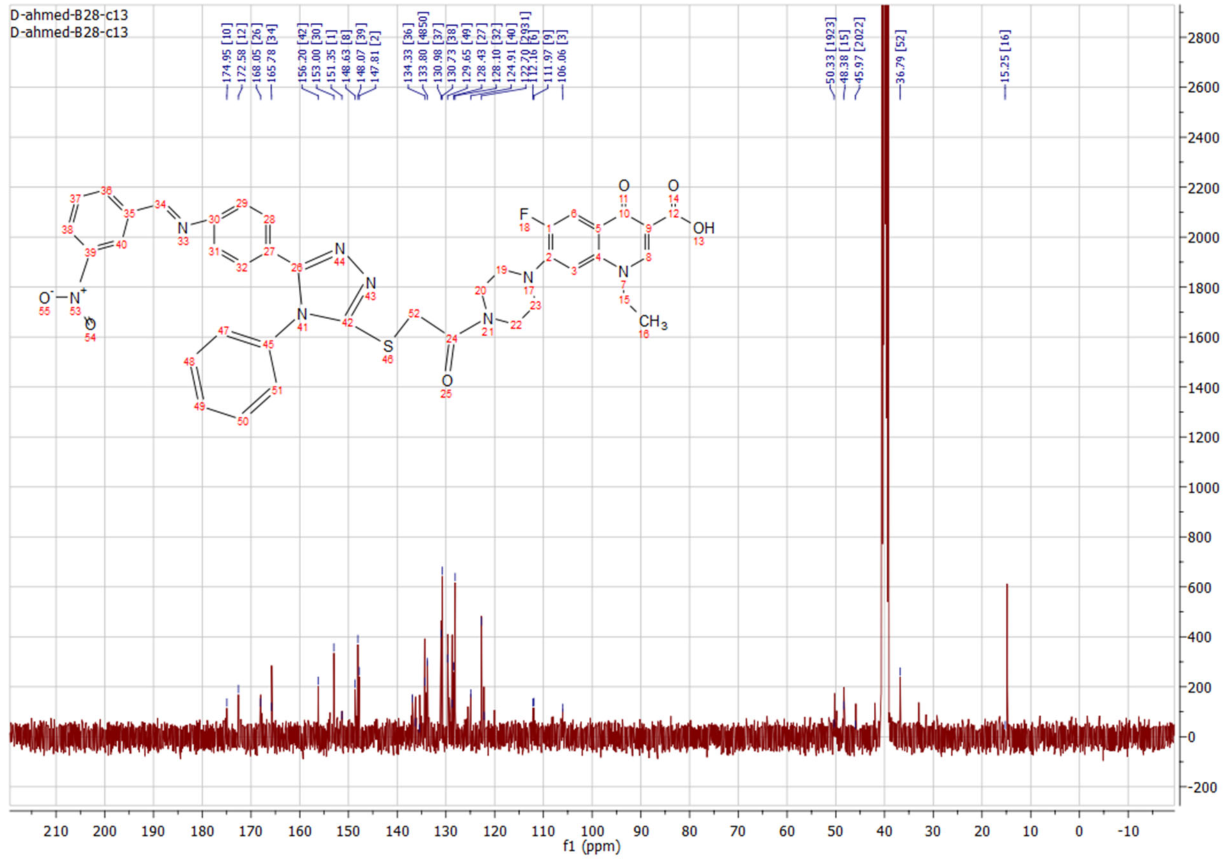

Figure S43b: ^{13}CNMR

Compound 13a

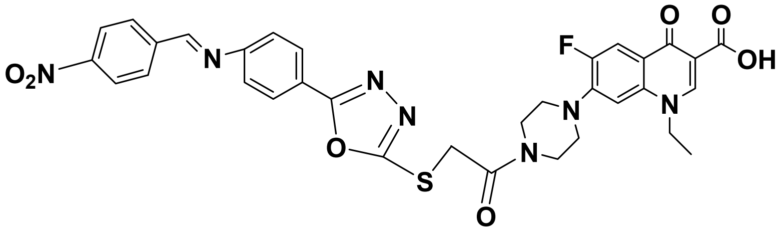

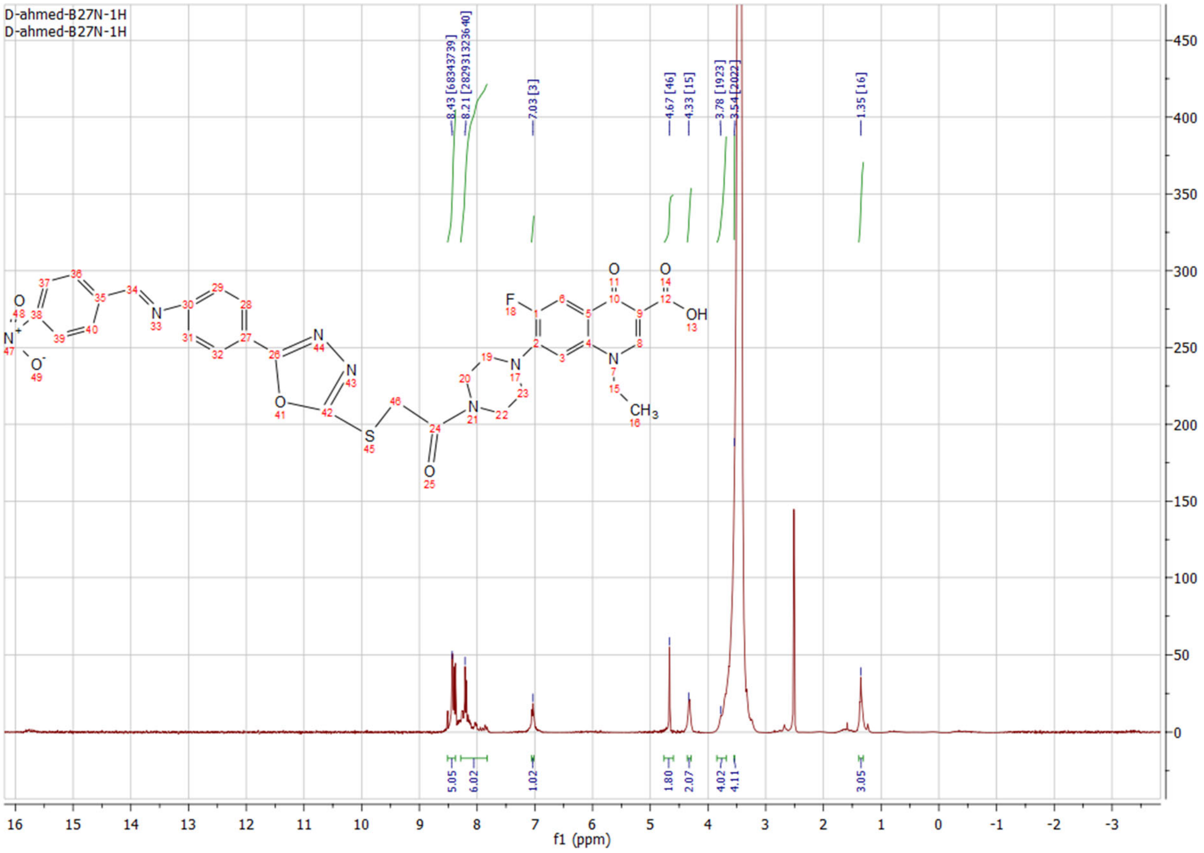

Figure S44a: ^{1}HNMR

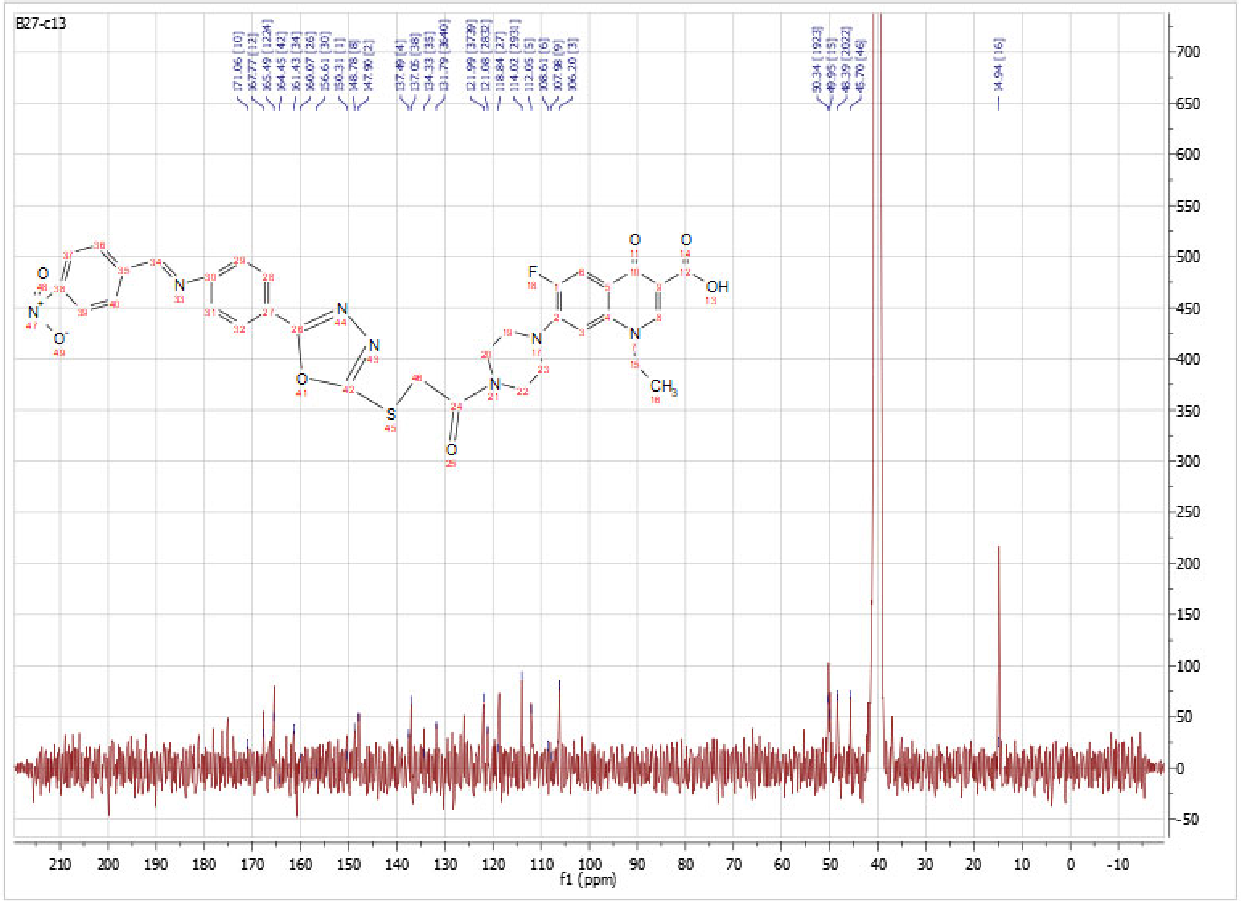

Figure S44b: ^{13}CNMR

Compound 13b

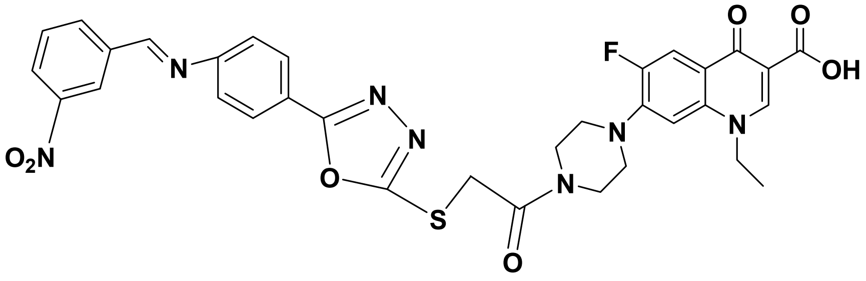

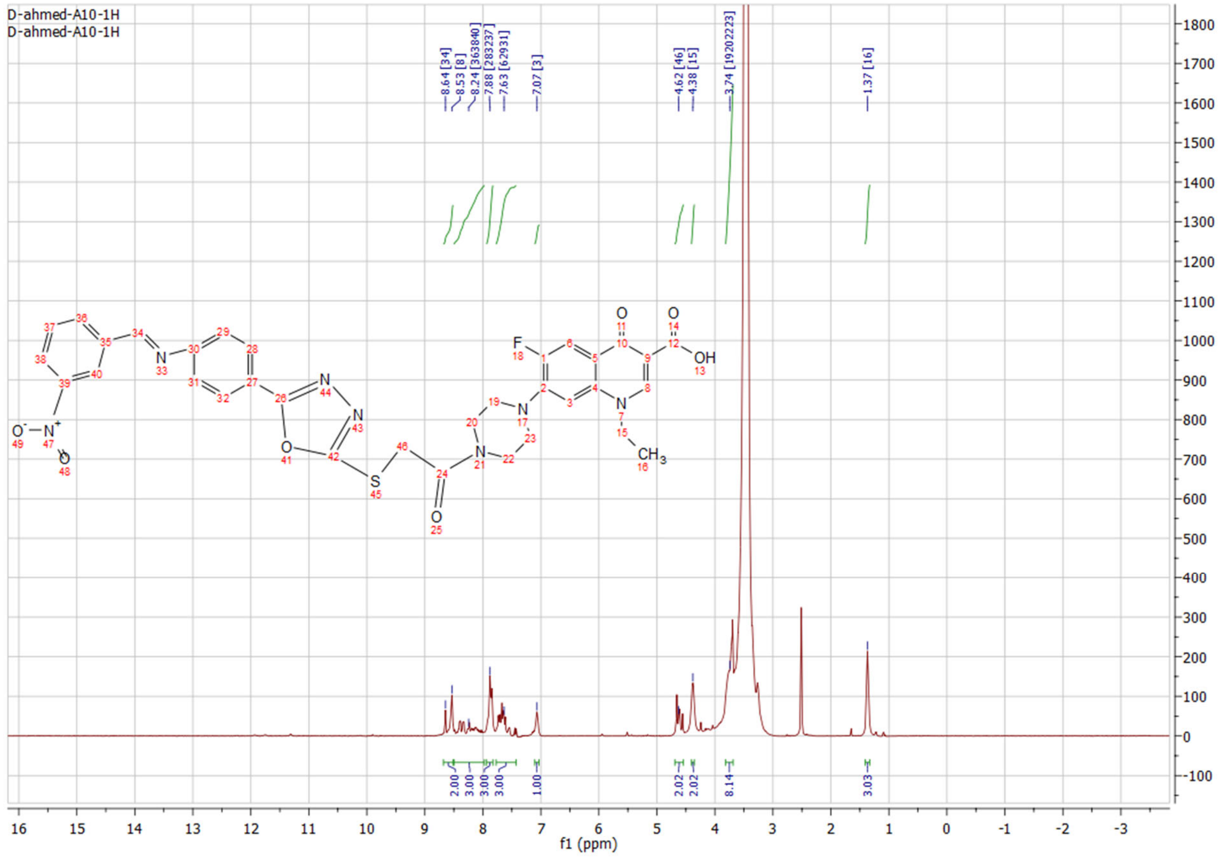

Figure S45a: ^{1}HNMR

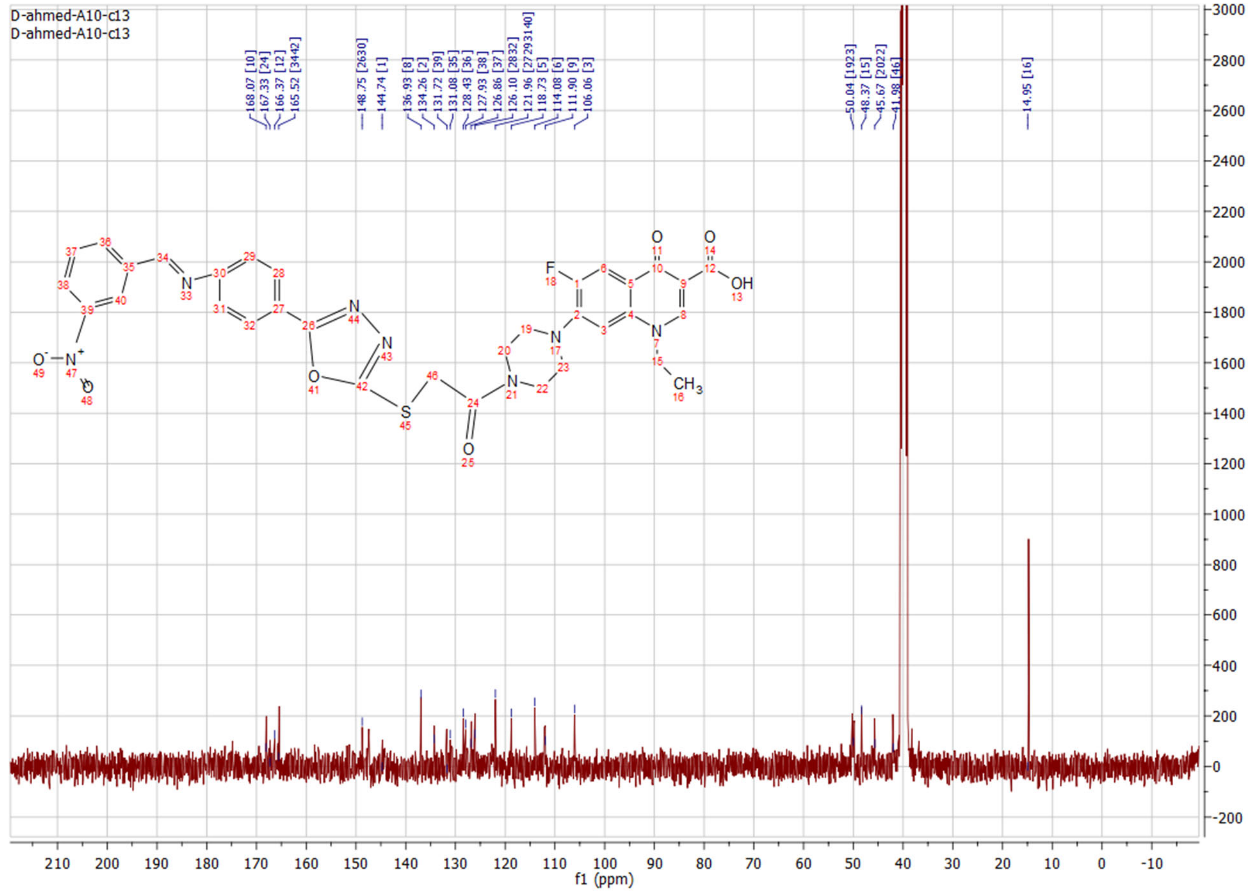

Figure S45b: ^{13}CNMR

Compound 13c

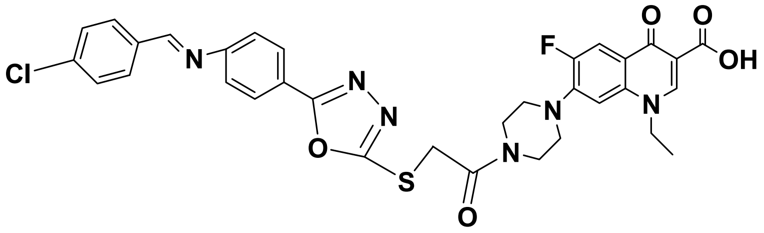

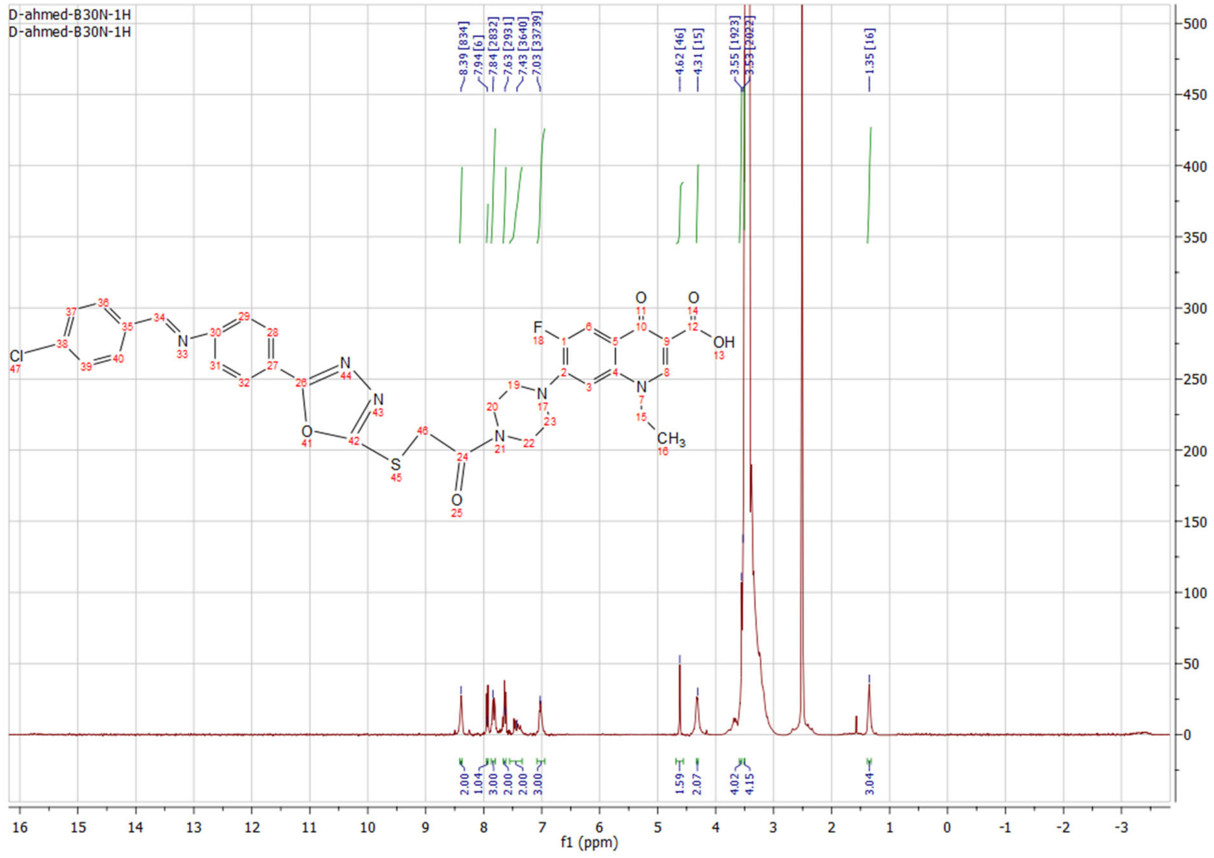

Figure S46a: ^{1}HNMR

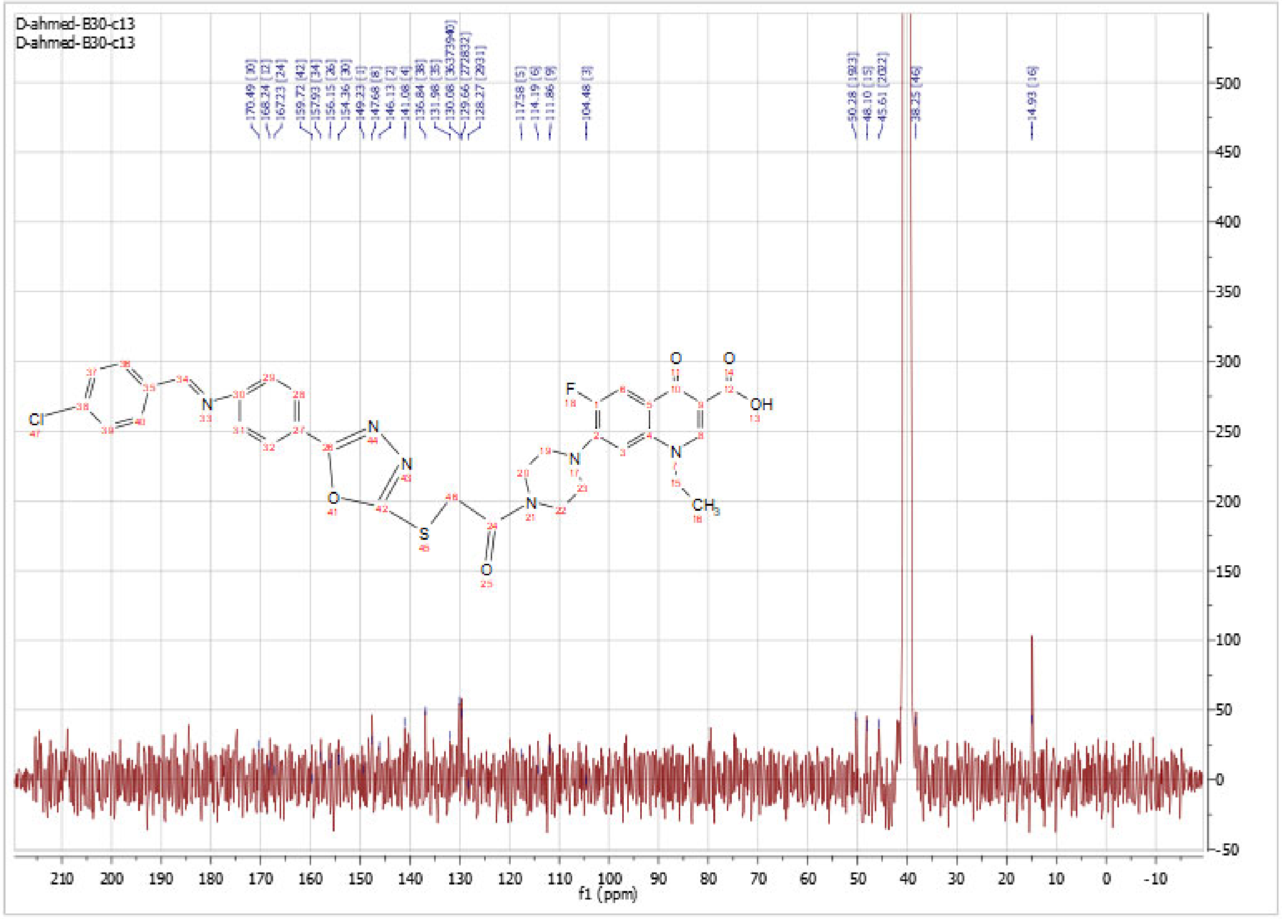

Figure S46b: ^{13}CNMR

Al-Azhar University
The Regional Center for Mycology and Biotechnology

Requester Data:
Name: Dr. Ahmed Mohsen Kamal
Authority: Faculty of Pharmacy, Assuit University

Sample Data:
Twelve samples had been submitted for elemental analysis.

Analysis Report:

Sample Code	C%	H%	N%
4a	63.97	5.71	12.59
4b	54.43	4.69	10.80
4c	59.47	5.15	11.78
4d	58.13	4.97	13.89
4e	62.46	5.72	11.88
4f	62.51	5.83	11.79
4g	62.49	5.81	11.84
4k	59.23	4.96	11.73
4h	60.29	5.17	11.52
4l	60.70	5.48	15.28
7a	62.43	6.71	12.84
7b	59.32	5.98	12.71

INVESTIGATOR DIRECTOR

Al-Azhar University
The Regional Center for Mycology and Biotechnology

Requester Data:
Name: Dr. Ahmed Mohsen Kamal
Authority: Faculty of Pharmacy, Assuit University

Sample Data:
Eleven samples had been submitted for elemental analysis.

Analysis Report:

Sample Code	C%	H%	N%
3b	60.49	6.78	13.58
3a	59.51	6.41	14.03
16	58.27	5.32	17.19
6a	61.43	4.79	11.28
6b	53.59	4.02	9.81
5a	55.13	4.95	15.51
5b	59.17	4.83	14.02
4i	61.38	5.37	11.87
4j	63.39	5.62	11.59
8b	50.86	4.53	20.54
14	58.21	4.73	13.29

INVESTIGATOR DIRECTOR

3c	62.71	7.04	12.49
8a	60.11	5.07	16.15
20a	60.71	4.59	11.46
20b	63.29	5.32	11.50
20c	61.59	5.12	11.28
21	59.11	5.34	15.19

17a	59.43	4.47	19.95	0
17b	59.37	4.39	19.88	0
17c	61.59	4.60	15.49	0
9a	62.70	3.94	17.62	8.12
9b	62.98	3.91	17.66	8.08
10a	55.43	3.27	17.43	9.95
10b	55.47	3.24	17.40	9.87
10c	57.28	3.28	13.54	10.29

INVESTIGATOR DIRECTOR

Sample Code	C%	H%	N%	S%
18a	59.94	4.81	15.50	0
18b	59.86	4.85	15.49	0
18c	60.89	4.92	13.51	0
12a	61.78	4.59	14.90	4.32
12b	61.76	4.62	14.98	4.29
13a	58.04	4.29	14.47	4.79
13b	57.96	4.23	14.52	4.58
13c	58.94	4.32	12.71	4.80

INVESTIGATOR DIRECTOR

Figure S47: Elemental analysis certificates

3. Quantitative structure activity relationship (QSAR)

Text S2: Quantitative structure activity relationship (QSAR)

QSAR analysis is a method used to build models that correlate between the biological activity and the structural descriptors of a library of compounds [1]. The generated models can be crucial for assessing the structural basis of the antibacterial activity and the substituent effects on bioactive compounds such as our *N4*-piperazinyl derivatives [2].

Here, we constructed a QSAR model using MOE 2020.01 (**Text S3**). A set of 24 newly synthesized derivatives (**Tab. S1**) was selected as training set based on their structural diversity. The biological activity was expressed as their log MIC value against *S. aureus* CCUG1800T. Molecular descriptors were chosen to cover a wide range of different thermodynamic, electronic, hydrophobic, geometric, topological, and quantum mechanical properties that are strongly related with biological activity [3]. Multiple linear regression analysis was used to derive the optimum QSAR model that correlates the biological activity with the calculated descriptors of the training set. Model validation was carried out by the normal, Leave-one-out (LOO) cross-validation, and external validation procedures [4].

Creation of QSAR model

For the current dataset of 24 compounds, the QSAR model was restricted to a maximum of four variables (descriptors ratio to be around 5:1).

Equation 1: Best performing QSAR model for the antibacterial activity against *S. aureus*.

Log MIC = -1.36959 + 0.00844 E_vdw + 0.34847 KierFlex - 0.00235 TPSA + 0.18451 Log P(o/w)   (Equation 1)

n = 24, RMSE= 0.13259,

r = 0.97717, r^2 = 0.95487,

q^2 = 0.867109 (Cross-validated squared correlation coefficient r^2 (LOO-q^2)).

The generated model showed that the biological activity positively correlated with Van der Waals energy (E_vdw), which is the potential energy Van der Waals component that contributes to the

formation of protein-ligand complexes. The antibacterial activity was also affected by the Kier molecular flexibility index (KierFlex), which is a topological descriptor of the flexibility of the molecule. The polar surface area (TPSA) was inversely correlated with the antibacterial activity of the synthesized derivatives. TPSA is widely used in QSAR models to correlate the ligand polar interactions with a specific target (mainly H-bonding). Furthermore, the antibacterial activity was correlated with the lipophilicity descriptor Log Octanol/water partition coefficient LogP(o/w). The lipophilicity of an active molecule Log P(o/w) demonstrates the ability of the compound to penetrate through hydrophobic barriers to reach its site of action (Fig. S48).

Statistical normal and cross-validation techniques have been applied to estimate the quality of the predictive ability of the generated model (**Tab. S2-3**). The validation values r, r^2, and q^2 indicate that the created model has a good predictive ability and correlation between biological activity and calculated molecular descriptors as r value higher than 0.9, r^2 value higher than 0.8, and q^2 value higher than 0.5 [5]. Another parameter to evaluate the quality and predictivity of the created model was the $r^2 - q^2$ value, which should not exceed 0.3 [6], and here the difference is 0.087761.

External validation of the model

External validation of the obtained model was achieved by using a test set of 9 compounds (**Tab. S6**). The QSAR model was used to predict their Log MIC, and then correlation between experimental and predicted Log MIC was analyzed. The r^2 for external validation was 0.7868, which indicates good predictive ability and robustness of the created model as acceptable r^2 values for external validation should be higher than 0.6 for a satisfactory model [6] (**Fig. S49)**.

Text S3: QSAR model

QuaSAR-Model(PLS): c:/users/ahmed716/desktop/qsar 2/training set.mdb

Mon Apr 04 23:24:49 2022

Activity Field : LogMIC

Weight Field : None

Condition Limit : 1e+006

Component Limit : 0

Observations : 24

Descriptors : 4

Components Used : 4

Condition Number : 23893.348

ROOT MEAN SQUARE ERROR (RMSE): 0.13259

CORRELATION COEFFICIENT (R2) : 0.95487

ESTIMATED LINEAR MODEL

LogMIC =

-1.36959

+0.00844 * E_vdw

+0.34847 * KierFlex

-0.00235 * TPSA

+0.18451 * logP(o/w)

RELATIVE IMPORTANCE OF DESCRIPTORS

0.141569 E_vdw

0.381215 KierFlex

0.079167 TPSA

0.291061 logP(o/w)

- **Cross-validation (internal validation):**

XRMSE=0.36418

XR2=0.867109

Tab. S1: Values of molecular descriptors for training and test set compounds.

	...	ID	LogMIC	AM1_dipole	ASA_H	E_sol	E_vdw	KierFlex	logS	mr	Q_VSA_F...	rgyr	TPSA	vdw_area	vol	VSA	weinerPol	logP(o/w)
1		4a	0.3440	7.0090	540.9705	-36.8637	73.988	4.6833	-4.3999	12.1286	0.5916	5.9283	93.1900	447.6459	420.3750	454.	57.0000	1.7030
2		4c	0.3110	7.2628	556.3414	-56.8710	56.418	5.3806	-5.1342	12.6380	0.6070	5.7887	93.1900	465.2273	460.6250	510.	59.0000	2.2950
3		4f	0.3150	6.7126	578.3163	-31.4836	56.338	5.3207	-4.4502	12.7712	0.5617	6.5559	102.4200	479.8817	468.0000	515.	61.0000	1.6590
4		16	0.3030	5.9292	557.1734	-33.6877	31.242	5.3861	-3.1887	12.8136	0.5223	6.2509	135.1800	484.0485	496.0000	557.	62.0000	0.0200
5		7a	0.9530	7.6496	517.2466	-31.9105	66.836	5.2600	-3.4611	11.7555	0.5493	5.2317	84.4000	444.7314	416.1250	455.	56.0000	1.2910
6		2	0.4010	9.6880	348.0848	-40.2300	56.439	4.5037	-3.5743	9.8281	0.4656	4.9442	81.1600	376.9911	343.0000	379.	49.0000	0.9940
7		4d	0.3110	7.0092	480.2172	-31.7610	78.713	5.1955	-5.1901	12.6428	0.5360	6.3643	139.0100	475.7842	440.0000	481.	63.0000	1.6380
8		3a	0.6930	5.2226	463.8798	-32.3538	59.785	4.8782	-2.9032	10.5553	0.5238	5.1618	93.1900	411.6118	374.5000	418.	51.0000	0.3900
9		5b	1.7900	9.1342	574.6436	-22.1770	47.847	5.1065	-5.6867	13.4510	0.5603	6.6727	106.0800	473.9200	489.3750	539.	63.0000	2.3300
10		4j	0.3050	8.4228	580.3395	-29.5900	53.625	5.2358	-4.7121	13.1383	0.5793	6.6448	110.2600	486.9034	479.5000	527.	63.0000	1.5560
11		6a	0.2940	5.4650	493.3344	-38.3907	74.874	4.4405	-5.1533	13.0071	0.5295	6.1434	118.5400	473.1201	443.8750	481.	70.0000	0.7910
12		3c	0.3380	7.0388	573.1783	-26.3598	61.759	5.7071	-4.0471	12.2206	0.5885	5.6455	93.1900	451.9167	440.5000	493.	57.0000	2.0280
13		4l	0.9450	6.4739	496.7192	-34.2758	73.744	4.6438	-3.1417	11.9554	0.5772	5.5398	106.0800	445.7902	413.1250	452.	57.0000	0.4710
14		4h	0.6040	6.2855	469.8829	-22.3575	77.845	5.2358	-4.3693	12.8170	0.5222	5.7671	130.4900	479.7013	445.1250	486.	63.0000	1.3800
15		3b	0.3760	8.1087	532.1318	-33.7487	37.351	5.3386	-3.1050	11.0281	0.5429	5.1887	93.1900	428.8439	421.7500	469.	52.0000	1.0040
16		5a	0.3360	7.0566	536.5938	-30.8439	41.636	5.0087	-3.8712	11.8249	0.5379	6.5516	106.0800	436.4624	429.3750	487.	54.0000	0.6840
17		4g	0.3150	8.6842	569.3973	-31.9990	58.492	5.3207	-4.4502	12.7712	0.5617	6.6853	102.4200	479.8817	465.6250	509.	61.0000	1.6960
18		4e	0.3150	9.1853	566.0490	-17.5681	63.523	5.3207	-4.4502	12.7749	0.5617	5.9849	102.4200	479.8817	466.5000	507.	62.0000	1.6570
19		18a	0.1900	7.6328	676.0071	-52.7874	67.278	7.1183	-7.1618	16.7305	0.5356	8.3401	180.4700	615.7256	614.3750	671.	80.0000	2.8190
20		8a	0.3670	3.5143	454.7698	-53.1722	44.811	4.1037	-3.1016	11.0714	0.5353	5.5253	98.9800	422.7965	403.8750	445.	53.0000	0.1820
21		12a	0.4740	6.4068	783.4682	-41.8828	94.584	7.9646	-11.9435	20.4127	0.6026	9.2842	170.0500	713.9664	712.8750	767.	96.0000	6.2910
22		13a	1.0660	6.0424	681.4841	-39.9058	66.393	7.4817	-10.9085	17.7720	0.5229	9.5327	178.2600	630.6871	645.1250	716.	83.0000	4.4720
23		4k	0.3090	7.8669	578.4159	-13.1298	42.518	5.0109	-4.9898	12.2137	0.5995	6.1325	93.1900	456.4643	463.7500	506.	62.0000	2.0440
24		4i	0.3260	5.9859	609.1835	-48.0631	33.546	4.8468	-4.6948	12.1756	0.5956	6.1755	93.1900	452.0551	467.7500	520.	59.0000	1.8560

	1	2	3	4	5	6	7	8	9	10	11	12	13	14	15	16
1. LogMIC	100	11	3	23	8	7	-17	12	-8	11	4	6	8	9	6	15
2. AM1_dipole	11	100	0	41	-5	5	3	-8	2	-6	-21	-9	-6	-7	-10	12
3. ASA_H	3	0	100	-11	17	83	-78	86	55	84	62	86	91	92	81	81
4. E_sol	23	41	-11	100	-2	-16	20	-20	3	-20	-23	-21	-20	-20	-17	-14
5. E_vdw	8	-5	17	-2	100	39	-48	49	6	33	43	48	32	27	52	48
6. KierFlex	7	5	83	-16	39	100	-87	91	15	87	80	93	92	92	86	86
7. logS	-17	3	-78	20	-48	-87	100	-95	-19	-93	-78	-93	-92	-91	-93	-95
8. mr	12	-8	86	-20	49	91	-95	100	24	95	86	99	98	97	98	89
9. Q_VSA_FHYD	-8	2	55	3	6	15	-19	24	100	14	-13	21	26	25	19	37
10. rgyr	11	-6	84	-20	33	87	-93	95	14	100	87	94	95	95	92	83
11. TPSA	4	-21	62	-23	43	80	-78	86	-13	87	100	88	85	85	87	61
12. vdw_area	6	-9	86	-21	48	93	-93	99	21	94	88	100	98	97	98	87
13. vol	8	-6	91	-20	32	92	-92	98	26	95	85	98	100	100	95	86
14. VSA	9	-7	92	-20	27	92	-91	97	25	95	85	97	100	100	94	85
15. weinerPol	6	-10	81	-17	52	86	-93	98	19	92	87	98	95	94	100	85

Fig. S48: Correlation matrix of the calculated descriptors.

Tab. S2: The experimental and predicted activities (LogMIC), residuals and Z-Scores for the tested compounds calculated using normal validation $PRED, $RES and $Z-SCORE and the corresponding cross-validation properties, $XPRED, $XRES and $XZ-SCORE.

	...	ID	LogMIC	MIC	$PRED	$RES	$Z-SCORE	$XPRED	$XRES	$XZ-SCORE
1		4a	0.3440	2.2100	0.4118	-0.0678	0.5112	0.4247	-0.0807	0.6050
2		4c	0.3110	2.0500	0.3931	-0.0821	0.6189	0.4027	-0.0917	0.6885
3		4f	0.3150	2.0700	0.4677	-0.1527	1.1514	0.4747	-0.1597	1.2083
4		16	0.3030	2.0100	0.5024	-0.1994	1.5040	0.6179	-0.3149	2.4210
5		7a	0.9530	8.9900	0.8290	0.1240	0.9350	0.6828	0.2702	2.1044
6		2	0.4010	2.5200	0.3685	0.0325	0.2451	0.3647	0.0363	0.2714
7		4d	0.3110	2.0500	0.5300	-0.2190	1.6521	0.5710	-0.2600	1.9946
8		3a	0.6930	4.9900	0.6759	0.0171	0.1288	0.6720	0.0210	0.1553
9		5b	1.7900	62.8000	1.8007	-0.0107	0.0804	5.3000	-3.5100	92.1262
10		4j	0.3050	2.0200	0.4144	-0.1094	0.8251	0.4199	-0.1149	0.8650
11		6a	0.2940	1.9700	0.4369	-0.1429	1.0779	0.4693	-0.1753	1.3260
12		3c	0.3380	2.1800	0.6046	-0.2666	2.0107	0.6406	-0.3026	2.3501
13		4l	0.9450	8.8200	0.7684	0.1766	1.3323	0.6462	0.2988	2.4230
14		4h	0.6040	4.0200	0.6567	-0.0527	0.3975	0.6697	-0.0657	0.4894
15		3b	0.3760	2.3800	0.4645	-0.0885	0.6675	0.4845	-0.1085	0.8149
16		5a	0.3360	2.1700	0.4088	-0.0728	0.5492	0.4176	-0.0816	0.6119
17		4g	0.3150	2.0700	0.4790	-0.1640	1.2369	0.4862	-0.1712	1.2971
18		4e	0.3150	2.0700	0.5286	-0.2136	1.6114	0.5375	-0.2225	1.7000
19		18a	0.1900	1.5500	0.7749	-0.5849	4.4111	0.8875	-0.6975	6.0688
20		8a	0.3670	2.3300	0.2338	0.1332	1.0045	0.2002	0.1668	1.2605
21		12a	0.4740	2.9800	0.7220	-0.2480	1.8705	0.9441	-0.4701	3.7853
22		13a	1.0660	11.6600	0.8625	0.2035	1.5348	0.4067	0.6593	7.8850
23		4k	0.3090	2.0400	0.1930	0.1160	0.8751	0.1653	0.1437	1.0831
24		4i	0.3260	2.1200	0.0969	0.2291	1.7280	0.0037	0.3223	2.4930

Tab. S5: Abbreviations of validation parameters.

**Value**	**Field Name**	**Description**
Model Prediction	$PRED	Value of the model
Model Residual	$RES	Difference between the value of the model and the activity field
Model Z-Score	$Z-SCORE	Absolute difference between the value of the model and the activity field, divided by the square root of the mean square error of the data set
Cross Prediction	$XPRED	Value of the model under a leave-one-out cross validation scheme
Cross Residual	$XRES	Difference between the value of the model under a leave-one-out cross validation scheme and the activity field
Cross Z-Score	$XZ-SCORE	Absolute difference between the value of the model under a leave-one-out cross validation scheme and the activity field, divided by the square root of the mean square error of the data set

Tab. S6: Experimental and predicted LogMIC of test set compounds.

Compound	Ex LogMIC	Pred LogMIC
7b	0.65	0.7473
4b	0.274	0.4221
6b	0.834	0.7352
8b	0.324	0.5216
14	1.17	0.8197
18c	0.8	0.9322
18b	0.19	0.3129
13b	0.463	0.6732
13c	1.07	1.0374

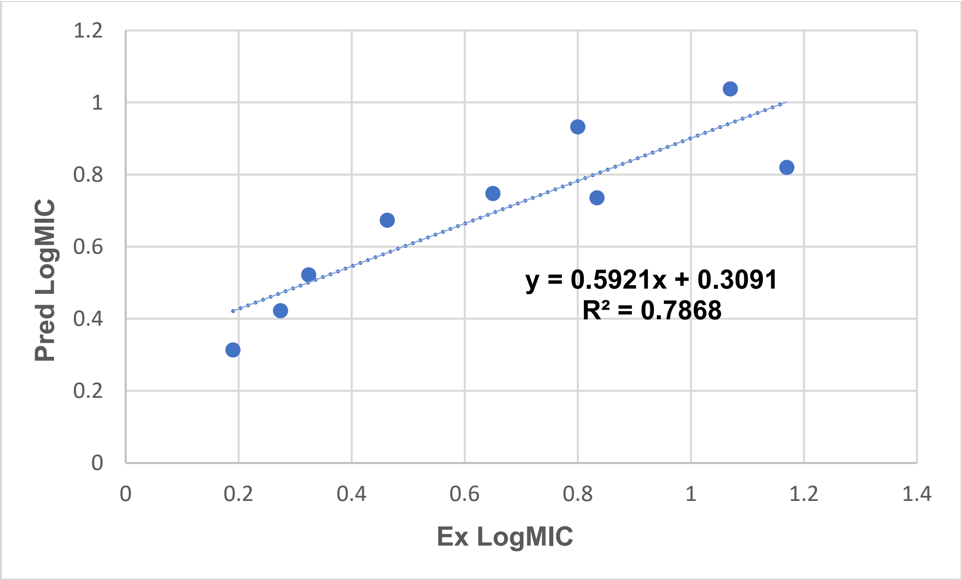

Fig. S49. Correlation between Exp Log MIC and Pred Log MIC of validation test set.

4. Prediction of physicochemical properties

Text S4: Prediction of physicochemical properties

The compliance of the newly synthesized compounds to Lipinski's and Veber's rules of oral bioavailability was estimated using MOE 2020.01. According to Lipinski's rule of five, a compound with a molecular mass under 500 Dalton (MW), a coefficient of partition between octanol and water (LogP(o/w)) lower than 5, no more than five hydrogen bond donors (lip_don) and no more than 10 hydrogen bond acceptors (lip_acc) could be a good drug candidate. Veber's rule states that a compound with 10 or fewer rotatable bonds (b_rotN) and a polar surface area (TPSA) no greater than 140 Å2 should present good oral bioavailability.

The results (Tab. S7) showed that except for 2-21 most of the tested derivatives are in accordance with Lipinski's and Veber's rules with high probability of reasonable oral absorption.

Tab. S7: Physicochemical properties of norfloxacin and designed compounds.

Code	Lip_acc	Lip_ don	LogP (O/W)	LogS	TPSA	Weight	Lip_dr uglike	Lip_ Violation	b_rot N	Veber_vi olation
**Nor**	6	2	0.7250	-2.5094	72.879	319.33	1	0	3	No
**2**	7	1	0.9940	-3.5743	81.160	395.81	1	0	5	No
**3a**	8	2	1.0039	-3.1049	93.190	418.46	1	0	8	No
**3b**	8	2	0.3899	-2.9032	93.190	404.44	1	0	7	No
**4a**	8	2	1.7029	-4.3998	93.190	452.48	1	0	7	No
**4b**	8	2	2.5009	-5.4902	93.190	531.38	1	1	7	No
**4c**	8	2	2.2950	-5.1341	93.190	486.93	1	0	7	No
**4d**	11	2	1.6380	-5.190	139.0	497.48	1	1	8	No
**4e**	9	2	1.6569	-4.4502	102.41	482.51	1	0	8	No
**4f**	9	2	1.659	-4.4502	102.41	482.51	1	0	8	No
**4g**	9	2	1.6959	-4.4502	102.41	482.51	1	0	8	No
**4h**	10	3	1.38	-4.3692	130.49	496.49	1	0	8	No
**4i**	8	2	1.8559	-4.6948	93.190	470.47	1	0	7	No
**4j**	9	2	1.5559	-4.7121	110.26	494.52	1	0	8	No
**4k**	8	2	2.0439	-4.9898	93.190	488.46	1	0	7	No
**4l**	9	2	0.4709	-3.1417	106.08	453.47	1	0	7	No
**3c**	8	2	2.0280	-4.0471	93.190	458.53	1	0	7	No
**5a**	9	2	0.6840	-3.8712	106.08	459.50	1	0	7	No
**5b**	9	2	2.3299	-5.6867	106.08	509.56	1	1	7	No
**16**	11	3	0.0199	-3.1886	135.17	496.49	1	1	9	No
**6a**	10	1	0.7910	-5.1532	118.54	506.48	1	1	6	No
**6b**	10	1	1.6260	-6.2436	118.54	585.38	1	1	6	No
**7a**	8	1	1.2910	-3.4611	84.40	444.50	1	0	6	No
**7b**	9	1	-0.116	-2.9984	93.629	446.47	1	0	6	No
**8a**	9	1	0.182	-3.1016	98.98	427.43	1	0	6	No
**8b**	13	1	-0.776	-4.4878	157.69	473.42	1	1	7	Yes
**14**	10	2	1.9889	-6.4578	122.62	537.57	1	1	6	No
**20a**	9	1	1.644	-4.985	101.47	296.47	1	0	5	No
**20b**	9	1	1.789	-5.1639	101.47	492.5	1	0	5	No
**20c**	10	1	1.447	-4.7404	110.69	508.5	1	1	6	No
**21**	10	1	2.4219	-4.9873	124.76	468.46	1	0	7	No
**18a**	14	3	2.8190	-7.1617	180.47	643.631	0	3	12	Yes

**18b**	14	3	2.85599	-7.1617	180.47	643.631	0	3	12	Yes
**18c**	11	3	3.4760	-7.1058	134.64	633.079	0	2	11	Yes
**12a**	14	1	6.29099	-11.943	170.05	760.807	0	4	12	Yes
**12b**	14	1	6.32800	-11.943	170.05	760.807	0	4	12	Yes
**13a**	14	1	4.4720	-10.908	178.25	685.692	0	3	11	Yes
**13b**	14	1	4.50899	-10.908	178.25	685.692	0	3	11	Yes
**13c**	11	1	5.1290	-10.852	132.44	675.14	0	3	10	Yes

5. *in silico* prediction of ADME/Tox

Text S5: *In silico* prediction of ADME/Tox

The *in silico* prediction of absorption, distribution, metabolism, and excretion (ADME) and toxicity (Tox) profiles is a helpful tool to predict the pharmacokinetic and toxicological properties of drug candidates, especially in pre-clinical stages. To improve ADME/Tox predictions, *in silico* and online models have been established. These models are useful to guide drug optimization and can aide in avoiding late-stage failures. Therefore, they can save time and money in the drug development process [7]. Two ADME/Tox web tools were used in this study: the freely accessible Swiss-ADME web tool (http://www.swissadme.ch/) [8], which represents a recent and relevant computational method to evaluate the pharmacokinetics profile of small molecules, and the freely accessible pkCSM pharmacokinetics web tool (http://structure.bioc.cam. ac.uk/pkcsm) [9]. All studied compounds showed good pharmacokinetic profiles, high gastrointestinal (GI) absorption (similar to norfloxacin which is already marketed for oral administration), and good bioavailability, which complied with the results obtained from MOE-predicted physicochemical properties and Lipinski's rule of five that discussed before.

Text S6: ADME/Tox prediction using pKCSM lab

pkCSM is a platform for the analysis and optimization of pharmacokinetic and toxicity properties implemented in a user-friendly, freely available web interface and constitutes a valuable tool to help medicinal chemists find the balance between potency, safety, and pharmacokinetic properties. Previous experiments showed that pkCSM performs as well as or better than several other widely used methods [10].

Caco-2 permeability (Log Papp in 10^{-6} cm/s)

The Caco-2 cell line is derived from human colon carcinoma. These cells share characteristics with intestinal epithelial cells such as the formation of a polarized monolayer [11]. The Caco-2 permeability assay measures the rate of flux of a compound across polarized Caco-2 cell monolayers. The data generated from this assay can be used to predict the *in vivo* absorption of drugs. A Log Papp ≤ 10^{-6} cm/s indicates low intestinal absorption (0-20%), a Log Papp 10^{-6}-10 x 10^{-6} cm/s indicates intermediate intestinal absorption (20-70%), and a Log Papp > 10 x 10^{-6} cm/s indicates high intestinal absorption (70-100%) [12]. According to this classification, most of our newly synthesized compounds showed intermediate intestinal absorption (**Tab. S8**).

Steady state volume of distribution (VDss)

The steady state volume of distribution (Vss) reflects the actual blood and tissue volume, into which a drug is distributed, and the relative binding of the drug to protein in these spaces [13]. A drug with a high Vd has a propensity to leave the plasma and enter the extravascular compartments of the body, meaning that a higher dose is required to achieve a given plasma concentration (high Vd -> more distribution to other tissues). Conversely, a drug with a low Vd has a propensity to remain in the plasma, meaning a lower is required to achieve a given plasma concentration (low Vd -> less distribution to other tissue) [14]. Most of our compounds show a good volume of distribution and moderate plasma protein binding compared to norfloxacin (**Tab. S8**).

Blood Brain Barrier permeability (LogBB)

The most common parameter used to quantify penetration of a compound across the blood-brain barrier (BBB) is the ratio of the concentration of compound measured in the brain to the concentration of compound measured in the blood at steady state. This ratio is expressed as logBB (log[brain]/[blood]) and determines the total extent of brain exposure, at a steady state. Values of logBB can be used to determine, if the compound is BBB+ (crosses the BBB) or BBB− (does not

cross the BBB) [15]. All our compounds have lower logBB values than norfloxacin, which indicates that they have a low probability for crossing the BBB, suggesting that they may have less effects on the central nervous system and thus no or lower neurotoxic side effects than norfloxacin (**Tab. S8**).

Metabolism

The predictions did not find any metabolizing enzyme for norfloxacin and compound **8a**. For all other compounds, it is predicted that they are metabolized by the enzymes CYP3A4 and CYP2D6 (**Tab. S8**).

Total body clearance

Total body clearance describes the volume of plasma, from which a drug would be totally removed per unit of time. Clearance is a measure of the body's ability to remove a drug by either metabolism or excretion. It is the parameter that determines the total systemic exposure to a drug, which is defined as the ratio of dose/clearance. Total body clearance is the sum of all processes, by which drugs are removed from the body or inactivated, primarily renal excretion and metabolism [16, 17]. The primary application of clearance as pharmacokinetic parameter is dose adjustment. A low clearance indicates a high systemic exposure, and a high clearance indicates low systemic exposure. Thus, adverse drug effects, which can be related to overexposure, would be expected more often in patients with low clearance [17]. All our compounds have good total clearance values compared to norfloxacin, which indicates good systemic exposure and promises lower or no adverse effects (**Tab. S8**).

Toxicity

Toxicity of our newly synthesized compounds was predicted by calculation oral rat acute toxicity (LD_{50}), oral rat chronic toxicity, hepatotoxicity, and Ames toxicity.

Oral rat acute toxicity (LD_{50})

Norfloxacin has predicted to have a median lethal dose (LD_{50}) of 2.139 mol/kg in an oral acute toxicity rat model. All our derivatives have higher predicted LD_{50} values, indicating that their structural modifications may reduce their toxicity (**Tab. S8**). Thus, they hold the promise to be safer alternatives to norfloxacin.

Oral rat chronic toxicity

In an oral chronic toxicity rat model, it was predicted that norfloxacin induces chronic toxicity at 1.153 mg per kg body weight per day (mg/kg_bw/day). Most of our derivatives are predicted to induce chronic toxicity at comparable or higher doses than norfloxacin, again suggesting that several of them may constitute less toxic alternatives (**Tab. S8**).

Hepatotoxicity

Norfloxacin, like other fluoroquinolones, is associated with a low rate (1% to 3%) of serum enzyme elevations during therapy. These abnormalities are generally mild, asymptomatic, and transient. Norfloxacin has also been linked to rare but occasionally severe and even fatal cases of acute liver injury [18]. Both norfloxacin and our new compounds were predicted to induce hepatotoxicity, but predictions show a lower LD_{50} for norfloxacin than for the derivatives, suggesting that they may be tolerated better than their parent compound (**Tab. S8**).

Ames toxicity

The Ames test is a method to assess the potential carcinogenic effect of chemicals by using the bacterial strain *Salmonella typhimurium*. This strain is histidine-auxotrophic and thus is unable to grow in medium lacking histidine. When the auxotrophic strains is exposed to a mutagen, it has a higher chance of reverting back to the wild type phenotype, enabling it to grow on minimal medium without histidine [19]. The only compound predicted to have mutagenic properties is 2 (**Tab. S8**).

Tab. S8: Predicted ADME/TOX properties by pKCSM.

Cmp	Caco2 permeability (log Papp in 10^{-6} cm/s)	Steady state volume of distribution VDss (log L/kg) (human)	Fraction unbound (human) (Fu)	BBB permeability (log BB)	Total Clearance (log ml/min/kg)	Oral Rat Acute Toxicity (LD50) (mol/kg)	Oral Rat Chronic Toxicity (LOAEL) (log mg/kg_bw/ day)	Hepatotoxicity	Ames toxicity	Metabolizing enzyme
Nor	0.363	-0.201	0.478	-.0559	0.356	2.139	1.153	Yes	No	No predicted
2	1.252	-0.925	0.254	-0.79	0.361	2.22	1.102	Yes	Yes	CYP2D6
4a	0.696	-0.877	0.062	-1.246	0.441	2.396	1.525	Yes	No	CYP2D6 CYP3A4
4b	0.592	-0.863	0.068	-1.433	0.129	2.44	1.439	Yes	No	CYP2D6 CYP3A4 CYP1A2
4c	0.589	-0.877	0.072	-1.422	0.15	2.435	1.456	Yes	No	CYP2D6 CYP3A4
4d	-0.285	-0.98	0.026	-1.459	0.435	2.272	2.099	Yes	No	CYP2D6 CYP3A4
4e	0.664	-0.921	0.065	-1.446	0.335	2.412	1.28	Yes	No	CYP2D6 CYP3A4
4f	0.613	-0.695	0.084	-1.451	0.475	2.52	1.318	Yes	No	CYP2D6 CYP3A4
4g	0.606	-0.798	0.068	-1.435	0.467	2.518	1.378	Yes	No	CYP2D6 CYP3A4
4k	0.638	-0.838	0.092	-1.664	0.312	2.493	1.689	Yes	No	CYP2D6 CYP3A4
4h	0.552	-1.07	0.134	-1.654	0.503	2.606	1.872	Yes	No	CYP2D6
4l	0.525	-1.052	0.19	-1.523	0.47	2.602	0.695	Yes	No	CYP2D6 CYP3A4
7a	1.222	-0.044	0.36	-1.242	0.343	2.22	1.065	Yes	No	CYP2D6 CYP3A4
7b	0.355	-0.278	0.405	-1.314	0.388	2.114	1.158	Yes	No	CYP2D6 CYP3A4
3b	0.24	-0.148	0.363	-1.298	0.435	2.11	1.434	Yes	No	CYP2D6 CYP3A4
3a	0.235	-0.252	0.408	-1.26	0.365	2.078	1.439	Yes	No	CYP2D6
16	-0.115	-1.424	0.158	-1.791	0.708	2.583	2.166	Yes	No	CYP2D6 CYP3A4
6a	0.187	-1.046	0.045	-0.874	0.061	2.663	1.967	Yes	No	CYP3A4
6b	0.589	-0.995	0.062	-1.051	0.145	2.694	1.892	Yes	No	CYP3A4
5a	0.559	-1.075	0.172	-1.651	0.379	2.636	0.997	Yes	No	CYP3A4
5b	0.697	-0.811	0.029	-1.618	0.41	2.497	1.153	Yes	No	CYP2D6

										CYP3A4
4i	0.639	-0.788	0.095	-1.461	0.369	2.484	1.339	Yes	No	CYP2D6<br>CYP3A4
4j	0.597	-0.792	0.069	-1.458	0.45	2.472	1.229	Yes	No	CYP2D6<br>CYP3A4
8b	-0.212	-1.57	0.167	-1.583	0.254	1.857	1.763	Yes	No	CYP3A4
14	0.771	-0.547	0.017	-1.735	0.354	2.303	2.152	Yes	No	CYP2D6<br>CYP3A4
3c	0.615	-0.002	0.339	-1.314	0.483	2.203	0.914	Yes	No	CYP2D6<br>CYP3A4
8a	0.318	-0.579	0.39	-1.234	0.304	2.459	0.455	Yes	No	Not predicted
20a	0.654	-0.062	0.316	-1.117	0.405	2.292	2.671	Yes	No	CYP3A4
20b	0.595	-0.028	0.304	-0.909	0.467	2.297	2.62	Yes	No	CYP3A4
20c	0.619	0	0.31	-2.993	0.48	2.254	2.66	Yes	No	CYP3A4
21	0.524	-.354	0.103	-1.348	0.2	2.389	2.062	Yes	No	CYP2D6<br>CYP3A4
18a	-0.421	-1.34	0.019	-1.831	0.637	2.396	2.687	Yes	No	CYP2D6<br>CYP3A4
18b	-0.455	-1.332	0.029	-1.825	0.379	2.382	2.693	Yes	No	CYP2D6<br>CYP3A4
18c	0.372	-1.149	0.005	-1.798	-0.063	2.472	1.988	Yes	No	CYP2D6<br>CYP3A4
12a	-0.388	-1.451	0.399	-1.731	0.161	2.753	2.314	Yes	No	CYP3A4
12b	-0.422	-1.455	0.404	-1.725	0.28	2.751	2.331	Yes	No	CYP3A4
13a	-0.264	-1.123	0.204	-1.821	0.205	2.674	2.134	Yes	No	CYP3A4
13b	-0.298	-1.143	0.217	-1.816	0.325	2.664	2.151	Yes	No	CYP3A4
13c	0.558	-0.653	0.181	-1.527	0.168	2.747	1.899	Yes	No	CYP3A4

Text S7: ADME prediction by SwissADME

Further investigation of ADME properties for the most active compounds was done using the SwissADME web tool [8]. The predicted values are shown in **Tab. S9**. All studied compounds have high predicted gastrointestinal (GI) absorption values and are thus estimated to have good absorption in the human gut (similar to norfloxacin, which is already marketed for oral administration). They also showed a good bioavailability, which complied with results obtained from MOE-predicted physicochemical properties and Lipinski's rule of five (**Text S3**).

The polar surface area (TPSA) is linked to drug bioavailability. A TPSA value equal to or less than 140 Å2 will have a high probability of good oral bioavailability in a rat model [20]. The TPSA values for the tested compounds ranged from 94.88 to 205.25Å2, thus most of them are predicted to show high passive oral absorption.

The compounds showed aqueous solubility level between -1.29 and -8.22, indicating good to moderate solubility in water. Lipophilicity was determined using the logarithm of the n-octanol/water partition coefficient, which was predicted using the Consensus LogPo/w descriptor of SwissADME. The LogPo/w is closely related to transport processes, including membrane permeability and penetration, which directly affects the ability of the drug to reach its target site [21]. The tested compounds had LogPo/w values ranging from 1.79 to 4.55. Thus, they are predicted to display good cell permeability. Tissue penetration was predicted to be moderate according to the general guide for good oral bioavailability ($0 < \log P < 3$) [22]. Most of the investigated compounds have a good bioavailability score ranging from 0.55 to 0.56, except for compounds **32a**, **33a**, and **34b**, which had bioavailability score value of 0.11. Distribution of all evaluated compounds was predicted using the glycoprotein P (P-gp) substrate.

Tab. S9: ADME properties predicted by SwissADME.

Cmpd	Consensus Log $P_{o/w}$	TPSA (Å²)	Log S (ESOL)	Water solubility class	GI absorption	BBB permeant	Bioavailability Score
Nor	0.98	74.57	-1.29	Very soluble	High	No	0.55
4a	2.26	94.88	-4.36	Moderately soluble	High	No	0.56
4b	2.89	94.88	-5.28	Moderately soluble	High	No	0.56
4e	2.41	104.11	-4.44	Moderately soluble	High	No	0.56
4g	2.44	104.11	-4.44	Moderately soluble	High	No	0.56
3c	1.86	94.88	-2.64	Soluble	High	No	0.56
20a	2.07	103.16	-3.64	Soluble	High	No	0.55
21	2.42	123.63	-3.82	Soluble	High	N0	0.55
18a	1.79	182.16	-3.88	Soluble	High	No	0.11
12a	4.55	197.04	-8.22	Poorly soluble	Low	No	0.11
13b	3.56	205.25	-6.95	Poorly soluble	Low	No	0.11

6. Molecular modeling

Text S8: Molecular docking

Docking on S. aureus DNA gyrase

Docking studies of the active compounds from each series were performed based on the three-dimensional structure and conformations of the crystal structure of moxifloxacin in complex with *S. aureus* DNA gyrase and DNA (PDB code 5cdq)[23], using the commercially available MOE 2020.01 software. The docking protocol was validated by re-docking of the co-crystalized moxifloxacin at the active site of topoisomerase IV (**Fig. S50**). The re-docking rmsd was 0.6010 Å and the binding score was -10.76 Kcal/mol. The validated docking setup was then used to investigate the ligand-receptor interactions for norfloxacin (score = -9.54 Kcal/mol)**.** The main binding interactions of norfloxacin were a coordination bond with Mg^{2+} metal, H-bonding between the carbonyl of the carboxylic acid group and Ser B84, a π-hydrogen bond with DA E2013 through the piperazine ring (deoxyadenine), and π-π stacking between the quinolone ring and DG D2009 (deoxyguanine) (**Fig. S51**). Molecular docking of compounds **4e, 6a, 12b,** and **16** are shown in **Fig. S52-55**.

Docking on A. baumannii DNA topoisomerase IV

The docking protocol was validated by re-docking of the co-crystalized moxifloxacin at the active site of topoisomerase IV, as shown in **Fig. S55.** The re-docking rmsd was 0.3718 Å and the binding score was -10.74 Kcal/mol. The validated docking setup was then used to investigate the ligand-receptor interactions for norfloxacin (score = -9.32 Kcal/mol)**.** The main interactions of norfloxacin were a coordination bond with Mg^{2+} metal, H-bonding between the carbonyl of the carboxylic acid group and Arg1123, a π-hydrogen bond with DA C20 (Deoxyadenine), and π-π stacking between quinolone ring and DA D16. Molecular docking of compounds **4a, 4e, 8a,** and **18a** are shown in **Fig. S56-59**. **Tab. S10** shows an overview of all results.

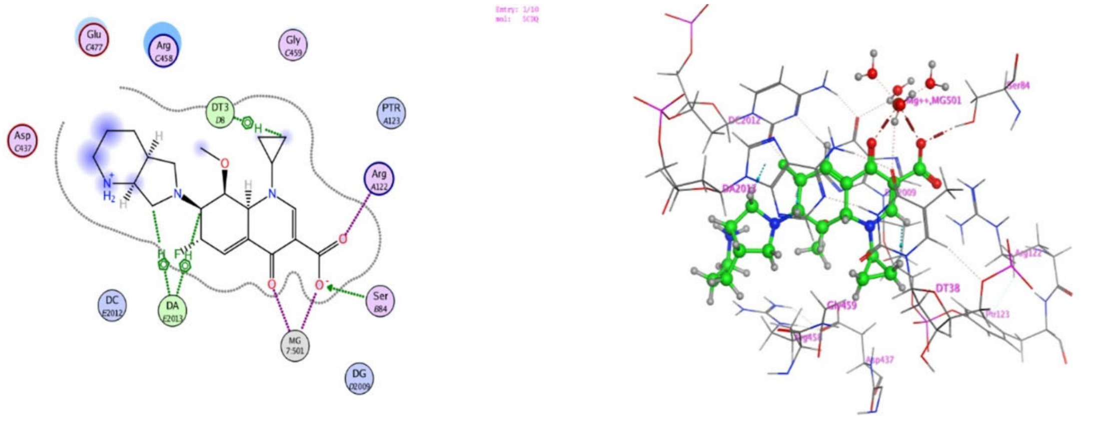

Fig. S50: 2D and 3D interactions of co-crystallized ligand moxifloxacin with DNA gyrase (PDB ID: 5CDQ).

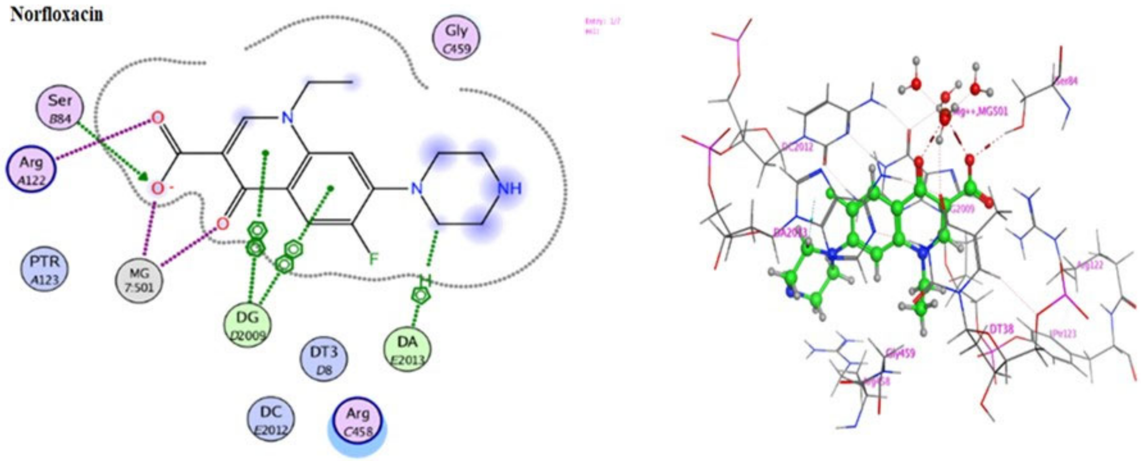

Fig. S51: 2D and 3D interactions of norfloxacin with DNA gyrase (PDB ID: 5CDQ).

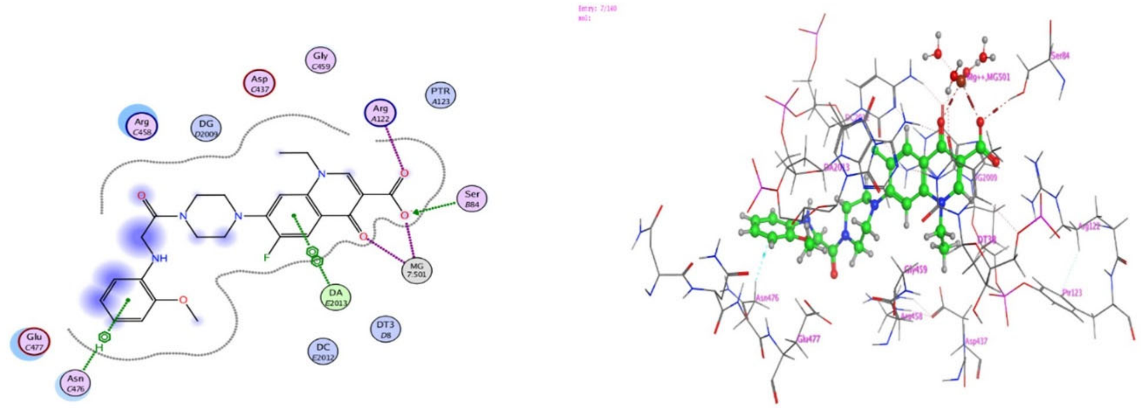

Fig. S52: 2D and 3D interactions of compound **4e** with DNA gyrase (PDB ID: 5CDQ).

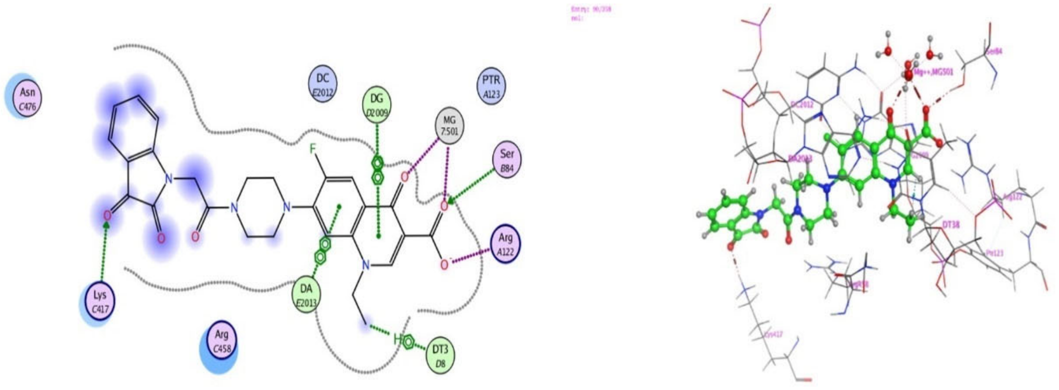

Fig. S53: 2D and 3D interactions of compound **6a** with DNA gyrase (PDB ID: 5CDQ).

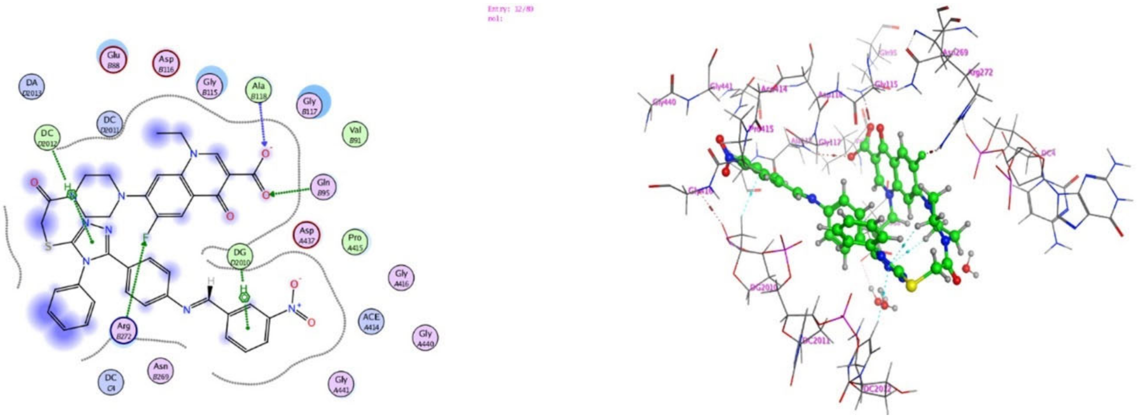

Fig. S54: 2D and 3D interactions of compound **12b** with DNA gyrase (PDB ID: 5CDQ).

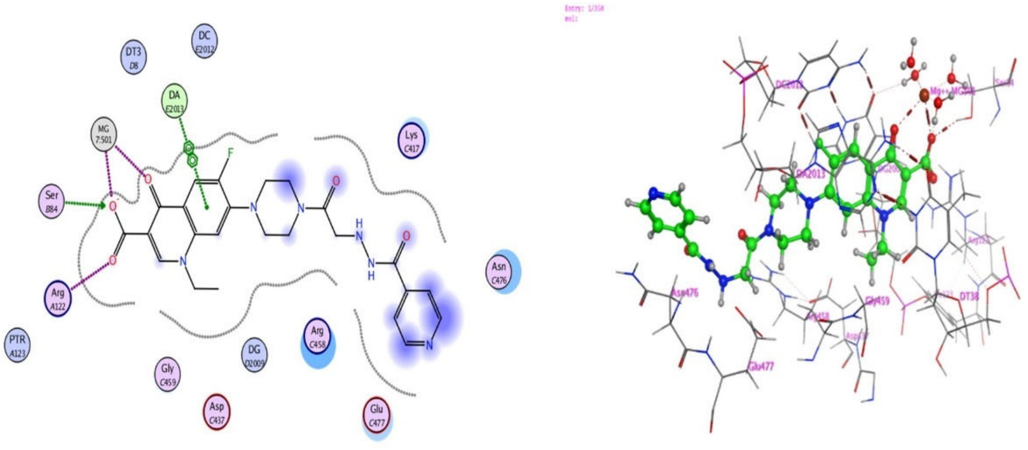

Fig. S55: 2D and 3D interactions of compound **16** with DNA gyrase (PDB ID: 5CDQ).

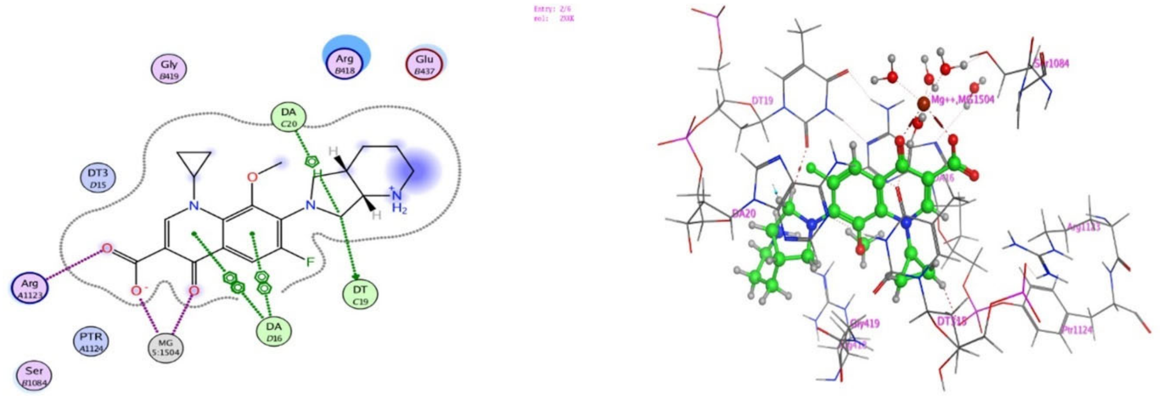

Fig. S56: 2D and 3D interactions of moxifloxacin with DNA topoisomerase IV (PDB ID: 2XKK).

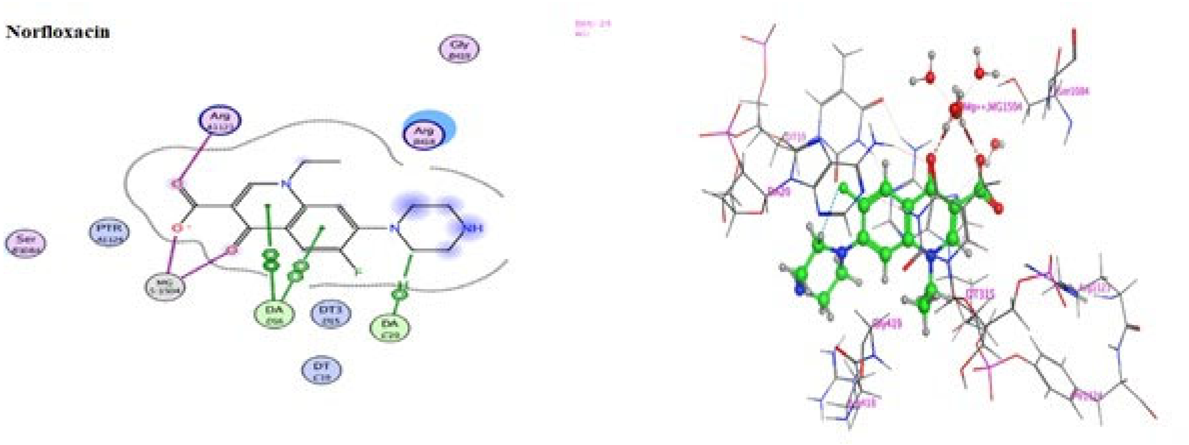

Fig. S57: 2D and 3D interactions of norfloxacin with DNA topoisomerase IV (PDB ID: 2XKK).

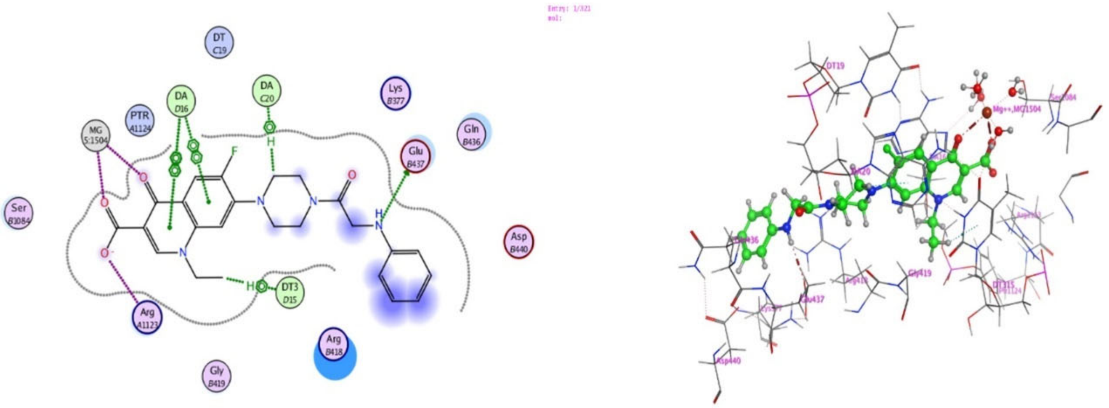

Fig. S58: 2D and 3D interactions of compound 4a with DNA topoisomerase IV (PDB ID: 2XKK).

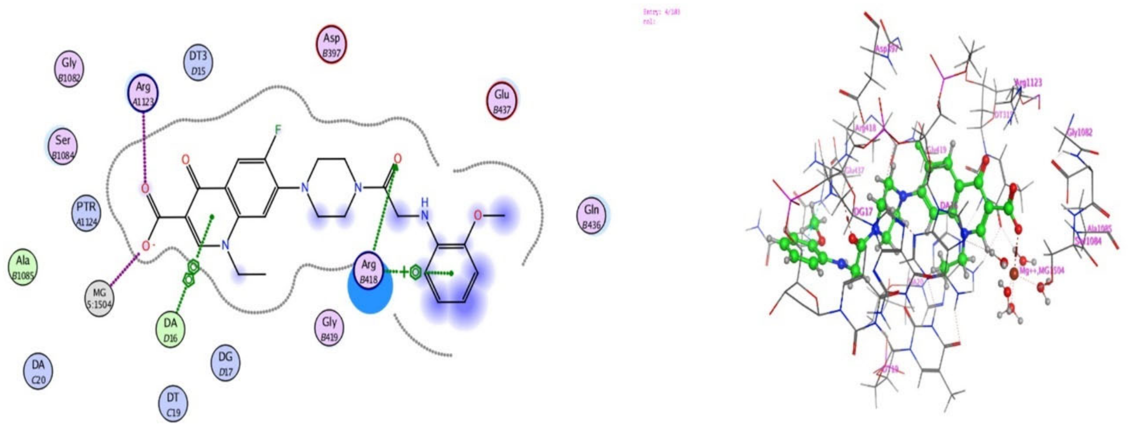

Fig. S59: 2D and 3D interactions of compound 4e with DNA topoisomerase IV (PDB ID: 2XKK).

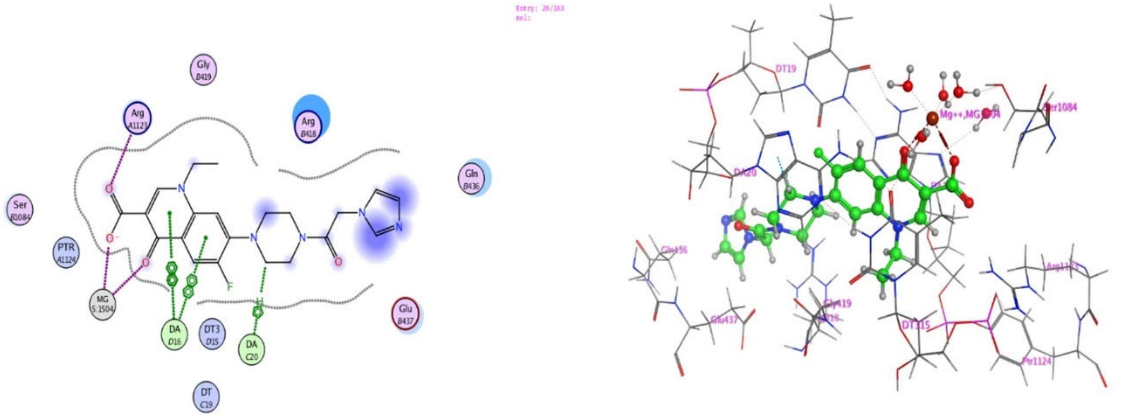

Fig. S60: 2D and 3D interactions of compound 8a with DNA topoisomerase IV (PDB ID: 2XKK).

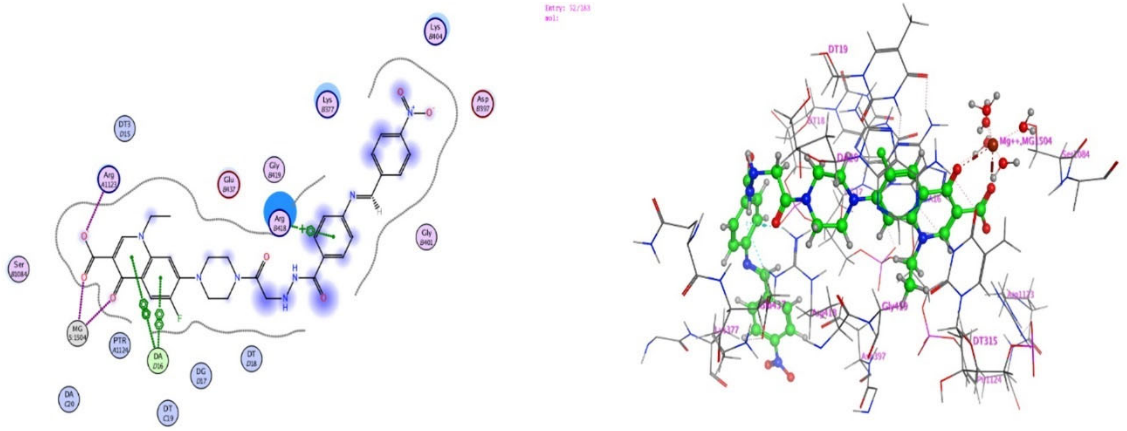

Fig. S61: 2D and 3D interactions of compound 18a with DNA topoisomerase IV (PDB ID: 2XKK).

Tab. S10: Interaction energies (Kcal/mol) and MIC (μM) of the tested compounds with DNA-gyrase/ topoisomerase-IV.

DNA gyrase (5cdq)			DNA topoisomerase IV (2xkk)		
Code	ΔG	MIC	Code	ΔG	MIC
**4b**	-12.21	1.88	**4a**	-11.03	8.84
**4e**	-11.88	2.07	**4e**	-11.12	8.29
**16**	-11.28	2.01	**8a**	-11.61	4.67
**6a**	-12.06	1.97	**18a**	-11.58	6.21
**18a**	-12.76	1.55	**12a**	-11.96	3.72
**12b**	-12.63	1.49	**21**	-11.89	0.266
**21**	-9.71	2.13			

7. Mode of action studies

DNA Gyrase

Code	IC50	con
4a		10
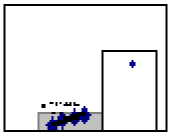		1
		0.
EC		

code	IC50	conc.	log	%inh
Nor		100	2	90.2
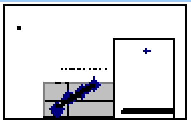		10	1	73.6
		1	0	50.1
		0.1	-1	20.4
EC				0

Code	IC50	conc.	Log	%inh
4e		100	2	92.7
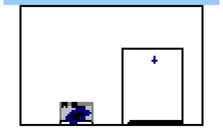		10	1	80.4
		1	0	62.2
		0.1	-1	34.6
EC				0

Code	IC50	conc.	Log	%inh
21		100	2	92.3
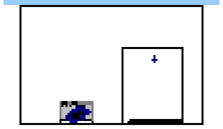		10	1	77.9
		1	0	57.6
		0.1	-1	28.4
EC				0

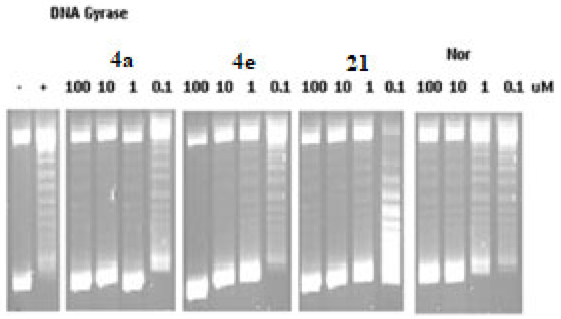

Fig. S62: %Inhibition of investigated compounds and norfloxacin on *E. coli* DNA gyrase.

TOPO IV

Code	IC50	conc.	Log	%inh
4b		100	2	87
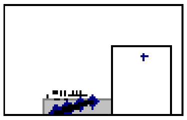		10	1	70.1
		1	0	43.3
		0.1	-1	20.6
EC				0

Code	IC50	conc.	Log	%inh
21		100	2	78
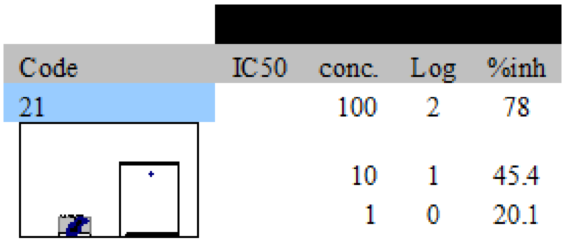		10	1	45.4
		1	0	20.1
		0.1	-1	5.47
EC				0

Code	IC50	conc.	Log	%inh
4e		100	2	78.4
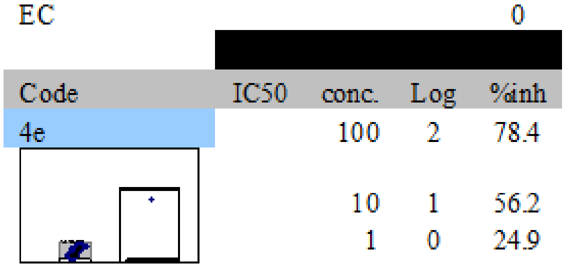		10	1	56.2
		1	0	24.9
		0.1	-1	10.1
EC				0

Code	IC50	conc.	Log	%inh
Nor		100	2	78.9
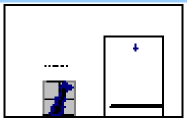		10	1	47.4
		1	0	22
		0.1	-1	8.36
EC				0

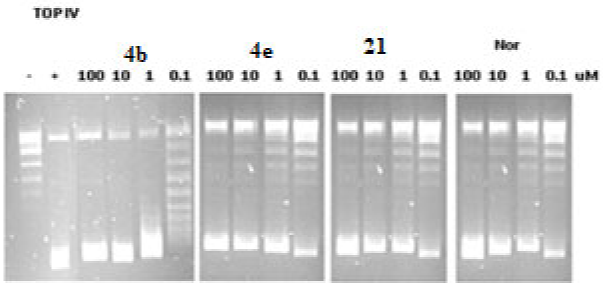

Fig. S63: % Inhibition of investigated compounds and norfloxacin on *E. coli* DNA topoisomerase IV.

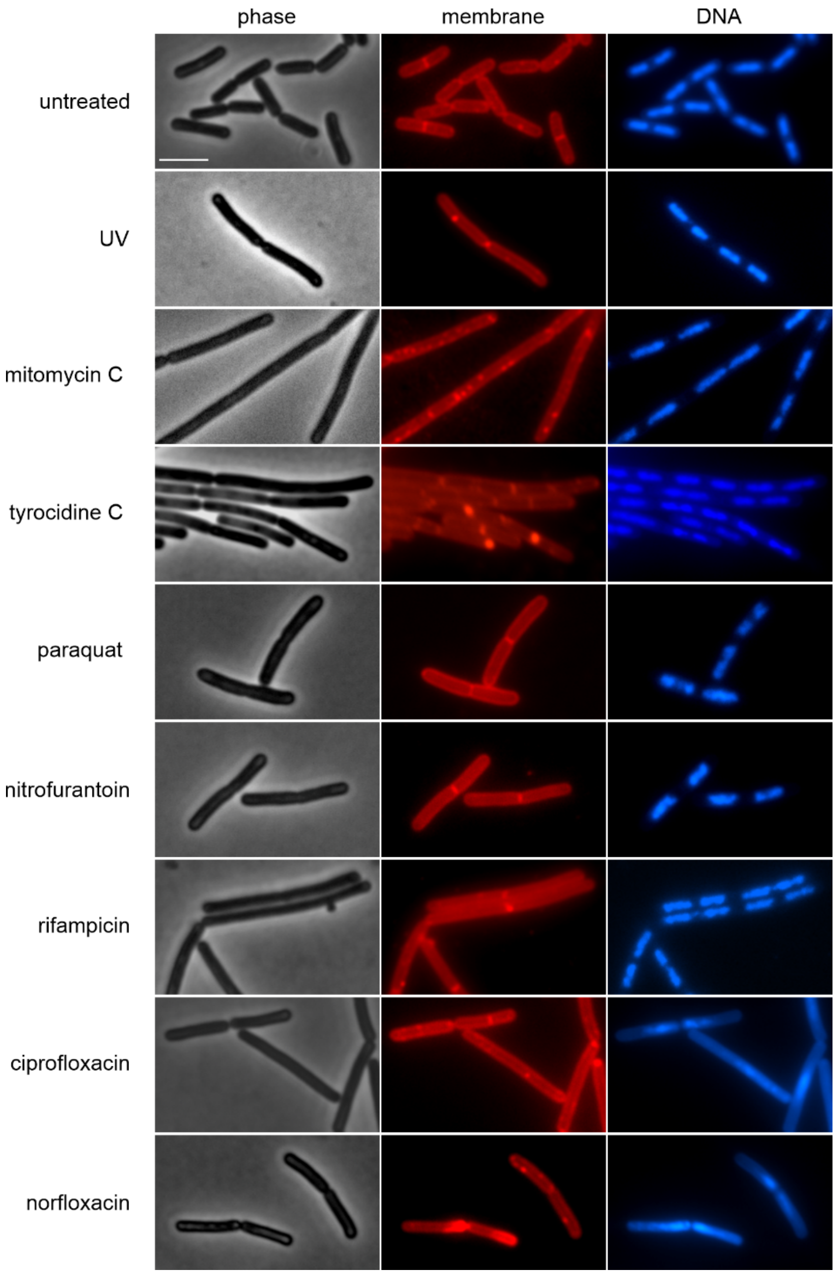

Fig. S64: Bacterial cytological profiling of *B. subtilis* DSM402 treated with antimicrobial compounds that affect DNA integrity. Cultures were grown until early log phase and subsequently treated with 0.5 µg/mL mitomycin C, 2.7 µg/mL tyrocidine C, 500 µM paraquat, 25 µM nitrofurantoin, 0.125 µg/mL rifampicin, 1 µg/mL ciprofloxacin, 6 µg/mL norfloxacin, or left untreated as control. Treatment time was 1h for all compounds except for tyrocidine C, which induces cell lysis after prolonged exposure, and was therefore applied for only 10 min. DNA condensation into oval foci localized at mid-cell or recently divided poles is highly specific for fluoroquinolones.

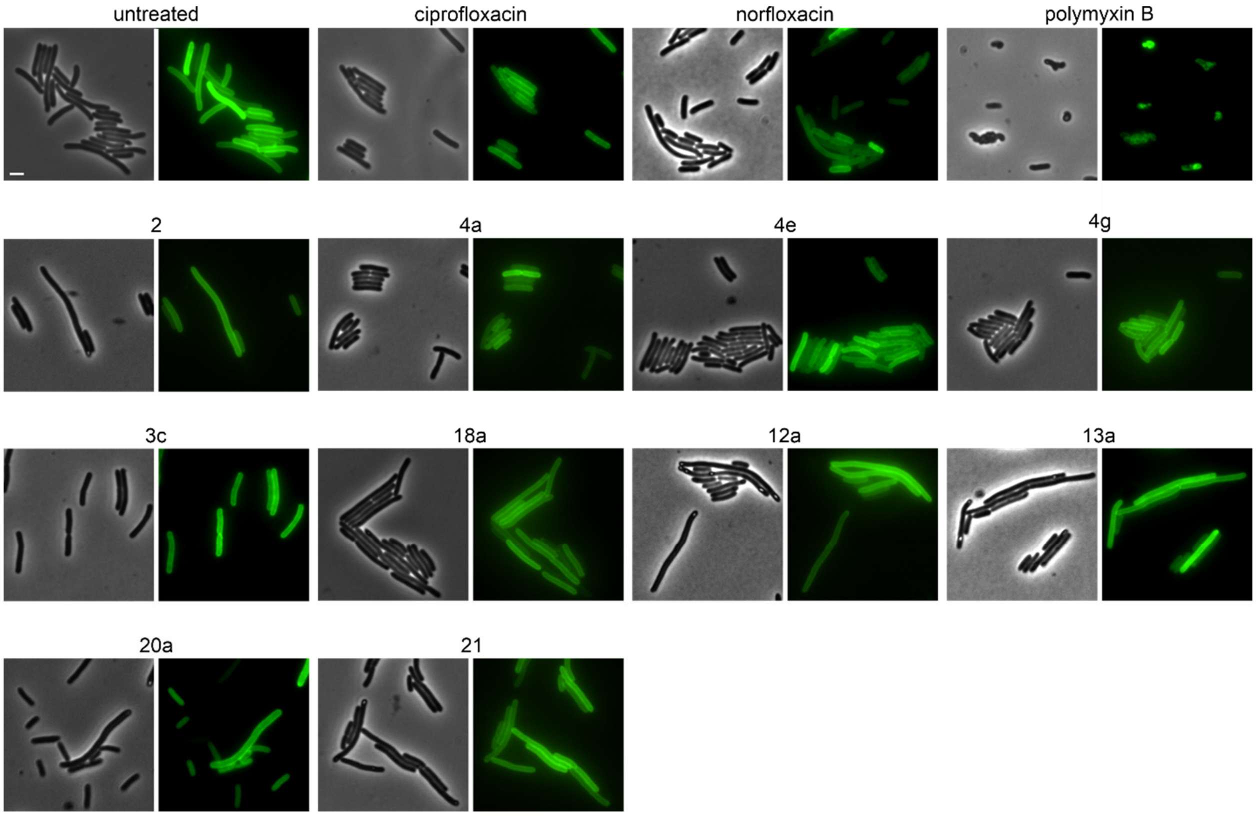

Fig. S65: Bacterial cytological profiling of *E. coli* BCB472. Cells were treated with 1x MIC of the respective compounds for 1 h prior to microscopy. Expression of NG-GlpT was induced with 10 µM IPTG for 1 h (concomitantly with antibiotic incubation). Scale bar 2 µm.

Tab. S11: Results summary of BCP in *E. coli*. Concentrations represent MIC values against *E. coli* W3110. Images were taken after 1 h of antibiotic treatment. Phase contrast images indicate cell lysis. The fluorescent membrane dye FM4-64 and the GFP-tagged membrane protein GlpT report on membrane effects. The fluorescent DNA stain DAPI reports on DNA condensation. Cip = ciprofloxacin, Nor = norfloxacin, PolB = polymyxin B.

compound	concentration (µM)	phase contrast	FM4-64	GlpT	DAPI	membrane damage	gyrase inhibition
**untreated**		dark	smooth	smooth	regular	no	no
**Cip**	0.39	dark	smooth	smooth	condensed	no	yes
**Nor**	0.37	dark	smooth	smooth	condensed	no	yes
**PolB**	0.83	dark	patchy	dispersed	dispersed	yes	no
**2**	6.31	dark	smooth	smooth	condensed	no	yes
**3c**	17.45	dark	smooth	smooth	condensed	no	yes
**4a**	8.84	dark	smooth	smooth	condensed	no	yes
**4e**	8.29	dark	smooth	smooth	condensed	no	yes
**4g**	16.58	dark	smooth	smooth	condensed	no	yes
**12a**	3.72	dark	Smooth	Smooth	condensed	no	yes
**13a**	11.66	dark	Smooth	Smooth	condensed	no	yes
**18a**	6.21	dark	Smooth	Smooth	condensed	no	yes
**20a**	3.02	dark	smooth	smooth	condensed	no	yes
**21**	0.266	dark	Smooth	Smooth	condensed	no	yes

Tab. S12: Results of checkerboard assays of norfloxacin derivatives combined with mupirocin. MIC values are given in µg/mL and represent the average of at least two replicate experiments. A FICI value below 0.5 is considered as synergistic. Mup = mupirocin, Cip = ciprofloxacin, Nor = norfloxacin, PolBN = polymyxin B nonapeptide.

compound	MIC_C	$MIC_C^{checkerboard}$	FIC_C	$MIC_M^{checkerboard}$	FIC_M	FICI	Outcome
**Mup**	64	-	-	-	-	-	
**Cip**	0.125	0.0313	0.25	2	0.0313	0.2813	synergistic
**Nor**	0.125	0.0391	0.25	32	0.5	0.8125	additive
**ACHN-975**	0.5	0.125	0.25	3	0.0469	0.2969	synergistic
**PolBN**	128	1	0.0078	1	0.0156	0.0234	synergistic
**2**	2	2.5	1.25	7.8	0.2656	1.5156	additive
**4a**	4	0.0156	0.0039	64	1	1.0039	additive
**4e**	4	0.0156	0.0039	64	1	1.0039	additive
**4g**	8	4	0.5	48	0.75	1.25	additive
**3c**	8	2.0156	0.252	36	0.5625	0.8144	additive
**7a**	16	4.0313	0.252	48	0.75	1.002	additive
**7b**	32	16	0.5	2	0.0313	0.5313	additive
**21**	0.125	0.0005	0.0039	64	1	1.004	additive
**18a**	4	0.0156	0.0039	64	1	1.004	additive
**12a**	5	0.6348	0.127	36	0.5625	0.6895	additive
**13a**	8	2.0156	0.252	48	0.75	1.002	additive

MIC_C: MIC of the test compound alone, $MIC_C^{checkerboard}$: MIC of the test compound in checkerboard assay, FIC_C: fractional inhibitory concentration of the test compound ($FIC_C = MIC_C^{checkerboard}/MIC_C$), MIC_M: MIC of mupirocin alone (64 µg/mL), $MIC_M^{checkerboard}$: MIC of mupirocin in checkerboard assay, FIC_M: fractional inhibitory concentration of the test compound ($FIC_M = MIC_M^{checkerboard}/MIC_M$), FICI: fractional inhibitory concentration index = $FIC_C + FIC_M$.

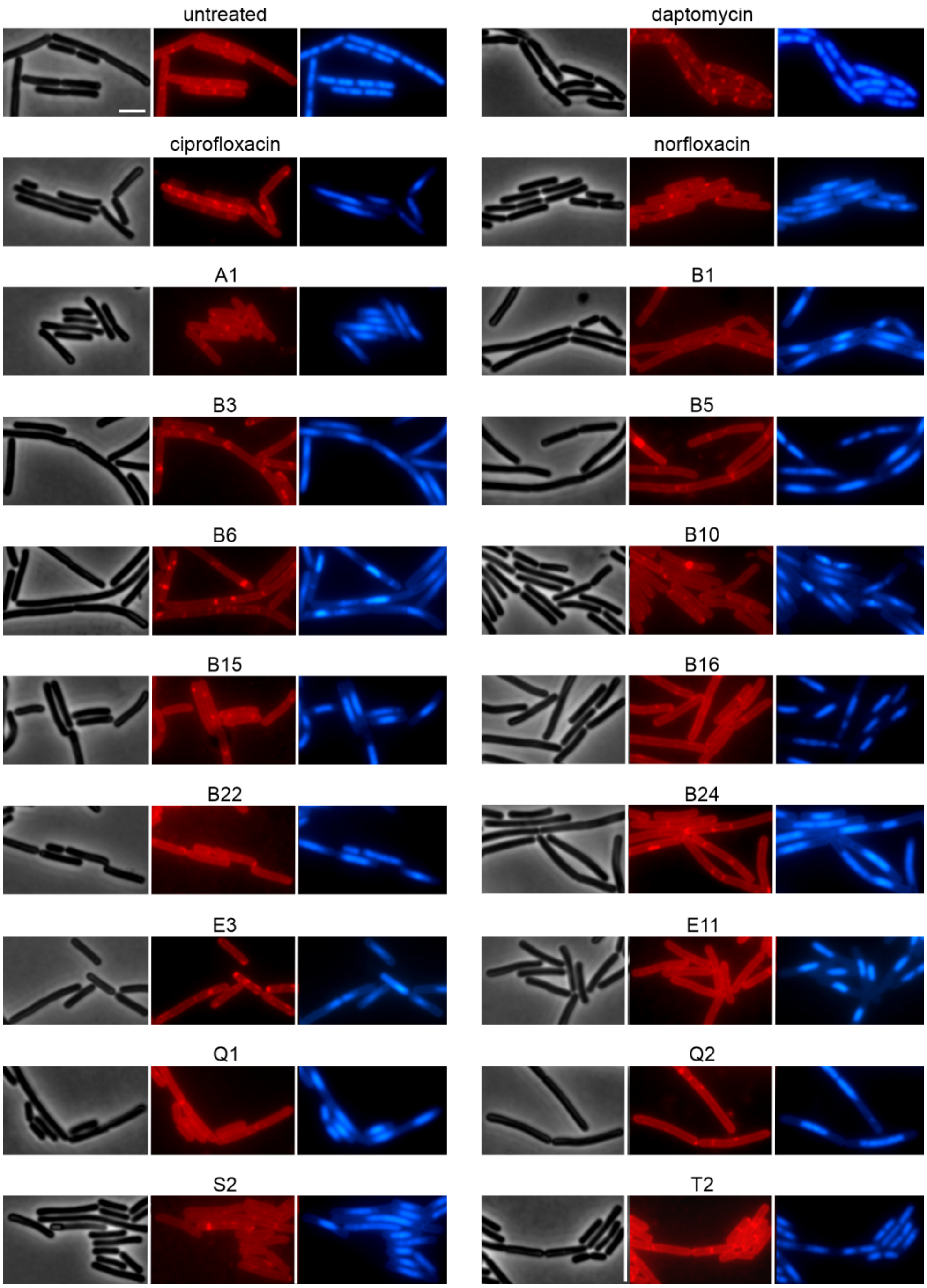

Fig. S66: Bacterial cytological profiling of *B. subtilis*. Fluorescence and phase contrast microscopy of *B. subtilis* DSM402. Cells were treated with 1x MIC of the respective compounds for 1 h prior to staining with FM4-64 (membrane, red) and DAPI (nucleoid, blue). Scale bar 2 µm.

Tab. S13: Results summary of BCP in *B. subtilis* DSM402. Concentrations represent MIC values against *B. subtilis* DSM402 in µg/mL. Images were taken after 1 h of antibiotic treatment. Phase contrast images indicate cell lysis. The fluorescent membrane dye FM4-64 reports on membrane effects. The fluorescent DNA stain DAPI reports on DNA condensation. Cip = ciprofloxacin, Nor = norfloxacin, Dap = daptomycin.

compound	concentration (µM)	phase contrast	FM4-64	DAPI	membrane damage	gyrase inhibition
**untreated**		dark	smooth	regular	no	no
**Cip**	3.01	dark	few patches	condensed	possibly	yes
**Nor**	18.11	dark	few patches	condensed	possibly	yes
**Dap**	0.61	dark	patchy	regular	yes	no
**2**	1.89	dark	smooth	condensed	no	yes
**4a**	8.84	dark	smooth	condensed	no	yes
**4c**	57.50	dark	patchy	condensed	yes	yes
**4e**	2.07	dark	smooth	condensed	no	yes
**4f**	6.21	dark	patchy	condensed	yes	yes
**4k**	6.14	dark	smooth	condensed	no	yes
**4l**	70.56	dark	smooth	condensed	no	yes
**16**	2.01	dark	patchy	condensed	yes	yes
**6a**	3.94	dark	smooth	condensed	no	yes
**8b**	33.79	dark	smooth	condensed	no	yes
**3c**	1.09	dark	patchy	condensed	yes	yes
**20a**	16.11	dark	patchy	condensed	yes	yes
**21**	2.13	dark	smooth	condensed	no	yes
**18a**	49.71	dark	smooth	condensed	no	yes
**18b**	3.10	dark	patchy	condensed	yes	yes
**12b**	21.03	dark	patchy	condensed	yes	yes
**13b**	5.83	dark	smooth	few condensed	no	possibly

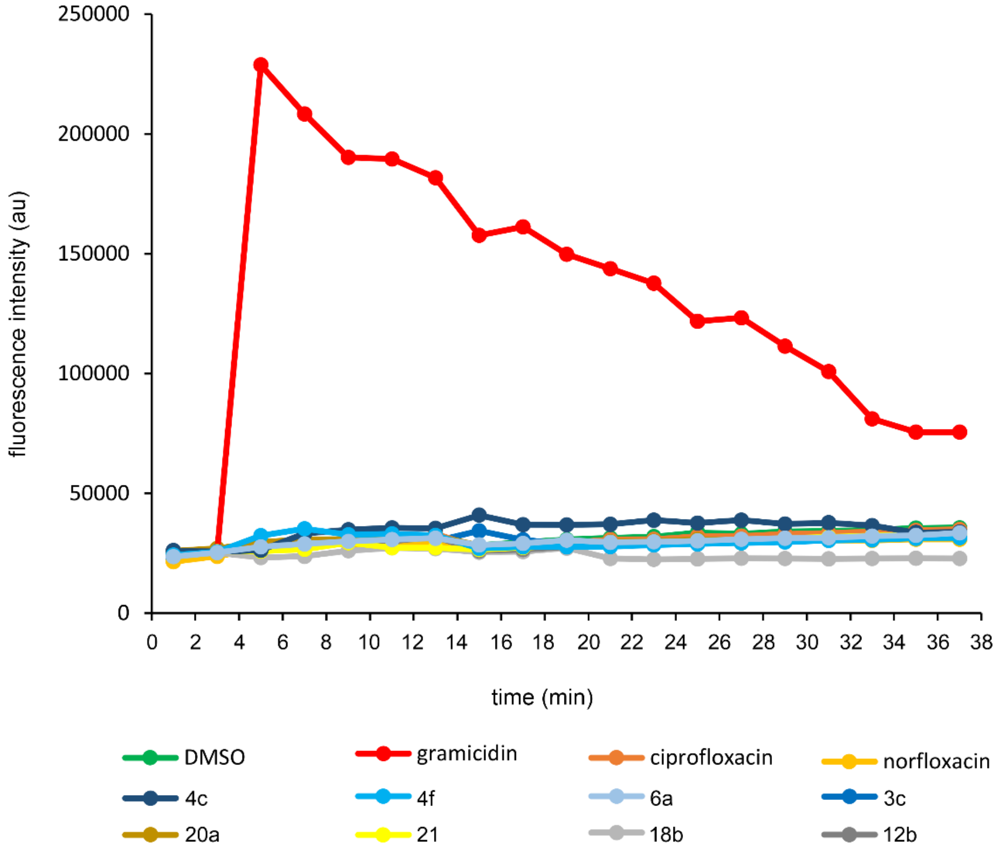

Fig. S67: Effects of the membrane potential in *B. subtilis* DSM 402. Bacteria were grown until early log phase in Muller Hinton broth and stained with the self-quenching membrane potentiometric fluorescence probe DiSC(3)5. The dye binds to polarized membranes and self-quenches. Upon depolarization, the dye is released leading to de-quenching and an increased fluorescence signal. Gramicidin (1 µg/mL), which forms a transmembrane ion channel, is used as a positive control.

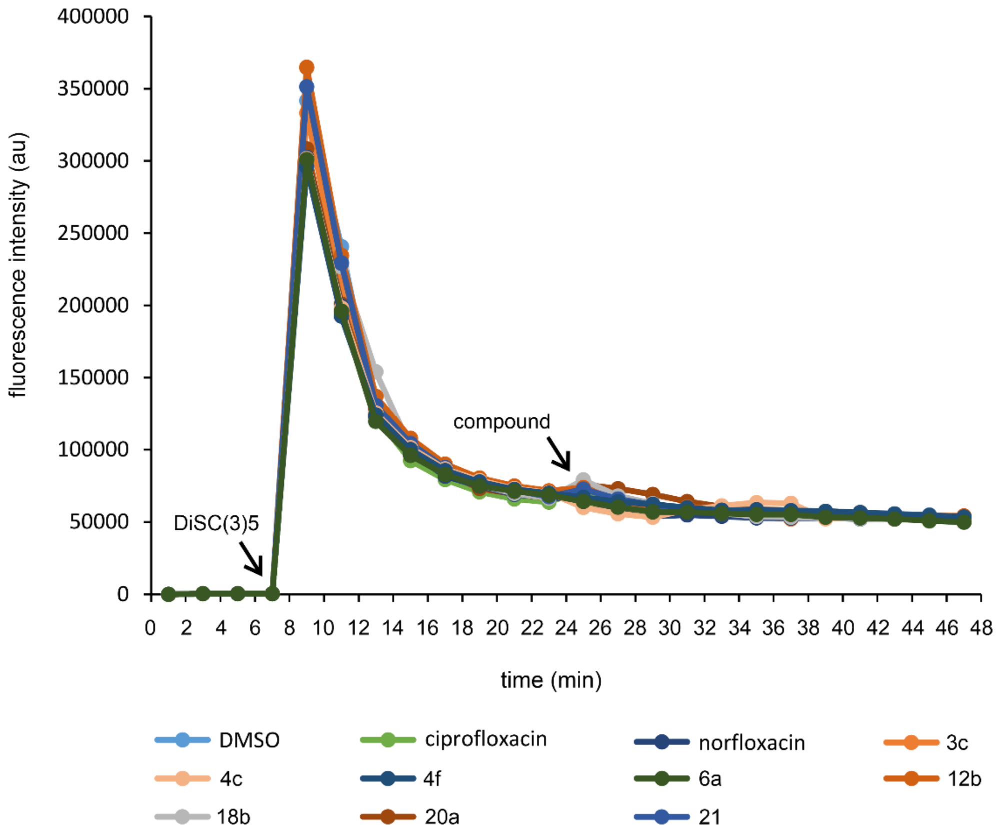

Fig. S68: Ciprofloxacin derivatives do not interfere with DiSC(3)5 fluorescence. Background fluorescence of Muller Hinton broth was measured for 144 seconds before the fluorescent dye was added. After the baseline stabilized (25 min), the respective compounds were added and measurements were continued for another 23 minutes.

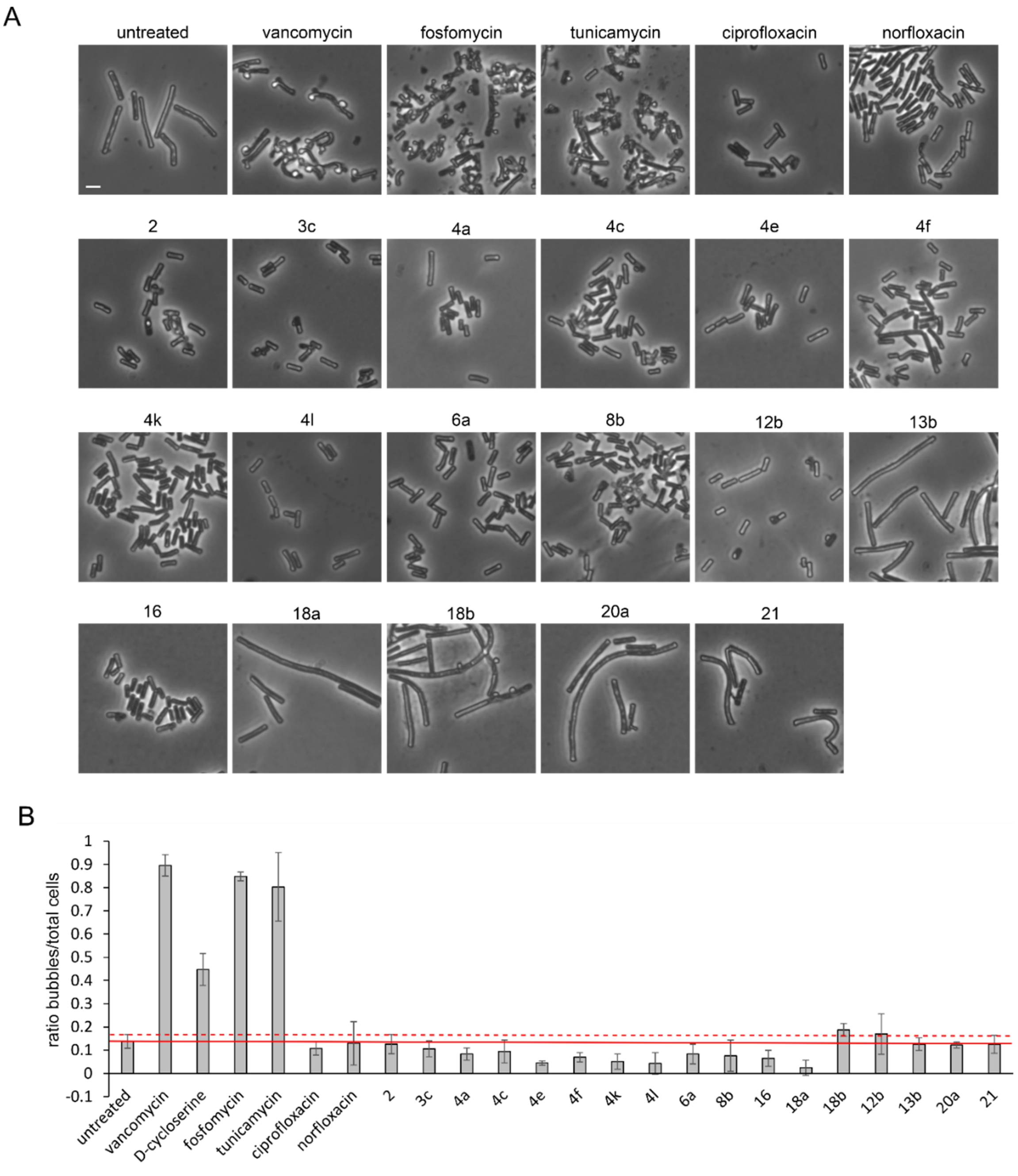

Fig. S69: Effects on peptidoglycan synthesis. **(A)** Phase contrast microscopy of *B. subtilis* 168CA. Cells were treated with 1xMIC of the respective compounds for 10 min (fosfomycin, tunicamycin, vancomycin) or 1 h (all other compounds) prior to fixation in 1:3 acetic acid/methanol. Scale bars 2 µm. **(B)** Quantification of microscopy images from (A) shown as ratio of bubbles per total number of cells. Error bars show standard deviation of three datasets. A minimum of 50 cells were examined per individual sample. Solid red line indicates the average, dotted red line the upper margin of standard deviation in the untreated control sample.

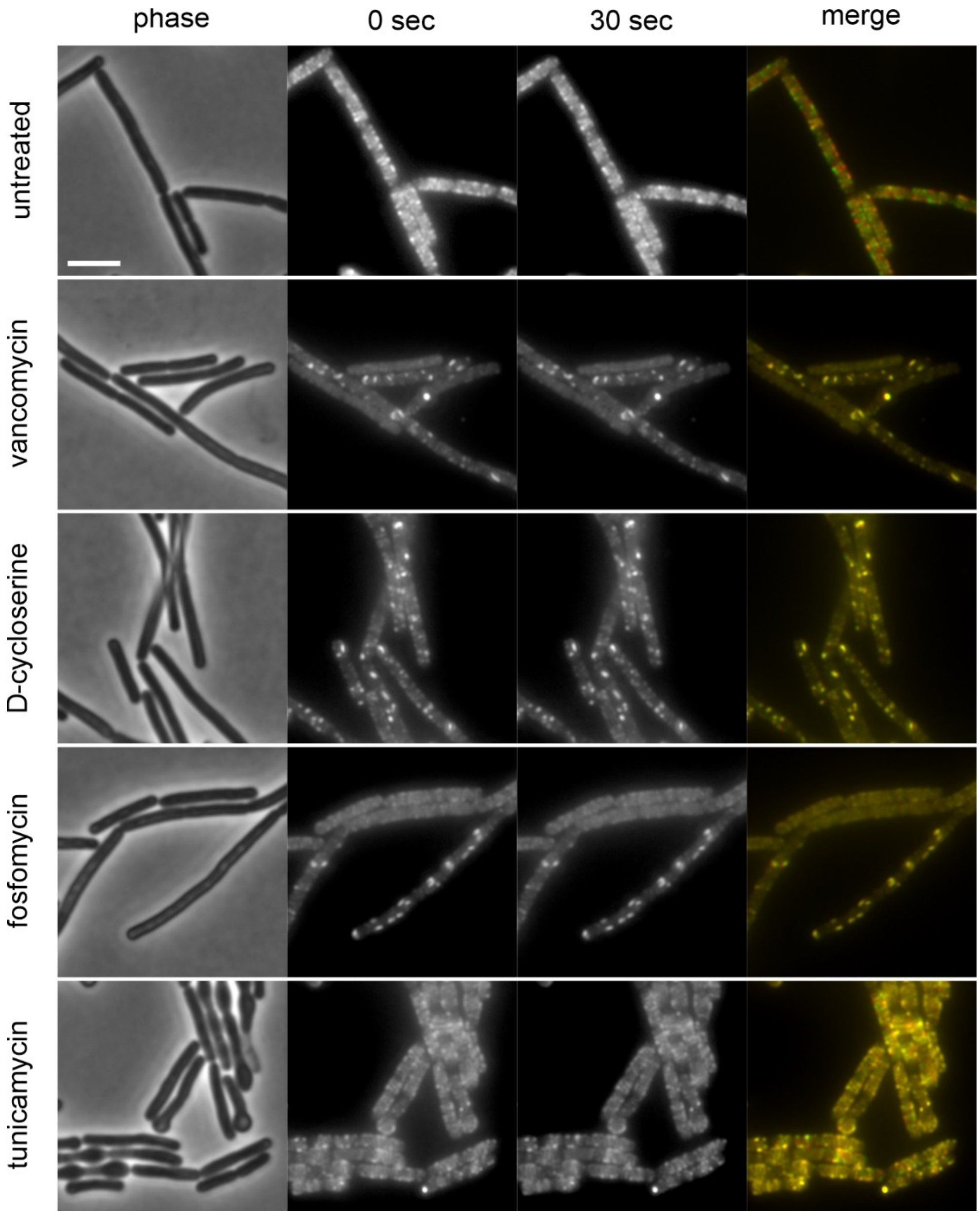

Fig. S70: MreB motility in *B. subtilis* MW10 after treatment with different cell wall synthesis inhibitors. Expression of GFP-MreB was induced with 0.3% xylose. Cells were treated with 1x MIC of the respective compounds and pictures were taken after 30 min of antibiotic exposure. Two images of the same field of view were recorded 30 sec apart and overlaid in ImageJ to visualize MreB mobility. A perfect overlap (yellow) indicates stalled MreB movement while distinct red and green spots are indicative of MreB mobility. Scale bar 2 µm.

8. HPLC analysis of lead compounds

Purity of lead compounds (**4a**, **4e** and **21**) were checked by TLC on silica gel coated aluminum sheet, Infrared spectroscopy, 1H NMR (400 MHz) and finally by HPLC analysis.

7-(4-(2-(phenylamino)acetyl)piperazin-1-yl)-1-ethyl-6-fluoro-1,4-dihydro-4-oxoquinoline-3-carboxylic acid 4a

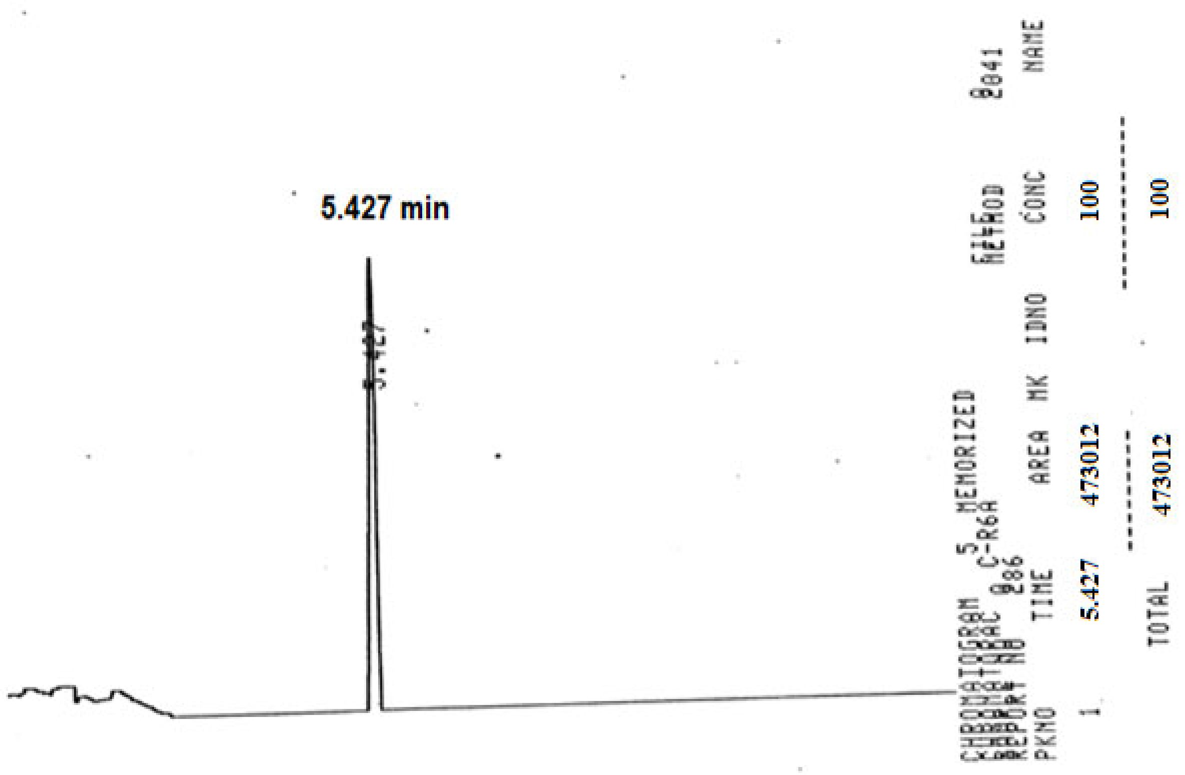

Fig. S71: Chromatogram of compound **4a.**

7-(4-(2-(2-methoxyphenylamino)acetyl)piperazin-1-yl)-1-ethyl-6-fluoro-1,4-dihydro-4-oxoquinoline-3-carboxylic acid 4e

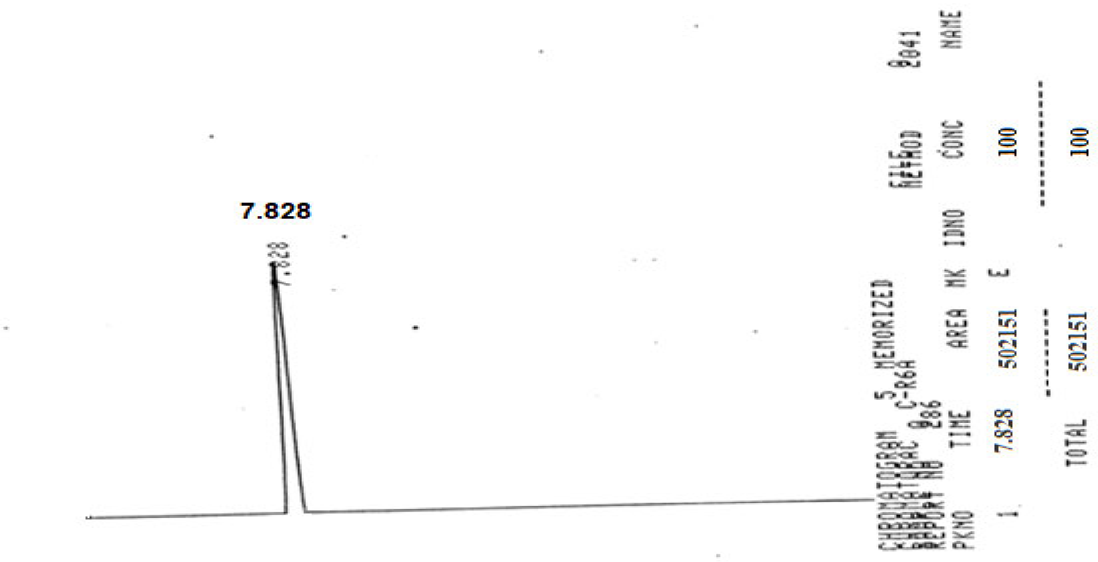

Fig. S72: Chromatogram of compound **4e.**

7-(4-((4-nitrophenylamino)methyl)piperazin-1-yl)-1-ethyl-6-fluoro-1,4-dihydro-4-oxoquinoline-3-carboxylic acid 21

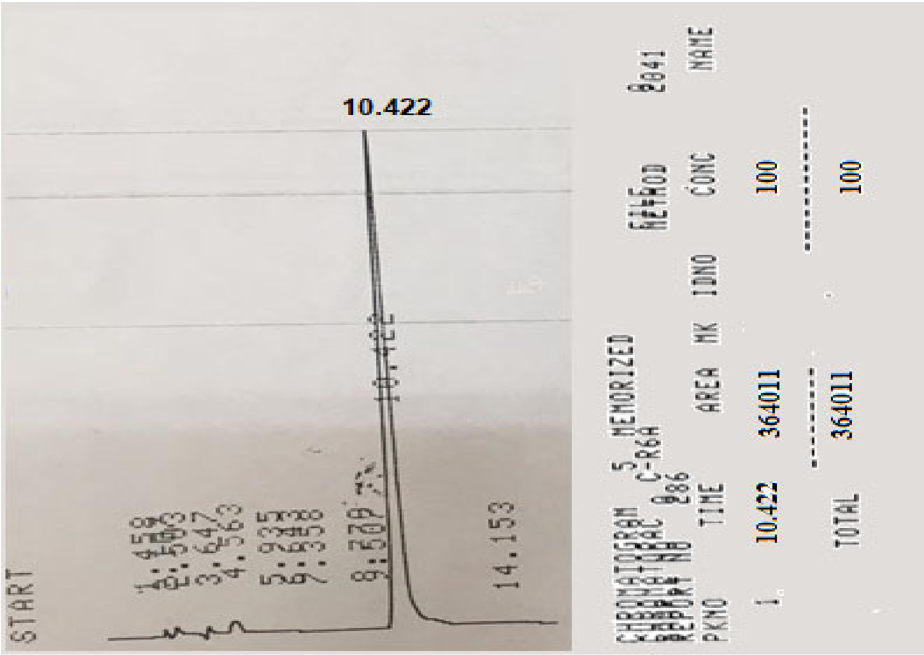

Fig. S73: Chromatogram of compound **21.**

9. Methods

Text S9: Synthesis of intermediates

The intermediate derivatives **3-7a-c** were prepared as outlined in **Scheme S6**. *p*-Aminobenzoic acid **1** was esterified with ethanol and sulfuric acid to afford benzocaine **2** [24 25]. Then, benzocaine Schiff bases **3a-c** were prepared by condensation of different aldehydes in 61-74 % yield [26 27]. In the next step, hydrazinolysis of the esters **3a-c** afforded the corresponding hydrazides **4a-c** in 64-76 % yield. Hydrazides **4a-b** were treated with phenylisothiocyanate in methanol to obtain the phenylthiosemicarbazide derivatives **5a-b** which were subjected to cyclization conditions with carbon disulphide and potassium hydroxide to form triazoles **6a-b** in 75-86 % yield**.** The hydrazides **4a-c** were refluxed under alkaline catalysis to prepare oxadiazoles **7a-c** in 76-87 % yield. Beside the expected aromatic protons, the ^{1}H NMR spectra of compounds revealed a characteristic singlet at δ ppm due to the protons and a δ ppm assigned to proton. Beside expected aromatic protons, ^{1}HNMR spectra of compounds **4a-b** were characterized by three singlet signals at δ 8.28-8.18, 7.78-7.74 and 3.41-3.32 ppm assigned to N=CH-Ph of imine group, NH and NH_2 of hydrazide group respectively. In addition, ^{1}HNMR spectra of compounds **6a-b** were characterized by two singlet signals at δ 12.08-11.93 and 10.32-10.12 ppm assigned to N=CH-Ph of imine group and SH group respectively. Moreover, compounds **7a-c** were characterized by two singlet signals at δ 11.48-10.48 and 10.49-4.04 ppm assigned to N=CH-Ph of imine group and SH group respectively.

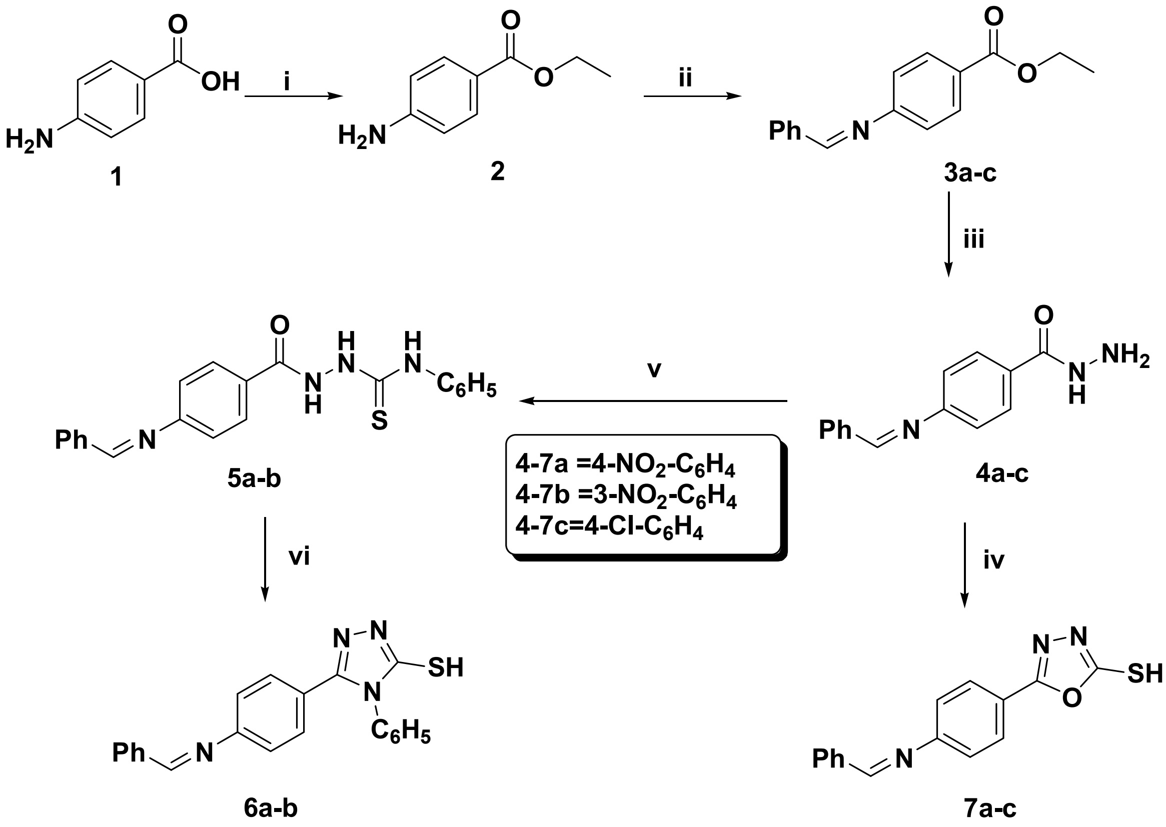

Scheme S6: Reagents and conditions: (i) EtOH, H_2SO_4, reflux, Na_2SO_4. (ii) MeOH, CH_3COOH, reflux. (iii) MeOH, hydrazine hydrate, reflux. (iv) PhSCN, MeOH, reflux. (v) MeOH, KOH, reflux, HCl. (vi) MeOH, KOH, CS_2, reflux, HCl.

Text S10: Chemical synthesis

General notes and materials

All reagents and solvents were of commercially available reagent grade quality and were used without further purification. The identity and purity of all synthesized compounds were confirmed by different techniques including spectral, elemental and HPLC analysis, and they were found sufficiently pure. The IR spectra were recorded on a Nicolet® iS10 FT-IR Spectrometer. The ^{1}H NMR spectra of compounds were recorded on 400 MHz AVANCE-III High Performance FT-NMR spectrometer, (Brucker-Biospin International AG, Switzerland). ^{13}C NMR spectra were carried on AVANCE-III High Performance FT-NMR spectrum (100 MHz), (Brucker-Biospin International AG, Switzerland). Chemical shifts (δ) are reported in part per million (ppm), Coupling constants are reported in Hertz (Hz) and spin multiplicities are represented by the following signals: Singlet (s), broad singlet (br. S) doublet (d), doublet of doublet (dd) and multiplet (m). Low resolution mass spectra (LRMS): were recorded on JEOL® mass spectrometer at faculty of science-Assiut University, Assiut, Egypt.

Elemental analysis was performed on Perkin Elmer 2400 CHN elemental analyzer and the values were within ± 0.4% of the theoretical values at the regional center for mycology and biotechnology (Al-Azhar University, Cairo/Egypt). Melting points were measured on a Stuart® SMP10 melting point apparatus.

Norfloxacin was available at Medicinal Chemistry Department-Faculty of Pharmacy-Assiut University. Identity and purity were checked by TLC on silica gel coated aluminum sheet and the spots were visualized using UV-lamp at λ 254 nm, Infrared spectroscopy analysis gave IR spectrum as reported: 3412, 3332, 3047, 2944, 2826, 1944,1731, 1616, 1583, 1481, 1251 and 1H NMR analysis give the same signals as reported (400 MHz, DMSO-d_6): 15.29 ppm (br. s, 1H, COOH), 8.91 ppm(s, 1H, H-2), 7.85 ppm (d, J_{H-F} = 12.9 Hz, 1H, H-5), 7.12 ppm (d, J_{H-F} = 6.6 Hz, 1H, H-8), 4.57 ppm (q, J = 4.8, 9.5 Hz, 2H,11-CH_2), 3.26-3.21 ppm (br. m, 4H, H of piperazine near aromatic ring), 2.92-2.87 ppm (br. m, 4H, H of piperazine near NH group), 2.51 ppm (s, 1H, NH of piperazine), 1.42 ppm (t, J = 5.1 Hz, 3H, 12-CH_3).

Synthesis of N-chloroacetyl norfloxacin derivative (2).

To a stirred solution of norfloxacin 3g (9.39 mmol) in 30 mL anhydrous tetrahydrofuran, 1.86 ml (13.33 mmol) of triethylamine was added. The mixture was then stirred in ice bath for 5 minutes and chloroacetylchloride 1.12 ml (14.06 mmol) was added dropwise, keep stirring for further 10 minutes in ice bath. The mixture was then heated at 90 °C till completion (the reaction was monitored by TLC using DCM/Methanol with ratio of 9.7:0.3 as a mobile phase), then the reaction mixture was cooled and filtered to get the product and recrystallized from DMF (88% yield)[28].

7-(4-(2-chloroacetyl)piperazin-1-yl)-1-ethyl-6-fluoro-1,4-dihydro-4-oxoquinoline-3-carboxylic acid (2)

Yield= 3.28 g (90%); yellow crystalline powder, mp:253-255 °C (reported:252 °C)[29]. ^{1}H NMR (400 MHz, CDCl3) (ppm): 15.32 (broad. s, 1H), 8.97 (s, 1H), 7.96 (d, $J_{H\text{-}F}$ = 12.9 Hz, 1H), 7.22 (d, $J_{H\text{-}F}$ = 6.6 Hz, 1H), 4.6 (q, J = 4.8, 9.5 Hz, 2H), 4.46 (s, 2H), 3.69 (br. m, 4H), 3.39 (br. m, 4H), 1.42 (t, J = 5.1 Hz, 3H).

Synthesis of N-acetylnorfloxacin derivatives with different amines (3-21).

Synthesis of target compounds was done by different methods. Method 1 was used for synthesis of target compounds hybridized with different 1ry amines, while method 2 used in case of 2nd amines. The reaction time and yield of each compound are illustrated in **Tab. S13**.

Method 1 for 1^{ry} amines

To a solution of *N*-chloroacetyl Norfloxacin **2** (0.5 g, 1.26 mmol) in15 ml DMF or dioxane (in case of **5a-b**) was added, portionwise, a solution of the respective primary amine (1.5 mmol), 1.26 mmol of potassium carbonate and a catalytic amount of potassium iodide (0.5 mmol). The resulting mixture was heated at 80 °C till completion (10-24 hours) monitored by TLC using mobile phase of chloroform and methanol with ratio of 9.7:0.3. After cooling to room temperature, water (30 ml) was added and the precipitates were then filtered, dried, and recrystallized from ethanol.

Method 2 for 2nd amines

To a solution of *N*-chloroacetyl Norfloxacin **2** (0.5 g, 1.26 mmol) in15 ml acetonitrile was added, portion wise, a solution of the respective secondary amine (1.2 mmol), 1 mmol of triethylamine or diisopropylethylamine (in case of **8a-b**) and a catalytic amount of potassium iodide (0.5 mmol The resulting mixture was heated at 60-80 °C till reaction is completed (8-12 hours) and TLC

monitoring achieved by using mobile phase of chloroform and methanol with ratio of 9.7:0.3. After cooling to room temperature, the precipitated products were then filtered, dried, and recrystallized from ethanol.

1-ethyl-7-(4-(2-(ethylamino)acetyl)piperazin-1-yl)-6-fluoro-1,4-dihydro-4-oxoquinoline-3-carboxylic acid 3a

Yield= 0.24 g (47%); yellowish white powder, mp:244-246 °C. **^{1}**HNMR (400 MHz, DMSO-d6) (ppm): 15.31 (br. s, 1H), 8.95 (s, 1H), 7.90 (d, $J_{H\text{-}F}$ = 12.9 Hz, 1H), 7.16 (d, $J_{H\text{-}F}$ = 6.6 Hz, 1H), 4.58 (q, J = 4.8, 9.5 Hz, 2H), 3.38:3.32 (br. m, 6H), 2.64:2.52 (br. m, 5H), 2.40 (t, J=7.2 Hz, 2H), 1.42 (t, J = 5.1 Hz, 3H), 1.03 (t, J = 7.2 Hz, 3H). ^{13}C NMR 100 MHz (DMSO-d_6): 173.7, 162.6, 151.1, 146.6, 144.7, 136.6, 120.9, 111.2, 109.9, 105.1, 57.8, 52.0, 51.5, 49.1, 48.5, 14.4, 11.5. Anal. Calcd for $C_{21}H_{27}FN_4O_4$: C, 60.27; H, 6.50; N, 13.39. Found: C, 60.49; H, 6.78; N, 13.58.

1-ethyl-6-fluoro-1,4-dihydro-4-oxo-7-(4-(2-(propylamino)acetyl)piperazin-1-yl)quinoline-3-carboxylic acid 3b

Yield= 0.28 g (53%); yellow powder, mp:236-238 °C. **^{1}**H NMR (400 MHz, DMSO-d6) (ppm): 15.35 (br. s, 1H), 8.63 (s, 1H), 7.86 (d, $J_{H\text{-}F}$ = 12.9 Hz, 1H), 7.08 (d, $J_{H\text{-}F}$ = 6.6 Hz, 1H), 4.43 (q, J = 4.8, 9.5 Hz, 2H), 4.09 (br. s, 1H), 3.78 (s, 2H, 2H), 3.65 (br. m, 4H), 3.22 (br. m, 4H), 2.68 (br. s, 1H), 2.55 (m, 2H), 1.37 (t, J = 5.1 Hz, 3H), 1.01 (t, J = 7.2 Hz, 3H). ^{13}C NMR 100 MHz (DMSO-d_6): 170.2, 167.7, 166.6, 150.6 (d, $J_{C\text{-}F}$ = 23.75 Hz), 147.0, 146.0, 137.0, 116.3, 112.0, 108.4, 106.3, 50.4, 44.6, 43.9, 41.5, 41.0, 15.7, 14.9. Anal. Calcd for $C_{20}H_{25}FN_4O_4$: C, 59.39; H, 6.23 N, 13.85. Found: C, 59.51; H, 6.41; N, 14.03.

7-(4-(2-(cyclohexylamino)acetyl)piperazin-1-yl)-1-ethyl-6-fluoro-1,4-dihydro-4-oxoquinoline 3-carboxylic acid 3c

Yield= 0.45 g (78%); beige powder, mp: 262-264 °C. **^{1}**H NMR (400 MHz, DMSO-d6) (ppm): 8.49 (s, 1H), 7.85 (d, $J_{H\text{-}F}$ = 12.9 Hz, 1H), 7.05 (d, $J_{H\text{-}F}$ = 6.6 Hz, 1H), 4.36 (q, J = 4.8, 9.5 Hz, 2H), 3.87-3.61 (br. m, 6H), 3.27-3.19 (br. m, 4H), 2.33 (br. s, 1H), 1.83-1.52 (m, 5H), 1.35 (t, J = 5.1 Hz, 3H), 1.25-1 (m, 6H). ^{13}C NMR 100 MHz (DMSO-d_6): 168.3, 166.7, 165.0, 150.6 (d, $J_{C\text{-}F}$ = 28.1 Hz), 149.1, 148.2, 137.7, 122.3, 112.0, 107.6, 106.7, 54.3, 49.9, 49.6, 45.4, 36.7, 25.9, 23.9, 15.2. Anal. Calcd for $C_{20}H_{25}FN_4O_4$: C, 62.87; H, 6.81; N, 12.22; Found: C, 62.71; H, 7.04; N, 12.49.

1-ethyl-6-fluoro-1,4-dihydro-4-oxo-7-(4-(2-(phenylamino)acetyl) piperazin-1-yl) quinoline-3-carboxylic acid 4a

Yield= 0.39 g (67%); pale brown powder, mp:175-177 °C. IR (KBr): 3447, 3362, 3057, 2836, 1711, 1666, 1626, 1283, 1251 cm^{-1}. ^{1}H NMR (400 MHz, DMSO-d6) (ppm): 15.29 (s, 1H), 8.95 (s, 1H), 7.95 (d, J_{H-F} = 12.9 Hz,1H), 7.15 (m, 3H), 6.6 (m, 3H), 5.55 (br. s,1H), 4.6 (q, J = 4.8, 9.5 Hz, 2H), 3.95- 3.69 (br. m, 6H), 3.44- 3.33 (br. m, 4H), 1.43 (t, J = 5.1 Hz, 3H). ^{13}C NMR 100 MHz (DMSO-d6): 176.7, 168.6, 166.5, 154.6, 151.8 (d, J_{C-F} = 248 Hz), 148.6, 146.0, 137.7, 129.4, 120.0, 116.7, 113.3, 111.8, 107.2, 49.8, 45.2, 41.3, 14.8. Anal. Calcd for $C_{24}H_{25}FN_4O_4$: C, 63.71; H, 5.57; N, 12.38. Found: C, 63.97; H, 5.71; N, 12.59.

7-(4-(2-(4-bromophenylamino) acetyl) piperazin-1-yl)-1-ethyl-6-fluoro-1,4-dihydro-4-oxoquinoline-3-carboxylic acid 4b

Yield= 0.43 g (81%); pale brown powder, mp:215-217 °C. ^{1}H NMR (400 MHz, DMSO-d6) (ppm): 15.29 (br. s, 1H), 8.8 (s, 1H), 7.8 (d, J_{H-F} = 12.9 Hz, 1H), 7.22-6.65 (m, 5 H), 5.9 (br. s, 1H), 4.5 (q, J = 4.8, 9.5 Hz, 2H), 3.98 (s, 2H), 3.77-3.66 (br. m, 4H), 3.3-3.21 (br. m, 4H) 1.37 (t, J = 5.1 Hz, 3H). ^{13}C NMR 100 MHz (DMSO-d6): 174.1, 167.9, 167.9, 150.8 (d, J_{C-F} = 247.1 Hz), 147.6, 147.2, 143.5, 136.3, 131.4, 123.1, 116.0, 114.6, 111.8, 106.9, 105.5, 50.0, 48.0, 44.7, 44.2, 14.6. Anal. Calcd for $C_{24}H_{24}BrFN_4O_4$: C, 54.25; H, 4.55; N, 10.54. Found: C, 54.43; H, 4.69; N, 10.80.

7-(4-(2-(4-chlorophenylamino)acetyl)piperazin-1-yl)-1-ethyl-6-fluoro-1,4-dihydro-4-oxoquinoline-3-carboxylic acid 4c

Yield= 0.41 g (78%); pale brown powder, mp:238-240 °C. ^{1}H NMR (400 MHz, DMSO-d6) (ppm): 15.29 (br. s, 1H), 8.9 (s, 1H), 7.9 (d, J_{H-F} = 12.9 Hz, 1H), 7.2-6.7 (m, 5 H), 5.83 (br. s,1H), 4.55 (q, J = 4.8, 9.5 Hz, 2H), 4 (s, 2H), 3.84-3.68 (br. m, 4H), 3.47-3.33 (br. m, 4H), 1.43 (t, J = 5.1 Hz, 3H). ^{13}C NMR 100 MHz (DMSO-d6): 176.4, 168.2, 166.7, 148.7, 147.6, 145.3, 137.5, 129.0, 120.0, 114.4, 111.7, 106.4, 49.8, 44.7, 40.0, 14.8. Anal. Calcd for $C_{24}H_{24}ClFN_4O_4$: C, 59.20; H, 4.97; N, 11.51. Found: C, 59.47; H, 5.15; N, 11.78.

7-(4-(2-(4-nitrophenylamino)acetyl)piperazin-1-yl)-1-ethyl-6-fluoro-1,4-dihydro-4-oxoquinoline-3-carboxylic acid 4d

Yield= 0.38 g (67%); dark yellow powder, mp: 278-280 °C; IR (KBr): 3436, 3312, 3041, 2826, 1717, 1659, 1623, 1438, 1263, 1224 cm^{-1}. ^{1}H NMR (400 MHz, DMSO-d6) (ppm): 8.65 (s, 1H), 8.02 (m, 3H), 7.17 (d, J =7.2 Hz, 2H), 6.8 (br. s, 2H), 4.45 (q, J = 4.8, 9.5 Hz, 2H), 4.21 (s, 2H), 3.79-3.7 (br. m, 4H), 3.38-3.32 (br. m, 4H), 1.39 (t, J = 5.1 Hz, 3H). ^{13}C NMR 100 MHz (DMSO-d6): 174.1, 167.9, 167.9, 151.0, 147.7, 147.0, 143.3, 136.4, 131.4, 115.9, 114.7, 111.6, 106.9, 106.1, 105.5, 50.0, 47.6, 44.7, 41.4, 14.6. Anal. Calcd for $C_{24}H_{24}FN_5O_6$: C, 57.94; H, 4.86; N, 14.08. Found: C, 58.13; H, 4.97; N, 13.89.

7-(4-(2-(2-methoxyphenylamino)acetyl)piperazin-1-yl)-1-ethyl-6-fluoro-1,4-dihydro-4-oxoquinoline-3-carboxylic acid 4e

Yield= 0.48 g (72%); yellowish green, mp:172-174 °C. ^{1}H NMR (400 MHz, DMSO-d6) (ppm): 15.32 (br. s, 1H), 8.95 (s, 1H), 7.94 (d, J_{H-F} = 12.9 Hz,1H), 7.2 (d, J_{H-F} = 12.9 Hz, 1H), 6.85-6.77 (m, 2H), 6.62-6.57 (m, 2H), 5.24 (br. s,1H), 4.59 (q, J = 4.8, 9.5 Hz, 2H), 3.99 (d, J = 3.6 Hz 2H), 3.8 (s, 3H), 3.73 (br. m, 4H), 3.39 (br. m, 4H), 1.42 (t, J = 5.1 Hz, 3H). ^{13}C NMR 100 MHz (DMSO-d6): 176.8, 168.4, 166.7, 155.0 (d, J_{C-F} = 247.6 Hz), 152.3, 149.0, 146.5, 145.7, 137.9, 121.6, 120.2, 116.9, 111.8, 110.5, 107.9, 106.9, 55.7, 50.0, 45.0, 44.3, 41.7, 15.0. Anal. Calcd for $C_{25}H_{27}FN_4O_5$: C, 62.23; H, 5.64; N, 11.61. Found: C, 62.46; H, 5.72; N, 11.88.

7-(4-(2-(4-methoxyphenylamino)acetyl)piperazin-1-yl)-1-ethyl-6-fluoro-1,4-dihydro-4-oxoquinoline-3-carboxylic acid 4f

Yield= 0.4 g (83%); grey powder, mp:227-229 °C. ^{1}H NMR (400 MHz, DMSO-d6) (ppm): 15.29 (br. s, 1H), 8.95 (s, 1H), 7.95 (d, J_{H-F} = 12.9 Hz, 1H), 6.65-7.35 (m, 5H), 4.6 (br. m, 3H), 4.03-3.65 (br. m, 9H), 3.48-3.34 (br. m, 4H), 1.44 (t, J = 5.1 Hz, 3H). ^{13}C NMR 100 MHz (DMSO-d6): 176.6, 166.4, 159.9, 152.1, 148.9, 145.7, 143.0, 142.4, 137.8, 123.4, 120.0, 115.1, 114.1, 111.7, 106.7, 55.9, 49.8, 49.1, 46.0, 41.7, 14.8. Anal. Calcd for $C_{25}H_{27}FN_4O_5$: C, 62.23; H, 5.64; N, 11.61. Found: C, 62.51; H, 5.83; N, 11.79.

7-(4-(2-(3-methoxyphenylamino)acetyl)piperazin-1-yl)-1-ethyl-6-fluoro-1,4-dihydro-4-oxoquinoline-3-carboxylic acid 4g

Yield= 0.43 g (65%); beige powder, mp:176-178 °C. ^{1}H NMR (400 MHz, DMSO-d6) (ppm): 15.29 (br. s, 1H), 8.8 (s, 1H), 7.75 (d, J_{H-F} = 12.9 Hz,1H), 7.1-6.95 (m, 2H), 6.22-6.16 (m, 3H), 5.61 (br. s, 1H), 4.45 (q, J = 4.8, 9.5 Hz, 2H), 3.97 (s, 2H), 3.79-3.6 (br. m, 7H), 3.3-3.16 (br. m,

4H), 1.36 (t, *J* = 5.1 Hz, 3H). ^{13}C NMR 100 MHz (DMSO-d6): 176.2, 168.1, 166.1, 160.4, 154.1 (d, J_{C-F} = 248.3 Hz), 149.5, 148.6, 145.3, 137.2, 129.5, 119.5, 111.3, 107.1, 106.2, 105.6, 101.7, 98.4, 54.7, 49.6, 49.1, 44.9, 43.8, 14.5. Anal. Calcd for $C_{25}H_{27}FN_4O_5$: C, 62.23; H, 5.64; N, 11.61. Found: C, 62.49; H, 5.81; N, 11.84.

1-ethyl-6-fluoro-1,4-dihydro-4-oxo-7-(4-(2-(1'-carboxyphenylamino-4'-yl)acetyl)piperazin-1-yl)quinoline-3-carboxylic acid 4h

Yield= 0.47 g (84%); pale brown powder, mp:212-214 °C. IR (KBr): 3459, 3371, 3062, 2943, 1708, 1660, 1638, 1279, 1171cm^{-1}. ^{1}H NMR (400 MHz, DMSO-d6) (ppm): 15.8-14.7 (br. s, 2H), 8.95 (s, 1H), 7.94 (d, J_{H-F} = 12.9 Hz, 1H), 7.68 (d, *J* = 8 Hz, 2H), 7.21 (d, J_{H-F} = 6.6 Hz, 1H), 6.6 (d, *J* = 8 Hz, 2H), 5.98 (br. S, 1H), 5 (br. S, 2H), 4.6 (s, 2H), 3.44-3.25 (br. m, 8H), 1.44 (t, *J* = 5.1 Hz, 3H). ^{13}C NMR 100 MHz (DMSO-d6): 176.5, 166.5, 165.8, 154.1, 151.9, 148.9, 145.3, 137.7, 131.7, 120.0, 116.1, 113.2, 111.9, 107.4, 106.1, 61.6, 49.6, 39.7, 14.8. Anal. Calcd for $C_{25}H_{25}FN_4O_6$: C, 60.48; H, 5.08; N, 11.28. Found: C, 60.29; H, 5.17; N, 11.52.

7-(4-(2-(4-flurophenylamino)acetyl)piperazin-1-yl)-1-ethyl-6-fluoro-1,4-dihydro-4-oxoquinoline-3-carboxylic acid 4i

Yield= 0.4 g (69%); beige powder, mp: 269-271 °C. ^{1}H NMR (400 MHz, DMSO-d6) (ppm): 15.34 (s, 1H), 8.37 (s, 1H), 7.81 (d, J_{H-F} = 12.9 Hz, 1H), 7.14-6.73 (m, 5 H), 5.25 (br. s,1H), 4.30 (q, *J* = 4.8, 9.5 Hz, 2H), 4.03 (d, *J* = 3.6 Hz, 2H), 3.7 (br. m, 4H), 3.22 (br. m, 4H), 1.33 (t, *J* = 5.1 Hz, 3H). ^{13}C NMR 100 MHz (DMSO-d_6): 175.0, 168.0, 164.3, 153.6, 151.0, 148.6, 146.5, 143.6, 136.9, 112.5, 111.6, 110.5, 108.8, 106.2, 104.2, 50.3, 47.7, 45.4, 42.0, 15.0. Anal. Calcd for $C_{24}H_{24}F_2N_4O_4$: C, 61.27; H, 5.14; N, 11.91. Found: C, 61.38; H, 5.37; N, 11.87.

1-ethyl-6-fluoro-1,4-dihydro-7-(4-(acetylphenylamino)acetylpiperazin-1-yl)-4-oxoquinoline-3-carboxylic acid 4j

Yield= 0.42 g (67%); pale brown powder, mp:214-216 °C. ^{1}H NMR (400 MHz, DMSO-d6) (ppm): 15.27 (s, 1H), 8.95 (s, 1H), 7.95 (d, J_{H-F} = 12.9 Hz, 1H), 7.23-7.05 (m, 3H), 6.74-6.48 (m, 3H), 4.59 (q, *J* = 4.8, 9.5 Hz, 2H), 4.00-3.64 (m, 6H), 3.44-3.32 (br. m, 4H), 2.51 (s, 3H), 1.43 (t, *J* = 5.1 Hz, 3H). ^{13}C NMR 100 MHz (DMSO-d_6): 192.8, 176.5, 167.1, 159.2, 153.8, 148.8, 145.6, 143.2, 137.6, 132.0, 124.7, 119.0, 116.1, 112.3, 111.5, 107.2, 60.6, 56.5, 49.8, 49.1, 24.4, 14.7. Anal. Calcd for $C_{26}H_{27}FN_4O_5$: C, 63.15; H, 5.50; N, 11.33. Found: C, 63.39; H, 5.62; N, 11.59.

7-(4-(2-(2,4-difluorophenylamino)acetyl)piperazin-1-yl)-1-ethyl-6-fluoro-1,4-dihydro-4-oxoquinoline-3-carboxylic acid 4k

Yield= 0.38 g (58%); white powder, mp:281-283 °C. ^{1}H NMR (400 MHz, DMSO-d6) (ppm): 15.27 (br. s, 1H), 8.39 (s, 1H), 7.82 (d, $J_{H\text{-}F}$ = 12.9 Hz, 1H), 7.12 (m, 1 H), 7.02 (d, $J_{H\text{-}F}$ = 6.6 Hz, 1H), 6.9 (m, 1H), 6.77 (m, 1H), 5.25 (br. s, 1H), 4.31 (q, *J* = 4.8, 9.5 Hz, 2H), 4.04 (d, *J*= 3.6 Hz, 2H), 3.71 (br. m, 4H), 3.24 (br. m, 4H), 1.34 (t, J = 5.1 Hz, 3H). ^{13}C NMR 100 MHz (DMSO-d6): 173.9, 168.0, 167.6, 153.2, 150.9, 148.9, 146.9, 143.2, 136.5, 133.2, 123.2, 113.1, 111.8, 110.7, 105.8, 103.7, 103.1, 49.9, 47.3, 44.3, 41.5, 14.5. Anal. Calcd for $C_{24}H_{23}F_3N_4O_4$: C, 59.01; H, 4.75; N, 11.47. Found: C, 59.23; H, 4.96; N, 11.73.

1-ethyl-6-fluoro-1,4-dihydro-4-oxo-7-(4-(2-(pyridin-4-ylamino)acetyl)piperazin-1-yl)quinoline-3-carboxylic acid 4l

Yield= 0.41 g (72%); pale brown powder, mp:294-296 °C. ^{1}HNMR (400 MHz, DMSO-d6) (ppm): 15.26 (br. s, 1H), 8.95 (s, 1H), 8.2 (br. s,1H), 8.04 (d, *J* = 8 Hz, 2H), 7.95 (d, $J_{H\text{-}F}$ = 12.9 Hz, 1H), 7.21 (d, $J_{H\text{–}F}$ = 6.6 Hz, 1H), 6.86 (d, *J* = 8 Hz, 2H), 5.34 (q, *J* = 4.8, 9.5 Hz, 2H), 4.61 (s, 2H), 3.73-3.67 (br. m, 4H), 3.51-3.38 (br. m, 4H), 1.44 (t, *J* = 5.1 Hz, 3H). ^{13}C NMR 100 MHz (DMSO-d6) 176.8, 166.5, 165.2, 159.4, 154.5, 152.0, 149.0, 145.4, 145.0, 137.7, 120.1, 111.9, 109.1, 107.7, 106.5, 57.6, 49.6, 14.5. Anal. Calcd for $C_{23}H_{24}FN_5O_4$: C, 60.92; H, 5.33; N, 15.44. Found: C, 60.70; H, 5.48; N, 15.28.

1-ethyl-6-fluoro-1,4-dihydro-4-oxo-7-(4-(2-(thiazol-2-ylamino)acetyl)piperazin-1-yl)quinoline-3-carboxylic acid 5a

Yield= 0.39 g (67%); brown powder, mp:238-240 °C. ^{1}H NMR (400 MHz, DMSO-d6) (ppm): 15.3 (br. s, 1H), 8.93 (s, 1H), 8.69 (s, 1H), 7.83 (m, 2H), 7.11 (m, 2H), 4.52 (m, 4H), 3.77-3.59 (br. m, 8H), 1.25 (t, *J* = 5.1 Hz, 3H). ^{13}C NMR 100 MHz (DMSO-d_6): 176.3, 171.6, 166.2, 162.5, 151.5 (d, $J_{C\text{-}F}$ = 24.7 Hz), 148.5, 145.2 (d, $J_{C\text{-}F}$ = 22.3 Hz), 137.5, 136.2, 119.5, 111.8, 108.8, 107.1, 106.1, 61.1, 53.8, 49.2, 41.2, 14.4. Anal. Calcd for $C_{21}H_{22}FN_5O_4S$: C, 54.89; H, 4.83; N, 15.24. Found: C, 55.13; H, 4.95; N, 15.51.

7-(4-(2-(benzo[d]thiazol-2-ylamino)acetyl)piperazin-1-yl)-1-ethyl-6-fluoro-1,4-dihydro-4-oxoquinoline-3-carboxylic acid 5b

Yield= 0.41 g (64%); white powder, mp:276-278 °C. ^{1}HNMR (400 MHz, DMSO-d6) (ppm): 15.26 (s, 1H), 8.95 (s, 1H), 8.20 (s, 1H), 8.04 (d, J = 7.1 Hz, 2H), 7.95 (d, J_{H-F} = 12.9 Hz, 1H), 7.22 (d, J_{H-F} = 6.6 Hz, 1H), 6.86 (d, J = 7.1 Hz, 2H), 5.34 (s, 2H), 4.61 (q, J = 4.8, 9.5 Hz, 2H), 3.74:3.66 (br. m, 4H), 3.52-3.37 (br. m, 4H), 1.44 (t, J = 5.1 Hz, 3H). ^{13}C NMR 100 MHz (DMSO-d_6): 172.0, 166.6, 165.9, 164.4, 154.1 (d, J_{C-F} = 23.4 Hz), 149.7, 145.6, 137.6, 136.7, 132.1, 131.2, 123.5, 121.3, 120.1, 109.2, 107.6, 106.8, 61.7, 49.6, 48.7, 41.6, 15.0. Anal. Calcd for $C_{25}H_{24}FN_5O_4S$: C, 58.93; H, 4.75; N, 13.74. Found: C, 59.17; H, 4.83; N, 14.02.

1-ethyl-6-fluoro-1,4-dihydro-4-oxo-7-(4-(2(isonicotinohydrazide)acetyl)piperazin-1-yl)quinoline-3-carboxylic acid 16

Yield= 0.4 g (64%); pale yellow powder, mp:225-227 °C. IR (KBr): 3419(NH str), 3354, 3067, 2923, 1727, 1658, 1630, 1269, 1241 cm^{-1}. ^{1}H NMR (400 MHz, DMSO-d6) (ppm): 15.31 (br. s, 1H), 9-8.96 (br. d, 2H), 8.03-7.83 (m, 3H), 7.29-7.15 (m, 3H), 4.65-4.58 (m, 4H), 3.94-3.64 (br. m, 9H), 1.44 (t, J = 5.1 Hz, 3H). ^{13}C NMR 100 MHz (DMSO-d_6): 176.8, 167.3, 166.6, 157.6, 152.3 (d, J_{C-F} = 27.8 Hz), 149.3, 147.4, 144.4, 141.1, 137.7, 122.2, 115.9, 111.8, 107.6, 106.8, 52.4, 49.9, 49.6, 41.6, 14.9. Anal. Calcd for $C_{24}H_{25}FN_6O_5$: C, 58.06; H, 5.08; N, 16.93. Found: C, 58.27; H, 5.32; N, 17.19.

1-ethyl-6-fluoro-1,4-dihydro-4-oxo-7-(4-(2-(2,3-dioxoindolin-1-yl)acetyl)piperazin-1-yl)quinoline-3-carboxylic acid 6a

Yield= 0.53 g (83%); brown powder, mp:284-286 °C. IR (KBr): 3245, 3136, 2837, 1748, 1723, 1645, 1623, 1246, 1107 cm^{-1}. ^{1}HNMR (400 MHz, DMSO-d6) (ppm): 15.11 (s, 1H), 8.64 (s, 1H), 7.96 (d, J_{H-F} = 12.9 Hz, 1H), 7.91-7.86 (m, 2H), 7.80-7.74 (m, 2H), 6.82 (d, J_{H-F} = 6.6 Hz, 1H), 4.73 (s, 2H), 4.33 (q, J = 4.8, 9.5 Hz, 2H), 3.36-3.28 (br. m, 4H), 2.93-2.87 (br. m, 4H), 1.57 (t, J = 5.1 Hz, 3H). ^{13}C NMR 100 MHz (DMSO-d6): 183.6, 176.7, 166.6, 166.0, 159.5, 154.6, 152.1 (d, J_{C-F} = 24.7 Hz), 148.9, 146.0, 138.9, 138.5, 137.7, 124.7, 123.8, 118.1, 112.5, 111.7, 107.6, 106.5, 61.9, 50.5, 49.9, 49.5, 14.7. Anal. Calcd for $C_{26}H_{23}FN_4O_6$: C, 61.66; H, 4.58; N, 11.06. Found: C, 61.43; H, 4.79; N, 11.28.

7-(4-(2-(5-bromo-2,3-dioxoindolin-1-yl)acetyl)piperazin-1-yl)-1-ethyl-6-fluoro-1,4-dihydro-4-oxoquinoline-3-carboxylic acid 6b

Yield= 0.56 g (75%); brown powder, mp:282-284 °C. IR (KBr): 3365, 3140, 2847, 1754, 1728, 1653, 1631, 1251, 1112 cm^{-1}. ^{1}H NMR (400 MHz, DMSO-d6) (ppm): 15.31 (s, 1H), 8.93 (s, 1H), 7.97-7.65 (m, 3H), 7.38-7.08 (m, 2H), 4.69-4.44 (m, 4H), 3.35-3.25 (m, 4H), 2.90-2.70 (m, 4H), 1.41 (t, J = 5.1 Hz, 3H). ^{13}C NMR 100 MHz (DMSO-d6): 182.4, 176.8, 166.7, 164.7, 158.3, 154.6, 151.0, 149.3, 145.7, 140.6, 137.9, 127.2, 119.5, 115.5, 114.2, 111.9, 107.9, 106.5, 52.7, 49.7, 44.7, 42.0, 15.2. Anal. Calcd for $C_{26}H_{22}BrFN_4O_6$: C, 53.35; H, 3.79; N, 9.57. Found: C, 53.59; H, 4.02; N, 9.81.

1-ethyl-6-fluoro-1,4-dihydro-4-oxo-7-(4-(2-(piperidin-1-yl)acetyl)piperazin-1-yl)quinoline-3-carboxylic acid 7a

Yield= 0.5 g (87%); white powder, mp:283-285 °C. ^{1}H NMR (400 MHz, DMSO-d6) (ppm): 15.3 (br. s, 1H), 8.96 (s, 1H), 7.96 (d, J_{H-F} = 12.9 Hz, 1H), 7.21 (d, J_{H-F} = 6.6 Hz, 1H), 4.6 (q, J = 4.8, 9.5 Hz, 2H), 3.82-3.65 (br. m, 4H), 3.4-3.24 (m, 10H), 2.46-2.36 (br. m, 4H), 1.45 (m, 5H). ^{13}C NMR 100 MHz (DMSO-d6): 176.5, 173.7, 162.9, 151.6, 147.3, 144.0, 136.8, 121.9, 112.3, 110.5, 106.5, 57.7, 49.0, 46.9, 45.8, 42.9, 42.8, 15.0, 9.0. Anal. Calcd for $C_{23}H_{29}FN_4O_4$: C, 62.15; H, 6.58; N, 12.60. Found: C, 62.43; H, 6.71; N, 12.84.

1-ethyl-6-fluoro-1,4-dihydro-7-(4-(2-morpholinoacetyl)piperazin-1-yl)-4-oxoquinoline-3-carboxylic acid 7b

Yield= 0.51 g (85%); white powder, mp:273-275 °C. ^{1}H NMR (400 MHz, DMSO-d6) (ppm): 15.3 (br. s, 1H), 8.96 (s, 1H), 7.96 (d, J_{H-F} = 12.9 Hz, 1H), 7.21 (d, J_{H-F} = 6.6 Hz, 1H), 4.6 (q, J = 4.8, 9.5 Hz, 2H), 3.79 (s, 2H), 3.68-3.58 (br. m, 4H), 3.4-3.21 (br. m, 8H), 2.47-2.41 (br. m, 4H), 1.44 (t, J = 5.1 Hz, 3H). ^{13}C NMR 100 MHz (DMSO-d6): 173.0, 162.4, 151.2, 146.8, 143.4, 136.6, 121.6, 111.6, 110.0, 105.8, 57.2, 48.5, 46.5, 45.0, 42.7, 14.5. Anal. Calcd for $C_{22}H_{27}FN_4O_5$: C, 59.18; H, 6.10; N, 12.55. Found: C, 59.32; H, 5.98; N, 12.71.

7-(4-(2-(1H-imidazol-1-yl)acetyl)piperazin-1-yl)-1-ethyl-6-fluoro-1,4-dihydro-4-oxoquinoline-3-carboxylic acid 8a

Yield= 0.39 g (72%); white powder, mp:253-255 °C. ^{1}HNMR (400 MHz, DMSO-d6) (ppm): 15.36 (br. s, 1H), 8.96 (s, 1H), 7.92 (d, J_{H-F} = 12.9 Hz, 1H), 7.52 (d, J = 7.8 Hz, 1H), 7.41 (s, 1H), 7.25 (d, J = 7.6 Hz, 1H), 7.19 (d, J_{H-F} = 6.6 Hz, 1H), 4.67-4.45 (m, 4H), 2.84-2.69 (br. m, 4H), 2.39-2.23 (br. m, 4H), 1.41 (t, J = 5.1 Hz, 3H). ^{13}C NMR 100 MHz (DMSO-d6): 176.7, 166.6, 164.4,

152.1, 149.1, 148.9, 145.6, 137.8, 137.6, 120.1, 120.0, 111.9, 107.6, 106.8, 52.3, 49.9, 49.5, 44.4, 14.8. Anal. Calcd for $C_{21}H_{22}FN_5O_4$: C, 59.01; H, 5.19; N, 16.38; Found: C, 60.11; H, 5.07; N, 16.15.

1-ethyl-6-fluoro-1,4-dihydro-7-(4-(2-(5-nitro-1H-1,2,4-triazol-1-yl)acetyl)piperazin-1-yl)-4-oxoquinoline-3-carboxylic acid 8b

Yield= 0.46 g (77%); yellow powder, mp:262-264 °C. IR (KBr): 3467, 3064, 2992, 1716, 1668, 1628, 1505, 1240 cm-1; ^{1}HNMR (400 MHz, DMSO-d6) (ppm): 15.3 (br. s, 1H), 8.96 (s, 1H), 8.81 (s, 1H), 7.95 (d, $J_{H\text{-}F}$ = 12.9 Hz, 1H), 7.22 (d, $J_{H\text{-}F}$ = 6.6 Hz, 1H), 5.59 (s, 2H), 4.6 (q, J = 4.8, 9.5 Hz, 2H), 3.72 (br. m, 4H), 3.44 (br. m, 4H), 1.42 (t, J = 5.1 Hz, 3H). ^{13}C NMR 100 MHz (DMSO-d6) 177.1, 166.7, 164.3, 162.4, 152.3, 149.3, 148.6, 145.7, 137.9, 120.2, 111.9, 107.9, 107.2, 52.7, 49.7, 44.7, 42.0, 14.9. Anal. Calcd for $C_{20}H_{20}FN_7O_6$: C, 50.74; H, 4.26; N, 20.71. Found: C, 50.86; H, 4.53; N, 20.54. LRMS for [$C_{20}H_{20}FN_7O_6$] + [M]+ calculated: 473.15 found: 473.14.

7-(4-(2-(3,4-dihydro-4-oxoquinazolin-2-ylthio)acetyl)piperazin-1-yl)-1-ethyl-6-fluoro-1,4-dihydro-4-oxoquinoline-3-carboxylic acid 14

Yield= 0.36 g (53%); beige powder, mp:277-279 °C. ^{1}H NMR (400 MHz, DMSO-d6) (ppm): 15.33 (s, 1H), 12.64 (s, 1H), 8.96 (s, 1H), 8.02 (d, J= 8 Hz, 1H), 7.96 (d, $J_{H\text{-}F}$ = 12.9 Hz, 1H), 7.73 (m, 1H), 7.52-7.39 (m, 2H), 7.22 (d, $J_{H\text{-}F}$ = 6.6 Hz, 1H), 4.59 (q, J = 4.8, 9.5 Hz, 2H), 4.36 (s, 2H), 3.90-3.71 (br. m, 4H), 3.46 (br. m, 4H), 1.42 (t, J = 5.1 Hz, 3H). ^{13}C NMR 100 MHz (DMSO-d_6) 177.1, 167.1, 166.4, 156.0, 149.3, 145.7, 137.6, 136.3, 135.0, 129.3, 126.6, 120.6, 119.9, 112.2, 111.6, 107.9, 106.9, 49.7, 46.0, 42.4, 33.6, 15.2. Anal. Calcd for $C_{26}H_{24}FN_5O_5S$: C, 58.09; H, 4.50; N, 13.03. Found: C, 58.21; H, 4.73; N, 13.29.

Synthesis of norfloxacin mannich derivatives

Synthesis of norfloxacin-isatin derivative hybrid (20a-c)

Equimolar mixture of norfloxacin (3 g, 9.39 mmol) and the respective indoline-2,3-dione (9.39 mmol) in ethanol (30 ml) was treated with 2 ml of formalin (37%) and heated at reflux overnight (chloroform and methanol with ratio of 9.7:0.3 used as a mobile phase in TLC monitoring). After cooling, the precipitated product was filtered, washed with methanol and water and dried. Recrystallization from DMF/water mixture afforded the desired Mannich bases.

Synthesis of norfloxacin-p-nitroaniline mannich base (21)

2 mL of formalin (37%) was added to an equimolar mixture of norfloxacin (9.39 mmol) and P-nitroaniline (9.39 mmol) in ethanol, few drops of glacial acetic acid were added as a catalyst (as mannich base was not formed using the conditions as in 4.1.4.1 procedure), the reaction mixturewas heated at reflux for 24 hours. After cooling, the precipitate was Filtered, washed with cold methanol and dried. The obtained mannich base purified through crystallization using DMF and water.

1-ethyl-6-fluoro-7-(4-((5-fluoro-2,3-dioxoindolin-1-yl)methyl)piperazin-1-yl)-1,4-dihydro-4-oxoquinoline-3-carboxylic acid 20a

Yield= 4.1 g (89%); orange powder, mp:248-250 °C. IR (KBr): 3451, 3049, 2852, 1749, 1716, 1619, 1250. ^{1}H NMR (400 MHz, DMSO-d6) (ppm): 15.35 (br. s, 1H), 8.95 (s, 1H), 7.9 (d, $J_{H\text{-}F}$ = 12.9 Hz, 1H), 7.57 (t. d, J= 12 $_{H\text{-}F}$, 9, 2.8 Hz, 1H), 7.49 (d. d, J = 7.2,2.8 Hz, 1H), 7.37 (d. d, J= 8.4, 3.6 Hz, 1H), 7.17 (d, $J_{H\text{-}F}$ = 6.6 Hz, 1H), 4.59 (q, J= 7.2, 2H), 4.53 (s, 2H), 3.33:3.31 (m, 4H), 2.84-2.81 (m, 4H), 1.4 (t, J = 5.1 Hz, 3H). ^{13}C NMR (100 MHz, DMSO-d_6): 182.6, 176.3, 166.2, 159.2, 157.5, 151.5 (d, $J_{C\text{-}F}$ = 245.2 Hz), 148.5, 147.5, 145.5 (d, $J_{C\text{-}F}$ = 22.6 Hz), 137.2, 124.0, 123.7, 119.4, 118.6, 113.5, 111.5, 107.1, 106.1, 61.5, 49.6, 49.4, 49.1, 14.7. Anal. Calcd for $C_{25}H_{22}F_2N_4O_5$: C, 60.48; H, 4.47; N, 11.29. Found: C, 60.71; H, 4.59; N, 11.46.

7-(4-((5-methyl-2,3-dioxoindolin-1-yl)methyl)piperazin-1-yl)-1-ethyl-6-fluoro-1,4-dihydro-4-oxoquinoline-3-carboxylic acid 20b

Yield= 3.95 g (85%); orange powder, mp:247-249 °C. ^{1}H NMR (400 MHz, DMSO-d6) (ppm): 15.36 (br. s, 1H), 8.96 (s, 1H), 7.92 (d, $J_{H\text{-}F}$ = 12.9 Hz, 1H), 7.54-7.18 (m, 4H), 4.56 (m, 4H), 3.4-3.3 (m, 4H), 2.85-2.78 (m, 4H), 2.31 (s, 3H), 1.41 (t, J = 5.1 Hz, 3H). ^{13}C NMR 100 MHz (DMSO-d_6): 183.9, 176.6, 166.6, 159.6, 152.2, 149.8, 149.1, 146.0, 138.8, 137.7, 133.1, 125.0, 119.9, 118.0, 112.3, 111.6, 107.5, 106.6, 61.8, 50.1, 49.8, 49.5, 20.5, 14.8. Anal. Calcd for $C_{26}H_{25}FN_4O_5$: C, 63.41; H, 5.12; N, 11.38. Found: C, 63.29; H, 5.32; N, 11.50.

7-(4-((5-methoxy-2,3-dioxoindolin-1-yl)methyl)piperazin-1-yl)-1-ethyl-6-fluoro-1,4-dihydro-4-oxoquinoline-3-carboxylic acid 20c

Yield= 3.7 g (77%); red powder, mp:251-253 °C. ^{1}H NMR (400 MHz, DMSO-d6) (ppm): 15.34 (br. s, 1H), 8.94 (s, 1H), 7.91 (d, $J_{H\text{-}F}$ = 12.9 Hz, 1H), 7.2 (m, 4H), 4.53 (m, 4H), 3.78 (s, 3H), 3.31

(m, 4H), 2.81-2.78 (m, 4H), 1.39 (t, J = 5.1 Hz, 3H). ^{13}C NMR 100 MHz (DMSO-d$_6$): 185.1, 176.7, 166.5, 160.0, 152.0, 149.3, 145.1, 144.5, 137.6, 125.4, 124.2, 118.6, 113.7, 112.1, 109.2, 107.7, 107.0, 60.3, 56.4, 49.7, 47.3, 43.0, 15.0. Anal. Calcd for $C_{26}H_{25}FN_4O_6$: C, 61.41; H, 4.96; N, 11.02. Found: C, 61.59; H, 5.12; N, 11.28.

7-(4-((4-nitrophenylamino)methyl)piperazin-1-yl)-1-ethyl-6-fluoro-1,4-dihydro-4-oxoquinoline-3-carboxylic acid 21

Yield= 3.45 g (78%); yellow powder, mp:246-248 °C. ^{1}H NMR (400 MHz, DMSO-d6) (ppm): 15.36 (br. s, 1H), 8.97 (s, 1H), 8.19-7.88 (m, 3H), 7.79 (s, 1H), 7.03 (m, 3H), 4.6 (br. s, 2H), 3.98 (s, 2H), 3.38-3.3 (m, 4H), 2.76-2.68 (m, 4H), 1.42 (t, J = 5.1 Hz, 3H). ^{13}C NMR 100 MHz (DMSO-d$_6$): 176.8, 166.7, 156.3, 154.6 (d, $J_{C\text{-}F}$ = 24.1 Hz), 149.0, 146.0, 141.6, 137.6, 126.6, 126.4, 114.9, 112.8, 111.9, 107.9, 64.9, 51.1, 50.1, 49.5, 15.0. Anal. Calcd for $C_{23}H_{24}FN_5O_5$: C, 58.84; H, 5.15; N, 14.92. Found: C, 59.11; H, 5.34; N, 15.19.

Synthesis of benzocaine [24]

To a stirred solution of p-aminobenzoic acid (10 g, 72.99 mmol) in ethanol (100 ml), sulfuric acid (5 ml) was added and heated under reflux temperature for 5 h. The reaction was monitored by thin layer chromatography. The reaction mixture was concentrated under vacuo, neutralized with saturated sodium bicarbonate solution (100 ml). The precipitated solid was filtered, washed with water (50 ml), dried under reduced pressure to afford white solid and recrystallized by aqueous methanol (mp of benzocaine: 89-91 °C, reported mp: 90 °C) [25].

Synthesis of benzocaine Schiff bases with different benzaldhydes [26]

To 2 g (12.1 mmol) of benzocaine in ethanol, 12.1 mmol of corresponding benzaldehyde and few drops of glacial acetic acid as a catalyst were added. Then the reaction mixture was heated at reflux overnight. When the reaction was completed, the reaction mixture solvent was reduced under vacuum and cooled overnight. Next, the solid residue was collected and washed with cold ethanol and water. The product was used without any further purification.

(E)-ethyl 4-(4-nitrobenzylideneamino)benzoate

Yield= 2.68 g (74%); greenish yellow powder, mp:174-176 °C (reported mp:177-180)[27].

(E)-ethyl 4-(3-nitrobenzylideneamino)benzoate

Yield= 2.63 g (73%); greenish yellow powder, mp:162-164 °C (reported mp:163)[27].

(E)-ethyl 4-(4-chlorobenzylideneamino)benzoate

Yield= 2.49 g (71%); greenish yellow crystals, mp:125-127 °C (reported mp:127.5-128)[27].

Synthesis of different 4-(benzylideneamino)benzohydrazide

To a solution of the respective benzocaine Schiff bases (2 g) in ethanol (25 mL) were added 3 equivalents of hydrazine hydrate (99 %). The mixture was heated at reflux for four hours. The solvent was evaporated under vacuum and the obtained solid was recrystallized from ethanol.

4-(4-nitrobenzylideneamino)benzohydrazide 17a

Yield= 1.45 g (76%); yellow powder, mp: 123-125 °C; ^{1}HNMR (400 MHz, DMSO-d6) (ppm): 8.18 (s, 1H), 8.16 (d, J = 8 Hz, 2H), 7.74 (s, 1H), 7.69-7.54 (m, 6H), 3.31 (br. s, 2H). ^{13}C NMR 100 MHz (DMSO-d_6): 166.7, 154.0, 146.0, 144.0, 134.2, 131.6, 125.6, 124.6, 113.2. Anal. Calcd for $C_{14}H_{12}N_4O_3$ (284.27): C, 59.15; H, 4.25; N, 19.71; Found: C, 59.43; H, 4.47; N, 19.95.

4-(3-nitrobenzylideneamino)benzohydrazide 17b

Yield= 1.39 g (73%); yellow powder, mp: 105-107 °C; ^{1}HNMR (400 MHz, DMSO-d6) (ppm): 8.27 (m, 2H), 8.05 (m, 1H), 8.03 (m, 1H), 7.88 (d, J = 7.2 Hz, 2H), 7.78 (s, 1H), 7.61 (m, 1H), 7.22 (m, 2H), 3.42 (br. s, 2H). ^{13}C NMR 100 MHz (DMSO-d_6): 161.0, 148.7, 139.0, 135.7, 135.3, 134.9, 131.5, 131.1, 130.5, 123.1, 121.8, 119.2. Anal. Calcd for $C_{14}H_{12}N_4O_3$ (284.27): C, 59.15; H, 4.25; N, 19.71; Found: C, 59.37; H, 4.39; N, 19.88.

4-(4-chlorobenzylideneamino)benzohydrazide 17c

Yield= 1.36 g (71%); yellowish white powder, mp: 101-103 °C; ^{1}HNMR (400 MHz, DMSO-d6) (ppm): 8.54 (s, 1H), 7.93 (d, J = 7.6 Hz, 2H), 7.68 (d, J = 7.6 Hz, 2H), 7.58-7.37 (m, 4H), 7.26 (s, 1H), 3.66 (br. s, 2H). ^{13}C NMR 100 MHz (DMSO-d_6): 166.3, 153.9, 146.1, 144.0, 134.6, 131.5, 125.8, 124.5, 113.2. Anal. Calcd for $C_{14}H_{12}ClN_3O$ (273.72): C, 61.43; H, 4.42; N, 15.35; Found: C, 61.59; H, 4.60; N, 15.49.

Synthesis of different 4-(benzylideneamino)benzophenyl thiosemicarbazid

To 0.5 g of 4-(benzylideneamino)benzohydrazid, was added 1 equivalent of phenylisothiocyanate in methanol (15 ml), and the mixture heated at reflux for 6-8 hours. The solvent evaporated under reduced pressure and the obtained solid used in the next reaction directly.

Synthesis of different 4-(benzylideneamino)phenyl)-4-phenyl-4H-1,2,4-triazole-3-thiol 9a-b

A stirring mixture of ***4-(benzylideneamino)benzophenyl thiosemicarbazid*** compounds and 15 ml ethanolic solution (6%) of potassium hydroxide was refluxed overnight. After cooling, the ethanol was distilled off under reduced pressure and the residue was dissolved in water, and then acidified with dilute hydrochloric acid (10%). The resulting precipitate was filtered, washed with water, dried, and recrystallized from ethanol.

4-(4-nitrobenzylideneamino)phenyl)-4-phenyl-4H-1,2,4-triazole-3-thiol 9a

Yield= 0.42 g (86%); yellow powder, mp: 227-229 °C; ^{1}HNMR (400 MHz, DMSO-d6) (ppm): 12.08 (s, 1H), 10.32 (s, 1H), 8.27-8.18 (m, 6H), 7.61-7.36 (m, 6H) 7.24 (m, 1H). ^{13}C NMR 100 MHz (DMSO-d_6): 177.0, 148.2, 141.0, 140.7, 139.4, 128.9, 128.7, 126.7, 126.2, 124.2. Anal. Calcd for $C_{21}H_{15}N_5O_2S$ (401.44): C, 62.83; H, 3.77; N, 17.45; S, 7.99; Found: C, 62.70; H, 3.94; N, 17.62; S, 8.12.

4-(3-nitrobenzylideneamino)phenyl)-4-phenyl-4H-1,2,4-triazole-3-thiol 9b

Yield= 0.36 g (75%); yellow powder, mp: 205-207 °C; ^{1}H NMR (400 MHz, DMSO-d6) (ppm): 10.70 (s, 1H), 8.16 (m, 2H), 7.97-7.70 (m, 7H), 7.53-7.38 (m, 4H), 7.27 (m, 1H). ^{13}C NMR (DMSO-d_6): 187.6, 167.2, 155.4, 147.3, 143.4, 134.8, 132.4, 131.7, 130.5, 129.9, 128.8, 127.0, 122.0, 118.6, 114.7. Anal. Calcd for $C_{21}H_{15}N_5O_2S$ (401.44): C, 62.83; H, 3.77; N, 17.45; S, 7.99; Found: C, 62.98; H,.91; N, 17.66; S, 8.08.

Synthesis of different 4-(benzylideneamino)phenyl)-1,3,4-oxadiazole-2-thiol 10a-c

A mixture of hydrazide (0.25 g), potassium hydroxide (1 equivalent), carbon disulfide (3 equivalents) and ethanol, was heated under reflux with stirring until the evolution of hydrogen sulfide ceased (12 hours). Ethanol was distilled off under reduced pressure and the residue was dissolved in water and then acidified with dilute hydrochloric acid (10%). The resulting precipitate was filtered, washed with water, dried, and recrystallized from ethanol.

4-(4-nitrobenzylideneamino)phenyl)-1,3,4-oxadiazole-2-thiol 10a

Yield= 0.25 g (87%); yellow powder, mp:227-229 °C; ^{1}HNMR (400 MHz, DMSO-d6) (ppm): 8.38 (s, 1H), 8.34 (d, *J* = 8.2 Hz, 2H), 8.28 (d, *J* = 7.8 Hz, 2H), 8.23-8 (m, 4H), 5.75 (s, 1H). ^{13}C NMR 100 MHz (DMSO-d_6): 166.3, 161.2, 150.6, 149.2, 136.8, 131.3, 130.1, 128.4, 125.1, 124.7, 124.2. Anal. Calcd for $C_{15}H_{10}N_4O_3S$ (326.33): C, 55.21; H, 3.09; N, 17.17; O, 14.71; S, 9.83; Found: C, 55.43; H, 3.27; N, 17.43; S, 9.95.

4-(3-nitrobenzylideneamino)phenyl)-1,3,4-oxadiazole-2-thiol 10b

Yield= 0.24 g (84%); yellow powder, mp:218-220 °C; ^{1}HNMR (400 MHz, DMSO-d6) (ppm): 12.22 (s, 1H), 10.51 (s, 1H), 8.41-8.25 (m, 3H), 7.94-7.88 (m, 4H), 7.75 (m, 1H). ^{13}C NMR 100 MHz (DMSO-d_6): 176.4, 161.0, 149.0, 143.0, 142.0, 136.3, 134.6, 131.3, 130.6, 126.6, 124.9, 123.6, 122.6. Anal. Calcd for $C_{15}H_{10}N_4O_3S$ (326.33): C, 55.21; H, 3.09; N, 17.17; O, 14.71; S, 9.83.; Found: C, 55.47; H, 3.24; N, 17.40: S, 9.87.

4-(4-chlorobenzylideneamino)phenyl)-1,3,4-oxadiazole-2-thiol 10c

Yield= 0.22 g (76%); yellow powder, mp: 175-177 °C; ^{1}HNMR (400 MHz, DMSO-d6) (ppm): 11.48 (s, 1H), 7.98-7.84 (m, 4H), 7.83-7.24 (m, 4H), 4.03 (s, 1H). ^{13}C NMR 100 MHz (DMSO-d_6): 166.3, 160.7, 139.9, 138.7, 136.9, 131.2, 130.1, 128.4, 125.1, 124.6, 124.3. Anal. Calcd for $C_{15}H_{10}ClN_3OS$ (315.78): C, 57.05; H, 3.19; N, 13.31: S, 10.15.; Found: C, 57.28; H, 3.28; N, 13.54; S, 10.29.

Synthesis of different 4-(benzylideneamino)benzohydrazide acetylnorfloxacin derivatives 18a-c

To a solution of *N*-chloroacetylnorfloxacin **2** (0.4 g, 1.01 mmol) in acetonitrile (15 ml), 1equivalent of corresponding hydrazide (**17a-c**), 0.14 g (1.01 mmol) of potassium carbonate and potassium iodide (0.083 g, 0.5 mmol) as a catalyst were added. Then, the mixture was heated at 60-80°C till completion (8-12 hrs.), cooled and the precipitate was filtered. Then the product was washed with acetonitrile and diethyl ether and recrystallized from ethanol.

7-(4-(4-(4-nitrobenzylideneamino)benzohydrazide)acetyl)piperazin-1-yl)-1-ethyl-6-fluoro-1,4-dihydro-4-oxoquinoline-3-carboxylic acid 18a

Yield= 0.45 g (69%); yellow powder, mp:240-242 °C; ^{1}HNMR (400 MHz, DMSO-d6) (ppm): 15.33 (br. s, 1H), 8.98-8.87 (m, 3H), 8.27-7.66 (m, 7H), 7.25-7.05 (m, 3H), 4.61-4.54 (m, 4H), 4.4 (br. s, 1H), 3.82-3.67 (m, 8H), 1.41 (t, J = 5.1 Hz, 3H). ^{13}C NMR 100 MHz (DMSO-d_6): 176.6,

167.9, 166.6, 163.4, 154.6, 152.0, 149, 148.9, 146.0, 143.9, 137.6, 131.6, 125.8, 124.7, 124.5, 119.9, 119.6, 111.6, 107.5, 106.6, 52.6, 50.0, 49.6, 41.5, 15.2. Anal. Calcd for $C_{32}H_{30}FN_7O_7$ (643.62): C, 59.72; H, 4.70; N, 15.23; Found: C, 59.94; H, 4.81; N, 15.50.

7-(4-(4-(3-nitrobenzylideneamino)benzohydrazide)acetyl)piperazin-1-yl)-1-ethyl-6-fluoro-1,4-dihydro-4-oxoquinoline-3-carboxylic acid 18b

Yield= 0.36 g (55%); yellow powder, mp:270-272 °C; ^{1}HNMR (400 MHz, DMSO-d6) (ppm): 8.48-8.40 (br. s, 2H), 8.28 (m, 1H), 8.03 (m, 1H), 7.87 (d, J = 7.8 Hz, 1H), 7.92-7.71 (m, 4H), 7.61 (m, 1H), 7.06 (br. s, 1H), 6.98 (d, $J_{H–F}$ = 6.6 Hz ,1H), 4.33-4.26 (m, 4H), 4.15 (br. s, 1H), 3.71-3.66 (m, 8H), 1.33 (t, J = 5.1 Hz, 3H). ^{13}C NMR 100 MHz (DMSO-d_6): 174.6, 170.8, 168.1, 155.7, 151.3, 148.6, 147.3, 139.1, 136.7, 135.0, 132.5, 131.8, 131, 121.9, 119.2, 115.6, 112.3, 105.9, 60.6, 50.2, 49.4, 48.0, 14.9. Anal. Calcd for $C_{32}H_{30}FN_7O_7$ (643.62): C, 59.72; H, 4.70; N, 15.23; Found: C, 59.86; H, 4.85; N, 15.49.

7-(4-(4-(4-chlorobenzylideneamino)benzohydrazide)acetyl)piperazin-1-yl)-1-ethyl-6-fluoro-1,4-dihydro-4-oxoquinoline-3-carboxylic acid 18c

Yield= 0.28 g (44%); yellowish white powder, mp:235-237 °C; ^{1}HNMR (400 MHz, DMSO-d6) (ppm): 9.16-9.10 (br. s, 2H), 8.80 (br. s, 1H), 8.35-8.30 (m, 4H), 8.24 (d, $J_{H\text{-}F}$ = 12.9 Hz, 1H), 8.03-7.98 (m, 4H), 7.43 (d, $J_{H–F}$ = 6.6 Hz ,1H), 4.73 (q, *J* = 4.8, 9.5 Hz, 2H), 4.58 (s, 2H), 4.40 (br. s, 1H), 4.34-4.19 (m, 4H), 3.74-3.57 (m, 4H), 1.77 (t, J = 5.1 Hz, 3H). ^{13}C NMR 100 MHz (DMSO-d_6):170.7, 168.1, 161.1, 158.0, 155.1, 150.6, 147.1, 146.8, 136.7, 136.5, 133.1, 130.5, 129.6, 121.8, 115.6, 112.1, 106.0, 60.6, 50.2, 48.0, 44.0, 14.9. Anal. Calcd for $C_{32}H_{30}ClFN_6O_5$ (633.07): C, 60.71; H, 4.78; N, 13.28; Found: C, 60.89; H, 4.92; N, 13.51.

Synthesis of different 5-(4-(benzylideneamino)phenyl)-4-phenyl-*4H*-1,2,4-triazole-3-thiol acetylnorfloxacin derivatives 12a-b

To a solution of *N*-chloroacetylnorfloxacin **2** (0.4 g, 1.01 mmol) in acetonitrile (15 ml), 1 equivalent of the corresponding triazolethiol, 0.14 g (1.01 mmol) of potassium carbonate and potassium iodide (0.083 g, 0.5 mmol) as a catalyst were added. The reaction mixture was heated at 60-80°C till completion (8-12 hrs.), then cooled, the precipitate was filtered and washed with acetonitrile and diethyl ether and recrystallized from ethanol.

7-(5-(4-(4-nitrobenzylideneamino)phenyl)-4-phenyl-4H-1,2,4-triazole-3-thiol)acetyl)piperazin-1-yl)-1-ethyl-6-fluoro-1,4-dihydro-4-oxoquinoline-3-carboxylic acid 12a

Yield= 0.46 g (60%); yellow powder, mp:222-224 °C; IR (KBr): 3292(OH str), 3053(aromatic C-H str), 2929(aliphatic C-H str), 1725(carboxylic C=O str), 1659(carbamidic C=O str), 1624(quinolone C=O str), 1589(C=N str), 1497(N=O str), 1255(C-O) cm^{-1}. ^{1}HNMR (400 MHz, DMSO-d6) (ppm): 15.31 (br. s, 1H),9.87 (s, 1H), 8.94 (s, 1H), 8.48 (d, J_{H-F} = 12.9 Hz, 1H), 8.27 (d, *J*= 8 Hz, 2H), 7.96 (d, *J*= 8 Hz, 2H), 7.05-7.69 (m, 10 H), 4.57 (q, J = 4.8, 9.5 Hz, 2H), 4.22 (s, 2H), 3.81 (m, 4H), 3.43 (m, 4H), 1.4 (t, *J* = 5.1 Hz, 3H). ^{13}C NMR 100 MHz (DMSO-d_6): 168.9, 166.2, 166.1, 160.8, 159.3, 158.1, 154.0, 151.0, 148.6, 147.7, 141.4, 140.2, 130.3, 129.0, 128.8, 128.0, 126.3, 124.1, 123.9, 123.0, 120.3, 111.4, 111.1, 107.1, 106.7, 49.2, 45.9, 41.5, 30.8, 14.4 Anal. Calcd for $C_{39}H_{33}FN_8O_6S$ (760.79): C, 61.57; H, 4.37; N, 14.73; S, 4.21; Found: C, 61.78; H, 4.59; N, 14.90; S, 4.32. LRMS for $[C_{24}H_{23}FN_4O_4]^+$ $[M]^+$ calculated: 760.22 found: 760.20.

7-(5-(4-(3-nitrobenzylideneamino)phenyl)-4-phenyl-4H-1,2,4-triazole-3-thiol)acetyl)piperazin-1-yl)-1-ethyl-6-fluoro-1,4-dihydro-4-oxoquinoline-3-carboxylic acid 12b

Yield= 0.41 g (53%); yellowish white powder, mp:263-265 °C; 1HNMR (400 MHz, DMSO-d6) (ppm): 8.52 (br. s, 2H), 8.25 (m, 1H), 8.13 (m, 1H), 7.93-6.94 (m, 13H), 4.43 (s, 2H), 4.36 (q, *J* = 4.8, 9.5 Hz, 2H), 3.74-3.65 (m, 8H), 1.36 (t, *J* = 5.1 Hz, 3H). ^{13}C NMR 100 MHz (DMSO-d_6): 175.0, 172.6, 168.1, 165.8, 156.2, 153.0, 151.4, 148.6, 148.1, 134.3, 133.8, 131.0, 130.7, 129.7, 128.4, 128.1, 124.9, 122.7, 122.2,112.2, 112.0. 106.1, 50.3, 48.4, 46.0, 36.8, 15.3. Anal. Calcd for $C_{39}H_{33}FN_8O_6S$ (760.79): C, 61.57; H, 4.37; N, 14.73; S, 4.21; Found: C, 61.76; H, 4.62; N, 14.98; S, 4.29.

Synthesis of different 5-(4-(4-nitrobenzylideneamino)phenyl)-1,3,4-oxadiazole-2-thiol acetylnorfloxacin derivatives 13a-c

To 0.4 g (1.01 mmol) of *N*-chloroacetyl Norfloxacin **2** in acetonitrile, 1 equivalent of corresponding oxadiazole and 0.14 ml (1.01 mmol) of triethylamine were added, then the mixture was heated at 60-80 °C for 8-12 hrs. Few milligrams (0.083 g, 0.5 mmol) of potassium iodide can be added as a catalyst. When reaction completed, the reaction mixture was cooled, filtered to collect the precipitate, washed with acetonitrile and diethyl ether and recrystallized from ethanol.

7-(5-(4-(4-nitrobenzylideneamino)phenyl)-1,3,4-oxadiazole-2-thiol)acetyl)piperazin-1-yl)-1-ethyl-6-fluoro-1,4-dihydro-4-oxoquinoline-3-carboxylic acid 13a

Yield= 0.38 g (55%); brown powder, mp:294-296 °C; ^{1}HNMR (400 MHz, DMSO-d6) (ppm): 8.51-8.37 (m, 5H), 8.28-7.83 (m, 6H), 7.03 (d, $J_{H–F}$ = 6.6 Hz, 1H), 4.66 (s, 2H), 4.32 (q, J = 4.8, 9.5 Hz, 2H), 3.77 (m, 4H), 3.54 (m. 4H), 1.35 (t, J = 5.1 Hz, 3H). ^{13}C NMR (100 MHz, DMSO-d$_6$): 171.1, 167.8, 165.5, 164.5, 161.4, 160.1, 156.6, 150.3, 148.8, 147.9, 137.5, 137.1, 134.3, 131.8, 122.0, 121.1, 118.8, 114.0, 112.1, 108.6, 108.0, 106.2, 50.3, 50.0, 48.4, 45.7, 14.9. Anal. Calcd for $C_{33}H_{28}FN_7O_7S$ (685.68): C, 57.80; H, 4.12; N, 14.30; S, 4.68; Found: C, 58.04; H, 4.29; N, 14.47; S, 4.79.

7-(5-(4-(3-nitrobenzylideneamino)phenyl)-1,3,4-oxadiazole-2-thiol)acetyl)piperazin-1-yl)-1-ethyl-6-fluoro-1,4-dihydro-4-oxoquinoline-3-carboxylic acid 13b

Yield= 0.35 g (50%); yellow powder, mp:277-279 °C; ^{1}HNMR (400 MHz, DMSO-d6) (ppm): 8.64 (s, 1H), 8.53 (s, 1H), 8.44-8.01 (m, 3H), 7.93-7.82 (m, 3H), 7.75-7.43 (m, 3H), 7.06 (d, $J_{H–F}$ = 6.6 Hz, 1H), 4.62 (s, 2H), 4.38 (q, J = 4.8, 9.5 Hz, 2H), 3.82-3.68 (m, 8H), 1.37 (t, J = 5.1 Hz, 3H). ^{13}C NMR 100 MHz (DMSO-d$_6$): 168.1, 167.3, 166.4, 165.5, 148.8, 144.5 (d, J_{C-F} = 247 Hz), 136.9, 134.3, 131.7, 131.1, 128.4, 127.9, 126.9, 126.1, 118.7, 114.1, 111.9, 106.1, 50.0, 48.4, 45.7, 42.0, 15.0. Anal. Calcd for $C_{20}H_{25}FN_4O_4$ (458.53): C, 57.80; H, 4.12; N, 14.30; S, 4.68; Found: C, 57.96; H, 4.23; N, 14.52; S, 4.58.

7-(5-(4-(4-chlorobenzylideneamino)phenyl)-1,3,4-oxadiazole-2-thiol)acetyl)piperazin-1-yl)-1-ethyl-6-fluoro-1,4-dihydro-4-oxoquinoline-3-carboxylic acid 13c

Yield= 0.31 g (45%); yellow powder, mp:288-290 °C; ^{1}HNMR (400 MHz, DMSO-d6) (ppm): 8.39 (br. s, 2H), 7.94 (d, J_{H-F} = 12.9 Hz, 1H, H-5), 7.83 (m, 2H), 7.63 (d, J= 8.2 Hz, 2H), 7.43 (m, 2H), 7.03 (m, 3H), 4.62 (s, 2H), 4.31 (q, J = 4.8, 9.5 Hz, 2H), 3.61-3.54 (m, 4H), 3.50 (m, 4H), 1.35 (t, J = 5.1 Hz, 3H). ^{13}C NMR (100 MHz, DMSO-d$_6$): 170.5, 168.2, 167.2, 159.7, 157.9, 156.2, 154.4, 149.2, 147.7, 146.1, 141.1, 136.8, 132.0, 130.1, 129.7, 128.3, 117.6, 114.2, 111.9, 104.5, 50.3, 48.1, 45.6, 38.3, 14.9. Anal. Calcd for $C_{33}H_{28}ClFN_6O_5S$ (675.13): C, 58.71; H, 4.18; N, 12.45; S, 4.75; Found: C, 58.94; H, 4.32; N, 12.71; S, 4.80.

Tab. S14: Yield and reaction time of target compounds.

**Code**	Yield (%)	Time (hr)	Code	Yield (%)	Time (hr)
**DMF, K_2CO_3 and dioxane, K_2CO_3 method**					
**4a**	52%	13	**4l**	59%	16
**4b**	60%	12	**3b**	50%	21
**4c**	57%	12.5	**3a**	44%	22
**4d**	49%	13	**16**	50%	17
**4e**	70%	20	**5a**	59%	13.5
**4f**	53%	17	**5b**	54%	18
**4g**	62%	14	**4i**	62%	12
**4k**	54%	15	**4j**	52%	24
**4h**	71%	14.5	**3c**	55%	18
**Acetonitrile, Et_3N method**					
**7a**	89%	12	**8b**	77%	9
**7b**	91%	12	**14**	53%	8
**6a**	83%	11	**8a**	72%	8.5
**6b**	75%	10			

Text S11: Antimicrobial activity

Bacterial strains and growth conditions

Bacterial strains used in this study are listed in **Tab S14**. *E. coli, S. aureus*, and *B. subtilis* strains were grown in Mueller Hinton broth, *P. aeruginosa* in cation-adjusted Muller Hinton II, and *M. tuberculosis* in Middlebrook 7H9 medium. *M. tuberculosis* was grown at 30 °C, all other strains at 37 °C. All cultures were grown under constant shaking. Expression of NeonGreen-GlpT in *E. coli* BCB472 was induced by addition of 20 µM isopropyl β- d-1-thiogalactopyranoside (IPTG) for 60 min. *B. subtilis* strains expressing GFP fusion proteins were constantly grown in the presence of 0.05% (TNVS175) or 0.1% xylose (all other strains) to maintain level expression of the fusion proteins. Unless otherwise stated, experiments were performed in triplicates.

Minimal inhibitory concentrations

Minimal inhibitory concentrations against *E. coli, P. aeruginosa, S. aureus,* and *B. subtilis* were performed in a microdilution protocol according to CLSI guidelines as described previously (6, 7). Antimicrobial activity against *M. tuberculosis* was tested using a modified protocol according to Schön *et al*. (8). Reported MIC values represent the average of duplicate experiments. The compounds most active against *E. coli* W3110, *S. aureus* 1800T, and *M. tuberculosis* MC26020 of each series were chosen for mode of action studies.

Tab S14: Strains used in this study. i. a. = if applicable, *mgfp* = monomeric green-fluorescent protein, *msfgfp* = monomeric superfolder green-fluorescent protein, #Ciprofloxacin=R, *Nitrofurantoin=R, Cefadroxil=R, Penicillin G/V=R, Isoxa-pc=R, Cefuroxim=R, Cefotaxim=R, Ceftazidim=R, Imipenem=R, Tobramycin=R, Trim-Sulfa=R, Norfloxacin=R, Ciprofloxacin=R, Clindamycin=R, Fusidic acid=S, Vancomycin=S, Netilmic=R

**Species and strain**	**Relevant genotype**	**Reference**
E. coli W3110	*F-, IN(rrnD-rrnE)1*	https://doi.org/10.13145/bacdive4747.20201210.5
*E. coli**		clinical resistant isolate
E. coli BCB472	*psav057-NeonGreen-2GS-GlpT*	[30]
K. pneumoniae ATCC10031		doi:10.13145/bacdive4968.20220920.7
P. aeruginosa PAO1		doi.org/10.13145/bacdive12801.20201210.5
S. aureus CCUG1800T		doi.org/10.13145/bacdive14487.20201210.5
S. aureus ATCC43300#		doi:10.13145/bacdive14464.20220920.7
M. tuberculosis MC26020	Δ*lysA* Δ*panCD*	[31]
B. subtilis DSM402	*trpC2*	doi.org/10.13145/bacdive1156.20201210.5
B. subtilis EKB46	*trpC2 amyE::spc Pxyl-msfgfp*	[32]
B. subtilis TNVS45	*trpC2 amyE::spc Pxyl-mgfp-ponA*	[32]
B. subtilis TNVS175	*trpC2 amyE::spc Pxyl-murG-msfgfp*	[32]
B. subtilis TNVS284	*trpC2 amyE::spc Pxyl-mraY-msfgfp*	[32]

#Ciprofloxacin = R
* Nitrofurantoin=R, Cefadroxil=R, Penicillin G/V=R, Isoxa-pc=R, Cefuroxim=R, Cefotaxim=R, Ceftazidim=R, Imipenem=R, Tobramycin=R, Trim-Sulfa=R, Ciprofloxacin=R, Clindamycin=R, Fusidic acid=S, Vancomycin=S, Netilmic=R

Text S12: Molecular modeling

Optimization of target compounds

The target ligands for modelling were built using the builder interface of the MOE software package 2020.01 and subjected to conformational search. Conformers were optimized by energy minimization until a RMSD gradient of 0.01 Kcal/mol and RMS distance of 0.1 Å with MMFF94X force-field and the partial charges were automatically calculated. The obtained output was then saved as MDB file to be used in the molecular modelling.

Calculation of physicochemical properties

Calculation of physicochemical properties for target compounds including: AM1_dipole (AM1), water accessible surface area (ASA), lipiniski acceptor count (lip_acc), lipiniski donor count (lip_don), lipiniski druglike test (lip_druglike), log octanol/water partition coefficient (logP(o/w)), log solubility in water (logS), topological polar surface area (TPSA), van der waals surface area (VSA), molecular weight (weight) and number of rotatable bonds (nrotb) which performed as the following: From the database viewer of the target compounds, choose DBV | Compute | Descriptors | Calculate. Then choose the desired descriptors from calculate descriptors panel.

Docking study

Preparation of enzyme structure

Molecular docking and visualization were performed on target enzymes using Molecular Operating Environment (MOE) 2020.01. The x-ray crystal structure of S. aureus DNA gyrase complexed with moxifloxacin with UniProt accession ID: Q99XG5 (PDB code: 5CDQ)[23] and crystal structure of A. baumannii topoisomerase IV complexed with moxifloxacin UniProt accession ID: B0VP98 (PDB code: 2XKK)[33] were obtained from the RCSB Protein Data Bank. Next, enzyme structures were prepared by using the MOE QuickPrep protocol. The designed compounds were docked on target enzymes using the method of Alpha triangle placement with Amber10: EHT forcefield. Refinement was performed with Forcefield and scored using the Affinity dG scoring system.

QSAR model generation

Dataset preparation

The target ligands for QSAR were 36 synthesized norfloxacin derivatives which divided randomly into training set (24 compounds) and test set for validation (9 compounds) which built using the builder interface of the MOE software package 2020.01 and subjected to conformational search. Energy minimization was done to obtained conformers until a RMSD gradient of 0.01 Kcal/mol and RMS distance of 0.1 Å with MMFF94X force-field and the partial charges were automatically calculated. The obtained database was then saved as MDB file to be used in the QSAR model.

Calculation of molecular descriptors

Molecular Properties for 33 compounds that were calculated five descriptors (E_vdw, kierflex, LogP, TPSA). These descriptors described molecular structures, including geometrical, physicochemical, sterical and lipophilic properties that are strongly related to biological activity. The MIC values of the dataset were converted to Log MIC which used as the dependent variable. Then these compounds are divided randomly into a training set (24 compounds) and test set for validation (9 compounds).

QSAR model development

After selecting molecular descriptors, the linear QSAR models were built using the training set data using partial least square method (PLS) in the following steps: From the database viewer of the target compounds, choose DBV | Compute | Model | QSAR. Then choose Log MIC at activity field in QuaSAR-Model panel, then select the calculated descriptors that shown in the panel and finally choose fit to get the model.

Validation of QSAR model

The evaluation of the QSAR model is divided into internal and external validations. The parameters for internal validation were r^2 (a correlation coefficient), q^2 (predictive ability of the built QSAR models in the training set data employing leave-one-out (LOO) cross-validation method), and r^2 _pred (predictive ability for the test set). QSAR model is selected if it complies with the three criteria: the values of the high correlation coefficient (r^2) between the experimental and the predicted values, the predictive ability of the model for the training set $q^2>0.5$, and the low standard deviation (RMSE). If the created model complies with these criteria, that means it is reliable and has good predictive ability[34].

Prediction the Log MIC of test set compounds

The linear QSAR model was used to predict the Log MIC values of synthesized norfloxacin derivatives as the following steps: From the database viewer of the target compounds, choose DBV | Compute | Model |Evaluate. Then choose the QSAR model at Model field in Model-Evaluate panel, and finally choose ok to get the results.

in silico ADME/Tox profile of the new compounds

Two ADME/Tox web tools were used in the predictive study:

1.pkCSM-pharmacokinetics(http://biosig.unimelb.edu.au/pkcsm/prediction)[9] web tool was used in this predictive study which is a new method for predicting and optimizing small-molecule ADME/Tox properties.

2.SwissADME web tool (http://www.swissadme.ch/)[8] which represents a recent and relevant computational method to evaluate the pharmacokinetics profile of small molecules.

The molecular structures of the new compounds and norfloxacin were built on ChemDraw Ultra 8.0, then copied as SMILES (simplified molecular-input line-entry specification) nomenclature then pasted into the used web tools, pkCSM-pharmacokinetics and SwissADME. The most important ADME/Tox properties provided from the web tools were selected to represent the ADME/Tox profile.

Text S13: Cytotoxicity studies

Cell viability, in terms of mitochondrial metabolic function, was evaluated by the reduction of 3-(4,5-dimethyl-2-thiazolyl)-2,5-diphenyl-2Htetrazolium bromide (MTT) to its insoluble formazan. Briefly, neuronal SH-SY5Y cells were seeded in a 96-well plate at 2 × 104 cells per well. Subsequently, SH-SY5Y cells were treated for 24 h with different concentrations of the studied compounds **4a** and **4e** (2.5 – 80 μM). Then the treatment medium was replaced with MTT solution (0.5 mg ml^{-1}) in Hank's Balanced Salt Solution (HBSS) for 2 h at 37°C in 5% CO2. After washing with HBSS, formazan crystals were dissolved in isopropanol. The amount of formazan was measured (ʎ = 570 nm, reference filter 690 nm) using a multilabel plate reader (VICTOR™ X3, PerkinElmer, Waltham, MA, USA) and an Anthos Zenyth 200rt microplate reader (Biochrom, UK). The cytotoxicity of the test compound was obtained using the following formula: [(A − B)/A × 100], where A represents the absorbance of untreated cells and B the absorbance of cells treated with different concentrations of the test compounds. Cytotoxic concentration for 50% of cells was determined by linear regression.

Text S14: Mode of action studies

***in vitro* inhibition of DNA gyrase and topoisomerase IV**

The cleavage assay has been carried out at the confirmatory diagnostic R&D sector (Vacsera-Egypt) using E. coli DNA gyrase and Topoisomerase IV cleavage Assay Kits (Inspiralis®). These kits are designed specifically for cleavage reactions. They contain DNA gyrase and Topo IV enzyme, supercoiled pBR322 DNA substrate and the Assay and Dilution buffers required for DNA cleavage reactions [35]. The most active compounds on *E. coli* with lower MIC values selected to be tested on DNA gyrase, while the most active ones on *S. aureus* were selected to be tested on Topo IV enzyme. Thirteen dilutions of each compound were prepared (0.05, 0.1, 0.25, 0.5, 1, 5, 10, 25, 50, 100, 250, 500, 1000 µM) using DMSO as solvent. IC50 values were determined at a final concentration of 0.1, 1, 10, and 100 µM. Norfloxacin was used as a reference drug.

Bacterial cytological profiling

Bacterial cytological profiling was performed using *E. coli* W3110, *E. coli* BCB472, and *B. subtilis* DSM402. *E. coli* W3110 and *B. subtilis* DSM402 were grown until an OD_{600} of 0.3 prior to treatment with 1xMIC of the respective compounds for 60 min. Compounds that did not show a clear gyrase inhibition phenotype at 1x MIC were additionally tested at 2x and 4x MIC to ensure that they do not inhibit gyrase at higher concentrations. After antibiotic treatment, samples were stained with 1 µM FM6-64 and 1 µM DAPI for 5 min. Samples were then spotted on agarose-covered microscopy slides as described previously (9). *E. coli* BCB472 was grown until an OD_{600} of 0.3 prior to addition of 10 µM isopropyl β-D-1-thiogalactopyranoside (IPTG) and the respective compounds as described above. After 60 min, samples were withdrawn and spotted on agarose-covered glass slides. All microscopy samples were observed with a Nikon Eclipse Ti2 equipped with a CFI Plan Apochromat DM Lambda 100X Oil objective (N.A. 1.45, W.D. 0.13mm), a Photometrics PRIME BSI camera, a Lumencor Sola SE II FISH 365 light source, and an Okolab temperature incubation chamber. Images were obtained using the NIS elements AR software version 5.21.03 and analyzed with ImageJ (10).

Outer membrane permeability

Outer membrane integrity was tested by assessing synergy with mupirocin, which is able to inhibit *E. coli* isoleucine tRNA synthase but displays poor minimal inhibitory concentrations against this

bacterium, because it cannot pass its outer membrane (11). Weakening of the outer membrane barrier, e. g. with polymyxin B nonapeptide, results in strongly increased mupirocin activity, which can be assessed with checkerboard assays to determine synergistic interactions (12). The fractional inhibitory concentration index was calculated according to the formula FICI = $(MIC_A^{combi}/MIC_A^{alone}) + (MIC_B^{combi}/MIC_B^{alone})$. FICI values of ≤0.5 were defined as synergy, >0.5 to ≤4.0 as additive (no interaction), and >4.0 was defined as antagonism. Checkerboard assays were performed in duplicate.

Peptidoglycan integrity assay

Peptidoglycan integrity was tested with an established acetic acid/methanol fixation protocol using *B. subtilis* DSM402 (13, 14). In short, *B. subtilis* was grown until an OD_{600} of 0.3, treated with 1x MIC of the respective antibiotics for 30 (vancomycin, D-cycloserine, fosfomycin, tunicamycin) or 60 min (all other compounds) and subsequently fixed in a 5-fold excess of a 1:3 mixture of acetic acid and methanol. This treatment leads to extrusion of the protoplast through cell wall breaches, appearing as 'bubbles' on the cell surface, which are indicative of compromised peptidoglycan integrity (13). Cultures treated with cell wall synthesis inhibitors were imaged after shorter treatment times, since prolonged treatment causes pronounced cell lysis leading to major disintegration of cells during fixation. Fixed samples were observed by phase contrast microscopy as described above. For quantification, the total number of cells and total number of bubbles were counted and expressed as ratio of bubbles/total cell number. A minimum of 50 cells were counted per replicate per condition.

Membrane protein localization

For protein localization experiments, *B. subtilis* EKB46, TNVS45, TNVS175, and TNVS284 (background for all strains is DSM402) were grown until an OD_{600} of 0.3 prior to treatment with 1x MIC of the respective compounds for 30 (vancomycin) or 60 min (all other compounds). Cultures treated with cell wall synthesis inhibitors were imaged after shorter treatment times, since prolonged treatment causes pronounced cell lysis leading to unspecific protein delocalization. Where heterogenous effects were observed, phenotypes were classified and cells per classified phenotype were counted. A minimum of 50 cells were counted per replicate per condition.

Text S15: HPLC analysis

HPLC analysis was carried out on a KNAUER HPLV pump 64 equipped with a reversed phase HPLC column (25 × 0.5 cm), a KNAUER VARIABLE wavelength detector, a Shimadzu c-r 6A chromatopac recording integrator, and a 20 μL injection loop. The stainless steel column packed was with octa silyl silica at 25 ºC. The mobile phase was composed of 250 volumes of methanol containing 1.15 g of 40% w/v solution of tetrabutylammonium hydroxide, 375 volumes of 0.05 M disodium hydrogen orthophosphate, and 375 volumes of 0.05 M sodium hydrogen orthophosphate. Solutions of compounds **4a**, **4e**, and **21** were prepared by dissolving 0.001 g of each substance in 10 mL of the mobile phase in a 10 mL volumetric flask aided by ultrasonication to obtain 0.01 % (w/v) solution. Samples were run at a flow rate of 1 mL/min. Peaks were detected at 278 nm and chromatography was continued for 12 times the retention time of the principal peak.

Text S16: References

(1) Gedeck, P.; Kramer, C.; Ertl, P. 4 - Computational Analysis of Structure–Activity Relationships. In *Progress in Medicinal Chemistry*, Lawton, G., Witty, D. R. Eds.; Vol. 49; Elsevier, 2010; pp 113-160.
(2) N. Ramalakshmi, S. A. a. S. B. QSAR and lead optimization. *Computer Applications in Drug Discovery and Development* **2019**, pp. 80–100.
(3) Tandon, H.; Ranjan, P.; Chakraborty, T.; Suhag, V. Polarizability: a promising descriptor to study chemical-biological interactions. *Mol. Divers.* **2021**, *25* (1), 249-262. DOI: 10.1007/s11030-020-10062-w From NLM.
(4) Zhou, Z.; Wang, Y.; Bryant, S. H. QSAR models for predicting cathepsin B inhibition by small molecules--continuous and binary QSAR models to classify cathepsin B inhibition activities of small molecules. *J. Mol. Graph. Model.* **2010**, *28* (8), 714-727. DOI: 10.1016/j.jmgm.2010.01.009 From NLM.
(5) Pratim Roy, P.; Paul, S.; Mitra, I.; Roy, K. On two novel parameters for validation of predictive QSAR models. *Molecules* **2009**, *14* (5), 1660-1701. DOI: 10.3390/molecules14051660 From NLM.
(6) Veerasamy, R.; Rajak, H.; Jain, A.; Sivadasan, S.; Christapher, P. V.; Agrawal, R. Validation of QSAR Models - Strategies and Importance. *Int J Drug Design and Discov* **2011**, *2*, 511-519.
(7) Durán-Iturbide, N. A.; Díaz-Eufracio, B. I.; Medina-Franco, J. L. In Silico ADME/Tox Profiling of Natural Products: A Focus on BIOFACQUIM. *ACS Omega* **2020**, *5* (26), 16076-16084. DOI: 10.1021/acsomega.0c01581.
(8) http://www.swissadme.ch/. **Accessed at January 12,. 2022.** .
(9) http://biosig.unimelb.edu.au/pkcsm/prediction. **Accessed at January 8,. 2022.**
(10) Pires, D. E.; Blundell, T. L.; Ascher, D. B. pkCSM: Predicting Small-Molecule Pharmacokinetic and Toxicity Properties Using Graph-Based Signatures. *J Med Chem* **2015**, *58* (9), 4066-4072. DOI: 10.1021/acs.jmedchem.5b00104 From NLM.
(11) O'Hagan, S.; Kell, D. B. The apparent permeabilities of Caco-2 cells to marketed drugs: magnitude, and independence from both biophysical properties and endogenite similarities. *PeerJ* **2015**, *3*, e1405. DOI: 10.7717/peerj.1405 From NLM.
(12) https://readycell.com/caco-2-permeability-protocol/. **Accessed at January 5,. 2022.**
(13) Yates, J.; Arundel, P. On the Volume of Distribution at Steady State and Its Relationship With Two-Compartmental Models. *Journal of Pharmaceutical Sciences* **2008**, *97*, 111-122. DOI: 10.1002/jps.21089.
(14) Mansoor, A.; Mahabadi, N. Volume of Distribution. In *StatPearls*, StatPearls Publishing Copyright © 2021, StatPearls Publishing LLC., 2021.
(15) Carpenter, T. S.; Kirshner, D. A.; Lau, E. Y.; Wong, S. E.; Nilmeier, J. P.; Lightstone, F. C. A method to predict blood-brain barrier permeability of drug-like compounds using molecular dynamics simulations. *Biophysical journal* **2014**, *107* (3), 630-641. DOI: 10.1016/j.bpj.2014.06.024 From NLM.
(16) Bardal, S. K.; Waechter, J. E.; Martin, D. S. Chapter 2 - Pharmacokinetics. In *Applied Pharmacology*, Bardal, S. K., Waechter, J. E., Martin, D. S. Eds.; W.B. Saunders, 2011; pp 17-34.
(17) Collins, J. M. Chapter 46 - Pharmacokinetics, Pharmacodynamics, and Pharmacogenetics1. In *The Molecular Basis of Cancer (Third Edition)*, Mendelsohn, J., Howley, P. M., Israel, M. A., Gray, J. W., Thompson, C. B. Eds.; W.B. Saunders, 2008; pp 547-552.

(18) 2020 Mar 10]. Available from: https://www.ncbi.nlm.nih.gov/books/NBK547850/, L. C. a. R. I. o. D.-I. L. I. I. B. M. N. I. o. D. a. D. a. K. D.-N. U. **Accessed at January 6,. 2022**. Adikwu, E. Fluoroquinolones Reported Hepatotoxicity. *Pharmacology & Pharmacy* **2012**, *03*, 328-336. DOI: 10.4236/pp.2012.33044.
(19) Jain, A. K.; Singh, D.; Dubey, K.; Maurya, R.; Mittal, S.; Pandey, A. K. Chapter 3 - Models and Methods for In Vitro Toxicity. In *In Vitro Toxicology*, Dhawan, A., Kwon, S. Eds.; Academic Press, 2018; pp 45-65.
(20) Veber, D. F.; Johnson, S. R.; Cheng, H.-Y.; Smith, B. R.; Ward, K. W.; Kopple, K. D. Molecular Properties That Influence the Oral Bioavailability of Drug Candidates. *Journal of Medicinal Chemistry* **2002**, *45* (12), 2615-2623. DOI: 10.1021/jm020017n.
(21) Taylor, J. B. T. D. J. Comprehensive medicinal chemistry II. **2007**. From http://worldcat.org /z-wcorg/.
(22) Zerroug, A.; Belaidi, S.; BenBrahim, I.; Sinha, L.; Chtita, S. Virtual screening in drug-likeness and structure/activity relationship of pyridazine derivatives as Anti-Alzheimer drugs. *Journal of King Saud University - Science* **2019**, *31* (4), 595-601. DOI: https://doi.org/10.1016/j.jksus.2018.03.024.
(23) https://www.rcsb.org/structure/5CDQ. **(Accessed at March 27,. 2022)**.
(24) Ferkous, F.; Mebrouk, D.; Benalia, M.; Boudaoud, A.; Al-Dujaili, A. *Study of Some Alkanes Thermodynamic Parameters Using New Liquid Crystals Containing Sulfur as Stationary Phases*; 2015. DOI: 10.13140/RG.2.1.4363.1208.
(25) Karunakaran, C.; Venkataramanan, R. Conversion of anilines into azobenzenes in acetic acid with perborate and Mo(VI): correlation of reactivities. *Chemical Papers* **2019**, *73* (2), 375-385. DOI: 10.1007/s11696-018-0599-z.
(26) Kshash, A. H. The Effect of Hydrogen Bonding and Azomethine Group Orientation on Liquid Crystal Properties in Benzylidene Aniline Compounds. *Acta chimica Slovenica* **2020**, *67* (3), 739-747. From NLM.
(27) Cao, C.; Lu, B.; Chen, G. Investigation of the substituent specific cross-interaction effects on 13C NMR of the C≡N bridging group in substituted benzylidene anilines. *Journal of Physical Organic Chemistry* **2011**, *24*, 335-341. DOI: 10.1002/poc.1760.
(28) Khan, K.; Khan, K.; Siddiqui, R.; Ambreen, N.; Sultana, N.; Tauseef, S.; Ahmad, A.; Perveen, S.; Dr, P.; Khan, H. Synthesis, antibacterial and antifungal evaluation of norfloxacin derivatives. *journal of pharmacy research* **2012**, *55*, 92.
(29) Marc, G.; Araniciu, C.; Oniga, S. D.; Vlase, L.; Pîrnău, A.; Nadăș, G. C.; Novac, C. Ș.; Matei, I. A.; Chifiriuc, M. C.; Măruțescu, L.; et al. Design, Synthesis and Biological Evaluation of New Piperazin-4-yl-(acetyl-thiazolidine-2,4-dione) Norfloxacin Analogues as Antimicrobial Agents. *Molecules (Basel, Switzerland)* **2019**, *24* (21). DOI: 10.3390/molecules24213959.
(30) Liu X, M. N., Bouhss A, den Blaauwen T. FtsW activity and lipid II synthesis are required for recruitment of MurJ to midcell during cell division in Escherichia coli. . *Mol Microbiol* **2018**, *109*, 855–884.
(31) Larsen MH, B. K., Chen B, Hsu T, Sambandamurthy VK, Lackner AA, Aye PP, Didier P, Huang D, Shao L, Wei H, Letvin NL, Frothingham R, Haynes BF, Chen ZW, Jacobs Jr WR. Efficacy and safety of live attenuated persistent and rapidly cleared Mycobacterium tuberculosis vaccine candidates in non-human primates. . *Vaccine* **2009**, *27*, 4709–4717.
(32) Müller, A.; Wenzel, M.; Strahl, H.; Grein, F.; Saaki, T. N. V.; Kohl, B.; Siersma, T.; Bandow, J. E.; Sahl, H. G.; Schneider, T.; et al. Daptomycin inhibits cell envelope synthesis by interfering with fluid membrane microdomains. *Proceedings of the National Academy of*

Sciences of the United States of America **2016**, *113* (45), E7077-e7086. DOI: 10.1073/pnas.1611173113 From NLM.
(33) https://www.rcsb.org/structure/2XKK. **(Accessed at March 23,.2022)**.
(34) Nguyen, P. T. V.; Van Dat, T.; Mizukami, S.; Nguyen, D. L. H.; Mosaddeque, F.; Kim, S. N.; Nguyen, D. H. B.; Đinh, O. T.; Vo, T. L.; Nguyen, G. L. T.; et al. 2D-quantitative structure-activity relationships model using PLS method for anti-malarial activities of anti-haemozoin compounds. *Malaria journal* **2021**, *20* (1), 264. DOI: 10.1186/s12936-021-03775-2 From NLM.
(35) Burrell, M. R.; Burton, N. P.; Maxwell, A. A high-throughput assay for DNA topoisomerases and other enzymes, based on DNA triplex formation. *Methods in molecular biology (Clifton, N.J.)* **2010**, *613*, 257-266. DOI: 10.1007/978-1-60327-418-0_16 From NLM.
